# Evidence‐based ranking of diabetes prevention in prediabetes: A comprehensive network meta‐analysis of 378 randomised controlled trials

**DOI:** 10.1002/ctm2.70829

**Published:** 2026-09-08

**Authors:** Bucur Maria, Eperjesi Orsolya, Panait Robert Iulian, Topala Mihaela, Babakhani Avin Aphrodite, Rancz Anett, Kói Tamás, Fazekas Karen, Obeidat Mahmoud, Papp Renáta, Ferdinándy Péter, Szentesi Andrea, Gheorghe Cristian, Hegyi Peter, Bunduc Stefania

**Affiliations:** ^1^ Centre for Translational Medicine Semmelweis University Budapest Hungary; ^2^ ‘Carol Davila’ University of Medicine and Pharmacy Bucharest Romania; ^3^ Department of Internal Medicine Toldy Ferenc Hospital Cegléd Hungary; ^4^ Institute of Pancreatic Diseases Semmelweis University Budapest Hungary; ^5^ Department of Neurology Semmelweis University Budapest Hungary; ^6^ János Szentágothai Neurosciences Division Semmelweis University Doctoral School Budapest Hungary; ^7^ Department of Stochastics Institute of Mathematics Budapest University of Technology and Economics Budapest Hungary; ^8^ Department of Pharmacology and Pharmacotherapy Semmelweis University Budapest Hungary; ^9^ Center for Pharmacology and Drug Research and Development Semmelweis University Budapest Hungary; ^10^ Pharmahungary Group Szeged Hungary; ^11^ Digestive Disease and Liver Transplant Center Fundeni Clinical Institute Bucharest Romania; ^12^ Institute for Translational Medicine Medical School University of Pécs Pécs Hungary

**Keywords:** diabetes, impaired fasting glucose, impaired glucose tolerance, insulin resistance, pancreas, prediabetes

## Abstract

**Background:**

Ten percent of the global population presents impaired glucose tolerance. Although numerous interventions have been investigated to prevent type 2 diabetes (T2D), their comparative effectiveness remains uncertain. In this study, we assessed and ranked current pharmacological and non‐pharmacological interventions for preventing diabetes in individuals with prediabetes.

**Methods:**

The study protocol was registered on PROSPERO (CRD42023473875). Randomised controlled trials (RCTs) reporting on adult patients with prediabetes undergoing any intervention addressing dysglycaemia were eligible. Our systematic search was conducted from database inception to 19 December 2025 in three databases (MEDLINE, Embase and Cochrane Library [CENTRAL]). Network meta‐analysis was performed using the random‐effect frequentist method. Our primary outcomes were the incidence of T2D, haemoglobin A1c (HbA1c), fasting plasma glucose (FPG) and 2‐h glucose after the glucose oral tolerance test (2hPG) expressed as relative risk (RR) and mean difference (MD), respectively, with the corresponding 95% confidence intervals (CIs).

**Results:**

We included 378 RCTs comprising 113 122 patients. The highest risk reduction for T2D when compared with control was obtained with dual incretin‐based therapy (glucagon‐like peptide 1 [GLP‐1] and gastric inhibitory polypeptide (GIP) receptor agonists [GLP‐1 + GIP RAs]) (RR = .1, CI [.05;  .21]), with GLP‐1 receptor agonists (GLP‐1 RAs) ranking second (RR = .26, CI [.19;  .4]) followed closely by metformin plus intensive lifestyle intervention (ILI) (RR = .27, CI [.12;  .64]). Only curcumin (MD = −.49, CI [−.70; −.28]), dual GLP‐1 + GIP RA (MD = −.46, CI [−.63; −.29]), single GLP‐1 RA (MD = −.41, CI [−.50; −.31]) and physical activity (MD = −.35, CI [−.48; −.23]) achieved >.3% HbA1c reductions after 1 year. Sulphonylureas and physical activity were most effective for the 1‐year FPG decrease, while the greatest 2hPG reduction was obtained with zinc supplementation and sulphonylureas.

**Conclusion:**

Incretin‐based therapies, particularly dual GLP‐1 + GIP RA and single GLP‐1 RA, followed by metformin together with ILI, are the most effective interventions to prevent T2D in patients with prediabetes.

## Graphical abstract

• We analysed 378 RCTs (113 122 participants).

• Dual GLP‐1/GIP agonists reduced T2D risk by 90%, followed by GLP‐1 agonists (74%) and metformin plus ILI (73%).

• Physical activity and ILI improved glycaemic markers at 12 months. Lifestyle benefits peaked at 3–6 months, whereas metformin effects appeared longer‐lasting.

• Lifestyle modifications remain central to T2D prevention, and can be complemented by pharmacotherapy.

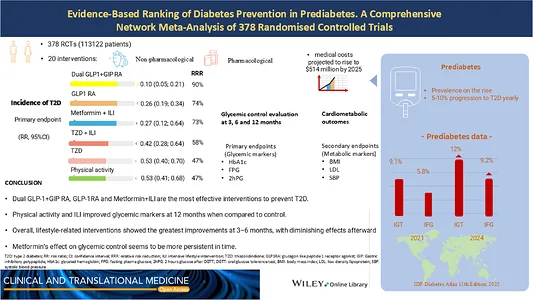

## 1 BACKGROUND

In 2024, 12% of the adult population (634.8 million) had impaired glucose tolerance (IGT), and 9.2% (487.7 million) had impaired fasting glucose (IFG); these conditions are experiencing a rising incidence.^1^ Moreover, 5%–10% of prediabetes cases will progress to type 2 diabetes mellitus (T2D) yearly.^2^ Currently, diabetes places a significant economic strain on healthcare systems, with medical costs projected to rise to $514 million in 2025. Beyond the risk of diabetes itself, individuals with prediabetes are also at increased risk for microvascular and macrovascular complications, including atherosclerosis and chronic kidney disease.^3^ Management of prediabetes and prevention of diabetes represent, therefore, a global healthcare concern.

Multiple landmark randomised controlled trials^4^, ^5^, ^6^ (RCTs) have demonstrated that intensive lifestyle and behavioural interventions, featuring individualised reduced‐calorie meal plans and exercise, are highly effective in preventing or delaying T2D and improving various cardiometabolic markers. According to Standards of Care in Diabetes—2025, an intensive lifestyle behaviour change program should be implemented in patients with prediabetes to achieve and maintain a weight reduction of at least 7% of the initial body weight. Certified technology‐assisted diabetes prevention programs may be effective in preventing T2D and should be considered as a viable option. Metformin is recommended as the therapeutic option for patients at high risk for progression to diabetes and is cost‐effective.^7^, ^8^ Glucagon‐like peptide 1 receptor agonists (GLP‐1 RAs) and sodium glucose co‐transporter 2 inhibitors (SGLT2i) have shown highly promising results.^9^, ^10^, ^11^, ^12^

To date, no pharmacological agent has been approved by the U.S. Food and Drug Administration specifically for the prevention of T2D.

Given the challenges of maintaining long‐term lifestyle modifications, the increasing prevalence of prediabetes, the high costs associated with diabetes care and the availability of new medications such as the GLP‐1 RAs, our network meta‐analysis aims to identify the most effective intervention for preventing or delaying diabetes in individuals with prediabetes.

## 2 METHODS

### 2.1 Search strategy and selection criteria

We report our systematic review and meta‐analysis based on the recommendation of the Preferred Reporting Items for Systematic Reviews and Meta‐Analyses (PRISMA) 2020 guideline^13^ (Supporting Information S1). During our research, we followed the recommendations of the Cochrane Handbook for Systematic Reviews.^14^ The study protocol was registered on PROSPERO (CRD42023473875). This study was conducted within the Systems Education Program at Semmelweis University.^15^

To establish the inclusion criteria, we used the PICO framework. Only RCTs reporting on adults with prediabetes (P), who had not previously received treatment and were undergoing an intervention (I) targeting dysglycaemia, were eligible. Eligible interventions included pharmacological therapies, lifestyle interventions (dietary modification and/or physical activity), education, nutritional supplements and their combinations. Prediabetes had to be diagnosed according to the latest American Diabetes Association (ADA) criteria (IFG 100–125 mg/dL [5.6–6.9 mmol/L], IGT 140–199 mg/dL [7.8–11.0 mmol/L] or haemoglobin A1c [HbA1c] 5.7–6.4% [39–47 mmol/mol]), although slight differences were also accepted. If the homeostasis model assessment of estimated insulin resistance (HOMA‐IR > 1.9) was mentioned among these criteria, the paper was still included. All control, placebo and standard care arms were grouped under a unified ‘control’ category. These control interventions shared the characteristic of not including an active diabetes‐prevention strategy beyond routine clinical practice. Our primary outcomes were the incidence of T2D and the change in HbA1c, fasting plasma glucose (FPG) and glucose after the oral glucose tolerance test (2hPG). The secondary outcomes were changes in body mass index (BMI), low‐density lipoprotein (LDL) and systolic blood pressure (SBP).

We excluded studies involving prediabetic patients with comorbid HIV or schizophrenia, as well as those focusing on prediabetic pregnant women, given that specific diagnostic criteria for prediabetes in pregnancy are not established, studies evaluating Chinese or alternative medicine, herbal supplements or medications no longer in clinical use (e.g., troglitazone).

Our systematic search was conducted from database inception to 8 November 2023 across three databases: MEDLINE (via PubMed), Embase and Cochrane Central Register of Controlled Trials (CENTRAL) without the use of filters or restrictions. The main domains of the search strategy were: prediabetes, diabetes, prevention and random. The literature search was updated on 19 December 2025. For the detailed search key and protocol, see Supporting Information S2.

The search results were imported into EndNote21 for citation management (Clarivate, 2023). Using the software, we first automatically removed duplicate records, followed by a manual check by a single investigator (B.M.). Two independent reviewers (B.M. and E.O.) then screened the remaining records according to predetermined criteria by title and abstract, using Rayyan web software (Rayyan, 2016^16^), and calculated the Cohen's kappa coefficient (*κ*) to assess the inter‐reviewer agreement. Disagreements were settled by consensus among team members and through third‐party arbitration when consensus could not be reached (B.S.).

The full‐text selection was conducted in the same manner, except that any exclusions made at this point were documented (Table S1A). Librarians, first and/or last authors, were contacted by email when the primary reviewers were unable to access the records using the available sources. The full text selection was performed by two teams of two independent reviewers (B.M., E.O., P.R. and T.M.), each team working on half of the full text pool. Studies reporting on the same population were identified, and if they overlapped in exposure and outcome but reported different follow‐up time points, they were all included. Otherwise, the latest article published was included. Additional articles were identified from the reference lists of primarily eligible studies, using Citationchaser web software (Citationchaser, 2021) on 24 May 2024.

### 2.2 Data analysis

From the eligible articles, data were collected into a standardised Excel sheet (Microsoft Corporation, 2018) by two teams of two independent reviewers (B.M., E.O., P.R. and T.M.).

The following data were extracted: first author, the year of publications, DOI, study period, prediabetes diagnosis criteria, comorbidities, patient demographics (for the entire population and for the control and intervention group if available), type of intervention/control and continuous outcomes values at baseline and follow‐up (FPG, 2hPG, HbA1c, LDL, BMI and SBP—data in mmol/L and % for HbA1c). For the incidence of T2D, the total number of patients and those with the event of interest (T2D development) in each group were extracted if available; otherwise, odds ratios (ORs), hazard ratios (HRs), absolute/cumulative incidences or incidence rates were extracted to derive or estimate the required event counts whenever possible.

Two teams of two independent reviewers (B.M., E.O., P.R. and T.M.) performed the risk of bias assessment using the RoB 2 tool.^17^ Risk of bias analyses were conducted separately for each outcome. The level of evidence (LoE) was assessed using the CINeMA tool for the network analyses.^18^, ^19^

### 2.3 Synthesis methods

Statistical analyses were carried out using the meta, netmeta, dmetar and forestploter  packages of R (R Core Team 2021, v4.1.2). The confidence level (1 ‒ *α*) was set at 95%. All the performed analyses included random‐effect terms. The statistical analyses follow the advice of Harrer et al.^20^

To compare T2D incidences between intervention and control groups, we extracted or estimated (e.g., from the incidence rate and its standard error (SE) by using the formula given in Chapter 7 of Rothman^21^), for each study arm, the total number of patients with the event of interest—diabetes development—and the total number of patients in each study arm and performed frequentist network meta‐analysis. For the incidence of T2D, we extracted the longest follow‐up period reported in each study.

For the continuous outcomes, the effect size was represented as change from baseline. The mean and standard deviation (SD) of change from baseline values were extracted from each treatment arm. Often, SD of change was not directly available; in such cases, we calculated it using the SE or confidence interval (CI) of mean change. If neither of these was available, the SD of change was calculated from the before (baseline) and after (follow‐up) SD values and Pearson's correlation coefficient *r*. We imputed *r* = .6 correlation. Moreover, we performed a sensitivity analysis by imputing several different correlations (see the Cochrane Handbook, version 6.5, Chapter 6.5.2.8).^22^

In the network meta‐analysis, the mean difference (MD) of the differences from baseline for each treatment arm was used for ranking. In each network meta‐analysis, multi‐arm study correlations were taken into account. For each outcome, we created network graphs to visualise the comparisons present in the studies involved. The point and interval estimates for each intervention compared to a reference intervention were summarised in forest plots. Netsplit forest plots and the *p*‐values comparing direct and indirect estimates were used to detect inconsistency. We also performed a network meta‐analysis comparing the changes from baseline separately for the approximately 6‐month and 1‐year follow‐ups. We also performed sensitivity analyses by retaining only treatments present in at least three studies in the network.

Furthermore, for each continuous outcome and intervention type, we calculated the pooled change from baseline separately for each treatment arm at several follow‐up time intervals: 3 months, 6 months and 1 year. For this purpose, we used classical inverse variance meta‐analysis with REML tau estimator. We visualised the results on forest plots and summary plots. To assess heterogeneity, we indicated the *I* ^2^ statistic and its CI on the forest plots of simple (non‐network) analyses.

A total of 20 intervention classes were defined for the meta‐analysis. These included each major class of antidiabetic agents: metformin, GLP‐1 RAs monotherapy or dual therapy with gastric inhibitory polypeptide (GIP), SGLT2i, dipeptidyl peptidase‐4 (DPP‐4) inhibitors, alpha‐glucosidase inhibitors (AGIs), sulphonylureas (SUs) and thiazolidinediones (TZDs). Other reported pharmacologic interventions included vitamin D, zinc, L‐arginine, curcumin, antihypertensive drugs and lipid‐lowering agents. Furthermore, non‐pharmacologic interventions were also grouped as intensive lifestyle intervention (ILI), lifestyle intervention or education (LI/education), enhanced education, physical activity and dietary intervention. Additionally, studies were grouped based on follow‐up duration into approximately 3‐month, 6‐month, 1‐year and more than 1‐year categories. Details about the grouping can be found in Supporting Information S3.

Protocol deviations: Due to the large number of reporting studies, it was not feasible to analyse other secondary outcomes, such as waist circumference, total cholesterol, triglycerides and diastolic blood pressure.

## 3 RESULTS

**FIGURE 1:**
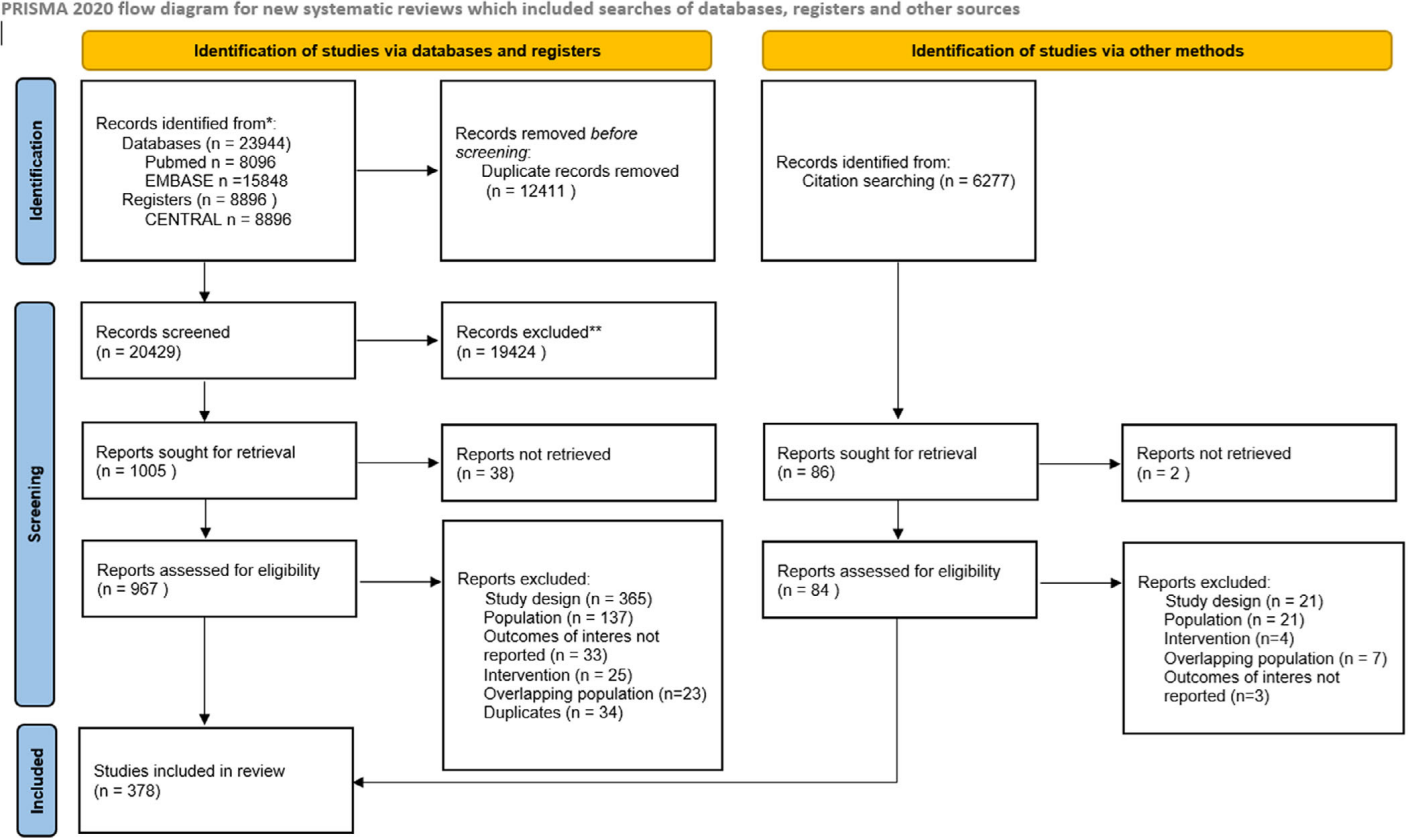
Preferred Reporting Items for Systematic Reviews and Meta‐Analyses (PRISMA) flowchart of study selection. The diagram outlines the identification, screening, eligibility assessment and inclusion of studies in the analysis, following the predefined selection criteria.

In total, 378 papers were eligible for the systematic review (Figure 1), of which 287 were included in the meta‐analysis. The baseline characteristics of the included studies are available in Table S1.

### 3.1 Ranking of the interventions for preventing diabetes in prediabetic population: a network meta‐analysis

**FIGURE 2:**
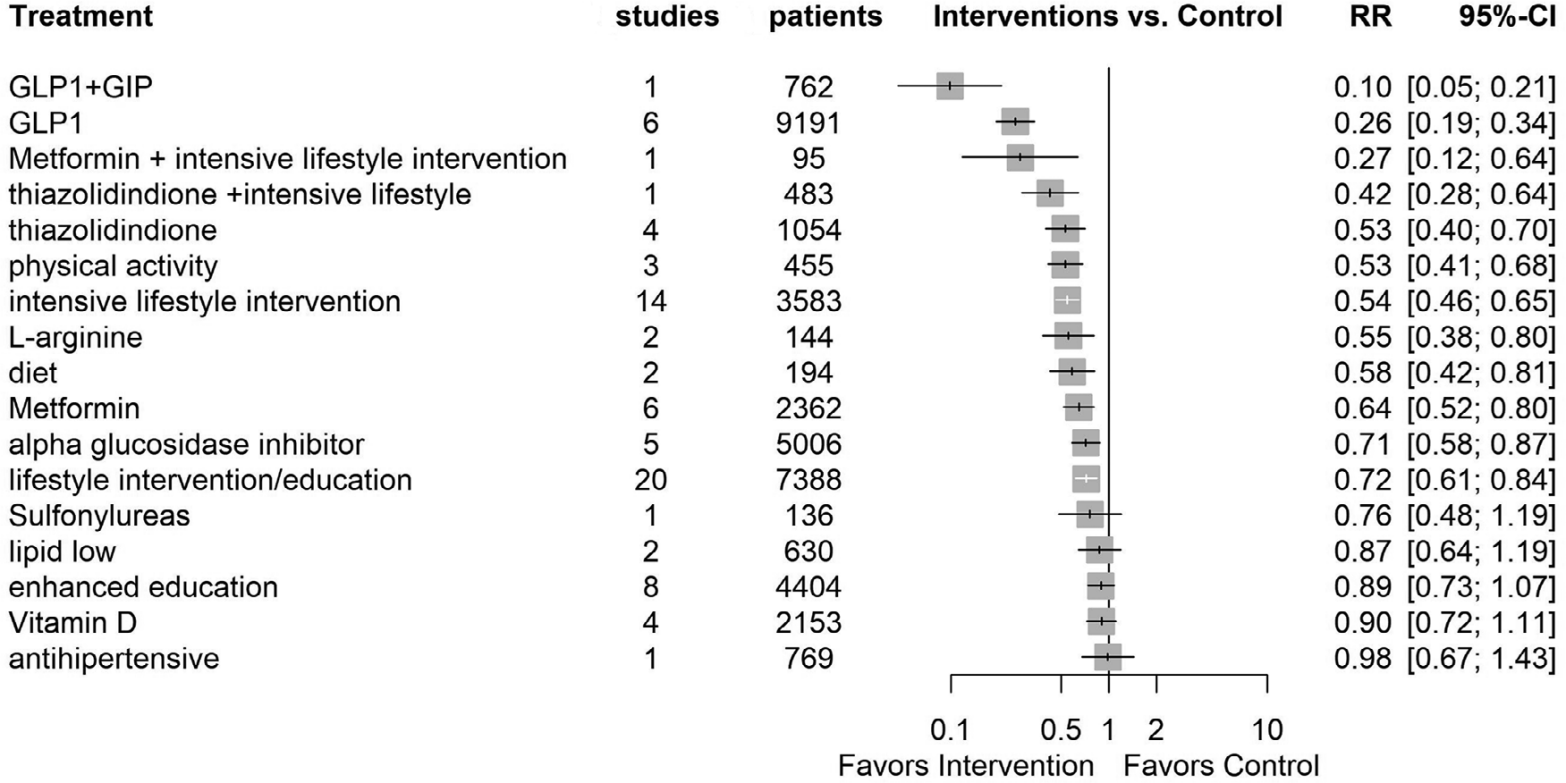
Forest plot of the effect of the evaluated interventions on the incidence of type 2 diabetes (T2D). Effect estimates are presented as risk ratios (RRs) with corresponding confidence intervals (CIs), comparing each intervention with the control group. The plot summarises the pooled effects across studies, highlighting the relative efficacy of the interventions in reducing T2D incidence.

Fifty‐nine papers reported the incidence of T2D (59 729 patients), and 18 interventions were analysed. The analysis comprises 153 possible pairwise comparisons and 29 direct comparisons (Table S2). When compared to control, dual GLP‐1 + GIP RA had the highest efficacy, reducing diabetes risk by 90% (relative risk [RR] = .10, CI [.05;  .21], LoE low) and it was closely followed by single GLP‐1 RAs: 74% (RR = .26, CI [.19;  .34], LoE moderate) and metformin plus ILI: 73% (RR = .27, CI [.12;  .64], LoE low). Antihypertensive agents had no significant effect on T2D risk reduction (RR = .98, CI [.67; 1.44]) (Figure 2). Heterogeneity varied from 0 in the case of direct comparisons for GLP‐1 RAs, metformin and vitamin D to 42% in the case of ILI, 64% for LI/education and physical activity and 67% in the case of AGI.

### 3.2 Ranking of the interventions for improving glycaemic control in prediabetic population: a network meta‐analysis

**FIGURE 3:**
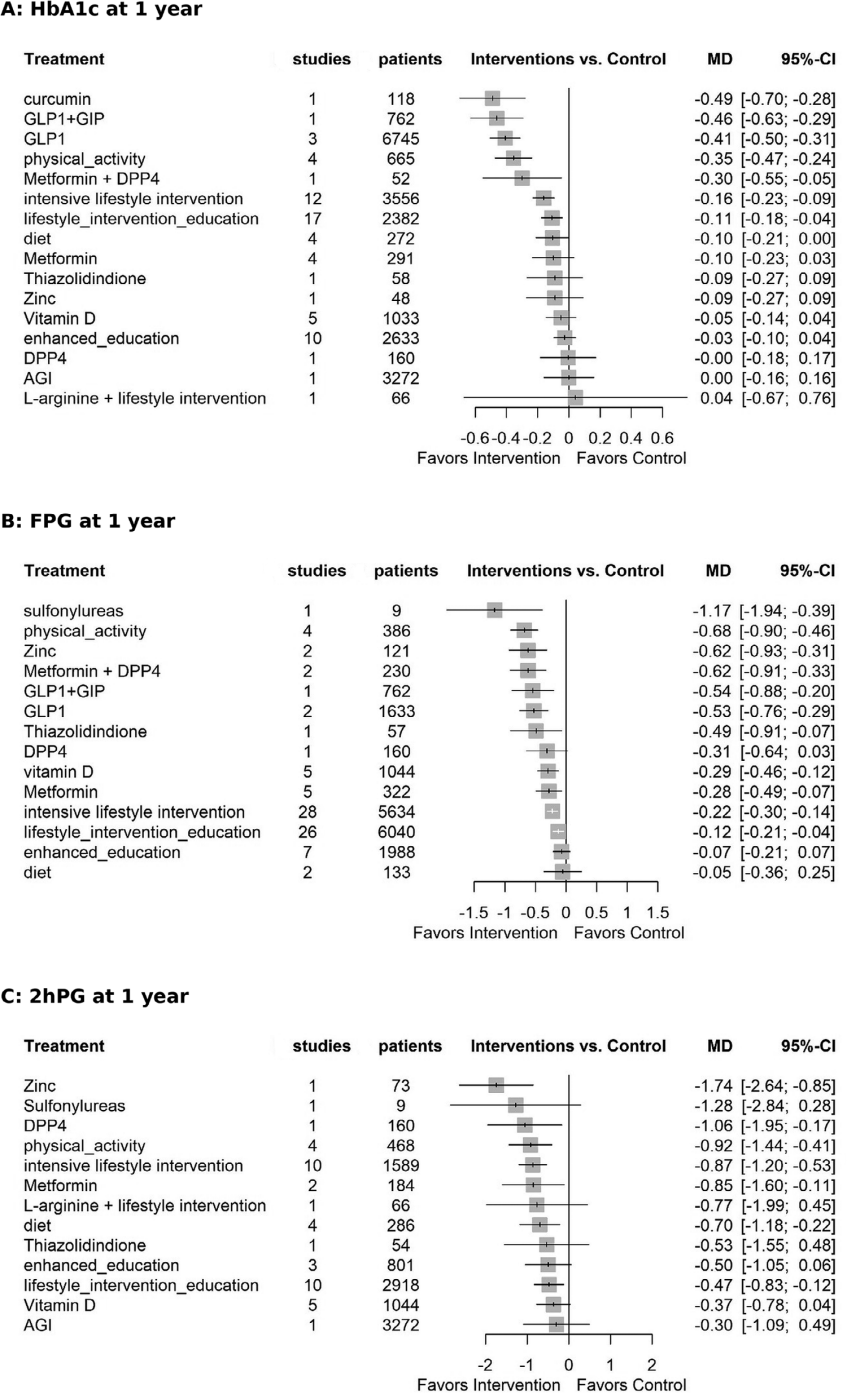
Changes from baseline in glycaemic outcomes at 12 months for each intervention compared with control. Panels show glycated haemoglobin (HbA1c; panel A), fasting plasma glucose (FPG; panel B) and 2‐h plasma glucose (2hPG; panel C). Mean differences (MDs) and 95% confidence intervals (CIs) are presented.

The ranking of interventions based on their efficacy in improving glycaemic control after 1 year is shown in Figure 3.

Changes in HbA1c at 1 year were reported in 48 studies on 37 548 participants evaluating 17 interventions. The network comprised 136 possible pairwise comparisons, of which 21 were informed by direct evidence. Among the investigated interventions, curcumin supplementation (MD = −.49, CI [−.70; −.28]), dual GLP‐1 + GIP RAs (MD = −.46, CI [−.63; −.29]), single GLP‐1 RAs (MD = −.41, CI [−.50; −.31]), physical activity (MD = −.35, CI [−.47; −.24]) and metformin + DPP‐4 (MD = −.30, CI [−.55; −.05]) were the most effective and the only ones to obtain a clinically significant reduction of HbA1c (≥.3%^23^). The treatment combination of L‐arginine with LI was the least effective (MD = .04, CI [−.67;  .76]) (Figure 3A). The individual results for the comparison between the direct and indirect estimates are available in Table S3. The subgroup analysis ranking the most effective interventions on decreasing HbA1c at 6 months revealed metformin + DPP‐4 (MD = −.50, CI [−.82; −.19]), followed by dual GLP‐1 + GIP RAs (MD = −.47, CI [−.71; −.23]), DPP‐4 (MD = −.38, CI [−.68; −.07]), single GLP‐1 RAs (MD = −.36, CI [−.59; −.12]), curcumin (MD = −.34, CI [−.61; −.07]) and metformin (MD = −.30, CI [−.47; −.14]), as the most effective and the only ones to obtain a clinically significant reduction. This was based on 43 studies involving 22 047 patients that assessed 17 interventions. Vitamin D supplementation ranked lowest (MD = .03, CI [−.13;  .19]) (Figure S5).

The ranking of the interventions based on their effect on FPG at 1‐year is summarised in Figure 3B (63 studies, 26 261 participants and 15 interventions). The network consisted of 105 possible pairwise comparisons, of which 21 were based on direct evidence. Compared to control, the most effective intervention were SUs (MD = −1.17, CI [−1.94; −.39]), and the least effective was diet (MD = −.05, CI [−.36;  .25]). Only SUs, physical activity, zinc and metformin + DPP‐4 induced a clinically significant reduction in FPG of at least  .55 mmol/L (10 mg/dL) that corresponds to a  .3% reduction in HbA1c.^24^ The individual results for comparing the direct and indirect estimates are available in Table S4. The 6‐month analysis (54 studies, 8489 participants and 15 interventions) revealed that the combination of metformin and DPP‐4, dual GIP + GLP‐1 RAs, lipid‐lowering agents and metformin monotherapy were most effective and the only interventions to achieve a clinically significant reduction in FPG at this timepoint. SUs ranked the lowest (Figure S9).

The change of 2hPG at 1‐year follow‐up was reported in 36 studies (17 47 participants and 14 interventions) (Figure 3C). The network comprised 91 possible pairwise comparisons, with 17 direct comparisons. Compared with control, zinc supplementation provided the greatest 2hPG reduction (MD = −1.74, CI [−2.64; −.85]) and AGIs the lowest (MD = −.30, CI [−1.09; −.49]) (Figure 3C). The individual results for the comparison between the direct and indirect estimates are available in Table S5. Notably, the clinically significant cut‐off for 2hPG is also at least  .55 mmol/L (10 mg/dL). At 6 months (24 studies, 2828 participants and 12 interventions), only zinc supplementation, antihypertensive drugs, physical activity and ILI induced both clinically and statistically significant reductions of 2hPG (Figure S13).

For all glucose metabolism markers, a sensitivity analysis was performed, including only interventions reported in at least three studies. The results of this analysis were consistent with those of the main analysis at both 6 months and 1 year, for all three outcomes (HbA1c, FPG and 2hPG) (Figures S4, S6, S8, S10, S12 and S14).

For continuous outcomes, heterogeneity was substantial across most analyses, with *I* ^2^ often greater than 90% (Figure S15).

**FIGURE 4:**
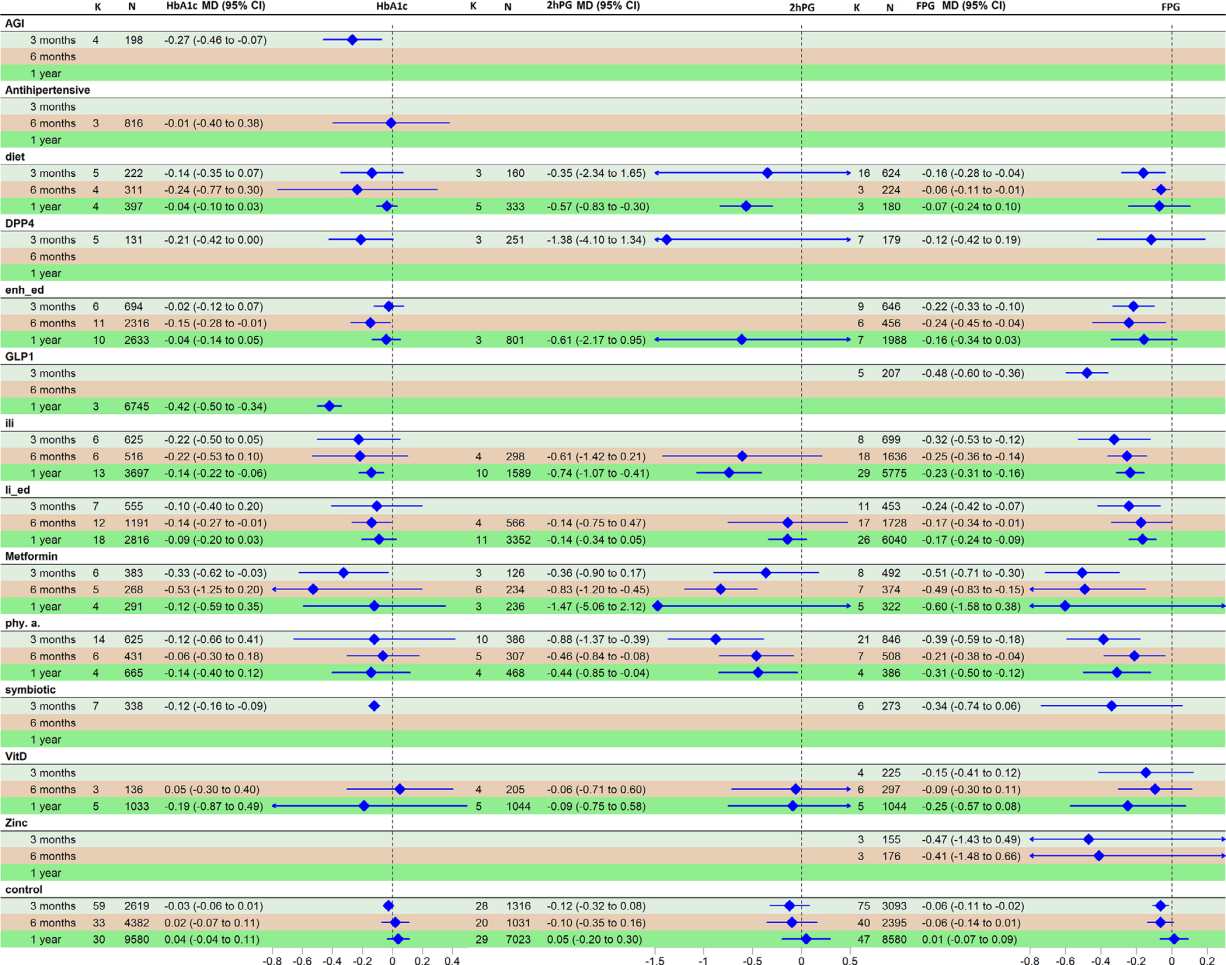
Changes from baseline in glycaemic outcomes within each intervention group at 3, 6 and 12 months. Each column presents a distinct glycaemic outcome—glycated haemoglobin (HbA1c), 2‐h plasma glucose (2hPG) and fasting plasma glucose (FPG), respectively—with results shown separately for each time point, illustrating the temporal evolution of treatment effects on glucose metabolism. Mean differences (MDs) and 95% confidence intervals (CIs) represent within‐group changes from baseline, not comparisons with control. *K*: number of studies; *N*: number of participants.

Figure 4 summarises the changes from baseline in the glycaemic control indices at 3, 6 and 12 months across treatment arms. The analysis demonstrates that most improvements in glycaemic profiles from various interventions occur within the first 3‒6 months, with metformin showing the most consistent and durable effects across the evaluated timepoints. Lifestyle‐based and GLP‐1‐based strategies also provide early benefits, although they tend to plateau. Heterogeneity and limited data restrict the interpretation of some interventions, especially supplements. Metformin, however, consistently reduced FPG from −.51 mmol/L at 3 months to −.60 mmol/L at 1 year, and 2hPG from −.36 to −1.47 mmol/L at 1 year. Notably, a similar pattern was observed in the control group, with initial improvements at 3 months followed by a gradual attenuation at later time points.

### 3.3 Efficacy of the interventions for improving cardiometabolic outcomes in prediabetic population: a network meta‐analysis

**FIGURE 5:**
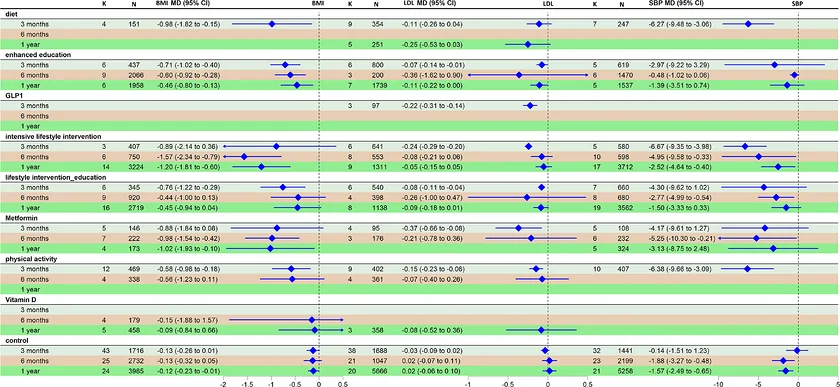
Changes from baseline in secondary cardiometabolic outcomes at 3, 6 and 12 months across the evaluated interventions. Each column corresponds to a specific outcome: body mass index (BMI), low‐density lipoprotein cholesterol (LDL‐C) and systolic blood pressure (SBP), respectively. For each outcome, pooled mean differences (MDs) and 95% confidence intervals (CIs) are shown for the different interventions at each time point, reflecting their broader impact on cardiometabolic risk factors over time. MDs and 95% CIs represent within‐group changes from baseline, not comparisons with control. *K*: number of studies; *N*: number of patients.

Figure 5 summarises the changes from baseline in the BMI, LDL and SBP at 3, 6 and 12 months across treatment arms. Among cardiometabolic outcomes, BMI reductions were most substantial with ILIs and metformin, and were sustained across all time points. LDL levels showed modest improvements, primarily with metformin, diet and GLP‐1. Notably, SBP improved significantly with physical activity interventions and diet, underscoring their role in reducing cardiovascular risk. Educational or supplement‐based strategies had a limited or inconsistent impact. The average baseline BMI of the population was approximately 29. At 1‐year follow‐up, the pooled MD for ILI was ‒1.20, corresponding to an estimated 4% reduction, almost approaching the threshold for clinical relevance (≥5%^25^). For metformin, the reduction at 1 year was approximately 3.5%, whereas other interventions did not yield clinically meaningful weight loss at any follow‐up point. For LDL, only metformin at 3 months achieved a clinically relevant reduction (≥10%^26^), with an approximate decrease of 11.4%. Lastly, only antihypertensives resulted in a clinically significant reduction in blood pressure, defined as a decrease in SBP of ≥10 mmHg^27^ at the 1‐year follow‐up.

### 3.4 Risk of bias and certainty of evidence

Overall, the risk of bias in the included studies was low to moderate. The risk of bias related to the randomisation process was generally rated as low to moderate. In most of the included studies, carers and individuals delivering the interventions were aware of participants' assigned groups, which may potentially introduce performance bias. For some interventions, such as ILI programs or physical activity, participants are often aware of the assigned intervention, which is unavoidable given the nature of the intervention. Additionally, most studies did not clearly report whether an intention‐to‐treat analysis was conducted, raising concerns about the appropriateness of the analytical approach used to estimate the effect of assignment to the intervention. Furthermore, in several studies, the pre‐specified analysis plan or protocol registration could not be identified, raising concerns ranging from minor to major regarding whether the data were analysed according to a predefined statistical plan finalised before the unblinded outcome data were available (Table S13).

According to the CINeMA assessment, the overall quality of evidence for the meta‐analyses was rated as low. However, for HbA1c and FPG, the evidence was stronger (moderate‒high), especially for the 1‐year analysis. Metformin, diet, ILI, LI and physical activity had the highest LoE (Tables S6–S12).

## 4 DISCUSSION

Our systematic review and network meta‐analysis on interventions to prevent diabetes progression among prediabetic patients revealed that incretin‐mimetic dual therapy with GIP and GLP‐1 RAs, as well as single GLP‐1 RAs, and metformin plus ILI are the most effective strategies. Furthermore, in the sensitivity analysis, GLP‐1 RAs, physical activity and metformin showed the strongest effect in reaching clinically relevant reductions in HbA1c, FPG and 2hPG at 1 year. For the improvement in cardiometabolic outcomes at 1 year, ILI had the strongest effect on BMI, while antihypertensives had the strongest effect on SBP.

When evaluating the reduction rate of T2D development, the differences between interventions were generally modest, with RRs largely overlapping across comparisons, excepting incretin‐mimetic dual therapy with GIP and GLP‐1RAs, which stood out from the other interventions by showing the largest estimated reduction in T2D risk compared with control. While ILI appears to have a beneficial effect in preventing T2D, its efficacy is more pronounced when combined with pharmacological therapies. Despite the ranking, no large differences were observed between interventions in reducing the glucose metabolism markers (FPG, 2hPG and HbA1c). Therefore, caution is warranted when interpreting the rankings, particularly in light of the wide CIs and limited direct evidence.

Our findings are broadly in line with previous network meta‐analyses. Sheng et al.^28^ evaluated both pharmacologic and lifestyle‐based strategies for preventing T2D in individuals with prediabetes and similarly identified GLP‐1 RAs among the most effective options. Their results also highlighted that treatment combinations—such as pairing TZDs with metformin or combining lifestyle interventions with TZDs—were more effective than individual treatments. Notably, their estimate for lifestyle interventions aligns closely with our findings for ILI. Likewise, their reported effectiveness of metformin alone is comparable to the results observed in our analysis. In a separate study, Amer et al.^29^ concluded that adding metformin to lifestyle modifications can lead to greater improvements in glycaemic control and a more pronounced reduction in diabetes risk compared to lifestyle changes alone. This synergistic effect likely stems from metformin's role in reducing hepatic glucose production while enhancing insulin sensitivity. Galaviz et al. concluded in their meta‐analysis that, while various pharmacological treatments can reverse prediabetes, lifestyle modification still offers the most robust evidence of effectiveness and should remain the primary recommended approach for managing this condition,^30^ a finding that aligns with our results. However, the meta‐analysis included only 47 papers. Additionally, a recent meta‐analysis by Wang et al.^31^ that explored lifestyle intervention assisted by technology (digital health intervention) reported a 12% reduction in progression to T2D (RR = .88, CI [.77; 1.01]) the same as with enhanced education in our analysis (RR = .89, CI [.73; 1.07]).

Notably, physical activity ranked higher than metformin or ILI in all analyses, highlighting the crucial effect of an active lifestyle relative to diet. Overall, from a pathophysiological standpoint, both insulin resistance and impaired β‐cell function are critical determinants of the development of glucose intolerance and the transition to overt diabetes. However, insulin resistance triggers a compensatory response, where the β‐cells increase insulin secretion to maintain glucose homeostasis, and this pathway ultimately results in reductions in β‐cell mass (a 40% reduction in β‐cell mass among prediabetes patients). The major contributor to insulin resistance is the pathological deposition of fat. While diet can control energy intake, exercise increases glucose uptake by the working muscle 7‒20 times above the basal rate by increasing glucose transporter‐4 translocation, with improvements in insulin sensitivity lasting up to 3 days.^32^ This can explain why physical activity is so effective, as it targets the primary cause of this condition.

It is known that the incidence of T2D rises in a gradual manner with a 2hPG concentration, even at levels below the threshold for IGT. Thus, a decrease in 2hPG should lead to a decreased risk of progression to T2D.^33^ In our analysis, this relationship was largely supported. Interventions such as physical activity, metformin and ILI reduced 2hPG more than FPG, and were also associated with substantial reductions in T2D incidence. These findings underscore the role of glucose lowering, particularly postprandial glucose, as a mediator in diabetes prevention. However, the LoE for the 2hPG analysis is mostly low and very low. Interestingly, SUs showed a relatively large reduction in 2hPG (−1.28 mmol/L; Figure 3C) but had a more modest effect on diabetes incidence (RR = .76; Figure 2), possibly due to their symptomatic mechanism of action, which does not modify the underlying pathophysiology (e.g., insulin resistance). Conversely, interventions such as vitamin D and AGIs had limited effects on both glycaemic values and diabetes incidence, with RRs for vitamin D similar to the ones reported by Barbarawi et al. (RR = .96, CI [.90; 1.03]).^34^ Interestingly, curcumin and zinc have been shown to be effective in reducing HbA1c and 2hPG, respectively; however, there is currently no data on their ability to reduce the progression to diabetes.

Overall, our results support the concept that interventions lowering 2‐h post‐load glucose also tend to reduce the risk of diabetes, aligning with the pathophysiological understanding of early dysglycaemia and its role in disease progression.

To date, no medications or alternative therapies have been approved by regulatory agencies specifically for preventing progression from prediabetes to T2D; however, GLP‐1 RAs appear to reduce the risk of diabetes progression. This was confirmed in a previous meta‐analysis by Sheng et al.^28^ Additionally, a recent pairwise meta‐analysis of 10 GLP‐1 RA trials conducted in broadly defined high‐risk populations^12^ reported a significant reduction in diabetes incidence (OR = .51, 95% CI  .28–.94). This finding was directionally consistent with our results, although our analysis yielded a larger effect estimate (RR = .26, 95% CI  .19–.34). However, the effect sizes are not directly comparable because of differences in effect measures, eligibility criteria, included studies and analytical frameworks.

### 4.1 Strengths and limitations

Several strengths of this meta‐analysis should be emphasised. First, the analysis included only RCTs, providing the highest LoE. Second, the large pooled sample size enhanced the statistical power and robustness of the results, surpassing those of any individual trial. Third, to our knowledge, our study is the first to perform a network meta‐analysis of glycaemic indices (HbA1c, FPG and 2hPG). Additionally, the time stratification is a true novelty, allowing for a more comprehensive understanding of the interventions’ effect. Furthermore, we extend the comparative framework by integrating and ranking newer therapeutic classes, particularly dual GLP‐1 + GIP RAs.

However, our findings should be interpreted in light of certain limitations. Notably, there was heterogeneity in study characteristics and effect sizes, although subgroup analyses helped explain part of this variability. Moreover, for several subgroup analyses, only single‐study comparisons were available for data analysis, resulting in limited direct evidence and yielding wide CIs, reducing the precision and certainty of the effect estimates. Despite their well‐established effects on weight, dual incretin‐based co‐agonists could not be included in the BMI analysis because weight outcomes were not reported separately for participants with prediabetes and normoglycaemia. Last, small differences between FPG, 2hPG and HbA1c data at 1 year may be explained by the inclusion of data derived from different studies and patient populations.

### 4.2 Implications for practice

Translating scientific knowledge for patients’ benefit is crucially important.^35^, ^36^ The time‐dependent trajectory of the interventions highlights the importance of early metabolic responsiveness, as well as the need for sustained patient adherence to the interventions and reinforcement strategies to maintain long‐term glycaemic benefits. The findings of this study further encourage healthcare professionals in preventive medicine to implement lifestyle‐based interventions in the management of prediabetes. However, as shown in the study, intensive treatment regimens (ILI, enhanced education) do not appear superior to basic lifestyle interventions, and the resources used for these types of interventions may not be justified. Overall, these results suggest that the initial phase of intervention is critical for achieving glycaemic benefit, highlighting the importance of early engagement and support.

To enhance adherence to lifestyle interventions, clinicians may actively identify and address practical obstacles such as limited time, financial constraints or poor access to healthy food and physical activity resources. Patients may be provided with free or low‐cost educational materials, such as nutritional guides, home exercise plans or vouchers for local gyms, which can significantly reduce dropout rates.

### 4.3 Implications for research

Future studies should further investigate the long‐term effectiveness and sustainability of combining metformin with ILI in individuals with prediabetes, particularly beyond the initial 3–6 months when the strongest effects are typically observed. Additionally, research is needed to explore strategies for maintaining patient adherence to lifestyle changes over time, including the role of digital tools, behavioural support and multidisciplinary care models. Furthermore, data on the 1‐year effect of the dual incretin‐mimetic therapy with GIP and GLP‐1 RAs on the glycaemic profile will clarify their role as a long‐term strategy among these patients.

## 5 CONCLUSION

Incretin‐based therapies, particularly dual GLP‐1 + GIP RAs and single GLP‐1 RAs followed by metformin plus ILI, are the most effective interventions for preventing the progression of prediabetes to T2D. However, for most interventions, a limited number of studies were available. Regarding improving glycaemic profiles, although lifestyle intervention strategies yield the best outcomes at 3–6 months, their effectiveness tends to decline over time, whereas the glycaemic benefits of metformin appear more persistent. In conclusion, lifestyle modifications remain central to T2D prevention, and can be successfully complemented by pharmacotherapy for long‐lasting results.

## AUTHOR CONTRIBUTIONS

*Conceptualisation, project administration, methodology, formal analysis and writing—original draft*: Bucur Maria. *Conceptualisation, formal analysis, visualisation and writing—review and editing*: Bunduc Stefania. *Conceptualisation, funding acquisition and writing—review and editing*: Hegyi Peter. *Conceptualisation, project administration, methodology, formal analysis, writing—review and editing, and supervision*: Rancz Anett. *Conceptualisation, formal analysis and visualisation*: Kói Tamás and Fazekas Karen. *Conceptualisation, methodology and writing—review and editing*: Obeidat Mahmoud. *Conceptualisation and writing—review and editing*: Papp Renáta, Ferdinándy Péter and Szentesi Andrea. *Conceptualisation, data curation and writing—review and editing*: Eperjesi Orsolya, Panait Robert Iulian, Topala Mihaela and Babakhani Avin Aphrodite. *Conceptualisation, supervision and writing—original draft*: Gheorghe Cristian.

All the authors certify that they have participated sufficiently in the work to take public responsibility for the content, including participation in the concept, design, analysis, writing or revision of the manuscript.

## CONFLICT OF INTEREST STATEMENT

The authors declare they have no conflicts of interest.

## ETHICS STATEMENT

No ethical approval was required for this systematic review with meta‐analysis, as all data were already published in peer‐reviewed journals. No patients were involved in the design, conduct or interpretation of our study.

## Supporting information



Supporting Information: ctm270829‐sup‐0001‐SuppMat.docx

## Data Availability

The datasets used in this study are available in the full‐text articles included in the systematic review and meta‐analysis and are also available from the corresponding author upon reasonable request.
