# Supplementary material for: Evidence‐based ranking of diabetes prevention in prediabetes: A comprehensive network meta‐analysis of 378 randomised controlled trials
Source: Clin Transl Med. 2026 Sep 8;16(9):e70829. doi: 10.1002/ctm2.70829 (PMC13554713; doi:10.1002/ctm2.70829)
Supplement: Supplementary file 1 [file CTM2-16-e70829-s001.docx]

**Evidence-Based Ranking of Diabetes Prevention in Prediabetes. A Comprehensive Network Meta-Analysis of 378 Randomised Controlled Trials**

**Bucur Maria^1,2^**, Eperjesi Orsolya^1,3,4^, Panait Robert Iulian ^1,2^, Topala Mihaela ^2^, Babakhani Avin Aphrodite^5,6^, Rancz Anett^1^, Kói Tamás ^1,7^, Fazekas Karen ^1^ Obeidat Mahmoud ^1^, Papp Renáta ^1,8,9^, Ferdinándy Péter ^8,9,10^, Szentesi Andrea ^1^, Gheorghe Cristian^2,11^, Hegyi Peter^1,4,12*^, Bunduc Stefania^1,2,11 *^

* The last two authors equally contributed

**Affiliations:**

1. Centre for Translational Medicine, Semmelweis University, Budapest 1085, Hungary;
2. “Carol Davila” University of Medicine and Pharmacy, Bucharest, Romania;
3. Department of Internal Medicine, Toldy Ferenc Hospital, Cegléd, Hungary
4. Institute of Pancreatic Diseases, Semmelweis University, Budapest, Hungary
5. Department of Neurology, Semmelweis University, Budapest, Hungary
6. János Szentágothai Neurosciences Division, Semmelweis University Doctoral School, Budapest, Hungary
7. Department of Stochastics, Institute of Mathematics, Budapest University of Technology and Economics, Budapest, Hungary
8. Department of Pharmacology and Pharmacotherapy, Semmelweis University, Budapest, Hungary;
9. Center for Pharmacology and Drug Research and Development, Semmelweis University, Budapest, Hungary;
10. Pharmahungary Group, Szeged, Hungary
11. Digestive Disease and Liver Transplant Center, Fundeni Clinical Institute, Romania;
12. Institute for Translational Medicine, Medical School, University of Pécs, Pécs, Hungary

Table of contents:

1. Supplementary Methods S1: PRISMA checklist
2. Supplementary Methods S2: PROSPERO registration and search key
3. Supplementary Methods S3: Grouping interventions
4. Table S1: Baseline characteristics table
5. Table S1A: Exclusion reasons
6. Figure S1: Network plot of possible treatments for reducing incidence of T2D
7. Figure S2: Direct comparisons of the treatments for reducing incidence of T2D
8. Table S2: Netslpit - incidence of T2D
9. Figure S3: Network plot of possible treatments for reducing HbA1c – time-point: 1 year
10. Table S3: Netslpit - HbA1c
11. Figure S4: Network plot of possible treatments for reducing HbA1c- sensitivity analysis – time-point: 1 year
12. Figure S5: Network plot of possible treatments for reducing HbA1c – time-point: 6 months
13. Figure S6: Network plot of possible treatments for reducing HbA1c – sensitivity analysis – time-point: 6 months
14. Figure S7: Network plot of possible treatments for reducing FPG – time-point: 1 year
15. Table S4: Netslpit - FPG
16. Figure S8: Network plot of possible treatments for reducing FPG - sensitivity analysis – time-point: 1 year
17. Figure S9: Network plot of possible treatments for reducing FPG – time-point: 6 months
18. Figure S10: Network plot of possible treatments for reducing FPG -sensitivity analysis – time-point: 6 months
19. Figure S11: Network plot of possible treatments for reducing 2hPG – time-point: 1 year
20. Table S5: Netslpit - 2hPG
21. Figure S12: : Network plot of possible treatments for reducing 2hPG – sensitivity analysis – time-point – 1 year
22. Figure S13: Network plot of possible treatments for reducing 2hPG – time-point: 6 months
23. Figure S14: Network plot of possible treatments for reducing 2hPG – sensitivity analysis – time-point: 6 months
24. Figure S15: Change from baseline in FPG with Metformin treatment
25. Table S6: League table
26. Table S7: Grading – incidence of T2D
27. Table S8: Grading – HbA1c – 1 year
28. Table S9: Grading – HbA1c – 6 months
29. Table S10: Grading – 2hPG – 1 year
30. Table S11: Grading – 2hPG – 6 months
31. Table S12: Grading – FPG – 1 year
32. Table S13: Grading – FPG – 6 months
33. Table S14: Risk of bias assessment (Rob)

# 1. PRISMA 2020 Main Checklist

| **Topic** | **No.** | **Item** | **Location where item is reported** |
| --- | --- | --- | --- |
| **TITLE** |  |  |  |
| **Title** | 1 | Identify the report as a systematic review. | page 1 |
| **ABSTRACT** |  |  |  |
| **Abstract** | 2 | See the PRISMA 2020 for Abstracts checklist |  |
| **INTRODUCTION** |  |  |  |
| **Rationale** | 3 | Describe the rationale for the review in the context of existing knowledge. | page 4, first paragraph |
| **Objectives** | 4 | Provide an explicit statement of the objective(s) or question(s) the review addresses. | page 4, 4th paragraph |
| **METHODS** |  |  |  |
| **Eligibility criteria** | 5 | Specify the inclusion and exclusion criteria for the review and how studies were grouped for the syntheses. | page 5, 2nd and 3rd paragraph |
| **Information sources** | 6 | Specify all databases, registers, websites, organisations, reference lists and other sources searched or consulted to identify studies. Specify the date when each source was last searched or consulted. | page 5, 4th paragraph |
| **Search strategy** | 7 | Present the full search strategies for all databases, registers and websites, including any filters and limits used. | supplementary material |
| **Selection process** | 8 | Specify the methods used to decide whether a study met the inclusion criteria of the review, including how many reviewers screened each record and each report retrieved, whether they worked independently, and if applicable, details of automation tools used in the process. | page 5, 6th paragraph |
| **Data collection process** | 9 | Specify the methods used to collect data from reports, including how many reviewers collected data from each report, whether they worked independently, any processes for obtaining or confirming data from study investigators, and if applicable, details of automation tools used in the process. | page 6, 2nd paragraph |
| **Data items** | 10a | List and define all outcomes for which data were sought. Specify whether all results that were compatible with each outcome domain in each study were sought (e.g. for all measures, time points, analyses), and if not, the methods used to decide which results to collect. | page 5, 3rd paragraph |
|  | 10b | List and define all other variables for which data were sought (e.g. participant and intervention characteristics, funding sources). Describe any assumptions made about any missing or unclear information. | page 6, 2nd paragraph |
| **Study risk of bias assessment** | 11 | Specify the methods used to assess risk of bias in the included studies, including details of the tool(s) used, how many reviewers assessed each study and whether they worked independently, and if applicable, details of automation tools used in the process. | page 6, 3rd paragraph |
| **Effect measures** | 12 | Specify for each outcome the effect measure(s) (e.g. risk ratio, mean difference) used in the synthesis or presentation of results. | page 6, 6th paragraph |
| **Synthesis methods** | 13a | Describe the processes used to decide which studies were eligible for each synthesis (e.g. tabulating the study intervention characteristics and comparing against the planned groups for each synthesis (item 5)). | page 6, 6th paragraph |
|  | 13b | Describe any methods required to prepare the data for presentation or synthesis, such as handling of missing summary statistics, or data conversions. | page 6, 6th paragraph |
|  | 13c | Describe any methods used to tabulate or visually display results of individual studies and syntheses. | page 6, 7tth paragraph |
|  | 13d | Describe any methods used to synthesize results and provide a rationale for the choice(s). If meta-analysis was performed, describe the model(s), method(s) to identify the presence and extent of statistical heterogeneity, and software package(s) used. | page 6, 4th paragraph |
|  | 13e | Describe any methods used to explore possible causes of heterogeneity among study results (e.g. subgroup analysis, meta-regression). | page 6, 7th paragraph |
|  | 13f | Describe any sensitivity analyses conducted to assess robustness of the synthesized results. | page 6, 3rd paragraph |
| **Reporting bias assessment** | 14 | Describe any methods used to assess risk of bias due to missing results in a synthesis (arising from reporting biases). | page 6, 3rd paragraph |
| **Certainty assessment** | 15 | Describe any methods used to assess certainty (or confidence) in the body of evidence for an outcome. | page 6, 3rd paragraph |
| **RESULTS** |  |  |  |
| **Study selection** | 16a | Describe the results of the search and selection process, from the number of records identified in the search to the number of studies included in the review, ideally using a flow diagram. | page 7, 4th paragraph |
|  | 16b | Cite studies that might appear to meet the inclusion criteria, but which were excluded, and explain why they were excluded. | n/a |
| **Study characteristics** | 17 | Cite each included study and present its characteristics. | supplementary material |
| **Risk of bias in studies** | 18 | Present assessments of risk of bias for each included study. | supplementary material |
| **Results of individual studies** | 19 | For all outcomes, present, for each study: (a) summary statistics for each group (where appropriate) and (b) an effect estimate and its precision (e.g. confidence/credible interval), ideally using structured tables or plots. | supplementary material |
| **Results of syntheses** | 20a | For each synthesis, briefly summarise the characteristics and risk of bias among contributing studies. | page 7, 4th paragraph |
|  | 20b | Present results of all statistical syntheses conducted. If meta-analysis was done, present for each the summary estimate and its precision (e.g. confidence/credible interval) and measures of statistical heterogeneity. If comparing groups, describe the direction of the effect. | pages 7,8,9,10,11,12,13 |
|  | 20c | Present results of all investigations of possible causes of heterogeneity among study results. | pages 7,8,9,10,11,12,13 |
|  | 20d | Present results of all sensitivity analyses conducted to assess the robustness of the synthesized results. | pages 7,8,9,10,11,12,13 |
| **Reporting biases** | 21 | Present assessments of risk of bias due to missing results (arising from reporting biases) for each synthesis assessed. | supplementary material |
| **Certainty of evidence** | 22 | Present assessments of certainty (or confidence) in the body of evidence for each outcome assessed. | page 8, 1st paragraph |
| **DISCUSSION** |  |  |  |
| **Discussion** | 23a | Provide a general interpretation of the results in the context of other evidence. | page 13, 2nd paragraph |
|  | 23b | Discuss any limitations of the evidence included in the review. | page 14, 5th paragraph |
|  | 23c | Discuss any limitations of the review processes used. | page 14, 5th paragraph |
|  | 23d | Discuss implications of the results for practice, policy, and future research. | page 14, 6th paragraph; page 15, 2nd paragraph |
| **OTHER INFORMATION** |  |  |  |
| **Registration and protocol** | 24a | Provide registration information for the review, including register name and registration number, or state that the review was not registered. | page 5, 1st paragraph |
|  | 24b | Indicate where the review protocol can be accessed, or state that a protocol was not prepared. | supplementary material |
|  | 24c | Describe and explain any amendments to information provided at registration or in the protocol. | page 7, 3rd paragraph |
| **Support** | 25 | Describe sources of financial or non-financial support for the review, and the role of the funders or sponsors in the review. | page 2, 4th paragraph |
| **Competing interests** | 26 | Declare any competing interests of review authors. | page 2, 1st paragraph |
| **Availability of data, code and other materials** | 27 | Report which of the following are publicly available and where they can be found: template data collection forms; data extracted from included studies; data used for all analyses; analytic code; any other materials used in the review. | n/a |

# PRIMSA Abstract Checklist

| **Topic** | **No.** | **Item** | **Reported?** |
| --- | --- | --- | --- |
| **TITLE** |  |  |  |
| **Title** | 1 | Identify the report as a systematic review. | Yes |
| **BACKGROUND** |  |  |  |
| **Objectives** | 2 | Provide an explicit statement of the main objective(s) or question(s) the review addresses. | Yes |
| **METHODS** |  |  |  |
| **Eligibility criteria** | 3 | Specify the inclusion and exclusion criteria for the review. | No |
| **Information sources** | 4 | Specify the information sources (e.g. databases, registers) used to identify studies and the date when each was last searched. | Yes |
| **Risk of bias** | 5 | Specify the methods used to assess risk of bias in the included studies. | No |
| **Synthesis of results** | 6 | Specify the methods used to present and synthesize results. | Yes |
| **RESULTS** |  |  |  |
| **Included studies** | 7 | Give the total number of included studies and participants and summarise relevant characteristics of studies. | Yes |
| **Synthesis of results** | 8 | Present results for main outcomes, preferably indicating the number of included studies and participants for each. If meta-analysis was done, report the summary estimate and confidence/credible interval. If comparing groups, indicate the direction of the effect (i.e. which group is favoured). | Yes |
| **DISCUSSION** |  |  |  |
| **Limitations of evidence** | 9 | Provide a brief summary of the limitations of the evidence included in the review (e.g. study risk of bias, inconsistency and imprecision). | No |
| **Interpretation** | 10 | Provide a general interpretation of the results and important implications. | Yes |
| **OTHER** |  |  |  |
| **Funding** | 11 | Specify the primary source of funding for the review. | Yes |
| **Registration** | 12 | Provide the register name and registration number. | Yes |

*From:* Page MJ, McKenzie JE, Bossuyt PM, Boutron I, Hoffmann TC, Mulrow CD, et al. The PRISMA 2020 statement: an updated guideline for reporting systematic reviews. MetaArXiv. 2020, September 14. DOI: 10.31222/osf.io/v7gm2. For more information, visit: <www.prisma-statement.org>

2. Supplementary Methods S1: PROSPERO registration and search key

We previously submitted our study protocol to the **International prospective register of systematic reviews (PROSPERO) (CRD42023473875).**

The study protocol can be accessed via the following link:

<https://www.crd.york.ac.uk/PROSPERO/view/CRD42023473875>

The search key used consists of 4 domains: the first one dedicated to prediabetes, second to T2D, third to terms related to prevention and the last to RCTs.

A. Search key PubMed and Cochrane Central Register of Controlled Trials (Central):

(prediab* OR “pre diabetes” OR “pre diabetic” OR “pre-diabetes” OR “pre-diabetic” OR “glucose intolerance” OR (“elevated” ”glucose”) OR (impair* “glucose”) OR (“abnormal” “glucose”) OR “potential diabetes” OR “potential type 2 diabetes” OR dysglycem* OR dysglicem*) AND (T2D OR T2DM OR diabet*) AND (“onset” OR “progression” OR “development” OR “diagnosis” OR “risk” OR “incidence” OR prevent* OR “remission” OR “revert” OR revers* OR “regression” OR “cure” OR treat* OR “reduction” OR improv* OR “delay”) AND random*

MEDLINE (via PubMed): 7284

Central: 7519

B. Search key Embase:

Advanced search key – options unchecked

(prediab* OR 'pre diabetes' OR 'pre diabetic' OR 'pre-diabetes' OR 'pre-diabetic' OR 'glucose intolerance' OR (elevated glucose) OR (impair* glucose) OR (abnormal glucose) OR 'potential diabetes' OR 'potential type 2 diabetes' OR dysglycem* OR dysglicem*) AND (t2d OR t2dm OR diabet*) AND (onset OR progression OR development OR diagnosis OR risk OR incidence OR prevent* OR remission OR revert OR revers* OR regression OR cure OR treat* OR reduction OR improv* OR delay) AND random*

Embase: 13303

3. Supplementary Methods S2: Grouping interventions

ILI was defined as any structured combination of physical activity and dietary change delivered comprehensively and intensively (e.g., with the use of trainers, gym memberships, or pedometers), whereas the LI group included interventions offering general lifestyle advice without such resources.

For pharmacological treatments, the highest reported dose was included in the final analysis when multiple doses were tested. The "enhanced education" group included studies that integrated digital health tools (e.g., mobile applications, SMS reminders) to deliver lifestyle-related content and support behavioral adherence. The physical activity category comprised diverse forms of movement, including aerobic and resistance training, yoga, and Nordic walking. The diet group included any intervention prescribing a specific dietary pattern (e.g., low-carbohydrate, high-protein, high-fiber, or Mediterranean diets); studies focusing on potentially harmful dietary patterns (e.g., high-refined carbohydrate intake) were excluded. Combined interventions were reported as such, except for the addition of basic lifestyle recommendations to both intervention groups and control, in which case the primary intervention was reported. The ILI combined with pharmacotherapy was reported as a combination.

Vitamin D and zinc, though typically included under supplements, were evaluated separately due to the ongoing debate surrounding their effects in the literature. Also, curcumin and L-arginine were reported separately for their prominent effect in the analysis. Antihypertensive and lipid-lowering drugs were treated as distinct categories, though they often appeared in combination with other interventions.

4. Table S1A: exclusion reasons

| Author | *Title* | Year of publication | Reason for exclusion |
| --- | --- | --- | --- |
| NA | *From the 9th ICO: Xendos study: Orlistat plus diet prevents, delays diabetes onset in obese patients.* | 2002 | study design |
| NA | *Acarbose delays progression to diabetes.* | 2002 | study design |
| NA | *Diabetes prevention: still no effective drugs.* | 2007 | study design |
| NA | *Rosiglitazone may prevent type 2 diabetes in high-risk patients.* | 2007 | study design |
| NA | *Rosiglitazone, but not ramipril, prevents onset of type 2 diabetes in high-risk patient* | 2007 | study design |
| NA | *A 6-year lifestyle intervention can prevent or delay diabetes for decades.* | 2008 | study design |
| NA | *Pioglitazone reduces the risk of converting impaired glucose tolerance to type 2 diabetes.* | 2011 | study design |
| NA | *Restoring Insulin Secretion (RISE): design of studies of β-cell preservation in prediabetes and early type 2 diabetes across the life span.* | 2014 | study design |
| NA | *Predictors of nonsevere and severe hypoglycemia during glucose-lowering treatment with insulin glargine or standard drugs in the ORIGIN trial.* | 2015 | study design |
| NA | *Metformin for prediabetes.* Medical Letter on Drugs and Therapeutics | 2016 | study design |
| NA | *Erratum: 3 years of liraglutide versus placebo for type 2 diabetes risk reduction and weight management in individuals with prediabetes: a randomised, double-blind trial* | 2017 | study design |
| NA | *Correction to Effects of acarbose on cardiovascular and diabetes outcomes in patients with coronary heart disease and impaired glucose tolerance (ACE): a randomised, double-blind, placebo-controlled trial* | 2019 | study design |
| NA | *Erratum: Effects of a diabetes prevention program on type 2 diabetes risk factors and quality of life among Latino youths with prediabetes: a randomized clinical trial* | 2022 | study design |
| NA | *Correction to Safety and effectiveness of metformin plus lifestyle intervention compared with lifestyle intervention alone in preventing progression to diabetes in a Chinese population with impaired glucose regulation: a multicentre, open-label, randomised controlled trial* | 2023 | study design |
| Abbaspoor Z. | *The effect of education through mobile phone short message service on promoting self-care in pre-diabetic pregnant women: a randomized controlled trial.* | 2020 | population |
| Aberle J. | *Effects of treatment with liraglutide 3.0 mg in subjects with BMI < 35 and BMI ≥35 kg/m2: Subgroup analysis of the SCALE Obesity and Prediabetes 56-week trial*. | 2016 | study design |
| Ackermann R. | *Long-term effects of a community-based lifestyle intervention to prevent type 2 diabetes: The DEPLOY extension pilot study.* | 2011 | population |
| Ackermann R. | *Rationale, design, and baseline characteristics of a community-based comparative effectiveness trial to prevent type 2 diabetes in economically disadvantaged adults: the RAPID Study.* | 2014 | study design |
| Ackermann R. | *A randomized comparative effectiveness trial of a primary care-community linkage for preventing type 2 diabetes.* | 2015 | study design |
| Agarwal S. | *Metformin in schizophrenia spectrum disorders and early co-morbid prediabetes/diabetes: A double-blind randomized control trial.* | 2019 | study design |
| Agarwal S. | *Metformin for early co-morbid prediabetes or diabetes in schizophrenia spectrum disorders: a double blind randomized pilot study.* | 2020 | study design |
| Agarwal S. | *Metformin for early comorbid glucose dysregulation and schizophrenia spectrum disorders: a pilot double-blind randomized clinical trial.* | 2021 | population |
| Al-Hamdan R. | *Efficacy of different prediabetes program models in improving clinical outcomes in people with prediabetes.* | 2020 | study design |
| Alaei Shahmiri F. | *High-dose thiamine supplementation improves glucose tolerance in hyperglycemic individuals: a randomized, double-blind cross-over trial.* | 2013 | population |
| Alaei Shahmiri F. | *High-dose thiamine supplementation may reduce resting energy expenditure in individuals with hyperglycemia: a randomized, double - blind cross-over trial.* | 2020 | population |
| Alaei Shahmiri F. | *High-dose thiamine supplementation may reduce resting energy expenditure in individuals with hyperglycemia: a randomized, double – blind cross-over trial.* | 2020 | duplicat |
| Alaei Shahmiri F. | *The impact of thiamine supplementation on blood pressure, serum lipids and C-reactive protein in individuals with hyperglycemia: a randomised, double-blind cross-over trial.* | 2015 | population |
| Alfawaz HA. | *Significant reduction in full metabolic syndrome in saudi subjects with elevated fasting glucose through an intensive lifestyle monitoring programme of 12 months.* | 2018 | study design |
| Ali A. | *Chromium effects on glucose tolerance and insulin sensitivity in persons at risk for diabetes mellitus.* | 2011 | population |
| Ali A. | *Chromium effects on glucose tolerance, insulin sensitivity and vascular function in people at risk for diabetes.* | 2010 | study design |
| Ali A. | *Chromium effects on glucose tolerance and insulin sensitivity in persons at risk for diabetes mellitus.* | 2011 | duplicat |
| Alkasem M. | *Effects of grape skin extract (GSE) on glycemic response to a starch challenge in prediabetic subjects.* | 2019 | study design |
| Allen P. | *Dietary, physical activity and metabolic changes among young urban Native American women with prediabetes participating in a primary prevention program* | 2006 | study design |
| Allen P. | *Impact of periodic follow-up testing among urban American Indian women with impaired fasting glucose.* | 2008 | outcomes of interest not reported |
| Amano Y. | *Glycemic index-based nutritional education improves blood glucose control in Japanese adults: a randomized controlled trial.* | 2007 | population |
| Amini P. | *Comment on 'Exercise training decreases pancreatic fat content and improves beta cell function regardless of baseline glucose tolerance: a randomised controlled trial'.* | 2019 | study design |
| An Y. | *Differences in efficacy of diet and/or exercise interventions in preventing diabetes in normal and overweight/obese people with IGT: results from the 20-year follow-up of daqing diabetes prevention study.* | 2017 | study design |
| Andreeva A. | *Cholecalciferol therapy effect on glucose metabolism in patients with prediabetes. Diabetologia,* | 2021 | study design |
| Andrulionyte L. | *Single nucleotide polymorphisms of the peroxisome proliferator-activated receptor-alpha gene (PPARA) influence the conversion from impaired glucose tolerance to type 2 diabetes: the STOP-NIDDM trial.* | 2007 | outcomes of interest not reported |
| Andrulionyte L. | *Single nucleotide polymorphisms of the Peroxisome Proliferator-Activated Receptor-α gene (PPARA) influence the conversion from impaired glucose tolerance to type 2 diabetes: The STOP-NIDDM trial. Diabetes,* | 2007 | duplicat |
| Anjana R. | *Reducing the risk of development of diabetes: Do we have an answer*? | 2012 | study design |
| Antonucci T. | *Impaired glucose tolerance is normalized by treatment with the thiazolidinedione troglitazone.* | 1997 | intervention |
| Aoki K. | *Effects of miglitol, sitagliptin or their combination on plasma glucose, insulin and incretin levels in non-diabetic men.* | 2010 | population |
| Asai A. | *Effect of mulberry leaf extract with enriched 1-deoxynojirimycin content on postprandial glycemic control in subjects with impaired glucose metabolism* | 2011 | intervention |
| Astrup A. | *Efficacy and safety of liraglutide 3.0 Mg in adult overweight and obese weight loss responders without diabetes: Results of the 56-week randomised, controlled, scale obesity and prediabetes trial.* | 2015 | study design |
| Astrup A. | *Weight loss and associated improvements in cardiometabolic risk factors with liraglutide 3.0 mg in the SCALE Obesity and Prediabetes randomised, double-blind, placebo-controlled 3-year trial. Obesity Facts,* | 2016 | study design |
| Astrup A. | *Weight loss and improvements in cardiometabolic risk factors with liraglutide 3.0 mg: The SCALE obesity and prediabetes randomized, double-blind, placebo-controlled 3-year trial.* | 2016 | study design |
| Astrup A. | *Pretreatment fasting plasma glucose and insulin as determinants of weight loss success: the new nordic diet study.* | 2017 | study design |
| Avenell A. | *Vitamin D supplementation and type 2 diabetes: a substudy of a randomised placebo-controlled trial in older people (RECORD trial, ISRCTN 51647438).* | 2009 | population |
| Ayame H. | *[The Da Qing IGT and Diabetes study].* | 2005 | study design |
| Azar K. | *Effectiveness of lifestyle intervention by obesity status: Does one size fit all?* | 2013 | study design |
| Azar K. | *Baseline obesity status modifies effectiveness of adapted diabetes prevention program lifestyle interventions for weight management in primary care.* | 2013 | population |
| Baker C. | *Circulating inflammatory biomarkers are not improved with combined low-volume high-intensity interval training and progressive resistance training in prediabetes.* | 2021 | study design |
| Baker L. | *Effects of aerobic exercise on cognition and biomarkers in adults with a double-hit risk for dementia: Mild cognitive impairment and prediabetes.* | 2014 | study design |
| Balbi Seixas M. | *Exercise and lifestyle education program for people living with diabetes and prediabetes in Brazil: a pilot randomized trial.* | 2022 | population |
| Bales C. | *Effects of a 6-month trial of weight-loss diet plus aerobic exercise versus matched (by dose/intensity) exercise alone on glucose tolerance and insulin resistance in subjects with pre-diabetes.* | 2014 | study design |
| Ballantyne C. | *Niacin extended-release and simvastatin in patients with impaired fasting glucose. Diabetes,* | 2009 | study design |
| Ballon J. | *Metformin and impaired glucose tolerance in overweight persons with schizophrenia.* | 2011 | study design |
| Banzer W. | *Improvement of prediabetic condition after 4 and 12 weeks intervention by the ACOORH concept-subanalysis of a multicenter RCT.* | 2018 | study design |
| Barengolts E. | *High dose vitamin d supplementation for 12 months improves insulin sensitivity and glucose disposition indices in African American men with hypovitaminosis d and dysglycemia: Results of the D-vitamin intervention in veteran administration (DIVA) randomized clinical trial (RCT).* | 2014 | study design |
| Barengolts E. | *In african american men with dygsycemia and vitamin d deficiency, vitamin d supplementation for 1 year may improve insulin sensitivity and glycemic status. DATA from a randomized controlled trial of vitamin D intervention at veteran administration (DIVA, NCT01375660).* | 2014 | study design |
| Barengolts E. | *EFFECT OF HIGH-DOSE VITAMIN D REPLETION ON GLYCEMIC CONTROL IN AFRICAN-AMERICAN MALES WITH PREDIABETES AND HYPOVITAMINOSIS D.* | 2015 | population |
| Batten R. | *A 12-Month Follow-Up of the Effects of a Digital Diabetes Prevention Program (VP Transform for Prediabetes) on Weight and Physical Activity Among Adults With Prediabetes: Secondary Analysis. JMIR Diabetes,* | 2022 | study design |
| Beck E. | *Effects of short and long term exercise training on glucose tolerance, lipid metabolism and endothelial function in overweight, pre-diabetic coronary artery disease patients.* | 2011 | study design |
| Beck E. | *Impact of exercise training on waist circumference, glucose metabolism and endothelial function in pre-diabetic, adipose patients with severe coronary heart disease.* | 2012 | study design |
| Ben-Yacov et al. | *Personalized nutrition for prediabetes by prediction of glycemic responses.* | 2020 | study design |
| Bennett S. | *Rosiglitazone improves insulin sensitivity, glucose tolerance and ambulatory blood pressure in subjects with impaired glucose tolerance.* | 2004 | intervention |
| Berkowitz K. | *Effect of troglitazone on insulin sensitivity and pancreatic beta-cell function in women at high risk for NIDDM.,* | 1996 | intervention |
| Berkowitz K. | *Effect of troglitazone on insulin sensitivity and pancreatic β-cell function in women at high risk for niddm.* | 1996 | duplicat |
| Bessell E. | *Changes in Prediabetes Status Among Adults During a 6-Month Randomized Placebo-controlled Supplement Trial With Nutrition and Lifestyle Counselling and 6-Month Follow-up.* | 2023 | population |
| Bessell E. | *Changes in prediabetes status among adults during a six-month randomised placebo-controlled supplement trial with nutrition and lifestyle counselling and six-month follow-up.* | 2023 | duplicat |
| Best C. | *Sitagliptin reduces inflammation and chronic immune cell activation in HIV+ adults with impaired glucose tolerance.* | 2015 | population |
| Bharmal S. | *Acute Nutritional Ketosis and Its Implications for Plasma Glucose and Glucoregulatory Peptides in Adults with Prediabetes: A Crossover Placebo-Controlled Randomized Trial.* | 2021 | outcomes of interest not reported |
| Bhatt S. | *Author Correction: Vitamin D Supplementation in Overweight/obese Asian Indian Women with Prediabetes Reduces Glycemic Measures and Truncal Subcutaneous Fat: A 78 Weeks Randomized Placebo-Controlled Trial (PREVENT-WIN Trial). Sci Rep,* | 2020 | study design |
| Bhatt S. | *Erratum: Author Correction: Vitamin D Supplementation in Overweight/obese Asian Indian Women with Prediabetes Reduces Glycemic Measures and Truncal Subcutaneous Fat: A 78 Weeks Randomized Placebo-Controlled Trial (PREVENT-WIN Trial)* | 2020 | study design |
| Biryukova E. | ***Metabolic and cardiovascular effects of early insulin glargin prescription: Based on data from ORIGIN study.*** | 2012 | study design |
| Blackman A. | *Effect of liraglutide 3.0 mg in individuals with obesity and moderate or severe obstructive sleep apnea: the SCALE Sleep Apnea randomized clinical trial.* | 2016 | population |
| Blackman A. | *Effect of liraglutide 3.0 mg in individuals with obesity and moderate or severe obstructive sleep apnea: The scale sleep apnea randomized clinical trial.* | 2016 | duplicat |
| Block G. | *A Fully Automated Diabetes Prevention Program, Alive-PD: Program Design and Randomized Controlled Trial Protocol.* | 2015 | study design |
| Block G. | *Fully-automated online diabetes prevention program, Alive-PD, is effective in lowering HbA1c, glucose, and weight. Diabetes,* | 2015 | study design |
| Block G. | *Improving diet, activity and wellness in adults at risk of diabetes: Randomized controlled trial.* | 2016 | outcomes of interest not reported |
| Blüher M. | *3-year efficacy and safety for liraglutide 3.0 mg in adults with obesity/overweight, prediabetes and baseline BMI < 35 vs. ≥35 kg/m2 in the SCALE Obesity and Prediabetes, double-blind, placebo-controlled trial.* | 2017 | study design |
| Böhmer K. | *Linear loss of insulin secretory capacity during the last six months preceding IDDM. No effect of antiedematous therapy with ketotifen.* | 1994 | population |
| Bone H. | *Effects of pioglitazone on bone in postmenopausal women with impaired fasting glucose or impaired glucose tolerance: a randomized, double-blind, placebo-controlled study.* | 2013 | duplicat |
| Bouchard D. | *Impact of health professionals' contact frequency on response to a lifestyle intervention with individuals at high risk for diabetes.* | 2012 | outcomes of interest not reported |
| Bouchard D. | *Lifestyle intervention in pre-diabetic adults and physical function.* | 2010 | study design |
| Boucher B. | *Does high-dose vitamin D supplementation impact insulin resistance and risk of development of diabetes in patients with pre-diabetes? A double-blind randomized controlled trial.* | 2019 | study design |
| Boucher B. | *Does high-dose vitamin D supplementation impact insulin resistance and risk of development of diabetes in patients with pre-diabetes? A double-blind randomized controlled trial.* | 2019 | study design |
| Bowen J. | *Effects of almond consumption on metabolic function and liver fat in overweight and obese adults with elevated fasting blood glucose: A randomised controlled trial.* | 2019 | population |
| Boyko E. | *Effects of ethnicity on diabetes incidence and prevention: results of the Diabetes REduction Assessment with ramipril and rosiglitazone Medication (DREAM) trial.* | 2010 | intervention |
| Brinkmann C. | *Lifestyle intervention reduces risk score for cardiovascular mortality in company employees with pre-diabetes or diabetes mellitus - A secondary analysis of the PreFord randomized controlled trial with 3 years of follow-up.* | 2023 | outcomes of interest not reported |
| Brinkmann C. | *Lifestyle intervention reduces risk score for cardiovascular mortality in company employees with pre-diabetes or diabetes mellitus – A secondary analysis of the PreFord randomized controlled trial with 3 years of follow-up.* | 2023 | duplicat |
| Buchanan T. | *Response of pancreatic beta-cells to improved insulin sensitivity in women at high risk for type 2 diabetes.* | 2000 | intervention |
| Buchanan T. | *Response of pancreatic β-cells to improved insulin sensitivity in women at high risk for type 2 diabetes.* | 2000 | duplicat |
| Bulatova N. | *Effect of metformin combined with lifestyle modification versus lifestyle modification alone on proinflammatory-oxidative status in drug-naïve pre-diabetic and diabetic patients: A randomized controlled study.* | 2018 | population |
| Cabre J. | *Ten-year follow-up of diabetes incidence in the DE-PLAN project (diabetes prevention using intensive lifestyle intervention) in Catalonia.* | 2019 | study design |
| Califf R. | *Prevention of diabetes and cardiovascular disease in patients with impaired glucose tolerance: rationale and design of the Nateglinide And Valsartan in Impaired Glucose Tolerance Outcomes Research (NAVIGATOR) Trial.* | 2008 | study design |
| Camp A. | *Successful implementation of a T2D prevention program in a community health center setting.* | 2013 | study design |
| Campos C. | *Effects of a community based collaborative diabetes control and prevention program for low-income overweight and obese patients in a suburban population-a pilot study.* | 2019 | study design |
| Cariou B. | *Effects of the new dual PPAR α/δ agonist GFT505 on lipid and glucose homeostasis in abdominally obese patients with combined dyslipidemia or impaired glucose metabolism.* | 2011 | intervention |
| Cariou B. | *Effects of the new dual PPARalpha/ agonist GFT505 on lipid and glucose homeostasis in abdominally obese patients with combined dyslipidemia or impaired glucose metabolism.* | 2011 | duplicat |
| Cavaghan M. | *Treatment with the oral antidiabetic agent troglitazone improves beta cell responses to glucose in subjects with impaired glucose tolerance.* | 1997 | intervention |
| Cavaghan M. | *Treatment with the oral antidiabetic agent troglitazone improves β cell responses to glucose in subjects with impaired glucose tolerance.* | 1997 | duplicat |
| Cha E. | *A feasibility study to develop a diabetes prevention program for young adults with prediabetes by using digital platforms and a handheld device.* | 2014 | study design |
| Chandrakala G. | *Long-term effects of a reduced fat diet intervention in pre-diabetes.* | 2010 | study design |
| Chatterjee R. | *A pilot study of potassium supplements on glucose metabolism in African-American adults with prediabetes.* | 2017 | study design |
| Chatterjee R. | *Effects of changes in potassium with valsartan use on diabetes risk: Nateglinide and Valsartan in Impaired Glucose Tolerance Outcomes Research (NAVIGATOR) trial.* | 2013 | outcomes of interest not reported |
| Chatterjee R. | *Changes in potassium do not mediate the protective effect of valsartan on Diabetes risk: The nateglinide and valsartan in impaired glucose tolerance outcomes research (navigator) trial.* | 2012 | study design |
| Chavez A. | *Early Pharmacological Intervention (EPIC) to Preserve BetaCell Function in Prediabetes (PreDM). ,* | 2023 | study design |
| Chavez Velazquez A. | *Effect of PharmacologIC (EPIC) Therapy on Insulin Sensitivity and Beta-Cell Function in Prediabetes (PreDM).* | 2022 | study design |
| Chazova I. | *Moxonidine improves glycaemic control in mildly hypertensive, overweight patients: a comparison with metformin.* | 2006 | population |
| Cheng S. | *Effects of aerobic exercise and diet intervention on glycaemic control and liver fat content in men and women aged 50-65 years with prediabetes and non-alcoholic fatty liver disease: a multicentre, randomised controlled trial.* | 2016 | study design |
| Cheon J. | *Additional benefits of pioglitazone to lifestyle modification in patients at increased risk for diabetes and newly developed diabetes.* | 2014 | study design |
| Chiasson J. | *Acarbose for the prevention of diabetes, hypertension, and cardiovascular disease in subjects with impaired glucose tolerance: the Study to Prevent Non-Insulin-Dependent Diabetes Mellitus (STOP-NIDDM) Trial.* | 2006 | overlapping population |
| Chiasson J. | *The STOP-NIDDM Trial: an international study on the efficacy of an alpha-glucosidase inhibitor to prevent type 2 diabetes in a population with impaired glucose tolerance: rationale, design, and preliminary screening data. Study to Prevent Non-Insulin-Dependent Diabetes Mellitus.* | 1998 | study design |
| Chiasson J. | *The STOP-NIDDM trial: An international study on the efficacy of an α- glucosidase inhibitor to prevent type 2 diabetes in a population with impaired glucose tolerance: Rationale, design, and preliminary screening data.* | 1998 | study design |
| Cicero A. | *Short-term effect of a combined nutraceutical on insulin-sensitivity and related parameters in subjects with ifg and metabolic syndrome: A double-blind, randomized, clinical trial.* | 2013 | population |
| Conlon D. | *DREAM- A diabetes prevention study* | 2006 | overlapping population |
| Cooreman M. | *Lanifibranor reverses fasting glucose levels to normoglycemia in prediabetic patients with nonalcoholic steatohepatitis (nash).* | 2021 | study design |
| Coppell K. | *The effectiveness of a primary care nursing-led dietary intervention for prediabetes: a mixed methods pilot study.* | 2017 | study design |
| Corpeleijn E. | *Lifestyle intervention and adipokine levels in subjects at high risk for type 2 diabetes: the Study on Lifestyle intervention and Impaired glucose tolerance Maastricht (SLIM).* | 2007 | overlapping population |
| Cos X. | *Significant type 2 diabetes incidence reduction in high risk participants, after three years of an intensive lifestyle intervention in primary care (DE-PLAN-CAT).* | 2010 | study design |
| Crandall J. | *Statin use and risk of developing diabetes: results from the Diabetes Prevention Program.* | 2017 | study design |
| Davidson M. | *The effect of high dose vitamin d supplementation in people with pre-diabetes and hypovitaminosis d.* | 2012 | study design |
| Davidson M. | *High-dose vitamin D supplementation in people with prediabetes and hypovitaminosis D.* | 2013 | population |
| Davidson M. | *Effect of colesevelam and niacin on low-density lipoprotein cholesterol and glycemic control in subjects with dyslipidemia and impaired fasting glucose.* | 2013 | population |
| Davidson M. | *The effect of colesevelam and niacin on low-density lipoprotein cholesterol and plasma glucose.* | 2012 | study design |
| Davies M. | *A community-based primary prevention programme for type 2 diabetes in the UK: A cluster randomised controlled trial.* | 2015 | study design |
| Dawson-Hughes B. | *Intratrial exposure to vitamin d and new-onset diabetes among adults with prediabetes: A secondary analysis from the vitamin d and type 2 diabetes (d2d) study.* | 2020 | overlapping population |
| De Bie T. | *12-Week Oral GABA Supplementation Reduces Fasting Glucose Concentration But Does Not Change Postprandial Glucose Response In Prediabetic Adults: A Double-Blind, Randomized, Placebo-Controlled Trial.* | 2023 | study design |
| Defronzo R. | *Prevention of diabetes with pioglitazone in ACT NOW: physiologic correlates.* | 2013 | overlapping population |
| Den Hertog H. | *Safety and feasibility of treatment with metformin in patients with TIA or minor ischemic stroke and impaired glucose tolerance: A randomized, open-label phase II trial.* | 2009 | study design |
| Derosa G. | *Nateglinide does not reduce the incidence of diabetes or cardiovascular outcomes in people with impaired glucose tolerance and cardiovascular disease or risk factors.* | 2011 | study design |
| Derosa G. | *Ascophyllum nodosum and Fucus vesiculosus on glycemic status and on endothelial damage markers in dysglicemic patients.* | 2019 | intervention |
| Derosa G. | *Metabolic Actions of a Supplement of Ilex Paraguariensis (An Extract of the Leaf Standardized to 2% I-Deoxinojirimcina), White Mulberry and Chromium Picolinate in Nondiabetic Subects with Dysglycemia: A Randomized Trial.* | 2021 | intervention |
| Derosa G. | *Ilex paraguariensis, white mulberry and chromium picolinate in patients with pre-diabetes. Phytotherapy Research,* | 2020 | intervention |
| Dorans K. | *A Randomized Trial of Low-Carbohydrate Diet on Hemoglobin A1c among Individuals with Elevated Hemoglobin A1c.* | 2022 | study design |
| Dos Santos M. | *International variation in outcomes among people with cardiovascular disease or cardiovascular risk factors and impaired glucose tolerance: Insights from the NAVIGATOR trial.* | 2017 | overlapping population |
| Duijzer G. | *SLIMMER: a randomised controlled trial of diabetes prevention in Dutch primary health care: design and methods for process, effect, and economic evaluation.* | 2014 | study design |
| Duijzer G. | *Type 2 diabetes prevention from evidence to practice: the SLIMMER lifestyle intervention.* | 2015 | study design |
| Durbin R. | *Thiazolidinedione therapy in the prevention/delay of type 2 diabetes in patients with impaired glucose tolerance and insulin resistance.* | 2004 | intervention |
| Duru O. | *The Effectiveness of Shared Decision Making for Diabetes Prevention: 24- and 36-Month Results From the Prediabetes Informed Decision and Education (PRIDE) Trial.* | 2023 | study design |
| Dutta D. | *Vitamin-D supplementation in prediabetes reduced progression to type2 diabetes through decreased insulin resistance and systemic inflammation: An open label randomized prospective study from Eastern India.* | 2014 | duplicat |
| Edelstein S. | *Lack of durable improvements in β-cell function following withdrawal of pharmacological interventions in adults with impaired glucose tolerance or recently diagnosed type 2 diabetes.* | 2019 | population |
| Edelstein S. | *Effects of treatment of impaired glucose tolerance or recently diagnosed type 2 diabetes with metformin alone or in combination with insulin glargine on β-cell function: Comparison of responses in youth and adults: The RISE consortium.* | 2019 | population |
| Eguchi K. | *Comparison of the effects of pioglitazone and metformin on insulin resistance and hormonal markers in patients with impaired glucose tolerance and early diabete.* | 2007 | population |
| Eguchi K. | *Comparison of the effects of pioglitazone and metformin on insulin resistance and hormonal markers in patients with impaired glucose tolerance and early diabetes.* | 2007 | duplicat |
| Espinoza S. | *Pioglitazone is equally effective for preventing diabetes in older compared to younger pre-diabetic adults.* | 2015 | study design |
| Espinoza S. | *Pioglitazone is equally effective for diabetes prevention in older compared with younger prediabetic adults.* | 2015 | study design |
| Espinoza S. | *Pioglitazone is equally effective for diabetes prevention in older versus younger adults with impaired glucose tolerance.* | 2016 | overlapping population |
| Færch K. | *Effects of dapagliflozin, metformin, or exercise on glucose metabolism in individuals with prediabetes: The pre-d trial.* | 2019 | study design |
| Faerch K. | *Effects of dapagliflozin, metformin or exercise on glucosemetabolism and glycaemic variability in individuals with prediabetes: the PRE-D trial.* | 2019 | study design |
| Faghihimani E. | *The effects of salsalate on biochemical factors of serum and endothelial dysfunction in patients with newly diagnosed type 2 diabetes.* | 2012 | outcomes of interest not reported |
| Fakhri M. | *The effect of salsalate on biochemical factors and endothelial dysfunction of prediabetic patients: A randomized clinical trial.* | 2014 | population |
| Farkouh M. | *Valsartan reduced diabetes but not CV events in patients with impaired glucose tolerance and CV risk factors: Commentary*. | 2010 | study design |
| Farmer J. | *Effect of ramipril on the incidence of diabetes (DREAM study).* | 2007 | study design |
| Fasching P. | *Metabolic effects of fish-oil supplementation in patients with impaired glucose tolerance.* | 1991 | study design |
| Feigl A. | *Microclinic social network interventions for obesity and diabetes in amman, Jordan: a 3-armed cluster randomized controlled trial of 28-week intervention and 12-month follow-up.* | 2015 | study design |
| Ferrannini G. | *Empagliflozin improves insulin sensitivity in patients with a recent coronary syndrome and newly detected dysglycaemia.* | 2023 | study design |
| Fine A. | *Prevention in Prison: The Diabetes Prevention Program in a Correctional Setting.* | 2019 | population |
| Florentin M. | *Colesevelam plus rosuvastatin 5 mg/Day versus rosuvastatin 10 mg/Day alone on markers of insulin resistance in patients with hypercholesterolemia and impaired fasting glucose.* | 2013 | duplicat |
| Flores-Saenz J. | *Crossover and Double Blind Study with Metformin and Rosiglitazone in Impaired Glucose Tolerance Subjects.* | 2003 | outcomes of interest not reported |
| Florez H. | *Metabolic benefits of a 2-year lifestyle intervention in the clinical setting: Diabetes prevention strategies in latinos from Venezuela, South America.* | 2011 | study design |
| Florez H. | *Lifestyle intervention improves global cardiovascular health in latinos with prediabetes in maracaibo, venezuela.* | 2012 | study design |
| Ford C. | *Dietary changes in a diabetes prevention intervention among people with prediabetes: the Diabetes Community Lifestyle Improvement Program trial.* | 2019 | population |
| Forouhi N. | *Effects of vitamin D2 or D3 supplementation on glycaemic control and cardiometabolic risk among people at risk of type 2 diabetes: results of a randomized double-blind placebo-controlled trial.* | 2016 | population |
| Fortin E. | *Empagliflozin improves insulin sensitivity in patients with recent acute coronary syndrome and newly detected dysglycaemia : Experiences from the randomized, controlled SOCOGAMI trial.* | 2023 | population |
| Francois M. | *Short-term interval and continuous training improves pancreatic β-cell function adjusted for skeletal muscle insulin resistance in adults with prediabetes.* | 2018 | study design |
| Fraticelli F. | *A nutritional web-based approach in obesity and diabetes before and during COVID-19 lockdown.* | 2020 | population |
| Fraticelli F. | *A nutritional web-based approach in obesity and diabetes before and during the COVID-19 lockdown.* | 2023 | duplicat |
| Fujioka K. | *Liraglutide 3.0 mg reduces body weight and improves cardiometabolic risk factors in adults with obesity or overweight and prediabetes: the scale obesity and prediabetes randomized, double-blind, placebo-controlled 3-year trial.* | 2016 | study design |
| Fujioka K. | *Early weight loss responders to liraglutide 3.0 mg achieved greater weight loss and regression to normoglycemia, and reduced development of T2D, at 3 years versus early non-responders in the scale obesity and prediabetes trial.* | 2016 | study design |
| Fujioka K. | *Early Weight Loss with Liraglutide 3.0 mg Predicts 1-Year Weight Loss and is Associated with Improvements in Clinical Markers.* | 2016 | study design |
| Fujioka K. | *Efficacy and safety of liraglutide 3.0 mg for weight management in overweight/obese adults: The SCALE obesity and prediabetes trial.* | 2014 | study design |
| Fujita Y. | *Effects of dietary instructions including meal sequencing in prediabetic subjects.* | 2018 | study design |
| Fukuda H. | *Evaluation of a diabetes patient education program consisting of a three-day hospitalization and a six-month follow-up by telephone counseling for mild type 2 diabetes and IGT.* | 1999 | population |
| Gabriel R. | *Reduction in the Risk of Peripheral Neuropathy and Preservation of Kidney Function with Metformin, Linagliptin, or Their Fixed-Dose Combination, Compared with Placebo in Prediabetes-A Randomized Controlled Trial*. | 2023 | study design |
| Gadde K. | *A low-dose, controlled-release formulation of phentermine/topiramate (PHEN/TPM) demonstrates significant weight loss, clinical improvement in overweight/obese patients with type 2 diabetes mellitus (T2DM) or prediabetes*. | 2010 | study design |
| Garcia A. | *Arabinoxylan consumption decreases postprandial serum glucose, serum insulin and plasma total ghrelin response in subjects with impaired glucose tolerance.* | 2007 | overlapping population |
| García-Serrano C. | *Enhancing Night and Day Circadian Contrast through Sleep Education in Prediabetes and Type 2 Diabetes Mellitus: A Randomized Controlled Trial* | 2022 | population |
| Gardner C. | *Effect of a ketogenic diet versus Mediterranean diet on glycated hemoglobin in individuals with prediabetes and type 2 diabetes mellitus: the interventional Keto-Med randomized crossover trial.* | 2022 | population |
| Garrib A. | *Metformin for the prevention of diabetes among people with HIV and either impaired fasting glucose or impaired glucose tolerance (prediabetes) in Tanzania: a Phase II randomised placebo-controlled trial.* | 2023 | population |
| Garvey W. | *Diabetes prevention in subjects with prediabetes using controlled-release phentermine/topiramate (PHEN/TPM CR) over 2 years.* | 2012 | study design |
| Garvey W. | *Weight-loss with low-dose, controlled-release phentermine/topiramate therapy improves glycaemic status and prevents progression to type 2 diabetes mellitus.* | 2011 | study design |
| Garvey W. | *Prevention of type 2 diabetes in subjects with prediabetes and metabolic syndrome treated with phentermine and topiramate extended release.* | 2014 | population |
| Gastaldelli A. | *Improvement in hepatic metabolism is associated with reduced conversion to diabetes in IGT subjects treated with pioglitazone (ACT NOW study).* | 2015 | study design |
| Geirsdottir O. | *Effect of 12-week resistance exercise program on body composition, muscle strength, physical function, and glucose metabolism in healthy, insulin-resistant, and diabetic elderly Icelanders.* | 2012 | population |
| Gerstein H. | *Basal insulin and cardiovascular and other outcomes in dysglycemia* | 2012 | population |
| Gerstein H. | *Effect of rosiglitazone on the frequency of diabetes in patients with impaired glucose tolerance or impaired fasting glucose: a randomised controlled trial.* | 2006 | intervention |
| Gibbs D. | *Can combination therapy prevent progression to type 2 diabetes?* | 2011 | study design |
| Goldfine A. | *A targeting inflammation using salsalate to prevent diabetes.* | 2011 | study design |
| Gong Q. | *Efficacy of lifestyle intervention in preventing diabetes in people with IGT with and without impaired fasting glucose: A secondary analysis of Da Qing diabetes prevention outcomes study (DQDPOS).* | 2020 | study design |
| Gong Q. | *Efficacy of lifestyle intervention in adults with impaired glucose tolerance with and without impaired fasting plasma glucose: A post hoc analysis of Da Qing Diabetes Prevention Outcome Study.* | 2021 | outcomes of interest not reported |
| Gong Q. | *Morbidity and mortality after lifestyle intervention for people with impaired glucose tolerance: 30-year results of the Da Qing Diabetes Prevention Outcome Study.* | 2019 | outcomes of interest not reported |
| Gonzalez S. | *The effect of six months of vitamin d supplemention in minorities with pre-diabetes and hypovitaminosis d.* | 2012 | study design |
| González-Ortiz M. | *Lixisenatide vs. exenatide on metabolic control, insulin secretion, and insulin sensitivity in impaired glucose tolerance patients.* | 2015 | study design |
| Gradinariu V. | *Effects of dietary quality, physical activity and weight loss on glucose homeostasis in persons with and without prediabetes in the PREMIER trial.* | 2023 | population |
| Grubina R. | *Insulin pensitizer therapy improves atherothrombotic and inflammatory biomarkers.* | 2012 | study design |
| Guardado-Mendoza R. | *Effect of linagliptin + metformin vs. Metformin alone to improve glucose metabolism and beta-cell function in prediabetes.* | 2017 | study design |
| Guardado-Mendoza R. | *Diabetes prevention with lifestyle, linagliptin and metformin in patients with prediabetes: the PRELLIM project.* | 2018 | study design |
| Guiru W. | *Study on influence of intensive lifestyle intervention to blood glucose for patients with coronary heart disease combined impaired fasting glucose.* | 2014 | study design |
| Hage C. | *Sitagliptin improves beta-cell function in patients with acute coronary syndromes and newly diagnosed glucose abnormalities--the BEGAMI study.* | 2013 | population |
| Hage C. | *Sitagliptin improves beta-cell function in patients with acute coronary syndromes and newly diagnosed glucose abnormalities-the BEGAMI study.* | 2013 | duplicat |
| Hage C. | *The DPP-IV inhibitor sitagliptin improves beta-cell function in patients with acute coronary syndromes and newly diagnosed glucose abnormalities.* | 2012 | study design |
| Hage C. | *Sitagliptin improves beta-cell function and glucose metabolism in patients with acute coronary syndromes and newly detected glucose perturbations.* | 2011 | study design |
| Hahn M. | *Metformin for Early Onset Comorbid Type 2 Diabetes or Prediabetes in Schizophrenia Spectrum Disorders: a Double-Blind Randomized Pilot Study.* | 2020 | study design |
| Haldar S. | *Plant-protein Rich, Low-calorie Diet to Improve Metabolic Health in Prediabetic Chinese Adults - A Randomized Controlled Trial.* | 2023 | study design |
| Handelsman Y. | *Colesevelam for hispanic patients with hypercholesterolemia and prediabetes.* | 2010 | study design |
| Handelsman Y. | *Colesevelam for Hispanic patients with hypercholesterolaemia and prediabetes.* | 2010 | study design |
| Handelsman Y. | *Use of phentermine/topiramate extended-release (PHEN/TPM ER) in obese/overweight subjects with dysglycemia.* | 2014 | study design |
| Handjieva-Darlenska T. | *Prevention of type 2 diabetes through diet and physical activity: the PREVIEW study.* | 2017 | study design |
| Hanf R. | *GFT505 improves glucose and lipid homeostasis in patients with impaired fasting glucose and impaired glucose tolerance.* | 2010 | study design |
| Hankosky E. | *Tirzepatide reduces the predicted risk of developing type 2 diabetes in people with obesity or overweight: Post hoc analysis of the SURMOUNT-1 trial.* | 2023 | population |
| Hariharan R. | *Carnosine supplementation improves glucose control in adults with pre-diabetes and type 2 diabetes: A randomised controlled trial, in medRxiv. B. de Courten, School of Health and Biomedical Sciences, RMIT University, Bundoora, Australia.* | 2023 | population |
| Hariharan R. | *Carnosine supplementation has glucose lowering effects even in prediabetes and type 2 diabetes.* | 2022 | study design |
| Harris K. | *Whole and refined grains have differential effects on cardiometabolic markers in overweight individuals with normal fasting glucose versus prediabetes.* | 2013 | study design |
| Heiman M. | *Improved oral glucose tolerance in prediabetics and type 2 diabetics (T2D) in a pilot clinical trial testing a novel gastrointestinal (GI) microbiome modulator.* | 2014 | study design |
| Hellgren M. | *Risk factors for progression to type 2 diabetes mellitus from prediabetes.* | 2020 | study design |
| Hellgren M. | *Feasibility of a controlled intervention with physical activity in individuals with impaired glucose tolerance, recruited by FINDRISC.* | 2013 | study design |
| Hermann L. | *Effects of antihyperglycaemic therapies on proinsulin and relation between proinsulin and cardiovascular risk factors in type 2 diabetes.* | 1999 | population |
| Hernandez-Alonso P. | *Effect of Pistachio Intake on Insulin Resistance and metabolic risk markers-the EPIRDEM Study.* | 2014 | study design |
| Hesselink A. | *Effectiveness of an intervention in primary care to prevent type 2 diabetes in people with impaired fasting glucose, a cluster-randomised controlled trial.* | 2013 | study design |
| Higashikawa F. | *5-aminolevulinic acid, a precursor of heme, reduces both fasting and postprandial glucose levels in mildly hyperglycemic subjects.* | 2013 | population |
| Hoellriegel R. | *Regular physical exercise training partially corrects metabolic alterations and improves peripheral endothelial function in pre-diabetic, adipose patients with coronary artery disease.* | 2011 | study design |
| Hoellriegel R. | *Regular physical exercise training improves diastolic function in pre-diabetic, adipose patients with coronary artery disease.* | 2011 | study design |
| Hoellriegel R. | *Short and long-term effects of regular physical exercise training on diastolic function in pre-diabetic, adipose patients with coronary artery disease.* | 2012 | study design |
| Hoellriegel R. | *Regular physical exercise training improves function of circulating endothelial progenitor cells and endothelial function in pre-diabetic, adipose patients with coronary heart disease.* | 2009 | study design |
| Hollander P. | *Sustained-release naltrexone/bupropion improves glucose control in individuals with prediabetes.* | 2014 | study design |
| Holman R. | *Evaluation of the glycemic effects and efficacy and safety of evolocumab (AMG 145) in subjects with or without dysglycemia or metabolic syndrome.* | 2015 | study design |
| Holman R. | *Incidence of diabetes following ramipril or rosiglitazone withdrawal.* | 2011 | study design |
| Home P. | *Low-dose rosiglitazone plus metformin reduces risk of incident type 2 diabetes compared with placebo in people with impaired glucose tolerance.* | 2011 | study design |
| Honig C. | *Differential effects of a two-year insoluble cereal fiber intervention in prediabetic subjects (the OPTImal FIber Trial - OPTIFIT).* | 2015 | study design |
| Horne B. | *Intermittent fasting interacts with red cell distribution width to differentially improve cardiometabolic health.* | 2021 | study design |
| Howard B. | *Postmenopausal hormone therapy is associated with atherosclerosis progression in women with abnormal glucose tolerance.* | 2004 | outcomes of interest not reported |
| Hummel J. | *Effects of empagliflozin on glucose metabolism and body fat composition in patients with prediabetes: A randomized, double-blind, placebo-controlled trial.* | 2020 | study design |
| Idris I. | *Semaglutide 2.4 mg improved glucose metabolism and reverted prediabetes to normoglycaemia in adults with overweight or obesity vs liraglutide 3.0 mg (STEP 8).* | 2023 | study design |
| Igata S. | *Visceral fat metabolic activity is independently associated with coronary artery inflammation in patients with impaired glucose intolerance or type 2 diabetes.* | 2014 | study design |
| Imai S. | *Randomized controlled trial of two forms of self-management group education in Japanese people with impaired glucose tolerance.* | 2008 | population |
| Inzucchi S. | *Ada presidents' select abstract: effect of dapagliflozin on the incidence of diabetes: a prespecified exploratory analysis from dapa-hf.* | 2020 | study design |
| Inzucchi S. | *Diabetes prevention with pioglitazone in insulin resistant patients with cerebrovascular disease.* | 2016 | study design |
| Inzucchi S. | *Pioglitazone Prevents Diabetes in Patients With Insulin Resistance and Cerebrovascular Disease.* | 2016 | population |
| Ipsen E. | *Pioglitazone for prevention or delay of type 2 diabetes mellitus and its associated complications in people at risk for the development of type 2 diabetes mellitus.* | 2020 | study design |
| Ishikawa S. | *Impact of sitagliptin on carotid intima-media thickness in patients with coronary artery disease and impaired glucose tolerance or mild diabetes mellitus.* | 2014 | population |
| Islek D. | *Cost-effectiveness of the stepwise approach to diabetes prevention in India.* | 2020 | population |
| Islek D. | *Cost-effectiveness of a Stepwise Approach vs Standard Care for Diabetes Prevention in India.* | 2020 | population |
| Izadi I. | *Glucoherb versus metformin on glycemic markers and glycosylated hemoglobin in prediabetes patients; a clinical trial study.* | 2020 | population |
| Jagannathan R. | *Clinical utility of 30-min plasma glucose for prediction of type 2 diabetes among people with prediabetes: Ancillary analysis of the diabetes community lifestyle improvement program.* | 2020 | overlapping population |
| Jeong S. | *Chitosan oligosaccharide (GO2KA1) improves postprandial glycemic response in subjects with impaired glucose tolerance and impaired fasting glucose and in healthy subjects: a crossover, randomized controlled trial.* | 2019 | population |
| Jiriyasin S. | *Metformin for preventing diabetes mellitus in HIV-infected patients with prediabetes: A randomized controlled trial.* | 2018 | study design |
| Kabisch S. | *Dose-dependent effects of insoluble fibre on glucose metabolism: a stratified post hoc analysis of the Optimal Fibre Trial (OptiFiT).* | 2021 | outcomes of interest not reported |
| Kabisch S. | *Effects of Insoluble Cereal Fibre on Body Fat Distribution in the Optimal Fibre Trial.* | 2021 | study design |
| Kabisch S. | *Investigation of sex-dependent effects of a one-year low-carb- vs Low-fat intervention in patients with high-risk prediabetes-A randomised controlled trial.* | 2021 | outcomes of interest not reported |
| Kabisch S. | *Obesity Does Not Modulate the Glycometabolic Benefit of Insoluble Cereal Fibre in Subjects with Prediabetes-A Stratified Post Hoc Analysis of the Optimal Fibre Trial (OptiFiT).* | 2019 | outcomes of interest not reported |
| Kabisch S. | *Predicting Factors for Metabolic Non-Response to a Complex Lifestyle Intervention-A Replication Analysis to a Randomized-Controlled Trial.* | 2022 | outcomes of interest not reported |
| Kajiyama S. | *Supplementation of hydrogen-rich water improves lipid and glucose metabolism in patients with type 2 diabetes or impaired glucose tolerance.* | 2008 | intervention |
| Kang J. | *Effect of a continuous diabetes lifestyle intervention program on male workers in Korea.* | 2010 | population |
| Kang R. | *Consumption of whole grains and legumes modulates the genetic effect of the APOA5 -1131C variant on changes in triglyceride and apolipoprotein A-V concentrations in patients with impaired fasting glucose or newly diagnosed type 2 diabetes.* | 2014 | population |
| Karan A. | *Impact of Food Order Behavioral Intervention on Weight and Nutrient Intake in Adults With Prediabetes.* | 2022 | study design |
| Karunapema P. | *Effectiveness of a simple lifestyle intervention in primary prevention of type 2 diabetes among semi-urban South Asian population with impaired fasting glucose*. | 2009 | study design |
| Kataoka Y. | *The comparable effect of lifestyle and pharmacological intervention to prevent coronary atherosclerosis in Japanese early-stage diabetic patients - The diabetes and diffuse coronary narrowing (DIANA) study.* | 2010 | study design |
| Katula J. | *Effects of a digital diabetes prevention program on hba1c and body weight in prediabetes.* | 2020 | study design |
| Katula J. | *Translating diabetes prevention: Healthy living partnerships to prevent diabetes (HELP PD).* | 2009 | study design |
| Katula J. | *Healthy Living Partnerships to Prevent Diabetes (HELP PD): design and methods.* | 2010 | study design |
| Kaur N. | *Diabetic yoga protocol improves glycemic, anthropometric and lipid levels in high risk individuals for diabetes: a randomized controlled trial from Northern India.* | 2021 | population |
| Kavitha Chandran et al. | *Primary prevention of type 2 diabetes mellitus: Multiple health care strategies.* | 2020 | study design |
| Kawahara T. | *Eldecalcitol, a vitamin D analog, for diabetes prevention in impaired glucose tolerance (DPVD study).* | 2018 | study design |
| Kawahara T. | *Eldecalcitol, a vitamin D analogue, for diabetes prevention in impaired glucose tolerance: DPVD study. Diabetologia,* | 2018 | study design |
| Kawahara T. | *Reduced progression to type 2 diabetes from impaired glucose tolerance after a 2-day in-hospital diabetes educational program.* | 2008 | duplicat |
| Kawamori R. | *Voglibose for prevention of type 2 diabetes mellitus: a randomised, double-blind trial in Japanese individuals with impaired glucose tolerance.* | 2009 | duplicat |
| Kawano H. | *Effects of combination lipid therapy on lipid profiles in patients with impaired glucose tolerance.* | 2013 | study design |
| Keech A. | *Secondary prevention of cardiovascular events with long-term pravastatin in patients with diabetes of impaired fasting glucose: Results from the LIPID trial.* | 2003 | duplicat |
| Kelly A. | *Randomized, Controlled Trial Of Sitagliptin And Islet Function In Cystic Fibrosis With Abnormal Glucose Tolerance* | 2021 | population |
| Kern P. | *Effects of KDT501 on metabolic parameters in insulin-resistant prediabetic humans.* | 2017 | population |
| Kern W. | *Reduction in the risk of developing type 2 diabetes (T2D) with liraglutide 3.0 mg in people with prediabetes from the SCALE Obesity and Prediabetes randomized, double-blind, placebo-controlled trial.* | 2016 | study design |
| Kessler K. | *Diurnal distribution of carbohydrates and fat affects substrate oxidation and adipokine secretion in humans. Am J Clin Nutr,* | 2018 | population |
| Kharmats A. | *A randomized clinical trial comparing low-fat with precision nutrition-based diets for weight loss: impact on glycemic variability and HbA1c.* | 2023 | duplicat |
| Kharmats A. | *A randomized clinical trial comparing low-fat with precision nutrition–based diets for weight loss: impact on glycemic variability and HbA1c.* | 2023 | duplicat |
| Kikuchi S. | *TCT-835 Impact of Early Intervention With Alogliptin on Coronary Plaque Regression in Patients with Acute Coronary Syndromes: a Prospective, Single-Center, Randomized Trial.* | 2019 | study design |
| Kim C. | *Reductions in glucose among postmenopausal women who use and do not use estrogen therapy.* | 2013 | study design |
| Kim H. | *The effects of pinitol on the glucose level, insulin resistance and the adipocytokine levels in patients who are in a prediabetic state.* | 2011 | study design |
| Kim M. | *Replacing with whole grains and legumes reduces Lp-PLA2 activities in plasma and PBMCs in patients with prediabetes or T2D.* | 2014 | population |
| Kim M. | *Replacing carbohydrate with protein and fat in prediabetes or type-2 diabetes: greater effect on metabolites in PBMC than plasma.* | 2016 | population |
| Kim S. | *Liraglutide for overweight, older individuals with prediabetes.* | 2013 | study design |
| Kimball S. | *Effect of a vitamin and mineral supplementation on glycemic status: Results from a community-based program.* | 2017 | study design |
| Kitabchi A. | *Remission of impaired glucose tolerance (IGT) to normal glucose tolerance (NGT) in obese adults with high protein vs. High carbohydrate diet.* | 2015 | study design |
| Knowler W. | *Persistent and Heterogeneous Long-Term Effects of the Diabetes Prevention Program (DPP) Intensive Lifestyle (ILS) Intervention on Diabetes Incidence.* | 2022 | study design |
| Ko K. | *Metformin intervention in obese non-diabetic patients with breast cancer: phase II randomized, double-blind, placebo-controlled trial.* | 2015 | population |
| Kobayakawa A. | *Improvement of fasting plasma glucose level after ingesting moderate amount of dietary fiber in Japanese men with mild hyperglycemia and visceral fat obesity.* | 2013 | population |
| Kodama N. | *Effects of pioglitazone versus glimepiride on abdominal fat glucose metabolism in patients with impaired glucose tolerance and/or type 2 diabetes mellitus.* | 2012 | study design |
| Kodama N. | *Effects of pioglitazone on visceral fat metabolic activity in impaired glucose tolerance or type 2 diabetes mellitus.* | 2013 | population |
| Kokun O. | *SCHOOLTEACHERS' PERSONAL-PROFESSIONAL HEALTH-PROTECTING FACTORS.* | 2023 | study design |
| Komiyama N. | *Effects of a 4-week 70% high carbohydrate/15% low fat diet on glucose tolerance and on lipid profiles.* | 2004 | population |
| Komorowski J. | *Chromium supplementation does not improve glucose tolerance, insulin sensitivity, or lipid profile: a randomized, placebo-controlled, double-blind trial of supplementation in subjects with impaired glucose tolerance: response to Gunton et al.* | 2005 | study design |
| Konopka A. | *Does metformin antagonize glucagon-induced endogenous glucose production in humans? Diabetes,* | 2015 | study design |
| Koska J. | *Effect of salsalate on insulin secretion and clearance in individuals with impaired glucose metabolism.* | 2011 | study design |
| Koyasu M. | *Impact of acarbose on carotid intima-media thickness in patients with newly diagnosed impaired glucose tolerance or mild type 2 diabetes mellitus: A one-year, prospective, randomized, open-label, parallel-group study in Japanese adults with established coronary artery disease*. | 2010 | population |
| Kramer M. | *Evaluation of a Diabetes Prevention Program Lifestyle Intervention in Older Adults: A Randomized Controlled Study in Three Senior/Community Centers of Varying Socioeconomic Status. Diabetes Educ,* | 2018 | population |
| Kramer M. | *Launching a lifestyle intervention program for diabetes prevention at the worksite.* | 2013 | study design |
| Kramer M. | *Evaluation of group lifestyle balance long-term maintenance strategies.* | 2011 | study design |
| Kraus W. | *Relationship between baseline physical activity assessed by pedometer count and new-onset diabetes in the NAVIGATOR trial.* | 2018 | study design |
| Kulzer B. | *Primary prevention of type 2 diabetes by lifestyle modification. Results of the PRAEDIAS prevention program.* | 2014 | overlapping population |
| Kumar M. | *Vitamin-D improves glycaemic outcome in prediabetes through reduced fetuin-A and systemic inflammation.* | 2018 | study design |
| Kumar M. | *Vitamin D improves glycemic outcomes in prediabetes through reduced fetuin-a and systemic inflammation.* | 2019 | study design |
| Kushner R. | *Effects of weight loss on prevention of type 2 diabetes mellitus in subjects with prediabetes using phentermine and topiramate extendedrelease for 2 years* | 2013 | study design |
| Kwak J. | *Dietary treatment with rice containing resistant starch improves markers of endothelial function with reduction of postprandial blood glucose and oxidative stress in patients with prediabetes or newly diagnosed type 2 diabetes.* | 2012 | population |
| Kwiecinska E. | *How can a primary care physician easily modify lifestyle of overweight subjects who are at risk of diabetes?* | 2012 | study design |
| Landry M. | *Effect of a ketogenic diet vs mediterranean diet on hba1c: the keto-med randomized crossover trial* | 2021 | study design |
| Larsen A. | *Metformin reduces myocardial oxygen consumption and improves myocardial efficiency in pre-diabetic heart failure patients* | 2019 | study design |
| Larsen J. | *Effect of Liraglutide Treatment on Prediabetes and Overweight or Obesity in Clozapine- or Olanzapine-Treated Patients With Schizophrenia Spectrum Disorder: A Randomized Clinical Trial* | 2017 | population |
| Larsen J. | *The GLP-1 analog liraglutide improves glucose tolerance and reduces body weight in schizophrenia spectrum disorder patients treated with clozapine or olanzapine* | 2017 | study design |
| Lau C. | *The effects of adding group-based lifestyle counselling to individual counselling on changes in plasma glucose levels in a randomized controlled trial: the Inter99 study* | 2011 | population |
| Lau D. | *Hypoglycemia reported with liraglutide 3.0 mg in overweight and obese adults with and without prediabetes: Results of the randomized, double-blind, placebo-controlled 56-week scale obesity and prediabetes trial* | 2015 | study design |
| Lau D. | *Characteristics of individuals developing type 2 diabetes in the scale obesity and prediabetes randomized, double-blind, liraglutide vs. placebo trial* | 2016 | study design |
| Lau D. | *Liraglutide 3.0 mg improves insulin secretion and action in overweight/ obese adults without type 2 diabetes: The SCALE obesity and prediabetes trial* | 2014 | study design |
| Le Roux C. | *Reduced risk of developing type 2 diabetes (T2D) with liraglutide 3.0 mg for weight management: Results from the SCALE obesity and prediabetes randomized, double-blind, placebo-controlled 3-year trial* | 2016 | study design |
| Le Roux C. | *Liraglutide 3.0 mg reduces body weight and improves cardiometabolic risk factors: SCALE obesity and prediabetes randomised, double-blind, placebo-controlled 3-year trial* | 2016 | study design |
| Leiter L. | *Efficacy and safety of alirocumab in people with prediabetes vs those with normoglycaemia at baseline: a pooled analysis of 10 phase III ODYSSEY clinical trials* | 2018 | study design |
| Lemieux P. | *Effects of 6-month Vitamin D supplementation on insulin sensitivity and secretion: A randomised, placebo-controlled trial* | 2019 | population |
| Lewis K. | *Weight loss achieved with medication can delay onset of type 2 diabetes in at-risk individuals* | 2014 | population |
| Li G. | *Is lifestyle modification combined with blood pressure lowering medication a better strategy for the intervention targeting CVD risk reduction in Chinese pre-diabetes population? Lesson from the China Da Qing diabetes prevention outcome study (CDQDPOS)* | 2009 | study design |
| Li G. | *Cardiovascular mortality, all-cause mortality, and diabetes incidence after lifestyle intervention for people with impaired glucose tolerance in the Da Qing Diabetes Prevention Study: a 23-year follow-up study* | 2014 | outcomes of interest not reported |
| Li G. | *The long-term effect of lifestyle interventions to prevent diabetes in the China Da Qing Diabetes Prevention Study: a 20-year follow-up study* | 2008 | outcomes of interest not reported |
| Li J. | *Effects of a low-carbohydrate/high-protein diet on metabolic health in individuals with chronic spinal cord injury: An exploratory analysis of results from a randomized controlled trial* | 2022 | population |
| Li S. | *Lifestyle intervention, metformin, and acarbose treatments differentially impact liver fat content, serum lipids, and hormone profiles in obese polycystic ovary syndrome patients with impaired glucose tolerance* | 2020 | study design |
| Li X. | *Higher blood pressure and predicted rate of diabetes in people with impaired glucose tolerance enhance the subsequent long-term risk of stroke: 23 year follow-up of the Daqing Diabetes Prevention Study* | 2017 | study design |
| Li X. | *Higher blood pressure predicts diabetes and enhances long-term risk of cardiovascular disease events in individuals with impaired glucose tolerance: Twenty-three-year follow-up of the Daqing diabetes prevention study* | 2019 | outcomes of interest not reported |
| Lindström J. | *Sustained reduction in the incidence of type 2 diabetes by lifestyle intervention: follow-up of the Finnish Diabetes Prevention Study* | 2006 | overlapping population |
| Lindström J. | *The Finnish Diabetes Prevention Study (DPS): Lifestyle intervention and 3-year results on diet and physical activity* | 2003 | overlapping population |
| Lindström J. | *High-fibre, low-fat diet predicts long-term weight loss and decreased type 2 diabetes risk: the Finnish Diabetes Prevention Study* | 2006 | overlapping population |
| Liontos A. | *Comparative effect of delapril-manidipine treatment versus telmisartan-amlodipine treatment in fasting glucose, fasting insulin, OGTT and HbA1c levels, in prediabetic hypertensive patients* | 2022 | study design |
| Liontos A. | *Comparative effect of delapril-manidipine treatment versus valsartan-amlodipine treatment in fasting glucose, fasting insulin, OGTT and HbA1c levels, in prediabetic hypertensive patients* | 2022 | study design |
| Liontos A. | *Comparative effect of valsartan-amlodipine treatment versus telmisartan-amlodipine treatment in fasting glucose, fasting insulin, OGTT and HbA1c levels, in prediabetic hypertensive patients* | 2022 | study design |
| Liontos A. | *Comparative effect of 3 different fix-combination antihypertensive treatments in fasting glucose, fasting insulin, OGTT and HbA1c levels, in prediabetic hypertensive patients* | 2022 | study design |
| Liping Y. | *Role of Purified Anthocyanins in Improving Cardiometabolic Risk Factors in Chinese Men and Women with Prediabetes or Early Untreated Diabetes--A Randomized Controlled Trial* | 2017 | population |
| Liu K. | *Effects of intermittent fasting compared to daily calorie restriction on intermediary metabolism in humans* | 2022 | study design |
| Liu L. | *The effects of resistance training on metabolic control in patients with prediabetes* | 2014 | study design |
| Liu X. | *Qi-gong mind-body therapy and diabetes control. A randomized controlled trial* | 2011 | population |
| Liu Y. | *A Dietary Supplement Containing Cinnamon, Chromium and Carnosine Decreases Fasting Plasma Glucose and Increases Lean Mass in Overweight or Obese Pre-Diabetic Subjects: A Randomized, Placebo-Controlled Trial* | 2015 | population |
| Liu Z. | *Effects of soy protein and isoflavones on glycemic control and insulin sensitivity: a 6-mo double-blind, randomized, placebo-controlled trial in postmenopausal Chinese women with prediabetes or untreated early diabetes* | 2010 | population |
| Ljunghall S. | *Treatment with one-alpha-hydroxycholecalciferol in middle-aged men with impaired glucose tolerance. A prospective randomized double-blind study* | 1987 | population |
| Lou Q. | *beta-cell function protection and metabolic effects of 6-month resistance training and aerobic training in prediabetic subjects: a randomized, multicenter controlled trial* | 2016 | study design |
| Lou Q. | *β-cell function protection and metabolic effects of 6-month resistance training and aerobic training in prediabetic subjects: a randomized, multicenter controlled trial* | 2016 | study design |
| Lu Y. | *Outcome of intensive integrated intervention in participants with impaired glucose regulation in China* | 2011 | population |
| Luo Y. | *Rationale, Design, and Baseline Characteristics of Beijing Prediabetes Reversion Program: A Randomized Controlled Clinical Trial to Evaluate the Efficacy of Lifestyle Intervention and/or Pioglitazone in Reversion to Normal Glucose Tolerance in Prediabetes* | 2017 | study design |
| Luo Y. | *Study design and baseline characteristics of beijing pre-diabetes reversion program (BPRP)* | 2012 | study design |
| Maccubbin D. | *Effects of niacin on the incidence of new onset diabetes and cardiovascular events in patients with normal and impaired glucose* | 2010 | study design |
| Mackerras D. | *Does a combined program of dietary modification and physical activity or the use of metformin reduce the conversion from impaired glucose tolerance to type 2 diabetes?* | 2003 | study design |
| Madhu S. | *A multicentric, randomized, controlled trial of yoga and fenugreek in prevention of type 2 diabetes mellitus: methodological details—the Indian Prevention of Diabetes Study (IPDS)* | 2023 | study design |
| Madhu S. | *Role of yoga in prevention of type 2 diabetes mellitus-interim results from the Indian prevention of diabetes study (IPDS)* | 2018 | study design |
| Madsbad S. | *Comparable efficacy and safety of liraglutide 3.0 mg for weight management across baseline BMI subgroups: Results from the SCALE obesity and prediabetes trial* | 2016 | study design |
| Maeda H. | *Effects of agar (kanten) diet on obese patients with impaired glucose tolerance and type 2 diabetes* | 2005 | population |
| Malin S. | *Impact of short-term exercise training intensity on β-cell function in older obese adults with prediabetes* | 2018 | overlapping population |
| Malin S. | *Two weeks of interval vs. Continuous exercise training on endothelial function in adults with prediabetes* | 2018 | study design |
| Marbury T. | *A pilot study to examine the feasibility of insulin glargine in subjects with impaired fasting glucose, impaired glucose tolerance or new-onset type 2 diabetes* | 2008 | population |
| Marina A. | *Colesevelam improves oral but not intravenous glucose tolerance by a mechanism independent of insulin sensitivity and β-cell function* | 2012 | study design |
| Martín-Frías M. | *Premeal repaglinide in impaired glucose tolerance in cystic fibrosis* | 2010 | study design |
| Martínez-Abundis E. | *Effect of linagliptin on glycemic control and variability in patients with impaired glucose tolerance* | 2015 | study design |
| Mathrani A. | *Effect of a 12-Week Polyphenol Rutin Intervention on Markers of Pancreatic β-Cell Function and Gut Microbiota in Adults with Overweight without Diabetes* | 2023 | outcomes of interest not reported |
| McDermott K. | *A yoga intervention for type 2 diabetes risk reduction: a pilot randomized controlled trial* | 2014 | population |
| McFarlane S. | *Diabetes prevention between RAAS inhibition and PPAR-gamma stimulation: the diabetes reduction assessment with ramipril and rosiglitazone medication (DREAM) trial* | 2007 | study design |
| Meyer N. | *Metabolic response to cereal fiber supplementation in subjects with prediabetes is depending on baseline glycemic and anthropometric status (OptiFiT)* | 2019 | study design |
| Mezones-Holguín E. | *Effectiveness of a clinic-based intensive lifestyle intervention to reduce the framingham diabetes risk score in latino patients with prediabetes* | 2011 | study design |
| Minicucci L. | *Slow-release insulin in cystic fibrosis patients with glucose intolerance: a randomized clinical trial* | 2012 | population |
| Misir S. | *Colesevelam HCl to treat hypercholesterolemia and improve glycemia in prediabetes: A randomized, prospective study* | 2010 | study design |
| Mizoguchi M. | *Pioglitazone attenuates atherosclerotic plaque inflammation in patients with impaired glucose tolerance or diabetes a prospective, randomized, comparator-controlled study using serial FDG PET/CT imaging study of carotid artery and ascending aorta* | 2011 | population |
| Moin T. | *Diabetes Prevention Program Translation in the Veterans Health Administration* | 2017 | study design |
| Molfino A. | *Caloric restriction and L-carnitine administration improves insulin sensitivity in patients with impaired glucose metabolism* | 2010 | population |
| Molitch M. | *The diabetes prevention program and its global implications* | 2003 | overlapping population |
| Moon K. | *Valsartan may decrease the incidence of diabetes mellitus* | 2011 | study design |
| Moreira-Lucas T. | *Effect of vitamin D fortified cheese on oral glucose tolerance in individuals exhibiting marginal vitamin D status and an increased risk for developing type 2 diabetes: A double-blind, randomized placebo-controlled clinical trial* | 2016 | study design |
| Moreira-Lucas T. | *Effect of vitamin D supplementation on oral glucose tolerance in individuals with low vitamin D status and increased risk for developing type 2 diabetes (EVIDENCE): A double-blind, randomized, placebo-controlled clinical trial* | 2017 | population |
| n R. | *Prevention Program of Diabetes in São Paulo* | 2013 | study design |
| Nagarathna R. | *Diabetes prevention through yoga-based lifestyle: A pan-India randomized controlled trial* | 2019 | study design |
| Naito E. | *Effect of Lactobacillus casei strain Shirota-fermented milk on metabolic abnormalities in obese prediabetic Japanese men: a randomised, double-blind, placebo-controlled trial* | 2018 | population |
| Nam S. | *Effect of a lifestyle weight loss intervention on visceral fat and glycemic control among individuals with and without type 2 diabetes* | 2015 | study design |
| Nanditha A. | *Impact of lifestyle intervention in primary prevention of Type 2 diabetes did not differ by baseline age and BMI among Asian-Indian people with impaired glucose tolerance* | 2016 | study design |
| NA | *Life Style Modifications Prevents Type 2 Diabetes in Asian Indians* | 2006 | study design |
| NA | *Effects of Pinitol on Hidrocarbonated Metabolism Parameters in Diabetic, Impaired and Normal Fasting Glucose Subjects* | 2012 | study design |
| Nemoto K. | *Should we intervene japanese adults with impaired glucose tolerance who have body mass indexes under 25 kg/m2? The kanagawa diabetes prevention program* | 2010 | study design |
| Nesto R. | *Evaluation of lorcaserin on progression of prediabetes to type 2 diabetes and reversion to euglycemia* | 2016 | study design |
| Nimitphong H. | *Metformin for Preventing Progression From Prediabetes to Diabetes Mellitus in People Living With Human Immunodeficiency Virus* | 2022 | population |
| Niskanen L. | *Liraglutide once daily reduces the prevalence of prediabetes and metabolic syndrome in obese non-diabetic adults: A 2-year randomized trial* | 2010 | study design |
| Nitta Y. | *Effects of pioglitazone on coronary arterial inflammation evaluated by serial FDG-PET/CT in patients with impaired glucose tolerant or type 2 diabetes* | 2012 | study design |
| Nitta Y. | *Pioglitazone decreases coronary artery inflammation in impaired glucose tolerance and diabetes mellitus: evaluation by FDG-PET/CT imaging* | 2013 | population |
| O'Dea A. | *An evaluation of Croí MyAction community lifestyle modification programme compared to standard care to reduce progression to diabetes/prediabetes in women with prior gestational diabetes mellitus (GDM)* | 2014 | study design |
| Odawara M. | *Pitavastatin reduced C-reactive protein without increasing the incidence of diabetes in Japanese individuals with impaired glucose tolerance (J-PREDICT study)* | 2014 | study design |
| Odawara M. | *Effect of pitavastatin on the incidence of diabetes in Japanese individuals with impaired glucose tolerance* | 2013 | study design |
| Ofili E. | *Metabolic effects associated with adding an angiotensin receptor blocker to a diuretic in prediabetic, obese African American patients with hypertension* | 2009 | study design |
| Oh M. | *Postprandial glucose-lowering effects of fermented red ginseng in subjects with impaired fasting glucose or type 2 diabetes: a randomized, double-blind, placebo-controlled clinical trial* | 2014 | intervention |
| Oikawa S. | *Effect of cilostazol on lipid, uric acid and glucose metabolism in patients with impaired glucose tolerance or type 2 diabetes mellitus a double-blind, placebo-controlled study* | 2001 | population |
| Oldroyd J. | *Randomised controlled trial evaluating the effectiveness of behavioural interventions to modify cardiovascular risk factors in men and women with impaired glucose tolerance: outcomes at 6 months* | 2001 | duplicat |
| Osmundson S. | *Early treatment of women with prediabetes in pregnancy: A randomized controlled trial* | 2015 | study design |
| Osmundson S. | *Early Screening and Treatment of Women with Prediabetes: A Randomized Controlled Trial* | 2015 | outcomes of interest not reported |
| Östman E. | *A dietary exchange of common bread for tailored bread of low glycaemic index and rich in dietary fibre improved insulin economy in young women with impaired glucose tolerance* | 2006 | outcomes of interest not reported |
| Palacios T. | *The effect of a multi-strain probiotic on metabolic bio-markers in adults with prediabetes and recently diagnosed type 2 diabetes mellitus (T2DM)* | 2016 | study design |
| Palacios T. | *Targeting the Intestinal Microbiota to Prevent Type 2 Diabetes and Enhance the Effect of Metformin on Glycaemia: A Randomised Controlled Pilot Study* | 2020 | population |
| Palermo A. | *Management of impaired glucose tolerance using buccal spray insulin* | 2009 | study design |
| Palermo A. | *Treatment of impaired glucose tolerance with buccal spray insulin: A 6 months randomised controlled trial* | 2011 | study design |
| Palermo A. | *Use of buccal spray insulin for impaired glucose tolerance: The prevoral 2 trial* | 2011 | study design |
| Palermo A. | *Buccal spray insulin: A new tool to treat subjects with impaired glucose tolerance* | 2011 | study design |
| Palermo A. | *Buccal spray insulin in subjects with impaired glucose tolerance: Improvement in HbA1c is lost after 6 months wash out therapy* | 2012 | study design |
| Palermo A. | *Buccal spray insulin in subjects with impaired glucose tolerance: Improvement in HbA1c is lost after 6 months washout therapy* | 2013 | study design |
| Palermo A. | *A new way to treat subjects with impaired glucose tolerance: Buccal spray insulin* | 2011 | study design |
| Pamidi S. | *Effective treatment of obstructive sleep apnea improves glucose tolerance in prediabetes: A randomized placebo-controlled study* | 2013 | study design |
| Pamidi S. | *Optimal CPAP treatment increases insulin sensitivity and decreases sympathetic activity in prediabetics: A randomized placebo-controlled study* | 2014 | study design |
| Pan C. | *Post-marketing surveillance of acarbose treatment in patients with type 2 diabetes mellitus and subjects with impaired glucose tolerance in China* | 2007 | study design |
| Pan X. | *[Effect of dietary and/or exercise intervention on incidence of diabetes in 530 subjects with impaired glucose tolerance from 1986-1992]* | 1995 | population |
| Papakonstantinou E. | Papakonstantinou, E., et al., *Effect of meal frequency on glucose and insulin responses in obese people with impaired glucose tolerance and with type 2 diabetes: A randomised trial* | 2017 | study design |
| Papoz L. | *Effect of oral hypoglycaemic drugs on glucose tolerance and insulin secretion in borderline diabetic patients* | 1978 | population |
| Park S. | *Antihyperglycemic effect of short-term arginyl-fructose supplementation in subjects with prediabetes and newly diagnosed type 2 diabetes: randomized, double-blinded, placebo-controlled trial* | 2015 | population |
| Pedley C. | *Healthy living partnerships to prevent diabetes (help PD): A randomized controlled trialto prevent diabetes through diet and exercise: 2 year effects on the metabolic syndrome* | 2015 | study design |
| Pedley C. | *24 month metabolic benefits of a community-based translation of the diabetes prevention program* | 2013 | study design |
| Pedley C. | *The 24-month metabolic benefits of the healthy living partnerships to prevent diabetes: A community-based translational study* | 2018 | outcomes of interest not reported |
| Pelusi C. | *Clomiphene citrate effect in obese men with low serum testosterone treated with metformin due to dysmetabolic disorders: A randomized, double-blind, placebo-controlled study* | 2017 | population |
| Pengpid S. | *Two-year results of a community-based randomized controlled lifestyle intervention trial to control prehypertension and/or prediabetes in Thailand: a brief report* | 2019 | population |
| Pengpid S. | *Effectiveness of a cluster-randomized controlled trial community-based lifestyle intervention program to control prehypertension and/or prediabetes in Thailand* | 2019 | population |
| Penn L. | *Importance of weight loss maintenance and risk prediction in the prevention of type 2 diabetes: analysis of European Diabetes Prevention Study RCT* | 2013 | study design |
| Perreault L. | *Once-weekly semaglutide 2.4 mg improved glucose metabolism and prediabetes in adults with overweight or obesity in the STEP 1 trial* | 2021 | study design |
| Perreault L. | *Once-weekly semaglutide 2.4 mg improved glucose metabolism and prediabetes in adults with overweight/obesity in the step 1 trial* | 2021 | study design |
| Pi-Sunyer F. | *Liraglutide 3.0 mg reduces the prevalence of prediabetes and delays onset of type 2 diabetes in overweight and obese adults: Results from scale obesity and prediabetes, a randomized, double-blind and placebo-controlled 56-week trial* | 2014 | study design |
| Pi-Sunyer X. | *Liraglutide 3.0 mg reduces the prevalence of prediabetes and delays onset of type 2 diabetes in overweight/obese adults: The SCALE obesity and prediabetes trial* | 2014 | study design |
| Pilmark N. | *The interaction between metformin and physical activity* | 2020 | study design |
| Pilmark N. | *The interaction between metformin and physical activity on postprandial glucose and glucose kinetics: a randomised, clinical trial* | 2021 | population |
| Pittas A. | *Vitamin D and calcium supplementation in adults at risk for type 2 diabetes. The CADDM randomised controlled trial* | 2010 | study design |
| Plat J. | *The effects of long-term almond consumption on whole-body insulin sensitivity, postprandial glucose responses, and free-living glucose patterns in men and women with prediabetes: A randomized controlled trial* | 2023 | study design |
| Polanco M. | *Preventing the development of type 2 diabetes in subjects at high risk in western Mexico, using metformin and changes in lifestyle, followed 10 years* | 2015 | study design |
| Pollack R. | *Resveratrol improves endothelial function and mitochondrial biogenesis in older adults with IGT, without effect on glucose metabolism* | 2015 | study design |
| Pölönen A. | *Dietary changes and their association with clinical risk factors after diabetes prevention intervention in primary health care* | 2019 | study design |
| Pourafshar S. | *Egg consumption may improve factors associated with glycemic control and insulin sensitivity in adults with pre- and type II diabetes* | 2018 | population |
| Pratley R. | *Efficacy and safety of efpeglenatide in key patient subgroups from the BALANCE randomized trial, stratified by pre-diabetes status, BMI, and age at baseline* | 2022 | intervention |
| Punthakee Z. | *Impact of rosiglitazone on body composition, hepatic fat, fatty acids, adipokines and glucose in persons with impaired fasting glucose or impaired glucose tolerance: a sub-study of the DREAM trial* | 2014 | intervention |
| Quinn C. | *Effect of pioglitazone on vascular function in impaired glucose tolerance* | 2009 | study design |
| Quist J. | *Effects of 10-h time-restricted eating on body weight and cardiometabolic health in individuals at high risk of type 2 diabetes (RESET): A randomised controlled trial* | 2022 | study design |
| Raben A. | *The preview intervention study-preliminary results from > 2,000 subjects on body weight and risk factors for type-2 diabetes* | 2017 | study design |
| Rachmani R. | *The effect of acarbose on insulin resistance in obese hypertensive subjects with normal glucose tolerance: a randomized controlled study* | 2004 | population |
| Raghavan A. | *Incidence of Type 2 Diabetes is Higher among Men with Persistent Impaired Glucose Tolerance than in Transient Impaired Glucose Tolerance - A 5 year Follow up Study* | 2018 | outcomes of interest not reported |
| Rahman S. | *Effect of rosiglitazone/ramipril on preclinical vasculopathy in newly diagnosed, untreated diabetes and IGT patients: 1-Year randomised, double-blind, placebo-controlled study* | 2007 | intervention |
| Ram J. | *Improvement in diet habits, independent of physical activity helps to reduce incident diabetes among prediabetic Asian Indian men* | 2014 | outcomes of interest not reported |
| Ramachandran A. | *Efficacy of primary prevention interventions when fasting and postglucose dysglycemia coexist: analysis of the Indian Diabetes Prevention Programmes (IDPP-1 and IDPP-2)* | 2010 | study design |
| Ramachandran A. | *Effectiveness of mobile phone messaging in prevention of type 2 diabetes by lifestyle modification in men in India: a prospective, parallel-group, randomized controlled trial* | 2015 | study design |
| Ramich O. | *Effects of early vs late time-restricted eating on 24-hour glucose profiles and internal circadian time in overweight and obese women* | 2023 | study design |
| Ramírez-Rodríguez A. | *Effect of dapagliflozin on insulin sensitivity and insulin secretion in patients with prediabetes* | 2017 | study design |
| Rana A. | *Effect of Vitamin D supplementation on microalbuminuria in normotensive pre-diabetics with vitamin D deficiency: A randomized controlled trial* | 2018 | study design |
| Ranasinghe P. | *Effects of the Lysulin™ supplementation on pre-diabetes: A randomized double-blind, placebo-controlled clinical trial* | 2020 | intervention |
| Redline S. | *A double-blind cross-over trial of CPAP vs sham-CPAP on metabolic control in individuals with impaired glucose tolerance and sleep apnea* | 2010 | study design |
| Retnakaran R. | *Changes over time in glycemic control, insulin sensitivity and beta-cell function in response to low-dose metformin and thiazolidinedione combination therapy in patients with impaired glucose tolerance* | 2011 | study design |
| Reusch J. | *Rosiglitazone improves c-reactive protein and liver function in impaired glucose tolerance but not coronary artery calcification or exercise function* | 2013 | study design |
| Reynier M. | *Metabolic effects of L-carnitine, L-lysine methionin during maximal exercise* | 1998 | population |
| Rezki A. | *Acute and long-term effects of saxagliptin on post-prandial glycemic response in obese patients with impaired glucose tolerance* | 2021 | overlapping population |
| Rezki A. | *Acute and long-term effects of saxagliptin on post-prandial glycaemic response and cardiovascular parameters in obese patients with impaired glucose tolerance* | 2017 | study design |
| Richelsen B. | *Effect of orlistat on weight regain and cardiovascular risk factors following a very-low-energy diet in abdominally obese patients: a 3-year randomized, placebo-controlled study* | 2007 | population |
| Rissanen A. | *Effect of anti-IL-1beta antibody (canakinumab) on insulin secretion rates in impaired glucose tolerance or type 2 diabetes: results of a randomized, placebo-controlled trial* | 2012 | duplicat |
| Rizos C. | *Co-administration of sartans with different degree of PPARγ activating capacity plus rosuvastatin in patients with multiple metabolic risk factors* | 2009 | study design |
| Rizos C. | *Differential effect of sartans with various PPARγ activating capacity combined with rosuvastatin on glucose metabolism indices* | 2010 | study design |
| Robledo K. | *Mediation analysis of the testosterone treatment effect to prevent type 2 diabetes in the T4DM trial* | 2023 | outcomes of interest not reported |
| Robledo K. | *Mediation analysis of the testosterone treatment effect to prevent type 2 diabetes in the Testosterone for Prevention of Type 2 Diabetes Mellitus trial* | 2023 | duplicat |
| Rodriguez A. | *Dimethylglycine dehydrogenase is rate limiting for betaine metabolism in prediabetes: A randomized clinical trial of betaine supplementation in obese adults* | 2018 | study design |
| Rosenstock J. | *Semaglutide 2.4 mg Improved Glucose Metabolism and Reverted Prediabetes to Normoglycemia in Adults with Overweight/Obesity vs. Liraglutide 3.0 mg (STEP 8)* | 2022 | study design |
| Rossen J. | *Effects of a three-armed randomised controlled trial using self-monitoring of daily steps with and without counselling in prediabetes and type 2 diabetes-the Sophia Step Study* | 2021 | population |
| Rossen J. | *Effects of a three-armed randomised controlled trial using self-monitoring of daily steps with and without counselling in prediabetes and type 2 diabetes—the Sophia Step Study* | 2021 | duplicat |
| Roumen C. | *Impact of 3-year lifestyle intervention on postprandial glucose metabolism: The SLIM study* | 2008 | population |
| Rubino D. | *Semaglutide 2.4 mg improved glucose metabolism and reverted prediabetes to normoglycaemia in adults with overweight or obesity vs liraglutide 3.0 mg (STEP 8)* | 2022 | study design |
| Ruirong P. | *Omega-3 plus plant sterols regulate glucose and lipid metabolism in impaired glucose regulation individuals: A parallel, double-blind controlled and randomized clinical trial* | 2019 | study design |
| Ryan D. | *Effects of low-dose, controlled-release phentermine/topiramate (PHEN/TPM) on weight, glycemic markers, and progression to type 2 diabetes mellitus (T2DM)* | 2010 | study design |
| Ryan D. | *Diabetes prevention and normalization of fasting glucose in subjects with prediabetes using controlled-release phentermine/ topiramate (PHEN/TPM CR) in a 2-year weight loss intervention* | 2011 | study design |
| Sakane N. | *Effects of telephone-delivered lifestyle support on the development of diabetes in subjects at high risk of type 2 diabetes: J-doit1, a pragmatic cluster randomized trial in the primary health care setting* | 2014 | study design |
| Sakane N. | *The impact of lifestyle intervention on stage of change toward healthy eating and development of diabetes in prediabetic individuals in precontemplation stage* | 2018 | study design |
| Sakane N. | *Japan diabetes outcome intervention trial-1 (J-DOIT1): a cluster randomized controlled trial of type 2 diabetes (T2DM) prevention by telephone-delivered lifestyle support for high-risk subjects* | 2013 | study design |
| Sakane N. | *Frequent self-weighing delays or prevents the development of type 2 diabetes in a real-world setting: A subanalysis of Japan diabetes outcome trial-1* | 2017 | study design |
| Salazar-Lopez S. | *Rescue therapy with linagliptin to improve glucose metabolism and pancretic beta cell function in patients with prediabetes with no response to metformin* | 2018 | study design |
| Santilli F. | *Effects of liraglutide vs lifestyle changes on subcutaneous and visceral fat, liver steatosis, insulin sensitivity and beta cell function after comparable weight loss* | 2015 | study design |
| Santilli F. | *Liraglutide is more effective than lifestyle changes in modulating subcutaneous and visceral fat distribution, liver steatosis, insulin sensitivity and beta-cell function after comparable weight loss* | 2015 | study design |
| Santilli F. | *Effects of liraglutide on weight loss, fat distribution, and b-cell function in obese subjects with prediabetes or early type 2 diabetes* | 2017 | population |
| Santilli F. | *Effects of Liraglutide on Weight Loss, Fat Distribution, and β-Cell Function in Obese Subjects With Prediabetes or Early Type 2 Diabetes* | 2017 | duplicat |
| Santilli F. | *Effects of liraglutide or lifestyle counseling on subcutaneous and visceral fat distribution, liver fat content, insulin sensitivity, and β-cell performance* | 2015 | study design |
| Saremi A. | *Pioglitazone slows progression of atherosclerosis in prediabetes independent of changes in cardiovascular risk factors* | 2013 | outcomes of interest not reported |
| Sarich T. | *Canagliflozin, a novel inhibitor of sodium glucose co-transporter 2, increases 24-hour urinary glucose excretion and reduces body weight in obese subjects over 2 weeks of treatment* | 2010 | study design |
| Sasaki J. | *A 52-week, randomized, open-label, parallel-group comparison of the tolerability and effects of pitavastatin and atorvastatin on high-density lipoprotein cholesterol levels and glucose metabolism in Japanese patients with elevated levels of low-density lipoprotein cholesterol and glucose intolerance* | 2008 | population |
| Saslow L. | *Twelve-month outcomes of a randomized trial of a moderate-carbohydrate versus very low-carbohydrate diet in overweight adults with type 2 diabetes mellitus or prediabetes* | 2017 | population |
| Saslow L. | *Comparing Very Low-Carbohydrate vs DASH Diets for Overweight or Obese Adults With Hypertension and Prediabetes or Type 2 Diabetes: a Randomized Trial* | 2023 | population |
| Sathish T. | *Effectiveness of lifestyle intervention in prevention of type 2 diabetes in India: A cluster randomised controlled trial of the Kerala diabetes prevention program* | 2017 | study design |
| Sawada T. | *Effects of 6-month eicosapentaenoic acid treatment on postprandial hyperglycemia, hyperlipidemia, insulin secretion ability, and concomitant endothelial dysfunction among newly-diagnosed impaired glucose metabolism patients with coronary artery disease. An open label, single blinded, prospective randomized controlled trial* | 2016 | population |
| Scheen A. | *[Info-congress. Prevention of type 2 diabetes in obese patients: first results with orlistat in the XENDOS study]* | 2002 | study design |
| Scheen A. | *Prevention of type 2 diabetes in obese subjects: First results with orlistat in the XENDOS study* | 2002 | duplicat |
| Scheen A. | *[DREAM study: prevention of type 2 diabetes with ramipril and/or rosiglitazone in persons with dysglycaemia but no cardiovascular desease]* | 2006 | study design |
| Scheen A. | *[NAVIGATOR: A trial of prevention of cardiovascular complications and type 2 diabetes with valsartan and/or nateglinide]* | 2010 | study design |
| Schuster D. | *Impact of metformin on glucose metabolism in nondiabetic, obese African Americans: A placebo-controlled, 24-month randomized study* | 2004 | study design |
| Schwab U. | *Effect of lifestyle intervention on the risk of incident diabetes in individuals with impaired fasting glucose and low or high genetic risk for the development of type 2 diabetes in men: a T2D-GENE trial* | 2021 | study design |
| Seixas M. | *Exercise and Lifestyle Education program for Brazilians living with prediabetes and diabetes: A pilot randomized trial* | 2022 | population |
| Shiba T. | *Beneficial effect of pitavastatin on the incidence of diabetes in women with impaired glucose tolerance: sub-analysis of j-predict* | 2014 | study design |
| Shiba T. | *Beneficial effect of pitavastatin on the incidence of diabetes in women was not associated with age: Sub-analysis of J-PREDICT* | 2014 | study design |
| Shishkova V. | *An assessment of meldonium therapy effect on glucose, lipid parameters, HBA1C and nt-probnp concentration in post stroke patients with diabetes mellitus and prediabetes* | 2020 | study design |
| Siebel A. | *Effects of the apoA-I inducer RVX-208 on glucose metabolism in individuals with prediabetes* | 2015 | study design |
| Simbauranga R. | *A randomised placebo-controlled double-blind phase II trial to determine the effects of metformin versus placebo on glycaemia in HIV-infected persons with pre-diabetes in Tanzania* | 2022 | study design |
| Singh A. | *Partitioning of radiological, stress and biochemical changes in pre-diabetic women subjected to Diabetic Yoga Protocol* | 2019 | population |
| Sirtori C. | *One-year treatment with ethyl esters of n-3 fatty acids in patients with hypertriglyceridemia and glucose intolerance: reduced triglyceridemia, total cholesterol and increased HDL-C without glycemic alterations* | 1998 | population |
| Sirvent P. | *TOTUM-63, a plant-based polyphenol-rich extract, improves glycaemic control in subjects with prediabetes or early stage newly-diagnosed type 2 diabetes in a randomized, double-blind, placebo-controlled trial* | 2022 | intervention |
| Slentz C. | *Effects of exercise amount and intensity versus lifestyle intervention on glucose tolerance in adults with prediabetes - STRRIDE PD* | 2013 | study design |
| Slomski A. | *Vitamin D Doesn't Protect against Diabetes* | 2019 | study design |
| Sluik D. | *Protein intake and the incidence of pre-diabetes and diabetes in 4 population-based studies: the PREVIEW project* | 2019 | study design |
| Smith R. | *Effects of pioglitazone on metabolic abnormalities, psychopathology, and cognitive function in schizophrenic patients treated with antipsychotic medication: a randomized double-blind study* | 2013 | population |
| Spence J. | *Pioglitazone for secondary stroke prevention in pre-diabetes: Implications for real-world practice* | 2019 | study design |
| Staimez L. | *Cardiovascular risk factors after lifestyle intervention in prediabetes: Findings from the D-CLIP trial* | 2015 | study design |
| Stentz F. | *Efficacy of high protein vs. High carbohydrate diet on remission of impaired glucose tolerance (IGT) to normal glucose tolerance (NGT) and physiological parameters in obese adults* | 2015 | study design |
| Stentz F. | *Remission of prediabetes and improved metabolic parameters with a high-protein diet* | 2016 | study design |
| Stepenka V. | *Metabolic benefits of lifestyle intervention in the clinical setting: A pilot study in latinos with prediabetes from Venezuela, South America* | 2010 | study design |
| Sultana S. | *A comparative study with metformin and pioglitazone versus metformin alone in nonalcoholic fatty liver disease in newly detected glucose intolerant patients* | 2012 | study design |
| Tahara N. | *Pioglitazone decreases asymmetric dimethylarginine levels in patients with impaired glucose tolerance or type 2 diabetes* | 2013 | population |
| Tanaka R. | *Effects of pioglitazone in patients with abnormal glucose metabolism after stroke: the J-spirit study* | 2013 | study design |
| Tay A. | *Profast: A randomized trial assessing the effects of intermittent fasting and lacticaseibacillus rhamnosus probiotic among people with prediabetes* | 2020 | population |
| Temprosa M. | *Long-term effects of metformin on diabetes prevention: Identification of subgroups that benefited most in the diabetes prevention program and diabetes prevention program outcomes study* | 2019 | overlapping population |
| Terauchi Y. | *Differential impact of pitavastatin on the incidence of diabetes in patients with or without hypertension: Sub-analysis of j-predict* | 2014 | study design |
| Thacker H. | *Evaluation Series on Safety and Efficacy of Nutritional Supplements in Newly Diagnosed Hyperglycemia: A Placebo-Controlled, Randomized Study* | 2016 | intervention |
| Toprak O. | *Magnesium Replacement Improves the Metabolic Profile in Obese and Pre-Diabetic Patients with Mild-to-Moderate Chronic Kidney Disease: A 3-Month, Randomised, Double-Blind, Placebo-Controlled Study* | 2017 | duplicat |
| Torabian-Riasati S. | *The effect of nut consumption on fasting plasma glucose levels in pre-diabetic and diabetic adults* | 2010 | study design |
| Trautmann M. | *Exenatide was associated with body weight reduction and normalisation of impaired glucose tolerance in non-diabetic, obese patients* | 2009 | study design |
| Tripathy D. | *Fibroblast Growth Factor-21 (FGF-21) predicts development of diabetes and pioglitazone attenuates the increase in FGF-21 in subjects with IGT: Results from the ACTNOW study* | 2016 | study design |
| Tripathy D. | *Effect of pioglitazone on beta cell function in impaired glucose tolerance: Results form actnow* | 2010 | study design |
| Tripathy D. | *Effect of pioglitazone on beta cell function and adipocyte insulin resistance in impaired glucose tolerance: Results form ACTNOW* | 2010 | study design |
| Tripathy D. | *A novel insulin resistance index to monitor changes in insulin sensitivity and glucose tolerance: the ACT NOW study* | 2015 | study design |
| Tsuchiya M. | *Improvement of plasma glucose at 60 min after the glucose load ameliorates postprandial hyperlipidaemia and LDL-size in patients with IGT or type 2 diabetes: Comparing miglitol and voglibose* | 2009 | study design |
| Tsuchiya M. | *Alogliptin ameliorates dyslipidemia and LDL-size in patients with IGT or type 2 diabetes: Comparing alogliptin and voglibose* | 2011 | study design |
| Tuomilehto H. | *Sleep duration, lifestyle intervention and incidence of type 2 diabetes in impaired glucose tolerance: The finnish diabetes prevention study* | 2009 | overlapping population |
| Tuomilehto H. | S*leep duration, lifestyle intervention, and incidence of type 2 diabetes in impaired glucose tolerance: The Finnish Diabetes Prevention Study* | 2009 | duplicat |
| #VALUE! | *The randomized trial about the efficacy of a 2-Day in-Hospital Diabetes Educational Program for prevention of type 2 diabetes (The JOETSU Diabetes Prevention Trial)* | 2007 | study design |
| Umpierrez G. | *Primary prevention of type 2 diabetes by lifestyle intervention in primary care setting* | 2014 | study design |
| Urakaze M. | *Clinical study of astaxanthin on glucose tolerance in nondiabetic subjects* | 2018 | study design |
| Uusitupa M. | *Long-term improvement in insulin sensitivity by changing lifestyles of people with impaired glucose tolerance: 4-year results from the Finnish Diabetes Prevention Study* | 2003 | overlapping population |
| Uusitupa M. | *The Finnish Diabetes Prevention Study* | 2000 | overlapping population |
| Vadini F. | *Liraglutide improves memory in obese patients with prediabetes or early type 2 diabetes: a randomized, controlled study* | 2020 | population |
| Van Der Zijl N. | *The effect of valsartan on beta-cell function and insulin sensitivity in normotensive subjects with impaired glucose metabolism* | 2010 | study design |
| Van Der Zijl N. | *Valsartan improves beta cell function and insulin sensitivity in normotensive subjects with impaired fasting glucose and/or impaired glucose tolerance* | 2010 | study design |
| Van Der Zijl N. | *Valsartan-induced improvement in insulin sensitivity is not paralleled by changes in microvascular function in individuals with impaired glucose metabolism* | 2011 | overlapping population |
| Van Der Zijl N. | *Valsartan-induced improvement of insulin sensitivity is not mediated by changes in microvascular function in individuals with impaired glucose metabolism* | 2011 | study design |
| Van Gaal L. | *Liraglutide 3.0 mg reduced the risk of developing type 2 diabetes and decreased body weight in the SCALE Obesity and Prediabetes randomised, double-blind, placebocontrolled trial* | 2016 | study design |
| Van Gaal L. | *Phentermine/topiramate combination therapy significantly improves glucose impairment in overweight/obese patients* | 2010 | study design |
| Van Poppel P. | *The interleukin-1 receptor antagonist anakinra improves beta cell function in subjects with impaired glucose tolerance* | 2013 | study design |
| Vettor R. | *Reduction in the risk of developing type 2 diabetes with liraglutide 3.0 mg in people with prediabetes from the SCALE Obesity and Prediabetes randomized, double-blind, placebo-controlled trial* | 2016 | study design |
| Vitolins M. | *The healthy living partnerships to prevent diabetes and the diabetes prevention program: a comparison of year 1 and 2 intervention results* | 2017 | study design |
| Wagner H. | *No effect of high-dose vitamin D treatment on beta cell function, insulin sensitivity or glucose homeostasis in subjects with abnormal glucose tolerance: A RCT* | 2016 | study design |
| Wagner H. | *No Effect of High-Dose Vitamin D Treatment on β-Cell Function, Insulin Sensitivity, or Glucose Homeostasis in Subjects With Abnormal Glucose Tolerance: A Randomized Clinical Trial* | 2016 | population |
| Wagner H. | *No effect of high-dose Vitamin D treatment on beta-cell function, insulin sensitivity, or glucose homeostasis in subjects with abnormal glucose tolerance: a randomized clinical trial* | 2016 | duplicat |
| Wallace H. | *No effect of high dose vitamin D supplementation on insulin resistance and cardiovascular risk factors in patients with pre-diabetes: A double-blind randomised placebo controlled trial* | 2017 | study design |
| Wallace H. | *The effect of vitamin D supplementation on insulin resistance in a pre-diabetic population: A double-blind randomised placebo controlled trial* | 2016 | study design |
| Wang Z. | *Effect of low-intensity ergometer aerobic training on glucose tolerance in severely impaired nondiabetic stroke patients* | 2014 | population |
| Warrilow A. | *Metformin use in prediabetes: is earlier intervention better?* | 2020 | study design |
| Wascher T. | *Reduction of postchallenge hyperglycaemia prevents acute endothelial dysfunction in subjects with impaired glucose tolerance* | 2005 | population |
| Watanabe M. | *Short-term effectiveness of an individual counseling program for impaired fasting glucose and mild type 2 diabetes in Japan: a multi-center randomized control trial* | 2007 | population |
| Weber M. | *The Stepwise Approach to Diabetes Prevention: Results From the D-CLIP Randomized Controlled Trial* | 2016 | population |
| Weinhold K. | *Worksite diabetes prevention trial improves outcomes among employees with prediabetes* | 2015 | study design |
| Wenclewska S. | *Vitamin D Supplementation Reduces Both Oxidative DNA Damage and Insulin Resistance in the Elderly with Metabolic Disorders* | 2019 | population |
| Wennehorst K. | *A Comprehensive Lifestyle Intervention to Prevent Type 2 Diabetes and Cardiovascular Diseases: the German CHIP Trial* | 2016 | population |
| Werner T. | *Magnesium citrate in untreated prediabetics and type ii diabetics-a 12 weeks placebo-controlled DB trial* | 2021 | study design |
| Werner T. | *Magnesium citrate supplementation in untreated prediabetics and type II diabetics without hypomagnesemia - a 12-weeks placebo-controlled double-blinded pilot trial* | 2019 | study design |
| Wilding et al. | *Efficacy and safety of liraglutide 3.0 mg for weight management in overweight and obese adults: the scale obesity and prediabetes, a randomised, doubleblind and placebo-controlled trial* | 2014 | study design |
| Wilding J. | *In overweight and obese adults, liraglutide 3.0mg reduces the prevalence of prediabetes and delays the onset of Type 2 diabetes: SCALE obesity and pre-diabetes trial* | 2015 | study design |
| Wilding J. | *Liraglutide 3.0 mg improves insulin secretion and action in overweight and obese adults without diabetes: Results from scale obesity and prediabetes, a randomized, double-blind and placebo-controlled 56-week trial* | 2014 | study design |
| Wilding J. | *Exposure-response analyses of liraglutide 3.0 mg for weight management* | 2016 | study design |
| Wilding J. | *Early weight loss responders to liraglutide 3.0 mg in the scale obesity and prediabetes trial achieve greater reversal of prediabetes than placebo* | 2015 | study design |
| Willis L. | *Effects of exercise amount and intensity versus a lifestyle intervention on fasting glucose in adults with pre-diabetes - STRRIDE-PD* | 2013 | study design |
| Wittert G. | *Testosterone treatment to prevent or revert type 2 diabetes in men enrolled in a lifestyle programme (T4DM): a randomised, double-blind, placebo-controlled, 2-year, phase 3b trial* | 2021 | population |
| Wittert G. | *Liraglutide 3.0mg improves body weight and cardiometabolic risk factors in overweight or obese adults without diabetes: the SCALE Obesity and Prediabetes randomised, double-blind, placebo-controlled 56-week trial* | 2014 | study design |
| Wong C. | *Effectiveness and cost-effectiveness of a two-year short messaging service (SMS) intervention for professional drivers with pre-diabetes* | 2017 | study design |
| Xiao L. | *Two-year weight-loss maintenance in primary care-based Diabetes Prevention Program lifestyle interventions* | 2013 | population |
| Xu D. | *Effects of lifestyle intervention and meal replacement on glycaemic and body-weight control in Chinese subjects with impaired glucose regulation: a 1-year randomised controlled trial* | 2013 | study design |
| Yalamanchi S. | *Fsigtt-based insulin sensitivity and secretion are not improved by high-dose Vitamin D2 supplementation in african-american male veterans with pre diabetes with hypovitaminosis D* | 2014 | study design |
| Yan H. | *Berberine vs. Pioglitazone for treatment of nonalcoholic fatty liver disease and its associated impaired glucose metabolism* | 2014 | study design |
| Yan H. | *Pioglitazone versus berberine for treatment of non-alcoholic fatty liver disease patients with impaired glucose regulation or type 2 diabetes mellitus* | 2012 | study design |
| Yan H. | *Berberine increases serum osteocalcin levels and improves early phase of insulin secretion in non-alcoholic fatty liver disease patients with impaired glucose metabolism* | 2014 | study design |
| Yang L. | Yang, L., et al., *Role of Purified Anthocyanins in Improving Cardiometabolic Risk Factors in Chinese Men and Women with Prediabetes or Early Untreated Diabetes-A Randomized Controlled Trial* | 2017 | population |
| Yang L. | *Effect of Anthocyanins Supplementation on Serum IGFBP-4 Fragments and Glycemic Control in Patients with Fasting Hyperglycemia: A Randomized Controlled Trial* | 2020 | population |
| Yang L. | *Anthocyanins regulate serum adipsin and visfatin in patients with prediabetes or newly diagnosed diabetes: a randomized controlled trial* | 2021 | population |
| Yang R. | *Intensive Blood Pressure Lowering and the Risk of New-Onset Diabetes in Patients with Hypertension-A Post Hoc Analysis of the STEP Randomized Trial* | 2022 | study design |
| Yates T. | *Twelve-month follow-up from the PREPARE (Prediabetes Risk Education and Physical Activity Recommendation and Encouragement) programme study: A randomized controlled trial* | 2009 | study design |
| Yates T. | *The effect of increased ambulatory activity on markers of chronic low-grade inflammation: evidence from the PREPARE programme randomized controlled trial* | 2010 | overlapping population |
| Yates T. | *Long-term outcomes from the PREPARE (Pre-diabetes Risk Education and Physical Activity Recommendation and Encouragement) randomised controlled trial* | 2010 | study design |
| Yates T. | *The Pre-diabetes Risk Education and Physical Activity Recommendation and Encouragement (PREPARE) programme study: are improvements in glucose regulation sustained at 2 years?* | 2011 | overlapping population |
| Ye Y. | *Effects of daidzein and genistein on glycaemic control and lipid metabolism in Chinese women with prediabetes or untreated early diabetes: A 6-mo double-blind, randomised, placebo-controlled trial* | 2011 | study design |
| Yuan H. | *Amlodipine plus telmisartan or amiloride for hypertension in moderate and high-risk patients: Focus on effects on metabolic profiles* | 2011 | study design |
| Yuan H. | *Amlodipine plus telmisartan or amiloride for hypertensive patients: Focus on effects on metabolic profiles* | 2011 | study design |
| Yuan X. | *Two years supervised resistance training prevented diabetes incidence in people with prediabetes-a randomized control trial* | 2018 | study design |
| Yuen K. | S*hort-term low-dose growth hormone administration in subjects with impaired glucose tolerance and the metabolic syndrome: Effects on β -cell function and post-load glucose tolerance* | 2004 | duplicat |
| #VALUE! | *More effective glycaemic control by metformin in African Americans than in Whites in the prediabetic population* | 2015 | outcomes of interest not reported |
| Zhang L. | *Efficacy of metformin in preventing progression to diabetes in Chinese subjects with impaired glucose regulation: Results from a multicentre, open-label RCT* | 2022 | study design |
| Zhang X. | *Red Raspberry and Fructo-Oligosaccharide Supplementation, Metabolic Biomarkers, and the Gut Microbiota in Adults with Prediabetes: A Randomized Crossover Clinical Trial* | 2022 | population |
| Zhong Q. | *The 18-month efficacy of an Intensive LifeStyle Modification Program (ILSM) to reduce type 2 diabetes risk among rural women: a cluster randomized controlled trial* | 2023 | population |
| Zhu J. | *Intervention effect of diabetic high risk population based on self-efficacy theory in Shanghai.* | 2014 | outcomes of interest not reported |
| Zidek W. | *Ramipril-based versus diuretic-based antihypertensive primary treatment in patients with pre-diabetes (ADaPT) study* | 2012 | study design |
| Zinman B. | *Preventing type 2 diabetes using combination therapy: design and methods of the CAnadian Normoglycaemia Outcomes Evaluation (CANOE) trial* | 2006 | study design |
| Zinman B. | *Low-dose combination therapy with rosiglitazone and metformin to prevent type 2 diabetes mellitus (CANOE trial): a double-blind randomised controlled study* | 2010 | intervention |
| Zinman B. | *Low-dose combination therapy with rosiglitazone and metformin to prevent type 2 diabetes mellitus (CANOE Trial): A double-blind randomized controlled study* | 2010 | duplicat |
| Zou X. | *An efficacy evaluation on prediabetes management using a machine learning-based risk stratification approach* | 2019 | study design |
| Zwickey H. | *Effect of the Anti-Inflammatory Diet in People with Diabetes and Pre-Diabetes: A Randomized Controlled Feeding Study* | 2019 | population |

| Afsharmanesh MR. | Okra (Abelmoschus esculentus) Intake Improves Lipid Profile and Liver Transaminases in Pre-diabetic Adults: A Randomized Double-blinded Trial | 2024 | outcomes of interest not reported |
| --- | --- | --- | --- |
| Basiri R. | Personalized Nutrition Therapy without Weight Loss Counseling Produces Weight Loss in Individuals with Prediabetes Who Are Overweight/Obese: A Randomized Controlled Trial | 2024 -1 | outcomes of interest not reported |
| Basiri R. | Enhancing the Impact of Individualized Nutrition Therapy with Real-Time Continuous Glucose Monitoring Feedback in Overweight and Obese Individuals with Prediabetes | 2024-2 | outcomes of interest not reported |
| Bee YM. | Effectiveness of an Incentives-Enhanced Stepped Care Intervention Program in Diabetes Prevention in a Multiethnic Asian Prediabetes Cohort: Results From the Pre-DICTED Randomized Controlled Trial | 2025 | study design |
| Chow E. | A randomised controlled trial of intermittently-scanned continuous glucose monitoring as an adjunct to lifestyle modification in impaired glucose tolerance | 2023 | study design |
| Díaz-Rizzolo DA. | Late eating is associated with poor glucose tolerance, independent of body weight, fat mass, energy intake and diet composition in prediabetes or early onset type 2 diabetes | 2024 | population |
| Gao J. | Effects of ginseng berry saponins from panax ginseng on glucose metabolism of patients with prediabetes: A randomized, double-blinded, placebo-controlled, crossover trial | 2024 | intervention |
| Hariharan R. | Carnosine supplementation improves glucose control in adults with pre-diabetes and type 2 diabetes: A randomised controlled trial | 2024 | population |
| Huang LC. | Effects of daily almond consumption on glycaemia in adults with elevated risk for diabetes: a randomised controlled trial | 2024 | study design |
| Karvela M. | Assessment of the impact of a personalised nutrition intervention in impaired glucose regulation over 26 weeks: a randomised controlled trial | 2024 | study design |
| Khanna D. | Efficacy of a Diabetes Specific Nutritional Supplement (DSNS) on Glycemic Response in Prediabetic Adults: A Two-Armed, Open-Labelled Randomized Controlled Study | 2024 | intervention |
| LeBlanc ES. | The sleep for health study: A randomized clinical trial of the impact of insomnia treatment on glycemia in people with prediabetes | 2025 | study design |
| Mira-Martínez S. | A Multifaceted Digital Intervention for the Prevention of Type 2 Diabetes Mellitus in Primary Care (PREDIABETEXT): Cluster Randomized Trial | 2025 | population |
| Mira-Martínez S. | Describing the initial results of a pragmatic, cluster randomized clinical trial to examine the impact of a multifaceted digital intervention for the prevention of type 2 diabetes mellitus in the primary care setting: intervention design, recruitment strategy and participants' baseline characteri | 2025 | population |
| Naito E. | Effect of Lactobacillus casei strain Shirota-fermented milk on metabolic abnormalities in obese prediabetic Japanese men: a randomised, double-blind, placebo-controlled trial | 2018 | study design |
| Choon Ming Ng. | A digital health-supported Prediabetes Intervention, Management and Evaluation (PRIME) Program improves Healthy Eating Index score: A cluster randomized controlled trial | 2025 | outcomes of interest not reported |
| Peña A. | Cost-Effectiveness Analysis of an Intensive Lifestyle Intervention Versus Usual Care for Latino Youth With Prediabetes | 2025 | population |
| Pretorius L. | Effects of L-Arabinose on Glycemic Responses After the Consumption of Sucrose-Rich Foods in Individuals with Impaired Fasting Glucose: A Randomized Controlled Cross-Over Trial | 2025 | intervention |
| Quist JS. | Effects of 3 months of 10-h per-day time-restricted eating and 3 months of follow-up on bodyweight and cardiometabolic health in Danish individuals at high risk of type 2 diabetes: the RESET single-centre, parallel, superiority, open-label, randomised controlled trial | 2024 | population |
| Shakya P. | Effectiveness of a group-based Diabetes Prevention Education Program (DiPEP) in a population with pre-diabetes: a cluster randomised controlled trial in Nepal | 2023 | population |
| Srivastava K. | Effectiveness of Multifaceted Lifestyle Interventions in Prediabetic Adults: A Field Study in an Urban Slum Population of Pune, India | 2024 | study design |

5. Table S1: Baseline characteristics table and systematic review (excel attached)

| **Publication**  **data** | | **Prediabetes criteria** | | | | | | **Intervention group** | | | | **Comparator/Control group** | | | | **sysy review** |
| --- | --- | --- | --- | --- | --- | --- | --- | --- | --- | --- | --- | --- | --- | --- | --- | --- |
| **First Author** | **Year of publication** | **HbA1c range** | | **FPG range** | | **2hPG range** | | **N** | **Age (years)** | | **Intervention details** | **N** | **Age (years)** | | **Control details** |  |
|  |  | **mmol/mol** | **%** | **mmol/L** | **mg/dL** | **mmol/L** | **mg/dL** |  | **Mean** | **SD** |  |  | **Mean** | **SD** |  |  |
| Ackermann[1] | 2015 |  | 5,7-6,9 |  | 100-125 |  | 140-199 | 257 | 50.8 | 12.2 | ILI | 252 | 51.2 | 12 | standard care |  |
| Admiraal[2] | 2013 | 42-46 |  | 5,7-6,9 |  | 7,8-10,9 |  | 177 | 44.7 | 10.6 | ILI | 158 | 45 | 9.5 | LI/education |  |
| Aekplakorn[3] | 2019 |  |  |  |  |  | 140-200 | 1030 | 50.9 | 6.3 | enhanced education | 873 | 50.8 | 6.5 | education |  |
| AkbariRad[4] | 2023 |  | 5,7-6,4 |  | 100-125 |  | 140-190 | 35 | 44 | 8.66 | probiotics + lifestyle intervention | 35 | 43 | 8.53 | LI/education |  |
| Al Thani[5] | 2019 |  | 5,6-6,4 | <7 |  |  |  | 57 | 45.51 | 8.96 | vitamin D | 75 | 44.89 | 8.88 | placebo |  |
| Al-Hamdan[6] | 2021 |  |  | 5,6-6,9 |  |  |  | 40 | 42.9 | 12.2 | LI/education | 37 | 50.9 | 7.1 | standard care |  |
| Al-Hamdan[6] | 2021 |  |  | 5,6-6,9 |  |  |  | 43 | 43.7 | 8.1 | enhanced education | 37 | 50.9 | 7.1 | standard care |  |
| Al-Hamdan[7] | 2019 |  | 5,7-6,5 |  |  |  |  | 74 | 40.6 | 9.8 | enhanced education | 49 | 40.6 | 12.7 | standard care |  |
| Alfawaz[8] | 2018 |  |  | 5,6-6,9 |  |  |  | 73 | 43.4 | 7.8 | ILI | 85 | 42.3 | 11.2 | LI/education |  |
| Alfawaz[8] | 2018 |  |  | 5,6-6,9 |  |  |  | 59 | 42.6 | 6.9 | Metformin | 85 | 42.3 | 11.2 | LI/education |  |
| Lindblad[9] | 2011 |  |  | 5,6-6 |  |  |  | 136 |  |  | Glimepiride | 138 |  |  | placebo |  |
| Lindstrom[10] | 2003 |  |  |  |  |  |  | 265 | 55 | 7 | ILI | 257 | 55 | 7 | standard care |  |
| Lindstrom[11] | 2013 |  |  |  |  |  |  |  | 265 |  | ILI | 257 |  |  | standard care | post-intervention follow-upLifestyle intervention in people at high risk of type 2 diabetes induces sustaining lifestyle change and results in long-term prevention of progression to type 2 diabetes. |
| Liu L[12] | 2021 |  | 5,7-6,4 |  | 100-125 |  | 140-199 | 43 | 60.35 | 4.29 | physical activity | 43 | 59.94 | 4.4 | control |  |
| Liu L[12] | 2021 |  | 5,7-6,4 |  | 100-125 |  | 140-199 | 42 | 60.12 | 3.97 | physical activity | 43 | 59.94 | 4.4 | control |  |
| Liu X[13] | 2021 |  |  |  |  |  |  | 72 | 68.52 | 7.61 | physical activity | 73 | 69.85 | 6.29 | physical activity |  |
| Liu Y[14] | 2022 |  | 5,7-6,5 | 6,1-7 |  | 7.8-11,1 |  | 1944 | 52.15 | 8.95 | LI/education | 921 | 53.7 | 7.61 | standard care |  |
| Luo X[15] | 2023 |  |  | 6,1-7 |  | 7,8-11,1 |  | 26 | 51 | 9.5 | physical activity | 21 | 50 | 6.5 | control |  |
| Luo X[15] | 2023 |  |  | 6,1-7 |  | 7,8-11,1 |  | 23 | 52 | 8.2 | physical activity | 21 | 50 | 6.5 | control |  |
| Luo Y[16] | 2022 |  |  | 6,1-7 | 110-126 | 7,8-11,1 | 140-200 | 490 | 53* |  | ILI | 480 | 53* |  | LI/education |  |
| Luo Y[16] | 2022 |  |  | 6,1-7 | 110-126 | 7,8-11,1 | 140-200 | 492 | 53* |  | pioglitazone + lifestyle intervention | 480 | 53* |  | LI/education |  |
| Luo Y[16] | 2022 |  |  | 6,1-7 | 110-126 | 7,8-11,1 | 140-200 | 483 | 53* |  | pioglitazone + ILI | 480 | 53* |  | LI/education |  |
| Kim [17] | 2013 |  |  | 5,6-6,9 |  | 7,8-11 |  | 24 | 58 | 7 | Liraglutide | 27 | 58 | 8 | placebo |  |
| Perreault - STEP 1[18] | 2022 |  | 5,7-6,4 | 5,6-6,9 |  |  |  | 593 | 48.5 | 12.5 | Semaglutide | 263 | 49.4 | 12.1 | placebo |  |
| Tao T[19] | 2021 |  | 5,7-6,4 |  | 100-125 |  | 140-199 | 50 | 28.84 | 6.26 | Exenatide | 50 | 26.28 | 6.22 | Metformin |  |
| Tao T[19] | 2021 |  | 5,7-6,4 |  | 100-125 |  | 140-199 | 50 | 26.82 | 6.32 | Exenatide + Metformin | 50 | 26.28 | 6.22 | Metformin |  |
| Faerch, K[20] | 2021 |  | 5,7-6,4 |  |  |  |  | 30 | 61.4 | 8.5 | Dapagliflozin | 30 | 57.2 | 9.9 | control |  |
| Faerch, K[20] | 2021 |  | 5,7-6,4 |  |  |  |  | 30 | 56.7 | 8.4 | Metformin | 30 | 57.2 | 9.9 | control |  |
| Faerch, K[20] | 2021 |  | 5,7-6,4 |  |  |  |  | 30 | 57.8 | 9.9 | physical activity | 30 | 57.2 | 9.9 | control |  |
| Inzucchi S[21] | 2021 |  | 5,7-6,4 |  |  |  |  | 860 |  |  | Dapagliflozin | 888 |  |  | placebo |  |
| Kullmann S[22] | 2022 |  |  |  |  |  |  | 19 | 57.2 | 9.9 | Empagliflozin | 21 | 62.5 | 7.7 | placebo |  |
| Ramirez-Rodriguez A[23] | 2020 |  |  |  |  |  |  | 12 | 51.5 | 6.3 | Dapagliflozin | 12 | 46.7 | 9.8 | placebo |  |
| Braun B [24] | 2013 |  |  |  |  |  | 140-199 | 8 | 45 | 7.5 | Metformin | 8 | 49.8 | 10.9 | placebo |  |
| Braun B [24] | 2013 |  |  |  |  |  | 140-199 | 8 | 45.4 | 8 | physical activity | 8 | 49.8 | 10.9 | placebo |  |
| Braun B [24] | 2013 |  |  |  |  |  | 140-199 | 8 | 49.1 | 6.6 | Metformin + physical activity | 8 | 49.8 | 10.9 | placebo |  |
| Caballero A[25] | 2004 |  |  |  |  |  |  | 29 | 47.7 | 9.8 | Metformin | 26 | 49.3 | 9.6 | placebo |  |
| Gabriel R[26] | 2023 |  |  |  |  |  |  | 203 | 58.1 | 7.5 | Metformin + lifestyle intervention | 195 | 58.5 | 7.5 | LI/education |  |
| Gabriel R[26] | 2023 |  |  |  |  |  |  | 205 | 58.1 | 7.4 | Linagliptin + lifestyle intervention | 195 | 58.5 | 7.5 | LI/education |  |
| Gabriel R[26] | 2023 |  |  |  |  |  |  | 206 | 58.1 | 8 | Metformin + Linagliptin + lifestyle intervention | 195 | 58.5 | 7.5 | LI/education |  |
| Gonzalez-Heredia T[27] | 2017 |  |  |  |  | 7,1-11 |  | 8 | 51.9 | 6.4 | Metformin | 8 | 49.3 | 5.7 | Linagliptin |  |
| Griffin S[28] | 2018 | 36,6-47,5 |  |  |  |  |  | 125 | 69.9 | 7.2 | Metformin | 124 | 70.1 | 6.2 | placebo |  |
| Guardado-Mendoza R[29] | 2020 |  |  |  |  |  | 140-199 | 70 | 48 | 11 | Metformin + lifestyle intervention | 74 | 50 | 9 | Linagliptin + Metformin |  |
| Iqbal Hydrie M[30] | 2012 |  |  |  |  |  | 140-199 | 114 | 43.1 | 10.1 | ILI | 108 | 44.2 | 10.9 | control |  |
| Iqbal Hydrie[30] | 2012 |  |  |  |  |  | 140-199 | 95 | 43.5 | 8.4 | Metformin + ILI | 108 | 44.2 | 10.9 | control |  |
| Knowler W[31] | 2002 |  |  |  | 95-125 |  | 140-199 | 1073 | 50.9 | 10.3 | Metfromin + lifestyle intervention | 1082 | 50.3 | 10.4 | LI/education |  |
| Knowler W[31] | 2002 |  |  |  | 95-125 |  | 140-199 | 1079 | 50.6 | 11.3 | ILI | 1082 | 50.3 | 10.4 | LI/education |  |
| Lehtovirta M[32] | 2001 |  |  |  |  | WHO criteria |  | 20 | 57.3 | 8 | Metformin | 20 | 58.6 | 12 | placebo |  |
| Li C[33] | 1999 |  |  |  |  | WHO criteria |  | 33 | 49 | 1.3 | Metformin | 37 | 50 | 1.1 | placebo |  |
| O'Brien M[34] | 2017 |  | 5,7-6,4 |  | 100-125 |  |  | 33 | 45.5 | 12.3 | ILI | 30 | 44 | 13.6 | standard care |  |
| O'Brien M[34] | 2017 |  | 5,7-6,4 |  | 100-125 |  |  | 29 | 45.8 | 11.7 | Metformin | 30 | 44 | 13.6 | standard care |  |
| Osei E[35] | 2021 |  |  |  |  | 7,8-11 |  | 26 | 70 | 11 | Metformin | 53 | 68 | 11 | placebo |  |
| OseiE[35] | 2021 |  |  |  |  | 7,8-11 |  | 22 | 66 | 11 | Sitagliptin | 53 | 68 | 11 | placebo |  |
| Ramachandran A[36] | 2006 |  |  |  |  | 7,8-11 |  | 133 | 46.1 | 5.7 | LI/education | 136 | 45.2 | 5.7 | control |  |
| Ramachandran A[36] | 2006 |  |  |  |  | 7,8-11 |  | 133 | 45.9 | 5.9 | Metformin | 136 | 45.2 | 5.7 | control |  |
| Ramachandran A[36] | 2006 |  |  |  |  | 7,8-11 |  | 129 | 46.3 | 5.7 | Metformin + lifestyle intervention | 136 | 45.2 | 5.7 | control |  |
| Viskochil R[37] | 2017 |  |  |  |  | IGT |  | 9 | 46.3 | 2.6 | Metformin | 8 | 49.8 | 3.9 | placebo |  |
| Viskochil R[37] | 2017 |  |  |  |  | IGT |  | 9 | 46.2 | 2.6 | physical activity | 8 | 49.8 | 3.9 | placebo |  |
| Viskochil R[37] | 2017 |  |  |  |  | IGT |  | 10 | 49.5 | 1.8 | Metformin + physical activity | 8 | 49.8 | 3.9 | placebo |  |
| Wan Q[38] | 2010 |  |  | 5,6-6,9 |  | 7,8-11 |  | 20 |  |  | Fenofibrate | 25 |  |  | control |  |
| Wan Q[38] | 2010 |  |  | 5,6-6,9 |  | 7,8-11 |  | 24 |  |  | Metformin | 25 |  |  | control |  |
| Wan Q[38] | 2010 |  |  | 5,6-6,9 |  | 7,8-11 |  | 25 |  |  | diet recommendations | 25 |  |  | control |  |
| Wang Z[39] | 2017 |  |  | IFP |  | IGT |  | 6 | 43.83 | 11.4 | Metformin | 5 | 49.57 | 10.7 | control |  |
| Wang Z[39] | 2017 |  |  | IFP |  | IGT |  | 7 | 38.8 | 11.8 | Saxagliptin | 5 | 49.57 | 10.7 | control |  |
| Wang Z[39] | 2017 |  |  | IFP |  | IGT |  | 6 | 46.17 | 17.2 | Saxagliptin | 5 | 49.57 | 10.7 | control |  |
| Zhang L[40] | 2023 |  |  | 6,1-7 |  | 7,8-11 |  | 847 | 53* |  | LI/education | 831 | 53* |  | Metformin |  |
| Zhao X[41] | 2015 |  |  | 6,1-6,9 |  | 7,8-11 |  | 30 | 46 | 5 | Metformin + lifestyle intervention | 30 | 48 | 7 | LI/education |  |
| Zhao X[41] | 2015 |  |  | 6,1-6,9 |  | 7,8-11 |  | 30 | 47 | 6 | Metformin + lifestyle intervention | 30 | 48 | 7 | LI/education |  |
| Zhao X[41] | 2015 |  |  | 6,1-6,9 |  | 7,8-11 |  | 30 | 45 | 5 | Metformin + lifestyle intervention | 30 | 48 | 7 | LI/education |  |
| Badaam K[42] | 2021 |  |  |  | 100-125 |  | 140-199 | 80 | 30.7 | 3.3 | physical activity | 80 | 31 | 3.4 | physical activity |  |
| Malin S[43] | 2019 |  |  |  | 100-126 |  | 140-199 | 14 | 60.4 | 2.3 | physical activity | 12 | 59.9 | 2.2 | physical activity |  |
| RezkAllah S[44] | 2019 |  | 5,7-6,4 |  | 100-125 |  |  | 20 | 31.8 | 5.3 | physical activity | 20 | 35.9 | 5.89 | control |  |
| RezkAllah S[44] | 2019 |  | 5,7-6,4 |  | 100-125 |  |  | 20 | 31 | 5.27 | physical activity | 20 | 35.9 | 5.89 | control |  |
| Rowan C[45] | 2017 |  | 5,7-6,4 |  |  |  |  | 10 | 47.7 | 6.93 | physical activity | 11 | 53.6 | 8.21 | physical activity |  |
| Chen X[46] | 2021 |  | 5,7-6,4 |  | 100-126 |  | 140-200 | 83 | 60.93 | 5.71 | physical activity | 83 | 60.73 | 5.83 | control |  |
| Chen X[46] | 2021 |  | 5,7-6,4 |  | 100-126 |  | 140-200 | 82 | 59.91 | 5.92 | physical activity | 83 | 60.73 | 5.83 | control |  |
| Dai X[47] | 2019 |  | 5,7-6,4 | 5,6-7 |  | 7,8-11,1 |  | 34 |  |  | physical activity | 35 |  |  | control |  |
| Dai X[47] | 2019 |  | 5,7-6,4 | 5,6-7 |  | 7,8-11,1 |  | 31 |  |  | physical activity | 35 |  |  | control |  |
| Dai X[47] | 2019 |  | 5,7-6,4 | 5,6-7 |  | 7,8-11,1 |  | 37 |  |  | physical activity | 35 |  |  | control |  |
| Kargarfard[48] | 2022 |  | 5,7-6,4 |  | 100-126 |  |  | 22 |  |  | physical activity | 20 |  |  | control |  |
| Ma X[49] | 2022 |  |  | 6,1-7 |  | 7,8-11,1 |  | 34 | 59.18 | 3.93 | physical activity | 32 | 59.09 | 5.25 | control |  |
| Ma X[49] | 2022 |  |  | 6,1-7 |  | 7,8-11,1 |  | 32 | 59.81 | 4.54 | physical activity | 32 | 59.09 | 5.25 | control |  |
| Yan J[50] | 2019 | ADA |  | ADA |  | ADA |  | 35 | 64.23 | 5.75 | physical activity | 35 | 60.31 | 7.56 | control |  |
| Yan J[50] | 2019 | ADA |  | ADA |  | ADA |  | 35 | 62.06 | 8.11 | physical activity |  | 60.31 | 7.56 | control |  |
| Yuan X[51] | 2020 |  | 5,7-6,4 |  | 100-125 |  | 140-199 | 83 | 60.93 | 5.71 | physical activity | 83 | 60.73 | 5.83 | control |  |
| Yuan X[51] | 2020 |  | 5,7-6,4 |  | 100-125 |  | 140-199 |  |  |  | physical activity | 83 | 60.73 | 5.83 | control |  |
| Ariel D[52] | 2014 |  |  |  | 100-126 |  | 140-190 | 23 | 58 | 7 | Liraglutide | 27 | 58 | 8 | placebo |  |
| Barclay C[53] | 2008 |  |  |  |  |  |  | 17 | 67.8 |  | diet | 20 | 61 |  | Low GI |  |
| Barengo N[54] | 2019 |  |  |  | 100-126 |  | 140-200 | 136 | 52.3 | 9.1 | ILI (Nutrition + Physical activity) | 122 | 53.7 | 8.9 | control |  |
| Barengo N[54] | 2019 |  |  |  | 100-126 |  | 140-200 | 132 | 52.6 | 8.2 | ILI (Physical activity + Nutrition) | 122 | 53.7 | 8.9 | control |  |
| Ben-Yacov O[55] | 2021 | 100-125 | 5,7-6,5 |  |  |  |  | 112 | 50 | 7 | diet | 113 | 50 | 7 | personalized |  |
| Bennett W[56] | 2023 |  |  |  | 95-126 |  |  | 35 | 57.9 | 7.5 | physical activity | 31 | 61 | 6.9 | physical activity |  |
| Bennett W[56] | 2023 |  |  |  | 95-126 |  |  | 32 | 61 | 7.1 | physical activity | 32 | 59.2 | 7.9 | LI/education |  |
| Chen X[57] | 2020 |  | 5,7-6,4 |  | 100-125 |  | >140 | 57 | 54.9 | 11.9 | diet | 43 | 51.9 | 11.8 | control |  |
| Dodevska M[58] | 2016 |  |  | 6,1-6,9 |  | 7,8-11 |  | 25 | 58.36 | 6.12 | resistant starch | 25 | 56.96 | 6.13 | fiber |  |
| Eriksson J [59] | 1999 |  |  |  |  |  |  | 112 | 54 | 7 | ILI | 100 | 52 | 7 | control |  |
| Errazuriz I[60] | 2017 |  |  | ADA |  | ADA |  | 15 | 62 | 10 | diet | 13 | 60 | 12 | control | Glucose and hormonal concentrations were similar in all groups before, and unchanged after, 12 weeks of intervention |
| Errazuriz I[60] | 2017 |  |  | ADA |  | ADA |  |  |  |  | diet |  |  |  | control |  |
| Feferman L[61] | 2020 |  | 5,7-6,4 |  |  |  |  | 5 | 41.6 | 14.9 | Carrageenan-Free Diet | 8 | 50.4 | 9.9 | control | Average HbA1c for the carrageenan-free diet group declined significantly compared to the carrageenan-diet group |
| Lankinen M[62] | 2011 |  |  | 5,6-6,9 |  | 7,8-11 |  | 37 | 58 | 7 | diet | 35 | 59 | 7 | refined wheat |  |
| Lankinen M[62] | 2011 |  |  | 5,6-6,9 |  | 7,8-11 |  | 34 | 58 | 8 | diet | 35 | 59 | 7 | refined wheat |  |
| Mashayekhi M[63] | 2023 |  | ADA |  | ADA |  | ADA | 44 |  |  | Liraglutide | 22 |  |  | hypocaloric diet |  |
| Mashayekhi M[63] | 2023 |  | ADA |  | ADA |  | ADA | 22 |  |  | Sitagliptin | 22 |  |  | hypocaloric diet |  |
| Oldroyd J[64] | 2001 |  |  |  |  |  |  | 35 |  |  | ILI | 32 |  |  | control |  |
| Palacios O[65] | 2020 |  | 5,7-6,4 |  | 100-125 |  |  |  |  |  | almonds |  |  |  | control | cross-over: Results indicated no effect of almond intake on FPG |
| Pan X[66] | 1997 |  |  |  |  |  |  | 130 | 44.7 | 9.4 | diet recommendations | 133 | 46.5 | 9.3 | control |  |
| Pan X[66] | 1997 |  |  |  |  |  |  | 141 | 44.2 | 8.7 | physical activity recommendations | 133 | 46.5 | 9.3 | control |  |
| Pan X[66] | 1997 |  |  |  |  |  |  | 126 | 44.4 | 9.2 | ILI | 133 | 46.5 | 9.3 | control |  |
| Parker A[67] | 2014 |  | 5,7-6,4 |  | 100-126 |  |  | 43 | 51.12 | 12.4 | ILI | 38 | 49.61 | 15.6 | usual care |  |
| Ramachandran A[68] | 2013 |  |  |  |  |  | ADA | 271 | 45.9 | 4.8 | enhanced education | 266 | 46.1 | 4.6 | LI/education |  |
| Roncero-Ramos I[69] | 2020 |  |  |  |  |  |  |  |  |  | diet |  |  |  | low fat | Long-term consumption of a low-fat diet was associated with a lower risk of T2DM when compared to the Mediterranean diet in the PreDM-IFG/IGT group (HR: 3.20; 95% CI 0.75e13.69 versus HR: 4.70; 95% CI 1.12e19.67, respectively). |
| Shahbazi S[70] | 2018 |  |  |  |  |  |  | 107 | 43.9 | 10.2 | diet | 109 | 43.4 | 9.6 | control |  |
| Shahbazi S[70] | 2018 |  |  |  |  |  |  | 106 | 43.5 | 9.5 | diet | 109 | 43.4 | 9.6 | control |  |
| Skoradal M[71] | 2018 |  | >5,8 |  |  |  |  | 27 | 60 | 6 | ILI | 23 | 62 | 6 | diet recommendations |  |
| Slentz C[72] | 2016 |  |  | 5,28-6,94 |  |  |  | 40 | 61.4 | 7.1 | physical activity | 38 | 60.4 | 7 | physical activity |  |
| Slentz C[72] | 2016 |  |  | 5,28-6,94 |  |  |  | 35 | 56.9 | 7.8 | physical activity | 37 | 57.6 | 8.1 | physical activity |  |
| Swinburn B[73] | 2001 |  |  |  |  |  |  | 66 | 52.5 | 0.8 | diet | 70 | 52 | 0.8 | control |  |
| Turner K[74] | 2015 |  |  |  |  |  |  |  |  |  | diet high in lean red meat with minimal dairy/diet high in dairy primarily with no red meat |  |  |  | control | cross-over: there was no difference in fasting glucose concentrations between diets |
| Utzschneider K[75] | 2020 |  |  |  | 100-115 |  | 140-199 | 17 | 50.3 | 2.6 | diet | 18 | 57.2 | 1.7 | Low GI |  |
| Vuksan V[76] | 2000 |  |  |  |  |  |  |  |  |  | fibre 8-23 g |  |  |  | wheat bran fiber | cross-over: the addition of 8–13 g/day of high-vis cosity glucomannan to a high-carbohydrate/low-saturated fat NCEP Step 2 diet improved metabolic control beyond diet alone in individuals with insulin resistance |
| Wein P[77] | 1999 |  |  |  |  |  |  | 100 | 39.5 |  | enhanced education | 100 | 37.8 |  | education |  |
| Wien M[78] | 2010 |  |  |  | 100-125 |  |  | 32 | 53 | 9 | diet | 33 | 54 | 11 | control |  |
| Zeng H[79] | 2023 |  |  | <7 |  | 7,8-11 |  | 64 | 56.83 | 5.69 | diet | 69 | 56.28 | 6.48 | control |  |
| Stentz F[80] | 2021 |  |  |  | <126 |  | <200 | 12 | 43.1 | 1.3 | diet | 12 | 41.1 | 1.7 | High carb |  |
| Svensson C [81] | 2019 | 43-47 |  | 6,1-6,9 |  | >7,8 |  | 47 | 42.1 | 10.7 | Liraglutide | 50 | 43 | 10.5 | placebo |  |
| Shukla A[82] | 2023 |  | 5,7-6,4 |  |  |  |  | 18 | 60.2 | 14.4 | LI/education + diet recommendations | 21 | 60.4 | 10.3 | education |  |
| Staite E[83] | 2020 | 39-47 |  |  |  |  |  | 98 | 51.7 | 7.6 | enhanced education | 102 | 52.7 | 8.2 | education |  |
| Smith J[84] | 2019 | 42-47 |  | 6,1-6,9 |  |  |  | 157 | 61.29 | 9.86 | enhanced education | 157 | 61.46 | 9.99 | standard care |  |
| Sutton E[85] | 2018 |  |  |  |  |  |  |  |  |  | Early Time-Restricted Feeding |  |  |  | control | cross-over: Although five weeks of eTRF did not improve glucose levels, it dramatically lowered insulin  levels and improved insulin sensitivity |
| Alfawaz H[86] | 2019 |  |  | 5,6-6,9 |  |  |  | 64 | 43.4 | 5.6 | LI/education | 75 | 43.3 | 6.7 | standard care |  |
| Almukhtar H[87] | 2021 |  |  |  |  |  |  | 20 |  |  | Atorvastatin | 15 |  |  | Rosuvastatin |  |
| Alvarez C[88] | 2012 |  |  |  |  |  |  | 12 | 39.2 | 9.5 | physical activity | 13 | 40.1 | 11.4 | control |  |
| Alvarez C[88] | 2012 |  |  |  |  |  |  | 8 | 33.9 | 9.3 | physical activity | 13 | 40.1 | 11.4 | control |  |
| Alvarez C[88] | 2012 |  |  |  |  |  |  | 10 | 43.3 | 8.1 | physical activity | 13 | 40.1 | 11.4 | control |  |
| Amer O[89] | 2020 |  |  |  | 100-125 |  |  | 73 | 43.4 | 7.8 | ILI | 85 | 42.3 | 11.3 | standard care |  |
| Chiasson J[90] | 1996 |  |  | 5,6-7,7 |  | 7,8-11,1 |  | 8 | 56.1 | 2.9 | Acarbose | 10 | 55.4 | 2.9 | placebo | *FPG range untill 7.7 mmol/l |
| Chiasson J[91] | 2002 |  |  | 5,6-7,7 |  | 7,8-11,1 |  | 682 | 54.3 | 7.9 | Acarbose | 686 | 54.6 | 7.9 | placebo |  |
| Chiasson J[92] | 2004 |  |  |  |  |  |  |  |  |  |  |  |  |  |  | explain how excluding patients from Chiasson 1996 with FPG>7 mmol/l would not change the results |
| Araki R[93] | 2017 |  | 5,7-6,4 |  | 100-125 |  |  | 20 | 54.8 | 2.2 | partially-abraded brown rice | 21 | 52.9 | 2.5 | diet |  |
| Aramaki M[94] | 2013 |  |  |  |  |  |  | 13 |  |  | Alogliptin | 12 |  |  | Voglibose |  |
| Arguelles-Tello F[95] | 2022 |  |  |  |  |  |  | 29 |  |  | Mazindol | 37 |  |  | Mazindol+Metformin | Both prediabetes subgroups  showed reductions in mean FPG, which was  sustained up to the reference range by the  6-month visit |
| Ata N[96] | 2022 |  |  | 5,7-6,9 |  |  |  | 70 |  |  | Vitamin D | 70 |  |  | placebo |  |
| Attallah H[97] | 2007 |  |  |  | <125 |  | 140-200 | 15 | 54.7 | 8.5 | Pioglitazone/GH/Pioglitazone + GH | 16 | 55.4 | 9 | placebo | In the GH group only, FPG increased/ However, mean glycohemoglobin levels for all groups did not change significantly during follow-up. |
| Attia J[98] | 2022 |  | 5,7-6,4 |  |  |  |  | 48 | 61.6* |  | Zinc | 50 | 63.6* |  | placebo |  |
| Barthow C [99] | 2022 |  | 5,9-6,6 |  |  |  |  | 76 |  |  | Lactobacillus rhamnosus capsules | 77 |  |  | placebo | 2X2 factorial: o effect was found for HN001  alone, for OBG alone or the combination of HN001 with  OBG for any of the outcomes measured at any time point |
| Barthow C[99] | 2022 |  | 5,9-6,6 |  |  |  |  | 76 |  |  | OBG (oat derived beta-glucan) | 77 |  |  |  | idem |
| Barzilay J[100] | 2011 |  |  |  | 100-125 |  | 140-199 | 769 |  |  | Telmisartan | 792 |  |  | placebo |  |
| Bauman V[101] | 2019 |  | 5,7-6,4 |  |  |  |  | 71 | 55.2 | 11.1 | LI/education | 77 | 53.3 | 9 | control |  |
| Bauman V[101] | 2019 |  | 5,7-6,4 |  |  |  |  | 65 | 54.1 | 9.9 | LI/education | 77 | 53.3 | 9 | control |  |
| Bauman V[101] | 2019 |  | 5,7-6,4 |  |  |  |  | 74 | 53.9 | 11.6 | LI/education | 77 | 53.3 | 9 | control |  |
| Bhatt S[102] | 2020 |  |  |  | 100-126 |  | 140-200 | 61 |  |  | Vitamin D + Calcium carbonate | 60 |  |  | placebo + Calcium carbonate |  |
| Bhopal R[103] | 2014 |  |  |  |  |  |  | 85 | 52.8 | 10.2 | ILI | 86 | 52.2 | 10.3 | LI/education |  |
| Block G[104] | 2015 |  | 5,7-6,4 |  | 100-125 |  |  | 163 | 55 | 8.8 | enhanced education | 176 | 54.9 | 9.1 | usual care |  |
| Bloomer R[105] | 2009 |  |  |  |  |  |  | 14 | 31 | 3 | acetil L-carnitine arginate | 15 | 35 | 3 | placebo |  |
| Bock G[106] | 2010 |  |  |  | 99-125 |  |  | 11 | 55.2 | 2.5 | Sitagliptin | 11 | 54.1 | 2.4 | placebo |  |
| Bone[107] | 2012 |  |  |  | 100-126 |  | 140-199 | 78 | 59 | 5 | Pioglitazone | 78 | 60.2 | 6.2 | placebo |  |
| Bosch J[108] | 2006 |  |  |  | 110-126 |  | 140-200 | 2623 | 54.7 | 10.9 | Ramipril | 2646 | 54.7 | 10.9 | placebo | 2X2 factorial: The incidence of the primary outcome (newly T2D or death) did not differ significantly between the  ramipril group (18.1%) and the placebo group (19.5%) |
| Cai Y[109] | 2023 |  |  | 6,1-7 |  | 7,8-11 |  | 17 | 63.41 | 5.06 | physical activity | 17 | 61.82 | 4.33 | control |  |
| Califf R[110] | 2010 |  |  |  | 95-126 |  | 140-200 | 4631 | 63.7 | 6.8 | Valsartan | 4675 | 63.8 | 6.8 | placebo | 2X2 factorial: The cumulative incidence of diabetes was 33.1% in the valsartan group, as com pared with 36.8% in the placebo group |
| Caviezel F[111] | 1981 |  |  |  |  |  |  | 7 |  |  | Aspirin | 7 |  |  | placebo | Fasting plasma glucose  and insulin levels did not change after 6 and 30 days of treatment with ASA. |
| Cezaretto A[112] | 2017 |  |  |  |  |  |  | 97 |  |  | enhanced education | 86 |  |  | standard care | The interdisciplinary intervention (enhanced ed) induced greater  decreases in fasting plasma glucose than the traditional  one only after 9 months. |
| Chair S[113] | 2022 |  |  |  | 100-125 |  |  | 34 | 34.68 | 4.37 | alternate-day fasting (ADF) | 34 | 34.97 | 6.23 | control | Compared with the control group, the ADF group showed more significant reductions in fasting blood glucose at both T1 (3 weeks) and T2 (3 months follow-up). |
| Chair S[113] | 2022 |  |  |  | 100-125 |  |  | 33 | 36.06 | 7.67 | 16/8 time-restricted fasting | 34 | 34.97 | 6.23 | control | The 16/8 TRF group showed more significant re ductions in fasting blood glucose only at T2 |
| Chatterjee R[114] | 2017 |  | 5,7-6,5 |  |  |  |  | 15 | 56.9 | 11 | KCl | 12 | 52.7 | 8.9 | placebo |  |
| Chauhan S[115] | 2021 |  |  |  |  |  |  |  |  |  | NRT_N0G5IJ |  |  |  | placebo | We observed that treatment with the unhydrolysed source material only did not exhibit any significant effects on glucose uptake. |
| Chen M[116] | 2017 |  |  |  | 100-125 |  |  | 38 | 51.47 | 8.46 | enhanced education | 40 | 50.78 | 8.93 | control |  |
| Cicero A[117] | 2020 |  |  |  | 100-125 |  |  | 40 | 54 | 3 | Phytosomal curcumin + lifestyle intervention | 40 | 53 | 5 | LI/education |  |
| Cole R[118] | 2013 |  |  |  | 100-125 |  |  | 34 | 61.2 | 8.4 | enhanced education | 31 | 55 | 9.9 | education |  |
| Corpelejin E[119] | 2006 |  |  |  |  | 7,8-12,5 |  | 47 | 56 | 0.9 | ILI | 50 | 58.5 | 1 | control |  |
| Costa[120] | 2003 |  |  | 6,1-7 |  |  |  | 16 | 48.9* |  | Atorvastatin | 17 | 55.4* |  | placebo | Atorvastatin treatment did not result in any change in oral glucose tolerance categories or glucose and insulin response in the OGTT when compared with the PCB group |
| Davies M[121] | 2016 |  |  |  |  |  |  | 447 | 63.9 | 7.6 | enhanced education | 433 | 63.9 | 7.9 | standard care |  |
| Pittas A[122] | 2019 |  | 5,7-6,4 |  | 100-125 |  | 140-199 | 1211 | 59.6 | 9.9 | Vitamin D | 1212 | 60.4 | 10 | placebo |  |
| De Bie T[123] | 2023 |  |  | 6,1-6,9 |  | 7,8-11,1 |  | 25 | 63 | 12 | GABA | 26 | 65 | 12 | placebo | Although we obtained some indication that GABA supplementation could reduce the fasting blood glucose concentration and improve the sleep quality of the participants, these results did not remain significant |
| Defronzo R[124] | 2011 |  |  |  | 90-125 |  | 140-199 | 303 | 53 | 0.4 | Pioglitazone | 299 | 51.5 | 0.7 | placebo |  |
| Derosa G[125] | 2006 |  |  |  |  |  |  | 54 | 51 | 5 | Acarbose + Doxazosin + diet | 53 | 50 | 4 | Acarbose + placebo + diet |  |
| Derosa G[126] | 2016 |  |  |  | 100-126 |  |  | 138 | 53.4 | 11.2 | n-3 polyunsaturated fatty acids | 143 | 54.8 | 12.1 | placebo |  |
| Dutta D[127] | 2014 |  |  |  |  |  |  | 68 | 48.37 | 10.5 | Vitamin D + Calcium carbonate | 57 | 47.4 | 11.5 | Calcium carbonate |  |
| Endevelt R[128] | 2015 |  |  |  | 110-125 |  | 140-199 | 111 | 51.9 | 7.6 | LI individual | 112 | 55.4 | 6.9 | LI group |  |
| Eriksson J[129] | 2006 |  |  |  |  |  |  | 17 | 59 | 2 | Glipizide | 17 | 54 | 3 | placebo |  |
| Everett B[130] | 2018 |  | 5,7-6,5 |  | 100-125 |  |  | 1094 | 61* |  | Canakinumab | 1645 | 61* |  | placebo | Treatment over a median period of 3.7 years did not reduce incident diabetes |
| Rissanen A [131] | 2012 |  |  |  |  |  |  | 28 |  |  | Canakinumab | 27 |  |  | placebo | Mean change in FPG favoured canakinumab in the IGT group, but the differences were not statistically significant. |
| Fagerberg B[132] | 1990 |  |  |  |  |  |  |  |  |  | Metoprolol |  |  |  | Atenolol | cross-over: Had similar effects on metabolic  control in hypertensive men and abnormal glucose tolerance |
| Faghihimani E [133] | 2012 |  |  | 5,6-6,9 |  | 7,8-11 |  | 33 | 47.5 | 0.8 | Salsalate | 33 | 48.3 | 0.9 | placebo | At the end of the study, FPG,  HOMA-IR, and insulin levels were significantly different  between salsalate and placebo groups. |
| Goldfine A[134] | 2013 |  |  | 5,6-7 |  | 7,8-11,1 |  | 34 | 60 | 6 | salsalate | 36 | 57 | 9 | placebo | In summary, salsalate therapy was well tolerated, lowered fasting glucose, increased adiponectin and reduced adipose tissue NF-κB activity |
| Fischer H[135] | 2016 |  | 5.7-6.4 |  |  |  |  | 78 | 47.7 | 12.4 | ILI | 79 | 45.2 | 10.6 | LI/education |  |
| Florentin M[136] | 2013 |  |  | 5.6-6.9 | 100-125 |  |  | 20 | 62.8 | 10 | colesevelam + rosuvastatin | 20 | 62.1 | 11.2 | rosuvastatin |  |
| Fritsche A[137] | 2021 |  |  |  |  | 7.8-11 |  | 356 |  |  | ILI | 351 |  |  | LI/education |  |
| Fritsche A[137] | 2021 |  |  |  |  | 7.8-11 |  | 100 |  |  | LI/education | 101 |  |  | control |  |
| Gagnon C[138] | 2011 |  |  | 6.1-6.9 |  | 7.8-11 |  | 22 | 54.8 | 11.7 | ILI | 26 | 58,4 | 10.7 | LI/education |  |
| Gao F[139] | 2017 |  |  |  |  |  |  | 189 |  |  | ILI | 188 |  |  | LI/education |  |
| Garcia A[140] | 2006 |  |  |  |  |  |  |  |  |  | Arabinoxylan Fibre |  |  |  | placebo | cross-over: fasting serum glucose was significantly lower after AX consump tion compared to placebo |
| Gerstein H[141] | 2020 |  |  |  |  | 7.8-11 |  | 3272 | 64.4 | 8.2 | acarbose | 3250 | 64.3 | 8 | placebo |  |
| Gonzalez-Ortiz M[142] | 2007 |  |  | 6.1-6.9 |  | 7.8-11 |  | 9 |  |  | pioglitazone | 9 |  |  | placebo |  |
| Gosselin L[143] | 2019 |  |  |  | 100-125 |  |  |  |  |  | alpha-lipoic acid |  |  |  | cellulose | cross-over: Although no change (p<0.05) in serum glucose was observed, alpha-lipoic acid-supplemented  12 subjects demonstrated reduced fasting serum insulin |
| Gravesteijn E[144] | 2023 |  |  | 6.1-6.9 |  | 7.8-11 |  |  |  |  | almond diet |  |  |  | control | cross-over: Almond consumption signifcantly decreased insulin sensitivity (P=0.002), and increased postprandial glucose  concentrations (P=0.019), as well as fasting insulin concentrations (P=0.003) as compared to the control period. |
| Griadil T[145] | 2023 |  | 5.7-6.4 | 5.6-7 |  | 7.8-11 |  | 37 | 47.6 | 2.3 | step-up approach | 42 | 49.3 | 2.1 | control | Analyzing the indicators of FPG and HbA1c a statistically significant difference was found when comparing FPG and HbA1c. Analyzing the 3-year follow-up of patients with PD,  cases of T2DM were detected in both groups, but there  was no statistically significant difference when comparing the indicators between the respective groups. |
| Grizales A[146] | 2018 |  | 5.7-6.5 |  | >100 |  | 140-199 | 14 | 57 | 8 | betaine | 13 | 61 | 7 | placebo |  |
| Guerrero-Romero F[147] | 2015 |  |  | 5.6-6.9 | 100-125 | 7.8-11 | 140-199 | 59 | 42.5 | 9.5 | Magnesium | 57 | 42.3 | 8 | placebo |  |
| Gulati S[148] | 2023 |  |  |  | 100-125 |  | 140-199 | 32 | 41.3 | 7.1 | diet | 34 | 42.6 | 9.1 | control |  |
| Gunton J[149] | 2005 |  |  |  |  | 7.8-11 |  | 20 |  |  | chromium picolinate | 20 |  |  | placebo |  |
| Handelsman Y[150] | 2009 |  |  |  | 100-125 |  |  | 38 | 55.2 | 10.9 | colesevelam HCl | 24 | 56 | 13 | placebo | Fasting plasma glucose  was reduced by 4.0 mg/dL with colesevelam HCl 3.8 g/day and by 6.1 mg/dL with colesevelam  HCl 4.5 g/day compared with placebo |
| Handelsman Y[150] | 2009 |  |  |  | 100-125 |  |  | 26 | 54.5 | 10.5 | colesevelam HCl | 24 | 56 | 13 | placebo |  |
| Handelsman J[151] | 2010 |  |  |  | 100-125 |  | 140-199 | 108 | 53.3 | 12.3 | colesevelam HCl | 108 | 55.7 | 11.1 | placebo |  |
| Hansen E[152] | 2012 |  |  |  |  | 7.8-11 |  | 9 |  |  |  | 9 |  |  | physical activity |  |
| Hegde S[153] | 2013 |  |  | 5.6-7 | 100-125 | 7.8-11 | 140-200 | 14 | 46.5 | 13 | physical activity | 15 | 44.67 | 9.57 | control |  |
| Heisler M[154] | 2023 |  | 5.7-6.4 |  |  |  |  | 177 | 55.8 | 13 | enhanced education | 178 | 65 | 12.9 | education |  |
| Hellgren M[155] | 2021 |  |  | 6.1-7 |  | 8.9-12.2 |  | 43 |  |  | ILI | 26 |  |  | LI/education | 5 years follow-up of Hellgreen 2016. Insulin resistance markers and incident T2DM were similar among the groups. |
| Hellgreen M[156] | 2016 |  |  | 6.1-7 |  |  |  | 66 | 63 | 9 | ILI | 30 | 63 | 9 | LI/education | A total of 17 individuals developed Type 2 diabetes, 23% were in the CIG (ILI) and 33% in the CAUG (LI/education);  so there was a 32% reduced risk in the intervention group |
| Hernandez-Alonso P[157] | 2014 |  |  |  |  |  |  |  |  |  | pistachio-supplemented diet |  |  |  | control | In the ITT analysis, glucose and insulin circulating levels decreased significantly (P <0.001) in the PD intervention compared with the CD |
| Hesselink A[158] | 2015 |  |  | 6.1-6.9 |  |  |  | 171 | 62.4 | 9.8 | LI/education | 129 | 65.1 | 9.7 | control/usual care |  |
| Holman R[159] | 2017 |  |  |  |  |  | 140-200 | 3272 | 64.4 |  | acarbose | 3250 | 64.3 | 8 | placebo |  |
| Holman R[160] | 2010 |  |  | 5.3-7 | 95-126 |  |  | 4645 | 63.7 | 6.8 | nateglinide | 4661 | 63.8 | 6.9 | placebo | 2X2 design: after adjustment for multiple testing, nateglinide, as compared with placebo, did  not significantly reduce the cumulative incidence of diabetes (36% and 34%, respectively |
| Honsek C[161] | 2018 |  |  |  |  |  |  | 89 | 59 | 10 | insoluble fibre | 91 | 60 | 10 | insoluble fiber |  |
| Hu S[162] | 2022 |  |  |  | 100-125 |  | 140-199 | 19 |  |  | physical activity | 19 |  |  |  |  |
| Hu Z[163] | 2017 |  |  | 6.1-7 | 110-126 | 7.8-11 | 140-200 | 214 | 69.2 | 6.8 | LI/education | 220 | 69.5 | 6.3 | education |  |
| Huang F[164] | 2014 |  |  |  |  | 7.8-11.1 |  | 36 | 55.3 | 7.1 | auricular vagus stimulation | 36 | 52.3 | 8.7 | sham | Compared with sham taVNS, taVNS significantly reduced the two-hour glucose tolerance (F(2) = 5.79, p = 0.004) |
| Huang Y[165] | 2022 |  |  |  |  | 7.8-11 |  | 26 | 62 | 5 | physical activity | 21 | 63.5 | 4.7 | control |  |
| Hustad K[166] | 2021 | >40 | >5.8 | >5.6 |  | >6.5 |  | 39 | 54.5 | 10.2 | salmon fish protein | 35 | 56.7 | 11 | placebo |  |
| Islam M[167] | 2016 |  |  | 5.6-6.9 |  |  |  | 28 | 42.1 | 7 | Zinc | 27 | 45.5 | 10.4 | placebo |  |
| Jorde R[168] | 2016 | 39.9-51.9 | 5-6.9 | 6-6.9 | 108-124 | 7.8-11 | 140-198 | 256 | 62.3 | 8.1 | Vitamin D | 255 | 61.9 | 9.2 | placebo |  |
| Kabisch S[169] | 2019 |  |  |  |  |  | 140-200 | 32 | 61.3 | 9.5 | fibre | 32 | 60.3 | 10.5 | placebo |  |
| Kabisch S[169] | 2019 |  |  |  |  |  | 140-200 | 35 | 58.9 | 9.1 | fibre | 37 | 59.7 | 8.1 | placebo |  |
| Kachuei A[170] | 2016 |  | 5.7-6.4 |  | 100-125 |  | 140-199 | 23 | 52.27 | 5.14 | treatment to eradicate HP infection | 26 | 52.39 | 7.44 | placebo | Results showed that  HP eradication by a 2-week antibiotic medication did not decrease insulin resistance and even increased FPI and insulin resistance  indices |
| Kaku K[171] | 2015 |  |  |  |  | 7.8-11 | 140-200 | 82 | 63.1 | 9.5 | sitagliptin | 83 | 61.9 | 10.6 | placebo |  |
| Kaku K[171] | 2015 |  |  |  |  | 7.8-11 | 140-200 | 77 | 61.9 | 9.3 | sitagliptin | 83 | 61.9 | 10.6 | placebo |  |
| Kanaya A[172] | 2003 |  |  | 6-6.9 | 110-125 |  |  | 1380 | 67 | 7 | conjugated estrogen + medroxyprogesterone acetate | 1383 | 67 | 7 | placebo | In women with coronary disease, hormone ther apy reduced the incidence of diabetes by 35% |
| Karandish M [173] | 2021 |  |  | 6-6.9 |  | 7.8-11 |  | 21 | 36.95 | 7.23 | curcumin | 20 | 34.19 | 7.03 | placebo |  |
| Karandish M[173] | 2021 |  |  | 6-6.9 |  | 7.8-11 |  | 21 | 38.19 | 4.87 | zinc | 20 | 34.19 | 7.03 | placebo |  |
| Karandish M[173] | 2021 |  |  | 6-6.9 |  | 7.8-11 |  | 20 | 34.48 | 6.45 | zinc + curcumin | 20 | 34.19 | 7.03 | placebo |  |
| Karimi Fard M[174] | 2019 |  |  | 6-6.9 |  | 7.8-11 |  | 30 | 56.5 | 6.3 | alendronate | 30 | 55.3 | 4 | placebo | Administration of 70 mg/week alendronate improves fasting plasma glucose, HbA1c and insulin indices in postmenopausal women |
| Kassaian N[175] | 2018 |  |  |  | 100-125 |  | 140-199 | 27 | 52.9 | 6.3 | probiotics | 28 | 52.97 | 5.9 | placebo |  |
| Kassaian N[175] | 2018 |  |  |  | 100-125 |  | 140-199 | 30 | 52.97 | 6.8 | synbiotics (pro + prebiotic) | 28 | 52.97 | 5.9 | placebo |  |
| Katula J[176] | 2022 | 39-46 | 5.7-6.4 |  |  |  |  | 299 | 55.3 | 12.9 | enhanced education | 300 | 55.6 | 12.6 | LI/education |  |
| Katula J[177] | 2013 |  |  |  | 95-125 |  |  | 151 | 57.3 | 10.1 | ILI | 150 | 58.5 | 9 | LI/education |  |
| Katula J[178] | 2011 |  |  |  | 95-125 |  |  | 151 | 57.3 | 10.1 | ILI | 150 | 58.5 | 9 | LI/education |  |
| Kawahara T[179] | 2022 | <48 | <6.5 | <7 | <126 | 7.8-11 | 140-199 | 630 | 61.1 | 8.8 | vitamin D | 626 | 61.4 | 9.1 | placebo |  |
| Kawahara T[180] | 2008 |  | 5.2-6.4 |  | >100 |  | 140-199 | 143 | 52.6 | 8.9 | ILI | 142 | 51.8 | 9.2 | control |  |
| Kawahara T[180] | 2008 |  | 5.2-6.4 |  | >100 |  | 140-199 | 141 | 51.1 | 9.3 | LI/education | 142 | 51.8 | 9.2 | control |  |
| Kawamori R[181] | 2009 |  | <6.5 | <6.9 |  | 7.8-11 |  | 897 | 55.7 | 9.08 | voglibose | 881 | 55.7 | 9.16 | placebo |  |
| Keech A[182] | 2003 |  |  | 6.1-6.9 |  |  |  |  |  |  | parvastatin |  |  |  | placebo |  |
| Keerthi G[183] | 2017 |  |  |  | 100-125 |  |  | 61 |  |  | physical activity | 60 |  |  | control |  |
| Kentrup H[184] | 1999 |  |  |  |  | >6.7 |  | 6 | 15.7 |  | acarbose | 6 | 13.2 |  | placebo | Acarbose has a positive therapeutic eect on glucose tolerance in cystic ®brosis patients, as shown by attenuation of postprandial plasma glucose increase and a signi®cant decrease in insulin secretion response. |
| Khalilzade S[185] | 2015 |  |  |  | 100-126 |  | 140-200 | 27 | 46.8 | 5.5 | bromocriptine | 26 | 49.5 | 5.9 | placebo | The mean body mass index, systolic blood pressure, fasting plasma glucose and glucose of 30 min, 60 min, 120 min of post OGTT, HbA1c, insulin, HOMA‑IR, lipid profile did not change, significantly, in both bromocriptine and placebo‑treated groups after 12 weeks |
| Kocher T[186] | 2019 |  | 5.7-6.5 |  |  |  |  | 51 |  |  | periodontal treatment + an adjunctive systemic antibiotic treatment | 50 |  |  | only periodontal treatment |  |
| Kosaka K[187] | 2005 |  |  |  |  |  | 140-199 | 102 |  |  | LI/education | 356 |  |  | control |  |
| Kratochvilova S [188] | 2016 |  |  |  |  |  |  |  |  |  | telmisartan |  |  |  |  |  |
| Kuchay M[189] | 2015 |  |  |  |  |  |  | 65 | 47.6 | 9.5 | vitamin D | 64 | 48.5 | 11.8 |  |  |
| Kulzer B[190] | 2009 |  |  |  | 100-126 |  | 140-199 | 91 |  |  | LI/education | 91 |  |  | standard care |  |
| Laaksonen D [191] | 2005 |  |  |  |  | 7.8-11 |  | 249 | 55.8 | 7.2 | physical activity | 238 | 55 | 6.9 | control |  |
| Lalla E[192] | 2015 |  | 5.7 |  |  |  |  | 47 |  |  | enhanced education | 47 |  |  | LI/education | In subjects identified with potential diabetes (baseline HbA1c ≥ 6.5%), HbA1c was reduced 1.46 0.28% compared to baseline |
| Le Roux C[193] | 2017 |  | 5.7-6.4 | 5.6-6.9 |  | 7.8-11 |  | 1505 | 47.5 | 11.7 | Liraglutide | 749 | 47.3 | 11.8 | placebo |  |
| Lee C[194] | 2022 |  | 5.7-6.4 |  | 100-125 |  |  | 48 | 61 | 8.8 | telmisartan + rosuvastatin | 51 | 59.3 | 8.8 | amlodipin + rosuvastatin |  |
| Lee J[195] | 2021 |  |  |  |  |  |  | 207 | 52.6 | 7.5 | LI/education | 208 | 53.5 | 7.4 | standard care |  |
| Lin B[196] | 2023 |  |  |  | 100-125 |  |  | 1651 | 67* |  | Standard BP control (BP<140 mmhg) | 1659 | 67* |  | Intensive BP control (BP<120 mmhg) | Our study found that in comparison to the standard BP control for hypertensive patients with IFG, intensive BP control was associated with a small increased risk of new-onset T2D, though it did not reach statistical signifcance |
| Lindahl B[197] | 2009 |  |  |  |  |  |  | 83 | 52.2 | 9 | ILI in wellness center for 1 month | 85 | 53.5 | 8.4 | control | The intervention affected several important cardio-metabolic risk variables beneficially, and reduced the risk for type 2 diabetes, but the effects persisted only as long as the new lifestyle was maintained. |
| Marrero D[198] | 2016 |  | 5.7-6.5 |  |  |  |  | 112 | 51.5 | 11.5 | ILI | 113 | 51.7 | 11 | LI/education |  |
| Martin J[199] | 2012 |  |  |  |  |  |  | 12 | 64.31 |  | Enhanced external counterpulsation (EECP) | 6 | 64 |  | control | Following 35 sessions of EECP, subjects with abnormal glucose tolerance demonstrated marked improvements in fasting indexes of gly cemic control. |
| Mensink M,[200] | 2003 |  |  | <7.8 |  | 7.8-12.5 |  | 55 | 55.6 |  | ILI | 59 | 57.8 |  | control |  |
| Mensink M[200] | 2003 |  |  | <7.8 |  | 7.8-12.5 |  | 47 | 55 |  | ILI | 55 | 58 |  | control |  |
| Meripour M[201] | 2023 |  |  |  |  |  |  | 120 |  |  | ILI | 120 |  |  | standard care |  |
| Misra P[202] | 2021 |  |  |  | 100-126 |  | 140-200 | 58 | 48.1 | 6.7 | Vit D + Calicume carbonate | 58 | 46.1 | 8.1 | placebo |  |
| Mkrtumyan A[203] | 2022 |  |  |  |  |  |  |  |  |  | Subetta therapy |  |  |  | placebo | 2-h plasma glucose in the Subetta group decreased by 2.05 ± 2.11 mmol/L (versus 0.56 ± 2.55 mmol/L in the Placebo group) after 12 weeks. FPG remained almost unchanged. HbA1c tended to decrease. T |
| MohammadniaMotlagh K[204] | 2022 |  |  |  | 100-125 |  |  | 36 | 46.85 | 7.42 | education | 35 | 47.21 | 7.56 | standard care |  |
| Monti L[205] | 2013 |  |  |  |  |  |  |  |  |  | L-arginine |  |  |  | placebo | cross-over: The circulating glucose, proinsulin/insulin ratio and fat mass were decreased in the L-arginine group compared with the placebo biscuit recipient group. |
| Monti L[206] | 2018 |  |  |  |  |  |  | 72 |  |  | L-arginine | 72 |  |  | placebo |  |
| Morey M[207] | 2012 |  |  |  | 100-125 |  |  | 180 | 67.1 | 6.3 | Enhanced education | 122 | 67.7 | 6.2 | standard care |  |
| Moungngern Y[208] | 2018 |  | 5.7-6.4 |  | 100-125 |  |  | 61 | 55.9 | 9.3 | enhanced education | 64 | 53.6 | 9.8 | education |  |
| Muilwijk M[209] | 2021 |  | 6-6.5 |  |  |  |  | 1838 |  |  | ILI | 1846 |  |  | control |  |
| Nahon K[210] | 2018 |  |  | 5.6- 6.9 |  | 7.8 - 11.1 |  | 14 | 45.3 | 1.7 | sitagliptin | 15 | 47.1 | 1.5 |  |  |
| Nanditha A[211] | 2020 |  | 6-6.4 |  |  |  |  | 1031 | 52.1 | 10.3 | enhanced education | 1031 | 52 | 10.2 | standard care |  |
| Napoli, N[212] | 2018 |  |  |  | 100-125 |  |  | 628 | 72.6 | 5.4 | denosumab + Ca + Vit D | 640 | 72.4 | 5 | placebo + Ca + Vit D | DMAb did not affect FSG in postmenopausal osteoporotic women with prediabetes or  diabetes |
| Nguyen S[213] | 2021 |  |  | 6.1-7.1 |  |  |  | 44 | 58.6 | 4.6 | ILI | 49 | 56.7 | 4.4 |  |  |
| Nijpels G[214] | 2008 |  |  |  |  | 7.1-11.1 |  | 60 | 58.5 | 7.9 | Acarbose | 58 | 56.5 | 7 | placebo |  |
| Niroomand M [215] | 2019 |  |  |  | 100-125 |  | 140-200 | 81 | 45 | 14 | Vit D | 81 | 48 | 11 | Placebo |  |
| O'Dea A[216] | 2015 |  |  | 5.6-6.9 |  | 7.8-11 |  | 24 |  |  | ILI | 26 |  |  | standard care |  |
| Obara K [217] | 2009 |  | 5.2-6.4 |  | 110-125 |  |  | 22 | 53.4 | 8.2 | isohumulone | 21 | 52.3 | 6.7 | placebo | After treatment, fasting blood glucose was decreased in the 32 mg and 48 mg groups after 4 weeks, but did not change in the placebo group. HbA1c was also significantly decreased after 4 weeks in the 16 mg group and after 8 weeks in the 32 mg and 48 mg group |
| Obara K[217] | 2009 |  | 5.2-6.4 |  | 110-125 |  |  | 20 | 53.3 | 7.2 | isohumulone | 21 | 52.3 | 6.7 | placebo |  |
| Obara K[217] | 2009 |  | 5.2-6.4 |  | 110-125 |  |  | 21 | 55.7 | 6.8 | isohumulone | 21 | 52.3 | 6.7 | placebo |  |
| Oh M[218] | 2021 |  |  |  |  |  | 140-199 | 20 | 56.4 | 11.6 | Lactobacilus plantarum | 20 | 53.55 | 10.2 | placebo |  |
| Osei K[219] | 2004 |  |  |  | <140 |  | 140-199 | 9 | 43.3 | 8.7 | Glipizide | 9 | 41 | 4.7 | placebo |  |
| Page R[220] | 1992 |  |  |  |  |  |  | 18 | 39 | 11 | ILI | 7 | 40 | 10 | control |  |
| Palermo A[221] | 2011 |  |  |  |  |  | 140-199 | 10 | 54 | 15 | Insulin Spray | 11 | 51 | 13 | Insuline Spary | Treatment with buccal spray insulin is a simple and valuable therapy for reducing postprandial hyperglycaemia in obese subjects with IGT |
| Pamidi S[222] | 2015 |  |  |  | 100-125 |  | 140-199 | 26 | 53.8 | 6.2 | CPAP | 13 | 55.2 | 8.4 | placebo | In patients with prediabetes, 8-hour nightly CPAP treatment for 2 weeks improves glucose metabolism compared with placebo. |
| Pan C[223] | 2003 |  |  |  | <125 |  | 140-200 | 125 | 53.4 | 8.63 | Acarbose | 127 | 55.6 | 8.31 | placebo |  |
| Panigrahi A[224] | 2023 |  | 5.7-6.4 | 5.6-6.9 |  | 7.8-11.1 |  | 17 | 43.5 | 8.9 | Berberine | 17 | 44.8 | 8.6 | placebo |  |
| Papakonstantinou E[225] | 2018 |  |  |  |  |  |  |  |  |  | isocaloric diet in 3 meals |  |  |  | isocaloric diet in 6 meals | cross-over:Our study shows that 6 vs 3 meals a day can perhaps improve and/or stabilize postprandial glucose regulation in prediabetes subjects |
| Penn L[226] | 2009 |  |  |  |  | 7.8-11.1 |  | 51 | 56.8 |  | ILI | 51 | 57.4 |  | standard care |  |
| Perl S[227] | 2010 |  |  |  |  |  |  |  |  |  | telmisartan |  |  |  | losartan | cross-over: Insulin resistance assessed by HOMA improved significantly after telmisartan only, as did glucose tolerance (pb0.01) |
| Peterson C[228] | 2018 |  | 5.7-6.4 |  | 100-125 |  |  | 29 | 54 | 10 | resistant starch | 30 | 55 | 10 | rapidly digestible starch |  |
| Pimentel G[229] | 2010 |  |  |  | 100-125 |  |  | 21 | 51.7 | 14.5 | Diet recomandations | 30 | 59.8 | 9.2 | control |  |
| Pouryousefi E[230] | 2022 |  | 5.7-6.4 |  | 100-125 |  | 140-199 | 22 | 49.64 | 7.93 | oleoylethanolamide | 21 | 49.76 | 8.1 | Placebo | At the end of the study, consumption of  OEA signifcantly reduced BG, insulin, IR, HbA1c, and CRP (P<0.05) |
| Punthakee Z[231] | 2016 |  |  |  |  |  |  |  |  |  | Insuline glargine |  |  |  | standrad care | 2X2 factorial: insulin glargine had neutral effects on health outcomes and salutary effects on metabolic contro |
| Punthakee Z[231] | 2016 |  |  |  |  |  |  |  |  |  | omega 3 fatty acid |  |  |  | placebo | 2X2 factorial: omega-3 fatty acid supplementation had no effect |
| Ranasinghe P[232] | 2018 |  |  |  |  |  |  | 100 | 51.9 | 6.7 | Zinc | 100 | 51.7 | 7.7 | placebo |  |
| Rasekhi H[233] | 2015 |  |  |  | 100-126 |  | 140-200 | 39 | 40.25 | 5.32 | Vit K | 43 | 40.09 | 4.65 | placebo |  |
| Rezki A[234] | 2021 |  |  | <7 |  | 7.8-11.1 |  | 12 | 40 | 10.7 | Saxagliptin | 12 | 49.8 | 14.6 | placebo |  |
| Riddle M[235] | 2013 |  |  |  |  |  |  | 737 | 64 | 8 | Insulin-Glargine | 710 | 63.77 | 7.95 | standard care | 2X2 factorial: In the sub group without diabetes at baseline, there was a small increase of median A1C from 5.7% initially to 6.0% with standard care and to 5.8%with glargine after 5 years of follow-up |
| Rosenstock J [236] | 2012 |  |  |  | 110-125 |  | 140-200 | 77 | 54.6 | 12.5 | Colesevelam | 76 | 56.4 | 10 | placebo | At study end, there was a significant mean reduction in glycated hemoglobin levels and median reduction in fasting plasma glucose levels with colesevelam compared with placebo (P # 0.02 for both). A fasting plasma glucose level , 100 mg/dL was achieved in 44% of colesevelam recipients compared with 23% of placebo recipients (P , 0.05) |
| Roshanravan N [237] | 2015 |  |  |  |  |  |  | 22 | 29.45 | 4.21 | Zinc | 22 | 29.82 | 5.41 | placebo |  |
| Saito T[238] | 2011 |  |  |  | 100-125 |  |  | 211 | 50* |  | enhanced education | 330 | 48* |  | education |  |
| Sakane N [239] | 2011 |  |  | 5.6-7 |  | 7.8-11.1 |  | 146 | 51 | 7 | LI/education | 150 | 51 | 6 | control |  |
| Sakane N[240] | 2015 |  |  |  | 100-125 |  |  | 1240 | 48.9 | 7.8 | enhanced education | 1367 | 38.9 | 7.5 | education |  |
| Sampson M[241] | 2020 |  | 6-6.5 |  | 100-126 |  |  | 424 | 66.5 | 8.5 | LI/education | 178 | 65.3 | 10 | control |  |
| Sampson M[241] | 2020 |  | 6-6.5 |  | 100-126 |  |  | 426 | 66.7 | 9.5 | enhanced education | 178 | 65.3 | 10 | control |  |
| Santos-Lozano J[242] | 2019 | yes |  | 5.6-6.9 | 100-125 | 7.8-11.1 | 140-199 | 84 | 67.9 | 9 | fatty acids -oleic acid | 92 | 67.4 | 9.2 | Placebo | At 2.5 years, 17 patients from intervention group developed diabetes, and 31 in control |
| Sazonov V[243] | 2013 |  |  | 5.6-7 |  |  |  | 354 |  |  | Niacin | 888 |  |  | Placebo |  |
| Serebrovska T[244] | 2019 |  |  | 5.6-6.9 |  | 7.7-11.1 |  | 17 | 67.7 | 7.7 | physical activity | 16 | 67.5 | 8.7 | sham + diet recommendations |  |
| Serebrovska T[244] | 2019 |  |  | 5.6-6.9 |  | 7.7-11.1 |  | 22 | 64.2 | 6.6 | physical activity | 16 | 67.5 | 8.7 | sham + diet recommendations |  |
| Shamizadeh T[244] | 2019 |  |  |  | 100-125 |  |  | 136 | 51.3 | 11.2 | LI/education | 136 | 53.6 | 9.4 | control |  |
| Sheikhbahaie F[245] | 2016 |  | 5.7-6.5 |  | 100-125 |  |  | 20 | 45.9 | 7.32 | Hydroxychloroquine | 19 | 46.79 | 6.35 | placebo | Except for serum level  of insulin that was significantly elevated after treatment by hydroxychloroquine, the changes in other  parameters remained insignificant. |
| Shek N[246] | 2013 |  |  |  |  | 7.8-11.1 |  | 225 | 38.89 | 4.63 | LI/education | 225 | 39.02 | 4.76 | control |  |
| Shetty B[247] | 2023 |  | 5.7-6.4 |  |  |  |  | 15 |  |  | photodynamic therapy+non-surgical periodontal therapy | 15 |  |  | non-surgical periodontal therapy | One session of NSPT with or without PDT reduce periodontal  inflammation but do not influence glycemic levels in prediabetic  patients. |
| Shionoiri H[248] | 1997 |  |  |  |  |  |  | 23 | 60.9 | 9.7 | Doxazosin | 20 | 59.8 | 11.7 | Placebo |  |
| Sollid S[249] | 2014 |  |  | 6-7 |  | 7.7-11.1 |  | 256 | 62.3 | 8.1 | vitamin D3 | 255 | 61.9 | 9.2 | Placebo |  |
| Spence J[250] | 2019 | yes | 5.7-6.4 |  | 100-125 |  |  | 644 | 63.23 | 9.73 | Pioglitazone | 810 | 64.04 | 10.1 | Placebo | 4.8 year follow-up, HR 0.46 95%CI 0.35-0.61 |
| Suleman F[251] | 2022 |  | 5.7-6.4 | 5.6-6.9 |  |  |  | 40 | 41.6 | 7.94 | Delta-tocotrienol | 37 | 42.43 | 10.9 | Placebo |  |
| Sussman J[252] | 2015 |  |  |  | 95-125 |  | 140-199 | 1027 | 50.8 | 8.9 | metformin +lifestyle intervention | 1030 | 50.4 | 8.8 | placebo + LI/education | Risk based reanalysis of Diabetes Prevention Program: Although the lifestyle intervention provided a sixfold greater absolute risk reduction in the highest risk quarter than in the lowest risk quarter, patients in the lowest risk quarter still received substantial benefit (three year absolute risk reduction 4.9% v 28.3% in highest risk quarter; numbers needed to treat of 20.4 and 3.5, respectively). The benefit of metformin, however, was seen almost entirely in patients in the top quarter of risk of diabetes. No benefit was seen in the lowest risk quarter |
| Sussman J[252] | 2015 |  |  |  | 95-125 |  | 140-199 | 1024 | 50.6 | 9.3 | ILI | 1030 | 50.4 | 8.8 | placebo + LI/education |  |
| Suzuki K[253] | 2008 |  |  |  |  |  |  | 6 | 57.6 | 4.9 | Candesartan + Diet recommendations | 6 | 53.1 | 5.1 | diet |  |
| Talaei A[254] | 2010 |  |  |  |  |  |  | 15 | 40.6 |  | Dehydroepiandrosterone(DHEA) | 15 | 41 |  | Placebo | cross-over: Mean change from baseline after first 3 months FPG = 0.5mmol/l (p=0.1). |
| Tenenbaum A[255] | 2004 |  |  | 6.1-6.9 | 110-125 |  |  | 156 | 61.1 | 5.9 | Benzafibrate retard | 147 | 60.7 | 7 | placebo |  |
| Thirunavukkarasu D[256] | 2017 |  |  |  | >100 |  |  | 25 | 52.36 | 0.66 | soy flour | 25 | 51.52 | 0.51 | wheat flour |  |
| Thirunavukkarasu D[256] | 2017 |  |  |  | >100 |  |  | 25 | 51.24 | 0.62 | soy flour | 25 | 51.52 | 0.51 | wheat flour |  |
| Toprak O[257] | 2017 |  |  |  | 100-126 |  |  | 57 | 55.58 | 13.8 | magnesium | 61 | 57.09 | 12.9 | placebo |  |
| Torgerson J[258] | 2004 |  |  |  |  | 7.8-11.1 |  |  |  |  | orlistat + lieftyle intervention |  |  |  | LI/education | After 4 years’ treatment, the cumulative incidence of diabetes was 9.0% with placebo and 6.2% with orlistat, corresponding to a risk reduction of 37.3% (P  0.0032). |
| Toro-Ramos T[259] | 2020 |  | 5.7-6.4 |  |  |  |  | 103 | 55.69 | 13.6 | enhanced education | 99 | 57.54 | 12.5 |  |  |
| Toshimitsu T[260] | 2020 |  | 5.5-6.4 |  | 100-125 |  |  | 62 | 50.6 | 6.9 | Lactobacillus plantarum OLL2712 | 64 | 51.2 | 7.6 | placebo |  |
| Tuomainen T[261] | 2015 |  |  | 5.6-6.9 |  | 7.8-11.1 |  | 24 |  |  | vitamint D3 | 21 |  |  | placebo |  |
| Tuomainen T[261] | 2015 |  |  | 5.6-6.9 |  | 7.8-11.1 |  | 21 |  |  | vitamint D3 | 21 |  |  | placebo |  |
| Tuomilehto J[262] | 2001 |  |  |  |  | 7.8-11 | 140-200 | 265 | 55 | 7 | ILI | 257 | 55 | 7 | standard care |  |
| Urakaze M[263] | 2021 |  | 5.6-6.4 |  | <125 |  |  | 16 |  |  | astaxanthin (antioxidant) | 13 |  |  | placebo |  |
| Uusitupa M[264] | 1992 |  |  |  |  |  |  | 13 |  |  | chromium-rich yeast | 11 |  |  | placebo |  |
| Van Der Zijl N[265] | 2011 |  |  | 6.1-7 |  | 7.8-11.1 |  | 40 | 58 | 1.1 | valsartan | 39 | 57.9 | 1.1 | placebo |  |
| Van Name M[266] | 2016 |  |  | 5.6-6.9 | 100-125 | 7.8-11 | 140-199 | 61 | 43.8 | 10.8 | ILI | 61 | 43 | 9.7 | usual care |  |
| Van Poppel P[267] | 2014 |  | 5.7-6.4 | 5.6-6.9 |  | 7.8-11 |  | 8 |  |  | anakinra | 8 |  |  | placebo | cross-over: Anakinra treatment did not change fasting glucose levels (5.5±0.2 vs. 5.5±0.3 mmol/l) or HbA1c levels compared with placebo (5.6±0.1% vs. 5.7±0.1%, p=0.068) however the insulino genic index derived from the OGTT improved after anakinra compared with placebo. |
| Vitolins M[268] | 2019 |  |  |  | 95-125 |  |  | 41 | 57* |  | ILI | 107 | 58* |  |  |  |
| Vitolins M[268] | 2019 |  |  |  | 95-125 |  |  | 41 | 57* |  | ILI | 107 | 58* |  |  |  |
| Von Walden F[269] | 2017 |  |  | 5.6-6.9 |  | 7.8-11 |  | 55 | 54 | 6.2 | physical activity | 17 | 54 | 6.9 |  |  |
| Von Walden F[269] | 2017 |  |  | 5.6-6.9 |  | 7.8-11 |  | 20 | 56 | 5.6 | physical activity | 17 | 54 | 6.9 |  |  |
| Wallace H[270] | 2019 |  |  | 5.6.-6.9 |  | 7.8-11 |  | 35 | 52.4 | 2 | vitamin D3 | 31 | 54 | 1.7 | placebo |  |
| Wang J[271] | 2019 |  |  |  |  |  |  | 30 | 56.17 | 7.17 | plant sterols | 28 | 56.18 | 4.06 | placebo |  |
| Wang J[271] | 2019 |  |  |  |  |  |  | 35 | 58.94 | 7.21 | omega-3 (EPA/DHA) | 28 | 56.18 | 4.06 | placebo |  |
| Wang J[271] | 2019 |  |  |  |  |  |  | 41 | 55.63 | 7.44 | Plant Sterols+omega 3 | 28 | 56.18 | 4.06 | placebo |  |
| Wani K[272] | 2020 |  |  | 5.6-6.9 |  |  |  | 150 | 43.1 | 9.4 | ILI | 150 | 43.75 | 10.9 | control |  |
| Weinhold K[273] | 2015 |  | 5.7-6.4 |  | 110-125 |  |  | 35 | 51.6 | 9.5 | ILI | 34 | 51 | 8.1 |  |  |
| Wong C[274] | 2013 |  |  | 5.9-6.9 |  | 7.8-11 |  | 54 | 54.1 | 6.1 | enhanced education | 50 | 55.2 | 6.5 | usual care |  |
| Wong C[275] | 2018 |  |  | 5.6-5.9 |  | 7.8-11 |  | 45 |  |  | enhanced education | 41 |  |  | usual care |  |
| Xiao W[276] | 2016 |  |  | 5.6-7 |  | <7.8 |  | 115 | 45.02 | 11.8 | Losartan | 112 | 46.59 | 12.1 | Levamlodipine |  |
| Yan Q[277] | 2023 | yes |  |  |  | 7.8-11 |  |  |  |  | Probiotics |  |  |  | placebo | At 2 years follow-up, 20 patients devloped T2D in intervention group and 13 in control |
| Yang L[278] | 2020 |  | 5.7-6.4 | 5.6-6.9 |  | 7.8-11 |  | 40 |  |  | anthocyanins | 36 |  |  | placebo |  |
| Yates T[279] | 2009 |  |  |  |  |  |  | 29 | 64 | 7 | LI/education | 29 | 65 | 10 | control |  |
| Yates T[279] | 2009 |  |  |  |  |  |  | 29 | 66 | 8 | ILI | 29 | 65 | 10 | control |  |
| Yates T[280] | 2011 |  |  |  |  |  |  | 22 | 64 | 7 | LI/education | 29 | 65 | 10 | control |  |
| Yates T[280] | 2011 |  |  |  |  |  |  | 22 | 66 | 8 | LI/education | 29 | 65 | 10 | control |  |
| Yeh M[281] | 2016 | 39-46 | 5.7-6.4 |  |  |  |  | 30 | 56.8 | 9.5 | ILI | 30 | 60.9 | 12.2 | control |  |
| Yin Z[282] | 2018 |  |  |  |  |  |  | 109 | 51.06 | 7.15 | ILI | 75 | 53.27 | 7.17 | LI/education |  |
| Yoo J[283] | 2018 |  |  |  |  |  | 140-199 | 39 | 49.79 | 8.4 | coenzim Q10 | 34 |  |  | placebo |  |
| Youngwanichsetha S[284] | 2015 |  |  |  | 100-125 |  | 140-199 | 85 | 35.5 | 6.25 | LI/education | 85 | 35.29 | 5.32 | control |  |
| Yuen K[285] | 2004 |  |  |  |  |  |  |  |  |  | Growth hormone H |  |  |  | placebo | cross-over: GH increased did not modify fasting glucose. After oral glucose intake, glucose tolerance improved (P , 0.03), but post-load insulin levels and b-cell function remained unchanged. |
| Zappe D[286] | 2008 |  |  | 5.5-6.9 | 100-125 |  |  | 189 | 50 | 11 | Valsartan | 190 | 48.8 | 11 | HCTZ |  |
| Zappe D[286] | 2008 |  |  | 5.5-6.9 | 100-125 |  |  | 187 | 50.2 | 11 | Valsartan/HCTZ | 190 | 48.8 | 11 | HCTZ |  |
| Zarrin R[287] | 2017 |  |  |  | 100-125 |  |  | 51 | 48.11 | 7.6 | vitamin D | 53 | 48.43 | 7.7 | placebo |  |
| Raben A[288] | 2021 |  |  |  |  |  |  | 555 | 51.6 | 11.5 | ILI (High protein+ Moderate intensity) | 559 | 51.4 | 11.2 | ILI (Moderate protein+Moderate intensity) | 2X2 factorial: Three-year incidence of T2D was much lower than predicted and did not differ between diets, PA or their combination |
| Raben A[288] | 2021 |  |  |  |  |  |  | 556 | 51.8 | 11.7 | ILI (High protein+ High intensity) | 553 | 51.4 | 11.8 | ILI (Moderate protein+High intensity) |  |
| Rajabi-Naeeni M[289] | 2020 |  |  |  | 100-125 |  |  | 42 | 39.78 | 6.88 | omega-3 | 42 | 41.85 | 7.48 | placebo | A signifcant reduction was observed in fasting glucose, in the co-supplementation group compared to the other three groups (P<0.05). |
| Rajabi-Naeeni M[289] | 2020 |  |  |  |  |  |  | 42 | 39.92 | 6.04 | vit D | 42 | 41.85 | 7.48 | placebo |  |
| Rajabi-Naeeni M[289] | 2020 |  |  |  |  |  |  | 42 | 39 | 7.68 | vit D + omega-3 | 42 | 41.85 | 7.48 | placebo |  |
| Rizos C[290] | 2010 |  |  | 5.6-6.9 | 100-125 |  |  | 52 | 60 | 10 | rosuvastatin + telmisartan | 51 | 58 | 12 | rosuvastatin + olmesartan |  |
| Rizos C[290] | 2010 |  |  |  |  |  |  | 48 | 60 | 10 | rosuvastatin + irbesartan | 51 | 58 | 12 | rosuvastatin + olmesartan |  |
| Rodriguez B[291] | 2012 |  | 5.8-7 |  |  |  |  | 50 | 57.56 | 8.12 | 5-ALA high dose | 51 | 60.37 | 6.88 | placebo | Among individuals taking 5-ALA supplements for 12 weeks, 2 hours post-OGTT glucose levels declined significantly compared to  those not taking the supplement ( p = 0.02) |
| Rodriguez B[291] | 2012 |  |  |  |  |  |  | 53 | 57.57 | 8.04 | 5-ALA low dose | 51 | 60.37 | 6.88 | placebo |  |
| Sakane N[292] | 2014 |  |  |  |  |  |  | 145 |  |  | LI/education | 149 |  |  | standard care |  |
| Sakane N[292] | 2014 |  |  |  |  |  |  | 84 | 51.9 | 6.2 | LI/education | 93 | 52 | 5.6 | standard care |  |
| Sakane N[293] | 2021 |  |  | 5.6-6.9 | 100-125 |  |  | 1240 |  |  | ILI | 1367 |  |  | LI/education |  |
| Sakane N[293] | 2021 |  |  |  |  |  |  | 49.50% | 47.8 | 7.6 | ILI | 51.50% | 48 | 7.3 | LI/education |  |
| Sakane N[293] | 2021 |  |  |  |  |  |  | 26.80% | 49.6 | 7.6 | ILI | 24.30% | 49.3 | 7.6 | LI/education |  |
| Sakane N[293] | 2021 |  |  |  |  |  |  | 17.30% | 51.1 | 8.1 | ILI | 18.70% | 51.4 | 7.3 | LI/education |  |
| Basri S[294] | 2022 |  |  |  |  |  |  |  |  |  | enhanced education |  |  |  | control | The two groups were demographically homogeneous before the study and did not show any statisticallysigniﬁcant differences |
| Xu D[295] | 2012 |  |  | 5.6-6.9 |  | 7.8-11 |  | 46 | 60.35 | 9.78 | LI/education | 42 | 56.55 | 8.61 | control |  |
| Zong Y[296] | 2015 |  |  | 6.1-7 |  | 7.8-11 |  | 87 |  |  | LI/education | 84 |  |  | control |  |
| Alshehri, [297] | 2015 |  |  |  | 100-125 |  |  | 25 | 43.6 | 2.4 | scaling and root planing (SRP) | 25 | 44.7 | 3.2 | SRP + oral doxycycline | There was no signifi cant reduction in FBG levels among patients in Groups 1 and 2 after 6 months of SRP. |
| Bui [298] | 2014 |  |  |  |  | 7,8-11 |  | 30 | 56.9 | 5.8 | pre-germinated brown rice | 30 | 56.6 | 5 | control |  |
| Burtscher [299] | 2012 |  |  |  | 100-125 |  | 140-199 | 12 | 57.8 | 6.5 | physical activity | 18 | 57.8 | 7.9 | LI/education | FPG decreased in all groups from baseline  to the second measurement (p < 0.001 |
| Burtscher [299] | 2012 |  |  |  | 100-125 |  | 140-199 | 12 | 54 | 8 | physical activity | 18 | 57.6 | 5.8 | LI/education |  |
| Burtscher [300] | 2009 |  |  |  | 100-125 |  |  | 18 | 59.1 | 7.8 | ILI | 18 | 55.8 | 5.5 | LI/education |  |
| Chuengsamarn[301] | 2012 |  | 5,7-6,4 |  | 100-124 |  | 140-199 | 118 | 56.95 |  | curcumin | 116 | 57.93 |  | placebo |  |
| Davy [302] | 2017 |  |  |  |  |  |  |  |  |  | physical activity maintanace |  |  |  | standard care | Results did not demonstrate a greater reduction in prediabetes prevalence in the SCT condition. |
| de la Luz Alvarez-Canales [303] | 2021 |  |  |  |  |  | 140-199 | 19 | 53 | 3 | Linagliptin + Metformin + lifestyle intervention | 12 | 52 | 2 | Metformin + LI/education |  |
| Eriksson [304] | 1998 |  |  |  |  |  |  | 7 |  |  | physical activity | 7 |  |  | control |  |
| Guess [305] | 2015 |  |  |  |  |  |  |  |  |  | inulin |  |  |  | cellulose | cross-over: There were no significant differences in mean glucose  concentration |
| Joseph [306] | 2017 |  | 5,7-6,4 |  |  |  |  | 30 | 41.33 | 6.63 | non-surgical periodontal therapy | 30 | 41.93 | 5.77 | control | Periodontal therapy improved  periodontal health status and decreased glycosylated  haemoglobin levels, thus reducing the probability  of occurrence of inflammation induced prediabetes in  patients with CHP. |
| Knowler [307] | 2009 |  |  |  |  |  |  | 910 | 55.3 | 11 | ILI | 932 | 54.8 | 10 | placebo | During 10 years follow-up after DPP, incidences in the former placebo and metformin groups fell to equal those in the former lifestyle group, but the cumulative incidence of diabetes remained lowest in the lifestyle group |
| Knowler [307] | 2009 |  |  |  |  |  |  | 924 | 55.5 | 10.1 | Metformin | 932 | 54.8 | 10 | placebo |  |
| König [308] | 2014 |  |  |  | 100-126 |  |  | 28 |  |  | low calorie - low GI diet | 14 |  |  | LI/education |  |
| Rave[309] | 2007 |  |  |  |  |  |  |  |  |  | whole grain dietary product (WG) |  |  |  | nutrient-dense meal replacement product (MR) | Fasting blood glucose and fasting serum insulin concentrations were lower both after diet with WG and with MR |
| Liberopoulos[310] | 2012 |  |  | 5.6-6.9 | 100-125 |  |  | 20 |  |  | rosuvastatin + manidipine | 20 |  |  | rosuvastatin + olmesartan |  |
| McDermott[311] | 2014 |  |  |  |  |  |  | 21 | 47 | 9.7 | yoga | 20 | 47.2 | 9.1 | standard care |  |
| Monti[312] | 2012 |  |  |  |  | 7.8-11.1 | 140-200 | 72 | 57.2 | 11.7 | L-arginine + lifestyle intervention | 72 | 58.2 | 9.4 | placebo + LI/education |  |
| Oldroyd[313] | 2006 |  |  |  |  | 7.8-11 |  | 37 | 58.2 |  | physical activity | 32 | 57.5 |  | control |  |
| Ramachandran[314] | 2009 |  |  |  |  | 7.8-11 |  | 204 | 45.1 | 6.1 | pioglitazone + lifestyle intervention | 203 | 45.5 | 6.3 | LI/education |  |
| Rosenstock[315] | 2007 |  |  |  |  | 7.8-11.1 |  | 90 | 57.1 | 10.7 | vildagliptin | 89 | 59.8 | 11.5 | placebo |  |
| Saloranta[316] | 2002 |  |  |  |  | 7,8-11 |  | 83 |  |  | Neteglinide | 43 |  |  | placebo | Treatment had no significant effect on FPG or on fasting insulin levels. The change from baseline HbA1c was not statistically significant except in the 120-mg group. |
| Saloranta[316] | 2002 |  |  |  |  |  |  | 76 |  |  | Neteglinide | 43 |  |  | placebo |  |
| Saloranta[316] | 2002 |  |  |  |  |  |  | 86 |  |  | Neteglinide | 43 |  |  | placebo |  |
| Stentz[317] | 2016 |  | 5,7-6,4 |  | <126 |  | 140-199 | 43.1 | 1.3 |  | high protein diet | 12 | 41.1 | 1.7 | diet |  |
| Wang [318] | 2013 |  | 5,7-6,4 |  |  |  |  | 29 | 50 | 9 | white ricce | 28 | 55 | 9 | diet |  |
| Weinstock[319] | 2012 |  |  |  |  |  | >140 |  |  |  | CPAP |  |  |  | sham CPAP | This study did not show that IGT normalizes after CPAP in subjects with moderate sleep apnea and obesity |
| Wohl [320] | 2010 |  |  |  |  |  |  |  |  |  | telmisartan |  |  |  | placebo | cross-over: Fasting plasma glucose was lower after telmisartan compared with placebo |
| Wolever[321] | 2002 |  |  |  |  | 7,8-11 |  | 11 | 58.8 |  | high-carbohydrate–high GI | 11 | 55.8 |  | diet |  |
| Wolever [321] | 2002 |  |  |  |  | 7,8-11 |  | 13 | 55.2 |  | high-carbohydrate–low-GI | 11 | 55.8 |  | diet |  |
| Rohling[322] | 2020 |  |  |  | 100-125 |  |  | 96 | 53 | 9 | ILI | 45 | 52 | 8 | LI/education |  |
| Amer[323] | 2024 |  | 5.7-6.4 |  | 100-126 |  | 140-199 | 41 | 42.9 | 8.43 | Obeticholic acid | 41 | 39.61 | 9.61 | placebo | In our study, use of Obeticholic acid daily for 3 months was found to reduce insulin resistance (P<0.001) with improved glycemic parameters (fasting plasma glucose, HOMA-IR, 2h pp and HbA1c). |
| Bai[324] | 2025 |  |  | <7 |  | 7.8-11.1 |  |  |  |  | Dorzagliatin |  |  |  | placebo | Basal arterialized blood glucose levels were similar following dorzagliatin and placebo administration in the IGT (5.2 ± 0.5 vs. 5.1 ± 0.4 mmol/L; P = 0.21). |
| Basiri[325] | 2025 |  | 5.7-6.4 |  | 100-125 |  |  | 11 | 66.18 | 3.25 | 300 g mango daily | 12 | 65.17 | 4.93 | isocaloric granola bar daily | Daily consumption of mango for 24 weeks improved both short- and long-term blood glucose control, reduced insulin resistance, enhanced insulin sensitivity. |
| Cesar[326] | 2025 |  | 5.7-6.4 |  | 100-125 |  | 140-199 | 35 | 54 | 8.5 | metformin plus the citrus flavonoid supplement (250 mg/day) | 36 | 50.1 | 9.4 | metformin+placebo | cross-over: the nutraceutical group demonstrated improved postprandial glucose metabolism, including a 5% reduction in 2-h OGTT glucose |
| Chotiprakornkul[327] | 2025 |  | 5.7-6.4 |  | 100-125 |  |  | 21 | 53.81 | 2.19 | freeze-dried banana blossom powder (FBP) | 21 | 50.67 | 2.77 | placebo | FBP had no significant impact on FBS, HbA1c, lipid profile, or insulin. However, participants in the FBP group showed significant reductions in body weight, BMI, and diastolic blood pressure |
| Crater[328] | 2024 |  | 5.7-6.4 |  |  |  |  | 20 | 53.8 | 5.6 | cannabinoid receptor-1 (CB1R) inverse agonist, INV- 202 | 17 | 55.5 | 7.4 | placebo | There was no significant difference in OGTT 0- to 3-hour area under the curve for INV-202 versus placebo. INV-202 produced a significant mean weight loss of 3.5 kg. |
| Dwibedi[329] | 2025 |  |  | 6.1-6.9 |  |  |  | 44 | 65 | 7 | broccoli sprout extract (BSE). | 45 | 61 | 10 | placebo | BSE did not meet the prespecified primary outcome, and the overall effect in individuals with prediabetes was a 0.2 mmol l−1 reduction in fasting blood glucose (95% confidence interval −0.44 to −0.01; P = 0.04). |
| Guzman[330] | 2025 |  | 5.7-6.4 |  | 100-125 |  |  | 13 | 49 | 4.15 | beef | 11 | 43.3 | 3.72 | poultry | The results indicated no significant differences between beef and poultry for iAUC C-peptide0–180 min/iAUC glucose0–180 min or any of the secondary and exploratory outcomes. |
| Hankosky[331] | 2025 |  |  |  |  |  |  | 247 | 49.3 | 12.2 | Tirzepatide 5 mg | 268 | 47.7 | 11.8 | placebo | Longterm treatment with tirzepatide was associated with a significant reduction in the risk of developing T2D in people with obesity and prediabetes. |
|  |  |  |  |  |  |  |  | 262 | 47.4 | 11.6 | Tirzepatide 10 mg | 268 | 47.7 | 11.8 | placebo |  |
|  |  |  |  |  |  |  |  | 253 | 48.4 | 11.7 | Tirzepatide 15 mg | 268 | 47.7 | 11.8 | placebo |  |
| Jeong[332] | 2024 |  |  |  | 100-125 |  | 140-199 | 49 | 61.33 | 11.54 | Red Ginseng Extract Powder | 49 | 59.22 | 14.22 | placebo | There were significant differences in changes in FBG and 30-, 60-, 90-, and 120-min blood glucose levels on an OGTT, Hb1Ac levels and glucose area under the curve at 12 weeks from baseline between the 2 group. |
| Kamali[333] | 2025 |  |  |  | 125 |  | 140-199 | 32 | 56.81 | 5.86 | 60 g of barley savigh | 32 | 57.56 | 6.02 | control | The mean FBS after 4 weeks was significantly lower in the intervention compared to the control group (p = 0.019). The mean blood sugar 2h post-prandial was significantly lower in the intervention group compared to the control group after 2 (p = 0.006) and 4 weeks (p =0.009). |
| Ma Y[334] | 2025 |  | 5.7-6.4 | 6.1-6.9 |  | 7.8-11 |  | 20 | 55.65 | 9.48 | real-time continuous glucose monitoring (RT-CGM) | 21 | 60.81 | 10.5 | diet | No statistically significant differences were found between baseline and follow-up measurements at one and two years. |
| Mathioudakis[335] | 2025 |  |  |  |  |  |  | 183 | 60 |  | fully automated AI-led DPP | 185 | 57 |  | human-led DPP | Among adults with prediabetes and overweight or obesity, referral to a fully automated AI-led DPP was noninferior to referral to a human-led DPP in achieving a composite outcome based on weight reduction, physical activity, and HbA1c. |
| Mes[336] | 2025 |  |  |  |  |  |  | 15 | 67.5 | 6.6 | freeze-dried bitter gourd (BG) fruit juice (2.4 g/day) | 15 | 68.1 | 4.8 | cucumber-based control product (CC). | cross-over, 4 weeks : no significant differences were found between the bitter gourd and placebo interventions |
| Mes | 2025 |  |  |  |  |  |  | 19 | 62 | 8.1 | freeze-dried whole fruits (3.6 g/day) | 17 | 64.5 | 5.7 | a cucumber-based matched control supplement | parallel, 12 weeks: significant reductions of FPG (p = 0.014), fasting insulin (p = 0.007), and HOMA-IR (p = 0.003) were found after a 12-week intervention with the bitter gourd supplement |
| Mohamed[337] | 2025 |  |  | 5.6-6.9 |  |  |  | 48 | 46.5 | 9.8 | whole fruit feijoa powder | 49 | 46.3 | 10.4 | placebo | Fx supplementation may enhance improvements in glycaemia and blood pressure achieved through. |
| Pickering[338] | 2023 |  |  | 5.5-6.9 |  | 7.8-11 |  | 25 | 58.8 |  | C grandic | 23 | 58.9 |  | placebo | At 12 weeks there was a significant difference between the C. grandis and control groups in the change between their pre and post treatment measurements of FBG and 2hPPBG. |
| Saadat[339] | 2015 |  |  |  |  |  |  | 12 | 56.66 | 2.233 | 0.25-mg cabergoline | 11 | 51.18 | 2.81 | placebo | Our results showed significant reductions in fasting (P = 0.004) and 2-h plasma glucose (P = 0.01) after treatment. |
| Şahin[340] | 2024 |  |  |  | 100-125 |  |  | 15 |  |  | 250 gm. of quinoa T1 | 15 |  |  | control | Significant reduction in HbA1c levels of Treatment 2. However, no significant difference in HbA1c level was observed in Treatment 1. |
| Şahin | 2024 |  |  |  | 100-125 |  |  | 15 |  |  | 250 gm of quinoa with 20gm of cinnamon powder T2 | 15 |  |  | control |  |
| Valder[341] | 2025 |  | 5.7-6.4 |  |  |  |  |  |  |  | red berry fruit juice |  |  |  | placebo | cross-over: Daily consumption of red berry fruit juice led to improvements in peripheral resistance |
| Valder | 2025 |  | 5.7-6.4 |  |  |  |  |  |  |  | red berry fruit juice + strength and endurance training |  |  |  | placebo+strength and endurance training | cross-over: Post-hoc tests revealed a significant decrease in relative muscle mass in the placebo group (p =0.048) while no significant change was observed in the juice group (p =0.205). |
| Zelicha[342] | 2024 |  | 5.7-6.4 |  | 100-125 |  |  |  |  |  | cinnamon (4 g/d) |  |  |  | placebo | cross-over: mean net-area-under-the-curve (netAUC) for glucose was significantly lower than for placebo when cinnamon was given (over 24 h; ES ¼ –0.66; 95 % CI: 2501.7, 5412.1, P ¼ 0.01). |
| Afshari[343] | 2025 |  |  |  |  |  |  | 21 |  |  | enhanced education | 36 |  |  | standard care |  |
| Ahmed[344] | 2026 |  |  |  | 110-125 |  | 140-199 | 1124 | 43.8 | 8.8 | intensive lifestyle intervention | 1041 | 44 | 8.6 | standard care |  |
| Arias-Fernández[345] | 2025 |  |  |  | 100-125 |  |  | 103 | 58.89 | 9.76 | enhanced education | 103 | 59.84 | 11.23 | education |  |
| Baker[346] | 2024 |  | 5.7-6.4 | 5.6-6.9 |  |  |  | 25 |  |  | physical activity | 26 |  |  | control |  |
| Bhowmik[347] | 2025 |  |  | 6.1-6.9 |  | 7.8-11 |  | 396 | 45.6 | 10.9 | intensive lifestyle intervention | 403 | 46.8 | 12.2 | standard care |  |
| Braun[348] | 2024 |  | 5.6-7 |  |  |  |  | 30 | 62.5 |  | Diet | 31 | 58 |  | control |  |
| Brown[349] | 2024 |  | 5.7-6.5 |  | 100-126 |  | 140-200 | 50 | 48.7 | 6.9 | enhanced education | 30 | 51.9 | 7.4 | education |  |
| Cakmak[350] | 2025 |  | 5.7-6.4 |  | 100-125 |  | 140-199 | 33 | 48.3 | 12 | education | 35 | 49 | 12.4 | standard care |  |
| Chen[351] | 2025 |  |  |  |  |  |  | 29 | 51 | 4.42 | physical activity | 36 | 51.69 | 4.61 | physical activity |  |
| Cheng[352] | 2024 |  |  |  |  |  |  | 11 | 49.7 | 9.6 | statin | 19 | 53.6 | 9 | placebo |  |
| Fridén[353] | 2025 |  |  |  |  |  |  | 24 |  |  | Diet | 15 |  |  | control |  |
| Hafeez[354] | 2025 |  | 5.7-6.4 |  | 100-125 |  |  | 36 | 32.25 | 5.69 | intensive lifestyle intervention | 36 | 30.75 | 4.14 | control |  |
| Hall[355] | 2024 |  | 5.8-6.5 |  |  |  |  |  |  |  | Diet |  |  |  | placebo |  |
| Jadhav[356] | 2024 |  | 5.7-6.4 |  | 100-125 |  |  |  |  |  | physical activity |  |  |  | standard care |  |
| Jastreboff[357] | 2025 |  |  |  |  |  |  |  |  |  | GLP1+GIP |  |  |  | placebo |  |
| Karaman[358] | 2025 |  |  |  |  |  |  | 21 | 46.52 | 7.49 | physical activity | 21 | 44.24 | 9.61 | control |  |
| Lamichhane[359] | 2025 |  | 5.7-6.4 |  | 100-125 |  |  | 14 |  |  | curcumin | 9 |  |  | placebo |  |
| Madhu[360] | 2024 |  |  |  | 100-125 |  | 140-199 | 488 | 43.7 | 9.1 | intensive lifestyle intervention | 486 | 44.6 | 9.1 | lifestyle intervention |  |
| Mari[361] | 2025 |  |  |  |  |  |  | 762 | 48.3 | 11.8 | GLP1+GIP | 270 | 47.7 | 11.9 | placebo |  |
| Mashayekhi[362] | 2024 |  |  |  |  |  |  |  |  |  | GLP1 |  |  |  | Diet |  |
| Molani-Gol[363] | 2025 |  | 5.7-6.4 |  | 100-125 |  |  | 48 | 51.5 | 9.75 | Vitamin D | 44 | 50.31 | 8.32 | placebo |  |
| Pasdar[364] | 2025 |  |  |  |  |  |  | 24 | 46.92 | 10.46 | Diet | 25 | 47 | 8.92 | control |  |
| Saboo[365] | 2024 |  | 5.7-6.4 |  | 110-125 |  |  | 125 | 42.5 | 10.45 | intensive lifestyle intervention | 125 | 42.7 | 9.51 | Diet |  |
| Sangeetha[366] | 2023 |  | 5.7-6.4 |  | 100-125 |  | 140-199 | 167 | 53.13 | 6.41 | Thiazolidindione | 167 | 56.11 | 5.36 | Metformin |  |
| Sarabi[367] | 2025 |  | 5.7-6.4 |  | 100-125 |  | 140-199 | 26 |  |  | probiotics | 26 |  |  | placebo |  |
| Sawangwong[368] | 2023 |  | 5.7-6.4 |  | 100-125 |  | 140-199 | 31 | 49.31 | 13.01 | physical activity | 30 | 51.16 | 11.92 | control |  |
| Stice E[369] | 2025 |  | 5.7-6.4 |  |  |  |  | 30 | 50 | 9.8 | intensive lifestyle intervention | 32 | 53.5 | 8.4 | lifestyle intervention |  |
| Sukatta[370] | 2025 |  | 5.7-6.4 |  | 100-125 |  |  | 10 | 48.7 | 11.1 | physical activity | 11 | 44.4 | 6.4 | placebo |  |
| Jariya Hangsantea[371] | 2024 |  |  |  |  |  |  | 124 | 48.55 | 8.4 | enhanced education | 75 | 48.07 | 8.39 | control |  |
| Toshimitsu [372] | 2024 |  | 5.6-6.4 |  |  |  |  | 73 | 50.7 | 9.1 | probiotics | 72 | 50.8 | 8.6 | placebo |  |
| Vargas MC[373] | 2024 |  | 5.7-6.4 |  |  |  |  | 20 | 49.5 | 9.5 | intensive lifestyle intervention | 20 | 50.4 | 9.4 | standard care |  |
| Pi-Sunyer[374] | 2015 | ADA |  |  |  |  |  | 1528 | 47.4 | 11.8 | GLP1 | 757 | 47.2 | 11.8 | placebo |  |
| Kahn[375] | 2024 | ADA |  |  |  |  |  | 5877 | 61.8 | 8.7 | GLP1 | 5819 | 62 | 8.6 | placebo |  |
| McGowan[376] | 2024 |  |  | 6-6.4 |  |  |  | 138 | 53 | 11 | GLP1 | 69 | 53 | 11 | placebo |  |
| Mu[377] | 2024 |  |  |  |  |  |  | 69 |  |  | GLP1 | 38 |  |  | placebo |  |
| Garvery[378] | 2022 |  |  |  |  |  |  | 77 |  |  | GLP1 | 64 |  |  | placebo |  |

6. Figure S1: Network plot of interventions for preventing T2D. Every knot represents a different therapy. The larger the knot, the greater the number of studies reporting on the specific treatment. Every edge compares different therapies. The edge width and the number above indicate how many studies investigated the specific comparison

7. Figure S2: Direct comparissons – incidence of T2D

7. Table S2: Direct and Indirect Comparisons with p-values – incidence of T2D (Netsplit)

| Comparison | k | Prop | NMA | Direct | Indirect | p-value |
| --- | --- | --- | --- | --- | --- | --- |
| alpha glucosidase inhibitor:antihipertensive | 0 | 0 | 0.7254 | . | 0.7254 | . |
| alpha glucosidase inhibitor:control | 5 | 1.00 | 0.7120 | 0.7120 | . | . |
| alpha glucosidase inhibitor:diet | 0 | 0 | 1.2197 | . | 1.2197 | . |
| alpha glucosidase inhibitor:enhanced education | 0 | 0 | 0.8021 | . | 0.8021 | . |
| alpha glucosidase inhibitor:GLP1 | 0 | 0 | 2.7867 | . | 2.7867 | . |
| alpha glucosidase inhibitor:GLP1+GIP | 0 | 0 | 7.2338 | . | 7.2338 | . |
| alpha glucosidase inhibitor:intensive lifestyle intervention | 0 | 0 | 1.3072 | . | 1.3072 | . |
| alpha glucosidase inhibitor:L-arginine | 0 | 0 | 1.2881 | . | 1.2881 | . |
| alpha glucosidase inhibitor:lifestyle intervention/education | 0 | 0 | 0.9922 | . | 0.9922 | . |
| alpha glucosidase inhibitor:lipid low | 0 | 0 | 0.8171 | . | 0.8171 | . |
| alpha glucosidase inhibitor:Metformin | 0 | 0 | 1.1054 | . | 1.1054 | . |
| alpha glucosidase inhibitor:Metformin + intensive lifestyle intervention | 0 | 0 | 2.5946 | . | 2.5946 | . |
| alpha glucosidase inhibitor:physical activity | 0 | 0 | 1.3447 | . | 1.3447 | . |
| alpha glucosidase inhibitor:Sulfonylureas | 0 | 0 | 0.9413 | . | 0.9413 | . |
| alpha glucosidase inhibitor:thiazolidindione | 0 | 0 | 1.3461 | . | 1.3461 | . |
| alpha glucosidase inhibitor:thiazolidindione +intensive lifestyle | 0 | 0 | 1.6792 | . | 1.6792 | . |
| alpha glucosidase inhibitor:Vitamin D | 0 | 0 | 0.7943 | . | 0.7943 | . |
| antihipertensive:control | 1 | 1.00 | 0.9815 | 0.9815 | . | . |
| antihipertensive:diet | 0 | 0 | 1.6814 | . | 1.6814 | . |
| antihipertensive:enhanced education | 0 | 0 | 1.1058 | . | 1.1058 | . |
| antihipertensive:GLP1 | 0 | 0 | 3.8417 | . | 3.8417 | . |
| antihipertensive:GLP1+GIP | 0 | 0 | 9.9723 | . | 9.9723 | . |
| antihipertensive:intensive lifestyle intervention | 0 | 0 | 1.8021 | . | 1.8021 | . |
| antihipertensive:L-arginine | 0 | 0 | 1.7758 | . | 1.7758 | . |
| antihipertensive:lifestyle intervention/education | 0 | 0 | 1.3678 | . | 1.3678 | . |
| antihipertensive:lipid low | 0 | 0 | 1.1265 | . | 1.1265 | . |
| antihipertensive:Metformin | 0 | 0 | 1.5239 | . | 1.5239 | . |
| antihipertensive:Metformin + intensive lifestyle intervention | 0 | 0 | 3.5768 | . | 3.5768 | . |
| antihipertensive:physical activity | 0 | 0 | 1.8538 | . | 1.8538 | . |
| antihipertensive:Sulfonylureas | 0 | 0 | 1.2976 | . | 1.2976 | . |
| antihipertensive:thiazolidindione | 0 | 0 | 1.8558 | . | 1.8558 | . |
| antihipertensive:thiazolidindione +intensive lifestyle | 0 | 0 | 2.3149 | . | 2.3149 | . |
| antihipertensive:Vitamin D | 0 | 0 | 1.0950 | . | 1.0950 | . |
| control:diet | 2 | 0.83 | 1.7131 | 1.5525 | 2.7373 | 0.1970 |
| control:enhanced education | 3 | 0.51 | 1.1266 | 1.1136 | 1.1401 | 0.9041 |
| control:GLP1 | 6 | 1.00 | 3.9140 | 3.9140 | . | . |
| control:GLP1+GIP | 1 | 1.00 | 10.1600 | 10.1600 | . | . |
| control:intensive lifestyle intervention | 7 | 0.52 | 1.8360 | 1.5340 | 2.2375 | 0.0312 |
| control:L-arginine | 1 | 0.60 | 1.8092 | 1.4444 | 2.5421 | 0.1484 |
| control:lifestyle intervention/education | 5 | 0.31 | 1.3935 | 1.6761 | 1.2816 | 0.1276 |
| control:lipid low | 2 | 1.00 | 1.1477 | 1.1477 | . | . |
| control:Metformin | 4 | 0.36 | 1.5526 | 1.4656 | 1.6046 | 0.6973 |
| control:Metformin + intensive lifestyle intervention | 1 | 1.00 | 3.6442 | 3.6442 | . | . |
| control:physical activity | 3 | 0.92 | 1.8887 | 1.8110 | 3.0379 | 0.2770 |
| control:Sulfonylureas | 1 | 1.00 | 1.3220 | 1.3220 | . | . |
| control:thiazolidindione | 2 | 0.22 | 1.8907 | 3.6383 | 1.5724 | 0.0173 |
| control:thiazolidindione +intensive lifestyle | 0 | 0 | 2.3585 | . | 2.3585 | . |
| control:Vitamin D | 4 | 1.00 | 1.1156 | 1.1156 | . | . |
| diet:enhanced education | 0 | 0 | 0.6577 | . | 0.6577 | . |
| diet:GLP1 | 0 | 0 | 2.2848 | . | 2.2848 | . |
| diet:GLP1+GIP | 0 | 0 | 5.9309 | . | 5.9309 | . |
| diet:intensive lifestyle intervention | 1 | 0.69 | 1.0718 | 1.0194 | 1.1974 | 0.6686 |
| diet:L-arginine | 0 | 0 | 1.0561 | . | 1.0561 | . |
| diet:lifestyle intervention/education | 0 | 0 | 0.8135 | . | 0.8135 | . |
| diet:lipid low | 0 | 0 | 0.6700 | . | 0.6700 | . |
| diet:Metformin | 0 | 0 | 0.9063 | . | 0.9063 | . |
| diet:Metformin + intensive lifestyle intervention | 0 | 0 | 2.1273 | . | 2.1273 | . |
| diet:physical activity | 1 | 0.79 | 1.1025 | 1.0671 | 1.2448 | 0.7346 |
| diet:Sulfonylureas | 0 | 0 | 0.7717 | . | 0.7717 | . |
| diet:thiazolidindione | 0 | 0 | 1.1037 | . | 1.1037 | . |
| diet:thiazolidindione +intensive lifestyle | 0 | 0 | 1.3768 | . | 1.3768 | . |
| diet:Vitamin D | 0 | 0 | 0.6512 | . | 0.6512 | . |
| enhanced education:GLP1 | 0 | 0 | 3.4741 | . | 3.4741 | . |
| enhanced education:GLP1+GIP | 0 | 0 | 9.0182 | . | 9.0182 | . |
| enhanced education:intensive lifestyle intervention | 0 | 0 | 1.6297 | . | 1.6297 | . |
| enhanced education:L-arginine | 0 | 0 | 1.6059 | . | 1.6059 | . |
| enhanced education:lifestyle intervention/education | 5 | 0.72 | 1.2369 | 1.2287 | 1.2580 | 0.9041 |
| enhanced education:lipid low | 0 | 0 | 1.0187 | . | 1.0187 | . |
| enhanced education:Metformin | 0 | 0 | 1.3781 | . | 1.3781 | . |
| enhanced education:Metformin + intensive lifestyle intervention | 0 | 0 | 3.2346 | . | 3.2346 | . |
| enhanced education:physical activity | 0 | 0 | 1.6765 | . | 1.6765 | . |
| enhanced education:Sulfonylureas | 0 | 0 | 1.1735 | . | 1.1735 | . |
| enhanced education:thiazolidindione | 0 | 0 | 1.6782 | . | 1.6782 | . |
| enhanced education:thiazolidindione +intensive lifestyle | 0 | 0 | 2.0934 | . | 2.0934 | . |
| enhanced education:Vitamin D | 0 | 0 | 0.9902 | . | 0.9902 | . |
| GLP1:GLP1+GIP | 0 | 0 | 2.5958 | . | 2.5958 | . |
| GLP1:intensive lifestyle intervention | 0 | 0 | 0.4691 | . | 0.4691 | . |
| GLP1:L-arginine | 0 | 0 | 0.4622 | . | 0.4622 | . |
| GLP1:lifestyle intervention/education | 0 | 0 | 0.3560 | . | 0.3560 | . |
| GLP1:lipid low | 0 | 0 | 0.2932 | . | 0.2932 | . |
| GLP1:Metformin | 0 | 0 | 0.3967 | . | 0.3967 | . |
| GLP1:Metformin + intensive lifestyle intervention | 0 | 0 | 0.9311 | . | 0.9311 | . |
| GLP1:physical activity | 0 | 0 | 0.4826 | . | 0.4826 | . |
| GLP1:Sulfonylureas | 0 | 0 | 0.3378 | . | 0.3378 | . |
| GLP1:thiazolidindione | 0 | 0 | 0.4831 | . | 0.4831 | . |
| GLP1:thiazolidindione +intensive lifestyle | 0 | 0 | 0.6026 | . | 0.6026 | . |
| GLP1:Vitamin D | 0 | 0 | 0.2850 | . | 0.2850 | . |
| GLP1+GIP:intensive lifestyle intervention | 0 | 0 | 0.1807 | . | 0.1807 | . |
| GLP1+GIP:L-arginine | 0 | 0 | 0.1781 | . | 0.1781 | . |
| GLP1+GIP:lifestyle intervention/education | 0 | 0 | 0.1372 | . | 0.1372 | . |
| GLP1+GIP:lipid low | 0 | 0 | 0.1130 | . | 0.1130 | . |
| GLP1+GIP:Metformin | 0 | 0 | 0.1528 | . | 0.1528 | . |
| GLP1+GIP:Metformin + intensive lifestyle intervention | 0 | 0 | 0.3587 | . | 0.3587 | . |
| GLP1+GIP:physical activity | 0 | 0 | 0.1859 | . | 0.1859 | . |
| GLP1+GIP:Sulfonylureas | 0 | 0 | 0.1301 | . | 0.1301 | . |
| GLP1+GIP:thiazolidindione | 0 | 0 | 0.1861 | . | 0.1861 | . |
| GLP1+GIP:thiazolidindione +intensive lifestyle | 0 | 0 | 0.2321 | . | 0.2321 | . |
| GLP1+GIP:Vitamin D | 0 | 0 | 0.1098 | . | 0.1098 | . |
| intensive lifestyle intervention:L-arginine | 0 | 0 | 0.9854 | . | 0.9854 | . |
| intensive lifestyle intervention:lifestyle intervention/education | 8 | 0.77 | 0.7590 | 0.7136 | 0.9340 | 0.1438 |
| intensive lifestyle intervention:lipid low | 0 | 0 | 0.6251 | . | 0.6251 | . |
| intensive lifestyle intervention:Metformin | 2 | 0.40 | 0.8456 | 0.6975 | 0.9638 | 0.1614 |
| intensive lifestyle intervention:Metformin + intensive lifestyle intervention | 0 | 0 | 1.9849 | . | 1.9849 | . |
| intensive lifestyle intervention:physical activity | 1 | 0.48 | 1.0287 | 1.0469 | 1.0124 | 0.9086 |
| intensive lifestyle intervention:Sulfonylureas | 0 | 0 | 0.7201 | . | 0.7201 | . |
| intensive lifestyle intervention:thiazolidindione | 1 | 0.42 | 1.0298 | 1.1156 | 0.9711 | 0.6317 |
| intensive lifestyle intervention:thiazolidindione +intensive lifestyle | 1 | 0.78 | 1.2846 | 1.4603 | 0.8191 | 0.2367 |
| intensive lifestyle intervention:Vitamin D | 0 | 0 | 0.6076 | . | 0.6076 | . |
| L-arginine:lifestyle intervention/education | 1 | 0.44 | 0.7702 | 0.5625 | 0.9900 | 0.1484 |
| L-arginine:lipid low | 0 | 0 | 0.6344 | . | 0.6344 | . |
| L-arginine:Metformin | 0 | 0 | 0.8582 | . | 0.8582 | . |

8. Figure S3: Network plot of possible treatments for reducing HbA1c – time-point: 1 year. Every knot represents a different therapy for reducing HbA1c. The larger a knot, the more studies included that treatment. Every edge compares different therapies. The width and the number above indicate how many studies investigated this comparison.

9. Table S3 : Direct and Indirect Comparisons of the treatment for reducing HbA1c, with p-values (Netsplit HbA1c 1 year)

| **Comparison** | **k** | **Prop** | **NMA** | **Direct** | **Indirect** | **Diff** | **p-value** |
| --- | --- | --- | --- | --- | --- | --- | --- |
| control:AGI | 1 | 1.00 | -0.0000 | -0.0000 | . | . | . |
| curcumin:AGI | 0 | 0 | -0.4890 | . | -0.4890 | . | . |
| diet:AGI | 0 | 0 | -0.1049 | . | -0.1049 | . | . |
| DPP4:AGI | 0 | 0 | -0.0044 | . | -0.0044 | . | . |
| enhanced_education:AGI | 0 | 0 | -0.0266 | . | -0.0266 | . | . |
| GLP1:AGI | 0 | 0 | -0.4077 | . | -0.4077 | . | . |
| GLP1+GIP:AGI | 0 | 0 | -0.4616 | . | -0.4616 | . | . |
| intensive lifestyle intervention:AGI | 0 | 0 | -0.1618 | . | -0.1618 | . | . |
| L-arginine + lifestyle intervention:AGI | 0 | 0 | 0.0417 | . | 0.0417 | . | . |
| lifestyle_intervention_education:AGI | 0 | 0 | -0.1083 | . | -0.1083 | . | . |
| Metformin:AGI | 0 | 0 | -0.0995 | . | -0.0995 | . | . |
| Metformin + DPP4:AGI | 0 | 0 | -0.2995 | . | -0.2995 | . | . |
| physical_activity:AGI | 0 | 0 | -0.3546 | . | -0.3546 | . | . |
| Thiazolidindione:AGI | 0 | 0 | -0.0900 | . | -0.0900 | . | . |
| Vitamin D:AGI | 0 | 0 | -0.0500 | . | -0.0500 | . | . |
| Zinc:AGI | 0 | 0 | -0.0900 | . | -0.0900 | . | . |
| control:curcumin | 1 | 1.00 | 0.4890 | 0.4890 | . | . | . |
| control:diet | 4 | 1.00 | 0.1049 | 0.1049 | . | . | . |
| control:DPP4 | 0 | 0 | 0.0044 | . | 0.0044 | . | . |
| control:enhanced_education | 4 | 0.63 | 0.0266 | 0.0261 | 0.0275 | -0.0014 | 0.9848 |
| control:GLP1 | 3 | 1.00 | 0.4077 | 0.4077 | . | . | . |
| control:GLP1+GIP | 1 | 1.00 | 0.4616 | 0.4616 | . | . | . |
| control:intensive lifestyle intervention | 5 | 0.65 | 0.1618 | 0.1636 | 0.1584 | 0.0052 | 0.9436 |
| control:L-arginine + lifestyle intervention | 0 | 0 | -0.0417 | . | -0.0417 | . | . |
| control:lifestyle_intervention_education | 2 | 0.26 | 0.1083 | 0.1294 | 0.1008 | 0.0286 | 0.7194 |
| control:Metformin | 1 | 0.45 | 0.0995 | 0.0000 | 0.1819 | -0.1819 | 0.1781 |
| control:Metformin + DPP4 | 0 | 0 | 0.2995 | . | 0.2995 | . | . |
| control:physical_activity | 4 | 1.00 | 0.3546 | 0.3546 | . | . | . |
| control:Thiazolidindione | 1 | 1.00 | 0.0900 | 0.0900 | . | . | . |
| control:Vitamin D | 5 | 1.00 | 0.0500 | 0.0500 | . | . | . |
| control:Zinc | 1 | 1.00 | 0.0900 | 0.0900 | . | . | . |
| curcumin:diet | 0 | 0 | -0.3841 | . | -0.3841 | . | . |
| curcumin:DPP4 | 0 | 0 | -0.4846 | . | -0.4846 | . | . |
| curcumin:enhanced_education | 0 | 0 | -0.4624 | . | -0.4624 | . | . |
| curcumin:GLP1 | 0 | 0 | -0.0813 | . | -0.0813 | . | . |
| curcumin:GLP1+GIP | 0 | 0 | -0.0274 | . | -0.0274 | . | . |
| curcumin:intensive lifestyle intervention | 0 | 0 | -0.3272 | . | -0.3272 | . | . |
| curcumin:L-arginine + lifestyle intervention | 0 | 0 | -0.5307 | . | -0.5307 | . | . |
| curcumin:lifestyle_intervention_education | 0 | 0 | -0.3807 | . | -0.3807 | . | . |
| curcumin:Metformin | 0 | 0 | -0.3895 | . | -0.3895 | . | . |
| curcumin:Metformin + DPP4 | 0 | 0 | -0.1895 | . | -0.1895 | . | . |
| curcumin:physical_activity | 0 | 0 | -0.1344 | . | -0.1344 | . | . |
| curcumin:Thiazolidindione | 0 | 0 | -0.3990 | . | -0.3990 | . | . |
| curcumin:Vitamin D | 0 | 0 | -0.4390 | . | -0.4390 | . | . |
| curcumin:Zinc | 0 | 0 | -0.3990 | . | -0.3990 | . | . |
| diet:DPP4 | 0 | 0 | -0.1005 | . | -0.1005 | . | . |
| diet:enhanced_education | 0 | 0 | -0.0783 | . | -0.0783 | . | . |
| diet:GLP1 | 0 | 0 | 0.3027 | . | 0.3027 | . | . |
| diet:GLP1+GIP | 0 | 0 | 0.3567 | . | 0.3567 | . | . |
| diet:intensive lifestyle intervention | 0 | 0 | 0.0569 | . | 0.0569 | . | . |
| diet:L-arginine + lifestyle intervention | 0 | 0 | -0.1466 | . | -0.1466 | . | . |
| diet:lifestyle_intervention_education | 0 | 0 | 0.0034 | . | 0.0034 | . | . |
| diet:Metformin | 0 | 0 | -0.0054 | . | -0.0054 | . | . |
| diet:Metformin + DPP4 | 0 | 0 | 0.1946 | . | 0.1946 | . | . |
| diet:physical_activity | 0 | 0 | 0.2496 | . | 0.2496 | . | . |
| diet:Thiazolidindione | 0 | 0 | -0.0149 | . | -0.0149 | . | . |
| diet:Vitamin D | 0 | 0 | -0.0549 | . | -0.0549 | . | . |
| diet:Zinc | 0 | 0 | -0.0149 | . | -0.0149 | . | . |
| DPP4:enhanced_education | 0 | 0 | 0.0222 | . | 0.0222 | . | . |
| DPP4:GLP1 | 0 | 0 | 0.4032 | . | 0.4032 | . | . |
| DPP4:GLP1+GIP | 0 | 0 | 0.4572 | . | 0.4572 | . | . |
| DPP4:intensive lifestyle intervention | 0 | 0 | 0.1574 | . | 0.1574 | . | . |
| DPP4:L-arginine + lifestyle intervention | 0 | 0 | -0.0461 | . | -0.0461 | . | . |
| DPP4:lifestyle_intervention_education | 1 | 0.86 | 0.1039 | 0.1000 | 0.1280 | -0.0280 | 0.9106 |
| DPP4:Metformin | 1 | 0.81 | 0.0951 | 0.1000 | 0.0747 | 0.0253 | 0.9106 |
| DPP4:Metformin + DPP4 | 0 | 0 | 0.2951 | . | 0.2951 | . | . |
| DPP4:physical_activity | 0 | 0 | 0.3501 | . | 0.3501 | . | . |
| DPP4:Thiazolidindione | 0 | 0 | 0.0856 | . | 0.0856 | . | . |
| DPP4:Vitamin D | 0 | 0 | 0.0456 | . | 0.0456 | . | . |
| DPP4:Zinc | 0 | 0 | 0.0856 | . | 0.0856 | . | . |
| enhanced_education:GLP1 | 0 | 0 | 0.3811 | . | 0.3811 | . | . |
| enhanced_education:GLP1+GIP | 0 | 0 | 0.4350 | . | 0.4350 | . | . |
| enhanced_education:intensive lifestyle intervention | 0 | 0 | 0.1352 | . | 0.1352 | . | . |
| enhanced_education:L-arginine + lifestyle intervention | 0 | 0 | -0.0683 | . | -0.0683 | . | . |
| enhanced_education:lifestyle_intervention_education | 6 | 0.72 | 0.0817 | 0.0813 | 0.0827 | -0.0014 | 0.9848 |
| enhanced_education:Metformin | 0 | 0 | 0.0729 | . | 0.0729 | . | . |
| enhanced_education:Metformin + DPP4 | 0 | 0 | 0.2729 | . | 0.2729 | . | . |
| enhanced_education:physical_activity | 0 | 0 | 0.3280 | . | 0.3280 | . | . |
| enhanced_education:Thiazolidindione | 0 | 0 | 0.0634 | . | 0.0634 | . | . |
| enhanced_education:Vitamin D | 0 | 0 | 0.0234 | . | 0.0234 | . | . |
| enhanced_education:Zinc | 0 | 0 | 0.0634 | . | 0.0634 | . | . |
| GLP1:GLP1+GIP | 0 | 0 | 0.0540 | . | 0.0540 | . | . |
| GLP1:intensive lifestyle intervention | 0 | 0 | -0.2459 | . | -0.2459 | . | . |
| GLP1:L-arginine + lifestyle intervention | 0 | 0 | -0.4494 | . | -0.4494 | . | . |
| GLP1:lifestyle_intervention_education | 0 | 0 | -0.2994 | . | -0.2994 | . | . |
| GLP1:Metformin | 0 | 0 | -0.3081 | . | -0.3081 | . | . |
| GLP1:Metformin + DPP4 | 0 | 0 | -0.1081 | . | -0.1081 | . | . |
| GLP1:physical_activity | 0 | 0 | -0.0531 | . | -0.0531 | . | . |
| GLP1:Thiazolidindione | 0 | 0 | -0.3177 | . | -0.3177 | . | . |
| GLP1:Vitamin D | 0 | 0 | -0.3576 | . | -0.3576 | . | . |
| GLP1:Zinc | 0 | 0 | -0.3177 | . | -0.3177 | . | . |
| GLP1+GIP:intensive lifestyle intervention | 0 | 0 | -0.2998 | . | -0.2998 | . | . |
| GLP1+GIP:L-arginine + lifestyle intervention | 0 | 0 | -0.5033 | . | -0.5033 | . | . |
| GLP1+GIP:lifestyle_intervention_education | 0 | 0 | -0.3533 | . | -0.3533 | . | . |
| GLP1+GIP:Metformin | 0 | 0 | -0.3621 | . | -0.3621 | . | . |
| GLP1+GIP:Metformin + DPP4 | 0 | 0 | -0.1621 | . | -0.1621 | . | . |
| GLP1+GIP:physical_activity | 0 | 0 | -0.1071 | . | -0.1071 | . | . |
| GLP1+GIP:Thiazolidindione | 0 | 0 | -0.3716 | . | -0.3716 | . | . |
| GLP1+GIP:Vitamin D | 0 | 0 | -0.4116 | . | -0.4116 | . | . |
| GLP1+GIP:Zinc | 0 | 0 | -0.3716 | . | -0.3716 | . | . |
| intensive lifestyle intervention:L-arginine + lifestyle intervention | 0 | 0 | -0.2035 | . | -0.2035 | . | . |
| intensive lifestyle intervention:lifestyle_intervention_education | 7 | 0.69 | -0.0535 | -0.0534 | -0.0537 | 0.0003 | 0.9963 |
| intensive lifestyle intervention:Metformin | 2 | 0.53 | -0.0623 | -0.0520 | -0.0736 | 0.0216 | 0.8705 |
| intensive lifestyle intervention:Metformin + DPP4 | 0 | 0 | 0.1377 | . | 0.1377 | . | . |
| intensive lifestyle intervention:physical_activity | 0 | 0 | 0.1928 | . | 0.1928 | . | . |
| intensive lifestyle intervention:Thiazolidindione | 0 | 0 | -0.0718 | . | -0.0718 | . | . |
| intensive lifestyle intervention:Vitamin D | 0 | 0 | -0.1118 | . | -0.1118 | . | . |
| intensive lifestyle intervention:Zinc | 0 | 0 | -0.0718 | . | -0.0718 | . | . |
| L-arginine + lifestyle intervention:lifestyle_intervention_education | 1 | 1.00 | 0.1500 | 0.1500 | . | . | . |
| L-arginine + lifestyle intervention:Metformin | 0 | 0 | 0.1412 | . | 0.1412 | . | . |
| L-arginine + lifestyle intervention:Metformin + DPP4 | 0 | 0 | 0.3412 | . | 0.3412 | . | . |

10. Figure S4: Network plot of possible treatments for reducing HbA1c- sensitivity analysis – time-point: 1 year

11. Figure S5: Network plot of possible treatments for reducing HbA1c – time-point: 6 months

12. Figure S6: Network plot of possible treatments for reducing HbA1c – sensitivity analysis – time-point: 6 months

13. Figure S7: Network plot of possible treatments for reducing FPG – time-point: 1 year. Every knot represents a different therapy for reducing FPG. The larger a knot, the more studies included that treatment. Every edge compares different therapies. The width and the number above indicate how many studies investigated this comparison.

14. Table S4 : Direct and Indirect Comparisons of the treatment for reducing FPG at 1 year, with p-values (Netsplit FPG 1 year)

| Comparison | k | Prop | NMA | Direct | Indirect | Diff | p-value |
| --- | --- | --- | --- | --- | --- | --- | --- |
| diet:control | 2 | 1.00 | -0.0547 | -0.0547 | . | . | . |
| DPP4:control | 0 | 0 | -0.3098 | . | -0.3098 | . | . |
| enhanced_education:control | 4 | 0.62 | -0.0716 | -0.0038 | -0.1832 | 0.1794 | 0.2267 |
| GLP1:control | 2 | 1.00 | -0.5254 | -0.5254 | . | . | . |
| GLP1+GIP:control | 1 | 1.00 | -0.5437 | -0.5437 | . | . | . |
| intensive lifestyle intervention:control | 16 | 0.72 | -0.2212 | -0.2111 | -0.2470 | 0.0359 | 0.7046 |
| lifestyle_intervention_education:control | 10 | 0.55 | -0.1249 | -0.1739 | -0.0644 | -0.1095 | 0.2197 |
| Metformin:control | 2 | 0.40 | -0.2790 | -0.2243 | -0.3159 | 0.0916 | 0.6768 |
| Metformin + DPP4:control | 0 | 0 | -0.6188 | . | -0.6188 | . | . |
| physical_activity:control | 4 | 1.00 | -0.6803 | -0.6803 | . | . | . |
| sulfonylureas:control | 1 | 1.00 | -1.1667 | -1.1667 | . | . | . |
| Thiazolidindione:control | 1 | 1.00 | -0.4889 | -0.4889 | . | . | . |
| vitamin D:control | 5 | 1.00 | -0.2926 | -0.2926 | . | . | . |
| Zinc:control | 2 | 1.00 | -0.6215 | -0.6215 | . | . | . |
| diet:DPP4 | 0 | 0 | 0.2551 | . | 0.2551 | . | . |
| diet:enhanced_education | 0 | 0 | 0.0169 | . | 0.0169 | . | . |
| diet:GLP1 | 0 | 0 | 0.4707 | . | 0.4707 | . | . |
| diet:GLP1+GIP | 0 | 0 | 0.4890 | . | 0.4890 | . | . |
| diet:intensive lifestyle intervention | 0 | 0 | 0.1666 | . | 0.1666 | . | . |
| diet:lifestyle_intervention_education | 0 | 0 | 0.0702 | . | 0.0702 | . | . |
| diet:Metformin | 0 | 0 | 0.2243 | . | 0.2243 | . | . |
| diet:Metformin + DPP4 | 0 | 0 | 0.5642 | . | 0.5642 | . | . |
| diet:physical_activity | 0 | 0 | 0.6256 | . | 0.6256 | . | . |
| diet:sulfonylureas | 0 | 0 | 1.1120 | . | 1.1120 | . | . |
| diet:Thiazolidindione | 0 | 0 | 0.4342 | . | 0.4342 | . | . |
| diet:vitamin D | 0 | 0 | 0.2380 | . | 0.2380 | . | . |
| diet:Zinc | 0 | 0 | 0.5668 | . | 0.5668 | . | . |
| DPP4:enhanced_education | 0 | 0 | -0.2382 | . | -0.2382 | . | . |
| DPP4:GLP1 | 0 | 0 | 0.2156 | . | 0.2156 | . | . |
| DPP4:GLP1+GIP | 0 | 0 | 0.2339 | . | 0.2339 | . | . |
| DPP4:intensive lifestyle intervention | 0 | 0 | -0.0886 | . | -0.0886 | . | . |
| DPP4:lifestyle_intervention_education | 1 | 0.82 | -0.1849 | -0.0700 | -0.7172 | 0.6472 | 0.1407 |
| DPP4:Metformin | 1 | 0.77 | -0.0308 | -0.0700 | 0.1019 | -0.1719 | 0.6590 |
| DPP4:Metformin + DPP4 | 1 | 0.86 | 0.3090 | 0.2300 | 0.8020 | -0.5720 | 0.2548 |
| DPP4:physical_activity | 0 | 0 | 0.3705 | . | 0.3705 | . | . |
| DPP4:sulfonylureas | 0 | 0 | 0.8569 | . | 0.8569 | . | . |
| DPP4:Thiazolidindione | 0 | 0 | 0.1791 | . | 0.1791 | . | . |
| DPP4:vitamin D | 0 | 0 | -0.0172 | . | -0.0172 | . | . |
| DPP4:Zinc | 0 | 0 | 0.3117 | . | 0.3117 | . | . |
| enhanced_education:GLP1 | 0 | 0 | 0.4538 | . | 0.4538 | . | . |
| enhanced_education:GLP1+GIP | 0 | 0 | 0.4721 | . | 0.4721 | . | . |
| enhanced_education:intensive lifestyle intervention | 0 | 0 | 0.1496 | . | 0.1496 | . | . |
| enhanced_education:lifestyle_intervention_education | 4 | 0.59 | 0.0533 | -0.0272 | 0.1706 | -0.1978 | 0.1793 |
| enhanced_education:Metformin | 0 | 0 | 0.2074 | . | 0.2074 | . | . |
| enhanced_education:Metformin + DPP4 | 0 | 0 | 0.5472 | . | 0.5472 | . | . |
| enhanced_education:physical_activity | 0 | 0 | 0.6087 | . | 0.6087 | . | . |
| enhanced_education:sulfonylureas | 0 | 0 | 1.0951 | . | 1.0951 | . | . |
| enhanced_education:Thiazolidindione | 0 | 0 | 0.4173 | . | 0.4173 | . | . |
| enhanced_education:vitamin D | 0 | 0 | 0.2210 | . | 0.2210 | . | . |
| enhanced_education:Zinc | 0 | 0 | 0.5499 | . | 0.5499 | . | . |
| GLP1:GLP1+GIP | 0 | 0 | 0.0183 | . | 0.0183 | . | . |
| GLP1:intensive lifestyle intervention | 0 | 0 | -0.3042 | . | -0.3042 | . | . |
| GLP1:lifestyle_intervention_education | 0 | 0 | -0.4005 | . | -0.4005 | . | . |
| GLP1:Metformin | 0 | 0 | -0.2464 | . | -0.2464 | . | . |
| GLP1:Metformin + DPP4 | 0 | 0 | 0.0934 | . | 0.0934 | . | . |
| GLP1:physical_activity | 0 | 0 | 0.1549 | . | 0.1549 | . | . |
| GLP1:sulfonylureas | 0 | 0 | 0.6413 | . | 0.6413 | . | . |
| GLP1:Thiazolidindione | 0 | 0 | -0.0365 | . | -0.0365 | . | . |
| GLP1:vitamin D | 0 | 0 | -0.2328 | . | -0.2328 | . | . |
| GLP1:Zinc | 0 | 0 | 0.0961 | . | 0.0961 | . | . |
| GLP1+GIP:intensive lifestyle intervention | 0 | 0 | -0.3225 | . | -0.3225 | . | . |
| GLP1+GIP:lifestyle_intervention_education | 0 | 0 | -0.4188 | . | -0.4188 | . | . |
| GLP1+GIP:Metformin | 0 | 0 | -0.2647 | . | -0.2647 | . | . |
| GLP1+GIP:Metformin + DPP4 | 0 | 0 | 0.0751 | . | 0.0751 | . | . |
| GLP1+GIP:physical_activity | 0 | 0 | 0.1366 | . | 0.1366 | . | . |
| GLP1+GIP:sulfonylureas | 0 | 0 | 0.6230 | . | 0.6230 | . | . |
| GLP1+GIP:Thiazolidindione | 0 | 0 | -0.0548 | . | -0.0548 | . | . |
| GLP1+GIP:vitamin D | 0 | 0 | -0.2511 | . | -0.2511 | . | . |
| GLP1+GIP:Zinc | 0 | 0 | 0.0778 | . | 0.0778 | . | . |
| intensive lifestyle intervention:lifestyle_intervention_education | 14 | 0.69 | -0.0964 | -0.1254 | -0.0312 | -0.0941 | 0.3113 |
| intensive lifestyle intervention:Metformin | 2 | 0.44 | 0.0578 | 0.1368 | -0.0039 | 0.1408 | 0.5167 |
| intensive lifestyle intervention:Metformin + DPP4 | 0 | 0 | 0.3976 | . | 0.3976 | . | . |
| intensive lifestyle intervention:physical_activity | 0 | 0 | 0.4591 | . | 0.4591 | . | . |
| intensive lifestyle intervention:sulfonylureas | 0 | 0 | 0.9455 | . | 0.9455 | . | . |
| intensive lifestyle intervention:Thiazolidindione | 0 | 0 | 0.2677 | . | 0.2677 | . | . |
| intensive lifestyle intervention:vitamin D | 0 | 0 | 0.0714 | . | 0.0714 | . | . |
| intensive lifestyle intervention:Zinc | 0 | 0 | 0.4003 | . | 0.4003 | . | . |
| lifestyle_intervention_education:Metformin | 2 | 0.49 | 0.1541 | 0.2292 | 0.0832 | 0.1461 | 0.4927 |
| lifestyle_intervention_education:Metformin + DPP4 | 1 | 0.60 | 0.4940 | 0.3000 | 0.7840 | -0.4840 | 0.1011 |
| lifestyle_intervention_education:physical_activity | 0 | 0 | 0.5554 | . | 0.5554 | . | . |
| lifestyle_intervention_education:sulfonylureas | 0 | 0 | 1.0418 | . | 1.0418 | . | . |
| lifestyle_intervention_education:Thiazolidindione | 0 | 0 | 0.3640 | . | 0.3640 | . | . |
| lifestyle_intervention_education:vitamin D | 0 | 0 | 0.1678 | . | 0.1678 | . | . |
| lifestyle_intervention_education:Zinc | 0 | 0 | 0.4966 | . | 0.4966 | . | . |
| Metformin:Metformin + DPP4 | 2 | 0.91 | 0.3398 | 0.3990 | -0.2542 | 0.6531 | 0.1407 |
| Metformin:physical_activity | 0 | 0 | 0.4013 | . | 0.4013 | . | . |
| Metformin:sulfonylureas | 0 | 0 | 0.8877 | . | 0.8877 | . | . |
| Metformin:Thiazolidindione | 0 | 0 | 0.2099 | . | 0.2099 | . | . |
| Metformin:vitamin D | 0 | 0 | 0.0136 | . | 0.0136 | . | . |
| Metformin:Zinc | 0 | 0 | 0.3425 | . | 0.3425 | . | . |
| Metformin + DPP4:physical_activity | 0 | 0 | 0.0614 | . | 0.0614 | . | . |
| Metformin + DPP4:sulfonylureas | 0 | 0 | 0.5478 | . | 0.5478 | . | . |
| Metformin + DPP4:Thiazolidindione | 0 | 0 | -0.1299 | . | -0.1299 | . | . |
| Metformin + DPP4:vitamin D | 0 | 0 | -0.3262 | . | -0.3262 | . | . |
| Metformin + DPP4:Zinc | 0 | 0 | 0.0027 | . | 0.0027 | . | . |
| physical_activity:sulfonylureas | 0 | 0 | 0.4864 | . | 0.4864 | . | . |
| physical_activity:Thiazolidindione | 0 | 0 | -0.1914 | . | -0.1914 | . | . |
| physical_activity:vitamin D | 0 | 0 | -0.3876 | . | -0.3876 | . | . |
| physical_activity:Zinc | 0 | 0 | -0.0588 | . | -0.0588 | . | . |
| sulfonylureas:Thiazolidindione | 0 | 0 | -0.6778 | . | -0.6778 | . | . |
| sulfonylureas:vitamin D | 0 | 0 | -0.8740 | . | -0.8740 | . | . |
| sulfonylureas:Zinc | 0 | 0 | -0.5452 | . | -0.5452 | . | . |
| Thiazolidindione:vitamin D | 0 | 0 | -0.1963 | . | -0.1963 | . | . |
| Thiazolidindione:Zinc | 0 | 0 | 0.1326 | . | 0.1326 | . | . |
| vitamin D:Zinc | 0 | 0 | 0.3289 | . | 0.3289 | . | . |

15. Figure S8: Network plot of possible treatments for reducing FPG - sensitivity analysis – time-point: 1 year

16. Figure S9: Network plot of possible treatments for reducing FPG – time-point: 6 months

17. Figure S10: Network plot of possible treatments for reducing FPG -sensitivity analysis – time-point: 6 months

18. Figure S11: Network plot of possible treatments for reducing 2hPG – time-point: 1 year. Every knot represents a different therapy for reducing 2hPG. The larger a knot, the more studies included that treatment. Every edge compares different therapies. The width and the number above indicate how many studies investigated this comparison.

19. Table S5: : Direct and Indirect Comparisons of the treatment for reducing 2hPG at 1 year, with p-values (Netsplit 2hPG – 1 year)

| Comparison | k | prop | nma | direct | indir. | Diff | p-value |
| --- | --- | --- | --- | --- | --- | --- | --- |
| control:AGI | 1 | 1.00 | 0.3000 | 0.3000 | . | . | . |
| diet:AGI | 0 | 0 | -0.3985 | . | -0.3985 | . | . |
| DPP4:AGI | 0 | 0 | -0.7576 | . | -0.7576 | . | . |
| enhanced_education:AGI | 0 | 0 | -0.1963 | . | -0.1963 | . | . |
| intensive lifestyle intervention:AGI | 0 | 0 | -0.5668 | . | -0.5668 | . | . |
| L-arginine + lifestyle intervention:AGI | 0 | 0 | -0.4688 | . | -0.4688 | . | . |
| lifestyle_intervention_education:AGI | 0 | 0 | -0.1749 | . | -0.1749 | . | . |
| Metformin:AGI | 0 | 0 | -0.5530 | . | -0.5530 | . | . |
| physical_activity:AGI | 0 | 0 | -0.6211 | . | -0.6211 | . | . |
| Sulfonylureas:AGI | 0 | 0 | -0.9778 | . | -0.9778 | . | . |
| Thiazolidindione:AGI | 0 | 0 | -0.2333 | . | -0.2333 | . | . |
| Vitamin D:AGI | 0 | 0 | -0.0704 | . | -0.0704 | . | . |
| Zinc:AGI | 0 | 0 | -1.4444 | . | -1.4444 | . | . |
| control:diet | 4 | 1.00 | 0.6985 | 0.6985 | . | . | . |
| control:DPP4 | 0 | 0 | 1.0576 | . | 1.0576 | . | . |
| control:enhanced_education | 2 | 0.63 | 0.4963 | 0.2368 | 0.9368 | -0.7000 | -1.20 |
| control:intensive lifestyle intervention | 6 | 0.72 | 0.8668 | 0.8966 | 0.7905 | 0.1061 | 0.28 |
| control:L-arginine + lifestyle intervention | 0 | 0 | 0.7688 | . | 0.7688 | . | . |
| control:lifestyle_intervention_education | 3 | 0.48 | 0.4749 | 0.4398 | 0.5072 | -0.0673 | -0.19 |
| control:Metformin | 1 | 0.41 | 0.8530 | 1.5000 | 0.4110 | 1.0890 | 1.41 |
| control:physical_activity | 4 | 1.00 | 0.9211 | 0.9211 | . | . | . |
| control:Sulfonylureas | 1 | 1.00 | 1.2778 | 1.2778 | . | . | . |
| control:Thiazolidindione | 1 | 1.00 | 0.5333 | 0.5333 | . | . | . |
| control:Vitamin D | 5 | 1.00 | 0.3704 | 0.3704 | . | . | . |
| control:Zinc | 1 | 1.00 | 1.7444 | 1.7444 | . | . | . |
| diet:DPP4 | 0 | 0 | 0.3591 | . | 0.3591 | . | . |
| diet:enhanced_education | 0 | 0 | -0.2022 | . | -0.2022 | . | . |
| diet:intensive lifestyle intervention | 0 | 0 | 0.1683 | . | 0.1683 | . | . |
| diet:L-arginine + lifestyle intervention | 0 | 0 | 0.0703 | . | 0.0703 | . | . |
| diet:lifestyle_intervention_education | 0 | 0 | -0.2236 | . | -0.2236 | . | . |
| diet:Metformin | 0 | 0 | 0.1545 | . | 0.1545 | . | . |
| diet:physical_activity | 0 | 0 | 0.2226 | . | 0.2226 | . | . |
| diet:Sulfonylureas | 0 | 0 | 0.5793 | . | 0.5793 | . | . |
| diet:Thiazolidindione | 0 | 0 | -0.1652 | . | -0.1652 | . | . |
| diet:Vitamin D | 0 | 0 | -0.3280 | . | -0.3280 | . | . |
| diet:Zinc | 0 | 0 | 1.0460 | . | 1.0460 | . | . |
| DPP4:enhanced_education | 0 | 0 | -0.5613 | . | -0.5613 | . | . |
| DPP4:intensive lifestyle intervention | 0 | 0 | -0.1908 | . | -0.1908 | . | . |
| DPP4:L-arginine + lifestyle intervention | 0 | 0 | -0.2888 | . | -0.2888 | . | . |
| DPP4:lifestyle_intervention_education | 1 | 0.92 | -0.5827 | -0.4000 | -2.5989 | 2.1989 | 1.41 |
| DPP4:Metformin | 1 | 0.91 | -0.2046 | -0.4000 | 1.7266 | -2.1266 | -1.41 |
| DPP4:physical_activity | 0 | 0 | -0.1365 | . | -0.1365 | . | . |
| DPP4:Sulfonylureas | 0 | 0 | 0.2202 | . | 0.2202 | . | . |
| DPP4:Thiazolidindione | 0 | 0 | -0.5243 | . | -0.5243 | . | . |
| DPP4:Vitamin D | 0 | 0 | -0.6871 | . | -0.6871 | . | . |
| DPP4:Zinc | 0 | 0 | 0.6869 | . | 0.6869 | . | . |
| enhanced_education:intensive lifestyle intervention | 0 | 0 | 0.3705 | . | 0.3705 | . | . |
| enhanced_education:L-arginine + lifestyle intervention | 0 | 0 | 0.2725 | . | 0.2725 | . | . |
| enhanced_education:lifestyle_intervention_education | 1 | 0.48 | -0.0214 | -0.3860 | 0.3140 | -0.7000 | -1.20 |
| enhanced_education:Metformin | 0 | 0 | 0.3567 | . | 0.3567 | . | . |
| enhanced_education:physical_activity | 0 | 0 | 0.4248 | . | 0.4248 | . | . |
| enhanced_education:Sulfonylureas | 0 | 0 | 0.7815 | . | 0.7815 | . | . |
| enhanced_education:Thiazolidindione | 0 | 0 | 0.0370 | . | 0.0370 | . | . |
| enhanced_education:Vitamin D | 0 | 0 | -0.1258 | . | -0.1258 | . | . |
| enhanced_education:Zinc | 0 | 0 | 1.2482 | . | 1.2482 | . | . |
| intensive lifestyle intervention:L-arginine + lifestyle intervention | 0 | 0 | -0.0980 | . | -0.0980 | . | . |
| intensive lifestyle intervention:lifestyle_intervention_education | 4 | 0.64 | -0.3919 | -0.3532 | -0.4592 | 0.1061 | 0.28 |
| intensive lifestyle intervention:Metformin | 0 | 0 | -0.0138 | . | -0.0138 | . | . |
| intensive lifestyle intervention:physical_activity | 0 | 0 | 0.0543 | . | 0.0543 | . | . |
| intensive lifestyle intervention:Sulfonylureas | 0 | 0 | 0.4110 | . | 0.4110 | . | . |
| intensive lifestyle intervention:Thiazolidindione | 0 | 0 | -0.3335 | . | -0.3335 | . | . |
| intensive lifestyle intervention:Vitamin D | 0 | 0 | -0.4964 | . | -0.4964 | . | . |
| intensive lifestyle intervention:Zinc | 0 | 0 | 0.8776 | . | 0.8776 | . | . |
| L-arginine + lifestyle intervention:lifestyle_intervention_education | 1 | 1.00 | -0.2939 | -0.2939 | . | . | . |
| L-arginine + lifestyle intervention:Metformin | 0 | 0 | 0.0842 | . | 0.0842 | . | . |
| L-arginine + lifestyle intervention:physical_activity | 0 | 0 | 0.1523 | . | 0.1523 | . | . |
| L-arginine + lifestyle intervention:Sulfonylureas | 0 | 0 | 0.5090 | . | 0.5090 | . | . |
| L-arginine + lifestyle intervention:Thiazolidindione | 0 | 0 | -0.2355 | . | -0.2355 | . | . |
| L-arginine + lifestyle intervention:Vitamin D | 0 | 0 | -0.3984 | . | -0.3984 | . | . |
| L-arginine + lifestyle intervention:Zinc | 0 | 0 | 0.9756 | . | 0.9756 | . | . |
| lifestyle_intervention_education:Metformin | 1 | 0.65 | 0.3780 | 0.0000 | 1.0890 | -1.0890 | -1.41 |
| lifestyle_intervention_education:physical_activity | 0 | 0 | 0.4462 | . | 0.4462 | . | . |
| lifestyle_intervention_education:Sulfonylureas | 0 | 0 | 0.8029 | . | 0.8029 | . | . |
| lifestyle_intervention_education:Thiazolidindione | 0 | 0 | 0.0584 | . | 0.0584 | . | . |
| lifestyle_intervention_education:Vitamin D | 0 | 0 | -0.1045 | . | -0.1045 | . | . |
| lifestyle_intervention_education:Zinc | 0 | 0 | 1.2695 | . | 1.2695 | . | . |
| Metformin:physical_activity | 0 | 0 | 0.0682 | . | 0.0682 | . | . |
| Metformin:Sulfonylureas | 0 | 0 | 0.4248 | . | 0.4248 | . | . |
| Metformin:Thiazolidindione | 0 | 0 | -0.3196 | . | -0.3196 | . | . |
| Metformin:Vitamin D | 0 | 0 | -0.4825 | . | -0.4825 | . | . |
| Metformin:Zinc | 0 | 0 | 0.8915 | . | 0.8915 | . | . |
| physical_activity:Sulfonylureas | 0 | 0 | 0.3566 | . | 0.3566 | . | . |
| physical_activity:Thiazolidindione | 0 | 0 | -0.3878 | . | -0.3878 | . | . |
| physical_activity:Vitamin D | 0 | 0 | -0.5507 | . | -0.5507 | . | . |
| physical_activity:Zinc | 0 | 0 | 0.8233 | . | 0.8233 | . | . |
| Sulfonylureas:Thiazolidindione | 0 | 0 | -0.7444 | . | -0.7444 | . | . |
| Sulfonylureas:Vitamin D | 0 | 0 | -0.9073 | . | -0.9073 | . | . |
| Sulfonylureas:Zinc | 0 | 0 | 0.4667 | . | 0.4667 | . | . |
| Thiazolidindione:Vitamin D | 0 | 0 | -0.1629 | . | -0.1629 | . | . |
| Thiazolidindione:Zinc | 0 | 0 | 1.2111 | . | 1.2111 | . | . |
| Vitamin D:Zinc | 0 | 0 | 1.3740 | . | 1.3740 | . | . |

20. Figure S12: Network plot of possible treatments for reducing 2hPG – sensitivity analysis – time-point – 1 year

21. Figure S13: Network plot of possible treatments for reducing 2hPG – time-point: 6 months

22. Figure S14: Network plot of possible treatments for reducing 2hPG – sensitivity analysis – time-point: 6 months

23. Figure S15: Change from baseline in FPG with Metformin treatment

N: number of patients, MD: mean difference, FPG: fasting plasma glucose

24. Table S6: League Table

Incidence of type 2 diabetes

HbA1c - approximately 6 months

HbA1c - approximately 1 year

FPG - approximately 6 months

FPG - approximately 1 year

2hPG - approximately 6 months

2hPG - approximately 1 year

25. Table S7: Grading – incidence of T2D (using CINeMA tool)

| Comparison | No of studies | Within-study bias | Reporting bias | Indirectness | Imprecision | Heterogeneity | Incoherence | Confidence rating |
| --- | --- | --- | --- | --- | --- | --- | --- | --- |
| GLP1:control | 6 | No concerns | Low risk | No concerns | No concerns | No concerns | Major concerns | Moderate |
| GLP1+GIP:control | 1 | Some concerns | Low risk | No concerns | No concerns | No concerns | Major concerns | Low |
| L-arginine:control | 1 | Some concerns | Low risk | No concerns | No concerns | No concerns | No concerns | High |
| L-arginine:lifestyle intervention/education | 1 | Some concerns | Low risk | No concerns | Some concerns | Some concerns | No concerns | Low |
| Metformin:control | 4 | Some concerns | Low risk | No concerns | No concerns | No concerns | No concerns | High |
| Metformin:intensive lifestyle intervention | 2 | Some concerns | Low risk | No concerns | Some concerns | Some concerns | No concerns | Low |
| Metformin:lifestyle intervention/education | 2 | Some concerns | Low risk | No concerns | Some concerns | Some concerns | No concerns | Low |
| Metformin + intensive lifestyle intervention:control | 1 | Some concerns | Low risk | No concerns | No concerns | No concerns | Major concerns | Low |
| Sulfonylureas:control | 1 | Some concerns | Low risk | No concerns | Some concerns | Some concerns | Major concerns | Very low |
| Vitamin D:control | 4 | No concerns | Low risk | No concerns | Some concerns | Some concerns | Major concerns | Very low |
| alpha glucosidase inhibitor:control | 5 | No concerns | Low risk | No concerns | No concerns | Some concerns | Major concerns | Low |
| antihipertensive:control | 1 | No concerns | Low risk | No concerns | Major concerns | No concerns | Major concerns | Very low |
| control:diet | 2 | Some concerns | Low risk | No concerns | No concerns | No concerns | No concerns | High |
| control:enhanced education | 3 | Some concerns | Low risk | No concerns | Some concerns | Some concerns | No concerns | Low |
| control:intensive lifestyle intervention | 7 | Some concerns | Low risk | No concerns | No concerns | No concerns | No concerns | High |
| control:lifestyle intervention/education | 5 | Some concerns | Low risk | No concerns | No concerns | Some concerns | No concerns | Moderate |
| control:lipid low | 2 | Some concerns | Low risk | No concerns | Some concerns | Some concerns | Major concerns | Very low |
| control:physical activity | 3 | Some concerns | Low risk | No concerns | No concerns | No concerns | No concerns | High |
| control:thiazolidindione | 2 | Some concerns | Low risk | No concerns | No concerns | No concerns | Some concerns | Moderate |
| diet:intensive lifestyle intervention | 1 | Some concerns | Low risk | No concerns | Major concerns | No concerns | No concerns | Low |
| diet:physical activity | 1 | Some concerns | Low risk | No concerns | Major concerns | No concerns | No concerns | Low |
| enhanced education:lifestyle intervention/education | 5 | Some concerns | Low risk | No concerns | No concerns | Some concerns | No concerns | Moderate |
| intensive lifestyle intervention:lifestyle intervention/education | 8 | Some concerns | Low risk | No concerns | No concerns | Some concerns | No concerns | Moderate |
| intensive lifestyle intervention:physical activity | 1 | Some concerns | Low risk | No concerns | Major concerns | No concerns | No concerns | Low |
| intensive lifestyle intervention:thiazolidindione | 1 | Some concerns | Low risk | No concerns | Major concerns | No concerns | No concerns | Low |
| intensive lifestyle intervention:thiazolidindione +intensive lifestyle | 1 | Some concerns | Low risk | No concerns | Some concerns | Some concerns | No concerns | Low |
| lifestyle intervention/education:thiazolidindione | 2 | Some concerns | Low risk | No concerns | No concerns | Some concerns | Major concerns | Very low |
| lifestyle intervention/education:thiazolidindione +intensive lifestyle | 1 | Some concerns | Low risk | No concerns | No concerns | No concerns | No concerns | High |
| thiazolidindione:thiazolidindione +intensive lifestyle | 1 | Some concerns | Low risk | No concerns | Major concerns | No concerns | No concerns | Low |
| GLP1:GLP1+GIP | 0 | Some concerns | Low risk | No concerns | No concerns | No concerns | Major concerns | Low |
| GLP1:L-arginine | 0 | Some concerns | Low risk | No concerns | No concerns | No concerns | Major concerns | Low |
| GLP1:Metformin | 0 | Some concerns | Low risk | No concerns | No concerns | No concerns | Major concerns | Low |
| GLP1:Metformin + intensive lifestyle intervention | 0 | Some concerns | Low risk | No concerns | Major concerns | No concerns | Major concerns | Very low |
| GLP1:Sulfonylureas | 0 | Some concerns | Low risk | No concerns | No concerns | No concerns | Major concerns | Low |
| GLP1:Vitamin D | 0 | No concerns | Low risk | No concerns | No concerns | No concerns | Major concerns | Moderate |
| GLP1:alpha glucosidase inhibitor | 0 | No concerns | Low risk | No concerns | No concerns | No concerns | Major concerns | Moderate |
| GLP1:antihipertensive | 0 | No concerns | Low risk | No concerns | No concerns | No concerns | Major concerns | Moderate |
| GLP1:diet | 0 | Some concerns | Low risk | No concerns | No concerns | No concerns | Major concerns | Low |
| GLP1:enhanced education | 0 | Some concerns | Low risk | No concerns | No concerns | No concerns | Major concerns | Low |
| GLP1:intensive lifestyle intervention | 0 | Some concerns | Low risk | No concerns | No concerns | No concerns | Major concerns | Low |
| GLP1:lifestyle intervention/education | 0 | Some concerns | Low risk | No concerns | No concerns | No concerns | Major concerns | Low |
| GLP1:lipid low | 0 | No concerns | Low risk | No concerns | No concerns | No concerns | Major concerns | Moderate |
| GLP1:physical activity | 0 | Some concerns | Low risk | No concerns | No concerns | No concerns | Major concerns | Low |
| GLP1:thiazolidindione | 0 | Some concerns | Low risk | No concerns | No concerns | No concerns | Major concerns | Low |
| GLP1:thiazolidindione +intensive lifestyle | 0 | Some concerns | Low risk | No concerns | No concerns | Some concerns | Major concerns | Very low |
| GLP1+GIP:L-arginine | 0 | Some concerns | Low risk | No concerns | No concerns | No concerns | Major concerns | Low |
| GLP1+GIP:Metformin | 0 | Some concerns | Low risk | No concerns | No concerns | No concerns | Major concerns | Low |
| GLP1+GIP:Metformin + intensive lifestyle intervention | 0 | Some concerns | Low risk | No concerns | Some concerns | No concerns | Major concerns | Very low |
| GLP1+GIP:Sulfonylureas | 0 | Some concerns | Low risk | No concerns | No concerns | No concerns | Major concerns | Low |
| GLP1+GIP:Vitamin D | 0 | Some concerns | Low risk | No concerns | No concerns | No concerns | Major concerns | Low |
| GLP1+GIP:alpha glucosidase inhibitor | 0 | Some concerns | Low risk | No concerns | No concerns | No concerns | Major concerns | Low |
| GLP1+GIP:antihipertensive | 0 | Some concerns | Low risk | No concerns | No concerns | No concerns | Major concerns | Low |
| GLP1+GIP:diet | 0 | Some concerns | Low risk | No concerns | No concerns | No concerns | Major concerns | Low |
| GLP1+GIP:enhanced education | 0 | Some concerns | Low risk | No concerns | No concerns | No concerns | Major concerns | Low |
| GLP1+GIP:intensive lifestyle intervention | 0 | Some concerns | Low risk | No concerns | No concerns | No concerns | Major concerns | Low |
| GLP1+GIP:lifestyle intervention/education | 0 | Some concerns | Low risk | No concerns | No concerns | No concerns | Major concerns | Low |
| GLP1+GIP:lipid low | 0 | Some concerns | Low risk | No concerns | No concerns | No concerns | Major concerns | Low |
| GLP1+GIP:physical activity | 0 | Some concerns | Low risk | No concerns | No concerns | No concerns | Major concerns | Low |
| GLP1+GIP:thiazolidindione | 0 | Some concerns | Low risk | No concerns | No concerns | No concerns | Major concerns | Low |
| GLP1+GIP:thiazolidindione +intensive lifestyle | 0 | Some concerns | Low risk | No concerns | No concerns | No concerns | Major concerns | Low |
| L-arginine:Metformin | 0 | Some concerns | Low risk | No concerns | Major concerns | No concerns | Major concerns | Very low |
| L-arginine:Metformin + intensive lifestyle intervention | 0 | Some concerns | Low risk | No concerns | Major concerns | No concerns | Major concerns | Very low |
| L-arginine:Sulfonylureas | 0 | Some concerns | Low risk | No concerns | Major concerns | No concerns | Major concerns | Very low |
| L-arginine:Vitamin D | 0 | Some concerns | Low risk | No concerns | No concerns | Some concerns | Major concerns | Very low |
| L-arginine:alpha glucosidase inhibitor | 0 | Some concerns | Low risk | No concerns | Some concerns | Some concerns | Major concerns | Very low |
| L-arginine:antihipertensive | 0 | Some concerns | Low risk | No concerns | No concerns | Some concerns | Major concerns | Very low |
| L-arginine:diet | 0 | Some concerns | Low risk | No concerns | Major concerns | No concerns | Major concerns | Very low |
| L-arginine:enhanced education | 0 | Some concerns | Low risk | No concerns | No concerns | Some concerns | Major concerns | Very low |
| L-arginine:intensive lifestyle intervention | 0 | Some concerns | Low risk | No concerns | Major concerns | No concerns | Major concerns | Very low |
| L-arginine:lipid low | 0 | Some concerns | Low risk | No concerns | Some concerns | No concerns | Major concerns | Very low |
| L-arginine:physical activity | 0 | Some concerns | Low risk | No concerns | Major concerns | No concerns | Major concerns | Very low |
| L-arginine:thiazolidindione | 0 | Some concerns | Low risk | No concerns | Major concerns | No concerns | Major concerns | Very low |
| L-arginine:thiazolidindione +intensive lifestyle | 0 | Some concerns | Low risk | No concerns | Major concerns | No concerns | Major concerns | Very low |
| Metformin:Metformin + intensive lifestyle intervention | 0 | Some concerns | Low risk | No concerns | Some concerns | No concerns | Major concerns | Very low |
| Metformin:Sulfonylureas | 0 | Some concerns | Low risk | No concerns | Major concerns | No concerns | Major concerns | Very low |
| Metformin:Vitamin D | 0 | Some concerns | Low risk | No concerns | No concerns | Some concerns | Major concerns | Very low |
| Metformin:alpha glucosidase inhibitor | 0 | Some concerns | Low risk | No concerns | Major concerns | No concerns | Major concerns | Very low |
| Metformin:antihipertensive | 0 | Some concerns | Low risk | No concerns | Some concerns | No concerns | Major concerns | Very low |
| Metformin:diet | 0 | Some concerns | Low risk | No concerns | Major concerns | No concerns | Major concerns | Very low |
| Metformin:enhanced education | 0 | Some concerns | Low risk | No concerns | No concerns | Some concerns | Major concerns | Very low |
| Metformin:lipid low | 0 | Some concerns | Low risk | No concerns | Some concerns | Some concerns | Major concerns | Very low |
| Metformin:physical activity | 0 | Some concerns | Low risk | No concerns | Some concerns | Some concerns | Major concerns | Very low |
| Metformin:thiazolidindione | 0 | Some concerns | Low risk | No concerns | Some concerns | Some concerns | Major concerns | Very low |
| Metformin:thiazolidindione +intensive lifestyle | 0 | Some concerns | Low risk | No concerns | Some concerns | No concerns | Major concerns | Very low |
| Metformin + intensive lifestyle intervention:Sulfonylureas | 0 | Some concerns | Low risk | No concerns | No concerns | Some concerns | Major concerns | Very low |
| Metformin + intensive lifestyle intervention:Vitamin D | 0 | Some concerns | Low risk | No concerns | No concerns | No concerns | Major concerns | Low |
| Metformin + intensive lifestyle intervention:alpha glucosidase inhibitor | 0 | Some concerns | Low risk | No concerns | No concerns | No concerns | Major concerns | Low |
| Metformin + intensive lifestyle intervention:antihipertensive | 0 | Some concerns | Low risk | No concerns | No concerns | No concerns | Major concerns | Low |
| Metformin + intensive lifestyle intervention:diet | 0 | Some concerns | Low risk | No concerns | Some concerns | Some concerns | Major concerns | Very low |
| Metformin + intensive lifestyle intervention:enhanced education | 0 | Some concerns | Low risk | No concerns | No concerns | No concerns | Major concerns | Low |
| Metformin + intensive lifestyle intervention:intensive lifestyle intervention | 0 | Some concerns | Low risk | No concerns | Some concerns | Some concerns | Major concerns | Very low |
| Metformin + intensive lifestyle intervention:lifestyle intervention/education | 0 | Some concerns | Low risk | No concerns | No concerns | No concerns | Major concerns | Low |
| Metformin + intensive lifestyle intervention:lipid low | 0 | Some concerns | Low risk | No concerns | No concerns | No concerns | Major concerns | Low |
| Metformin + intensive lifestyle intervention:physical activity | 0 | Some concerns | Low risk | No concerns | Major concerns | No concerns | Major concerns | Very low |
| Metformin + intensive lifestyle intervention:thiazolidindione | 0 | Some concerns | Low risk | No concerns | Major concerns | No concerns | Major concerns | Very low |
| Metformin + intensive lifestyle intervention:thiazolidindione +intensive lifestyle | 0 | Some concerns | Low risk | No concerns | Major concerns | No concerns | Major concerns | Very low |
| Sulfonylureas:Vitamin D | 0 | Some concerns | Low risk | No concerns | Major concerns | No concerns | Major concerns | Very low |
| Sulfonylureas:alpha glucosidase inhibitor | 0 | Some concerns | Low risk | No concerns | Major concerns | No concerns | Major concerns | Very low |
| Sulfonylureas:antihipertensive | 0 | Some concerns | Low risk | No concerns | Major concerns | No concerns | Major concerns | Very low |
| Sulfonylureas:diet | 0 | Some concerns | Low risk | No concerns | Major concerns | No concerns | Major concerns | Very low |
| Sulfonylureas:enhanced education | 0 | Some concerns | Low risk | No concerns | Major concerns | No concerns | Major concerns | Very low |
| Sulfonylureas:intensive lifestyle intervention | 0 | Some concerns | Low risk | No concerns | Some concerns | Some concerns | Major concerns | Very low |
| Sulfonylureas:lifestyle intervention/education | 0 | Some concerns | Low risk | No concerns | Major concerns | No concerns | Major concerns | Very low |
| Sulfonylureas:lipid low | 0 | Some concerns | Low risk | No concerns | Major concerns | No concerns | Major concerns | Very low |
| Sulfonylureas:physical activity | 0 | Some concerns | Low risk | No concerns | Some concerns | Some concerns | Major concerns | Very low |
| Sulfonylureas:thiazolidindione | 0 | Some concerns | Low risk | No concerns | Some concerns | Some concerns | Major concerns | Very low |
| Sulfonylureas:thiazolidindione +intensive lifestyle | 0 | Some concerns | Low risk | No concerns | Some concerns | No concerns | Major concerns | Very low |
| Vitamin D:alpha glucosidase inhibitor | 0 | No concerns | Low risk | No concerns | Some concerns | Some concerns | Major concerns | Very low |
| Vitamin D:antihipertensive | 0 | No concerns | Low risk | No concerns | Major concerns | No concerns | Major concerns | Very low |
| Vitamin D:diet | 0 | Some concerns | Low risk | No concerns | No concerns | Some concerns | Major concerns | Very low |
| Vitamin D:enhanced education | 0 | Some concerns | Low risk | No concerns | Major concerns | No concerns | Major concerns | Very low |
| Vitamin D:intensive lifestyle intervention | 0 | Some concerns | Low risk | No concerns | No concerns | No concerns | Major concerns | Low |
| Vitamin D:lifestyle intervention/education | 0 | Some concerns | Low risk | No concerns | Some concerns | Some concerns | Major concerns | Very low |
| Vitamin D:lipid low | 0 | No concerns | Low risk | No concerns | Major concerns | No concerns | Major concerns | Very low |
| Vitamin D:physical activity | 0 | Some concerns | Low risk | No concerns | No concerns | No concerns | Major concerns | Low |
| Vitamin D:thiazolidindione | 0 | Some concerns | Low risk | No concerns | No concerns | No concerns | Major concerns | Low |
| Vitamin D:thiazolidindione +intensive lifestyle | 0 | Some concerns | Low risk | No concerns | No concerns | No concerns | Major concerns | Low |
| alpha glucosidase inhibitor:antihipertensive | 0 | No concerns | Low risk | No concerns | Some concerns | Some concerns | Major concerns | Very low |
| alpha glucosidase inhibitor:diet | 0 | Some concerns | Low risk | No concerns | Major concerns | No concerns | Major concerns | Very low |
| alpha glucosidase inhibitor:enhanced education | 0 | Some concerns | Low risk | No concerns | Some concerns | Some concerns | Major concerns | Very low |
| alpha glucosidase inhibitor:intensive lifestyle intervention | 0 | Some concerns | Low risk | No concerns | No concerns | Some concerns | Major concerns | Very low |
| alpha glucosidase inhibitor:lifestyle intervention/education | 0 | Some concerns | Low risk | No concerns | Major concerns | No concerns | Major concerns | Very low |
| alpha glucosidase inhibitor:lipid low | 0 | Some concerns | Low risk | No concerns | Some concerns | Some concerns | Major concerns | Very low |
| alpha glucosidase inhibitor:physical activity | 0 | Some concerns | Low risk | No concerns | Some concerns | No concerns | Major concerns | Very low |
| alpha glucosidase inhibitor:thiazolidindione | 0 | Some concerns | Low risk | No concerns | Some concerns | Some concerns | Major concerns | Very low |
| alpha glucosidase inhibitor:thiazolidindione +intensive lifestyle | 0 | Some concerns | Low risk | No concerns | No concerns | Some concerns | Major concerns | Very low |
| antihipertensive:diet | 0 | Some concerns | Low risk | No concerns | No concerns | Some concerns | Major concerns | Very low |
| antihipertensive:enhanced education | 0 | Some concerns | Low risk | No concerns | Major concerns | No concerns | Major concerns | Very low |
| antihipertensive:intensive lifestyle intervention | 0 | Some concerns | Low risk | No concerns | No concerns | No concerns | Major concerns | Low |
| antihipertensive:lifestyle intervention/education | 0 | Some concerns | Low risk | No concerns | Some concerns | Some concerns | Major concerns | Very low |
| antihipertensive:lipid low | 0 | No concerns | Low risk | No concerns | Major concerns | No concerns | Major concerns | Very low |
| antihipertensive:physical activity | 0 | Some concerns | Low risk | No concerns | No concerns | No concerns | Major concerns | Low |
| antihipertensive:thiazolidindione | 0 | Some concerns | Low risk | No concerns | No concerns | No concerns | Major concerns | Low |
| antihipertensive:thiazolidindione +intensive lifestyle | 0 | Some concerns | Low risk | No concerns | No concerns | No concerns | Major concerns | Low |
| control:thiazolidindione +intensive lifestyle | 0 | Some concerns | Low risk | No concerns | No concerns | No concerns | Major concerns | Low |
| diet:enhanced education | 0 | Some concerns | Low risk | No concerns | No concerns | Some concerns | Major concerns | Very low |
| diet:lifestyle intervention/education | 0 | Some concerns | Low risk | No concerns | Some concerns | Some concerns | Major concerns | Very low |
| diet:lipid low | 0 | Some concerns | Low risk | No concerns | Some concerns | No concerns | Major concerns | Very low |
| diet:thiazolidindione | 0 | Some concerns | Low risk | No concerns | Major concerns | No concerns | Major concerns | Very low |
| diet:thiazolidindione +intensive lifestyle | 0 | Some concerns | Low risk | No concerns | Major concerns | No concerns | Major concerns | Very low |
| enhanced education:intensive lifestyle intervention | 0 | Some concerns | Low risk | No concerns | No concerns | No concerns | Major concerns | Low |
| enhanced education:lipid low | 0 | Some concerns | Low risk | No concerns | Major concerns | No concerns | Major concerns | Very low |
| enhanced education:physical activity | 0 | Some concerns | Low risk | No concerns | No concerns | No concerns | Major concerns | Low |
| enhanced education:thiazolidindione | 0 | Some concerns | Low risk | No concerns | No concerns | No concerns | Major concerns | Low |
| enhanced education:thiazolidindione +intensive lifestyle | 0 | Some concerns | Low risk | No concerns | No concerns | No concerns | Major concerns | Low |
| intensive lifestyle intervention:lipid low | 0 | Some concerns | Low risk | No concerns | No concerns | Some concerns | Major concerns | Very low |
| lifestyle intervention/education:lipid low | 0 | Some concerns | Low risk | No concerns | Some concerns | Some concerns | Major concerns | Very low |
| lifestyle intervention/education:physical activity | 0 | Some concerns | Low risk | No concerns | No concerns | Some concerns | Major concerns | Very low |
| lipid low:physical activity | 0 | Some concerns | Low risk | No concerns | No concerns | Some concerns | Major concerns | Very low |
| lipid low:thiazolidindione | 0 | Some concerns | Low risk | No concerns | No concerns | Some concerns | Major concerns | Very low |
| lipid low:thiazolidindione +intensive lifestyle | 0 | Some concerns | Low risk | No concerns | No concerns | No concerns | Major concerns | Low |
| physical activity:thiazolidindione | 0 | Some concerns | Low risk | No concerns | Major concerns | No concerns | Major concerns | Very low |
| physical activity:thiazolidindione +intensive lifestyle | 0 | Some concerns | Low risk | No concerns | Major concerns | No concerns | Major concerns | Very low |

26. Table S8: Grading – HbA1c – 1 year (using CINeMA tool)

| Comparison | No of studies | Within-study bias | Reporting bias | Indirectness | Imprecision | Heterogeneity | Incoherence | Confidence rating |
| --- | --- | --- | --- | --- | --- | --- | --- | --- |
| AGI:control | 1 | Some concerns | Low risk | No concerns | No concerns | No concerns | Major concerns | Low |
| DPP4:Metformin | 1 | Some concerns | Low risk | No concerns | No concerns | Some concerns | No concerns | Moderate |
| DPP4:lifestyle_intervention_education | 1 | Some concerns | Low risk | No concerns | No concerns | Some concerns | No concerns | Moderate |
| GLP1:control | 3 | No concerns | Low risk | No concerns | No concerns | No concerns | Major concerns | Moderate |
| GLP1+GIP:control | 1 | Some concerns | Low risk | No concerns | No concerns | No concerns | Major concerns | Low |
| L-arginine + lifestyle intervention:lifestyle_intervention_education | 1 | Some concerns | Low risk | No concerns | Major concerns | No concerns | Major concerns | Very low |
| Metformin:Metformin + DPP4 | 1 | Some concerns | Low risk | No concerns | Some concerns | No concerns | Major concerns | Very low |
| Metformin:control | 1 | Some concerns | Low risk | No concerns | No concerns | Some concerns | No concerns | Moderate |
| Metformin:intensive lifestyle intervention | 2 | Some concerns | Low risk | No concerns | No concerns | No concerns | No concerns | High |
| Metformin:lifestyle_intervention_education | 2 | Some concerns | Low risk | No concerns | No concerns | No concerns | No concerns | High |
| Thiazolidindione:control | 1 | Some concerns | Low risk | No concerns | No concerns | Some concerns | Major concerns | Very low |
| Vitamin D:control | 5 | No concerns | Low risk | No concerns | No concerns | No concerns | Major concerns | Moderate |
| Zinc:control | 1 | No concerns | Low risk | No concerns | No concerns | Some concerns | Major concerns | Low |
| control:curcumin | 1 | No concerns | Low risk | No concerns | No concerns | No concerns | Major concerns | Moderate |
| control:diet | 4 | Some concerns | Low risk | No concerns | No concerns | Some concerns | Major concerns | Very low |
| control:enhanced_education | 4 | Some concerns | Low risk | No concerns | No concerns | No concerns | No concerns | High |
| control:intensive lifestyle intervention | 5 | Some concerns | Low risk | No concerns | No concerns | Some concerns | No concerns | Moderate |
| control:lifestyle_intervention_education | 2 | Some concerns | Low risk | No concerns | No concerns | No concerns | No concerns | High |
| control:physical_activity | 4 | Some concerns | Low risk | No concerns | No concerns | No concerns | Major concerns | Low |
| enhanced_education:lifestyle_intervention_education | 6 | Some concerns | Low risk | No concerns | No concerns | No concerns | No concerns | High |
| intensive lifestyle intervention:lifestyle_intervention_education | 7 | Some concerns | Low risk | No concerns | No concerns | No concerns | No concerns | High |
| AGI:DPP4 | 0 | Some concerns | Low risk | No concerns | No concerns | Some concerns | Major concerns | Very low |
| AGI:GLP1 | 0 | Some concerns | Low risk | No concerns | No concerns | No concerns | Major concerns | Low |
| AGI:GLP1+GIP | 0 | Some concerns | Low risk | No concerns | No concerns | No concerns | Major concerns | Low |
| AGI:L-arginine + lifestyle intervention | 0 | Some concerns | Low risk | No concerns | Major concerns | No concerns | Major concerns | Very low |
| AGI:Metformin | 0 | Some concerns | Low risk | No concerns | Some concerns | No concerns | Major concerns | Very low |
| AGI:Metformin + DPP4 | 0 | Some concerns | Low risk | No concerns | No concerns | Some concerns | Major concerns | Very low |
| AGI:Thiazolidindione | 0 | Some concerns | Low risk | No concerns | Some concerns | No concerns | Major concerns | Very low |
| AGI:Vitamin D | 0 | Some concerns | Low risk | No concerns | No concerns | Some concerns | Major concerns | Very low |
| AGI:Zinc | 0 | Some concerns | Low risk | No concerns | Some concerns | No concerns | Major concerns | Very low |
| AGI:curcumin | 0 | Some concerns | Low risk | No concerns | No concerns | No concerns | Major concerns | Low |
| AGI:diet | 0 | Some concerns | Low risk | No concerns | No concerns | Some concerns | Major concerns | Very low |
| AGI:enhanced_education | 0 | Some concerns | Low risk | No concerns | No concerns | No concerns | Major concerns | Low |
| AGI:intensive lifestyle intervention | 0 | Some concerns | Low risk | No concerns | Some concerns | No concerns | Major concerns | Very low |
| AGI:lifestyle_intervention_education | 0 | Some concerns | Low risk | No concerns | No concerns | Some concerns | Major concerns | Very low |
| AGI:physical_activity | 0 | Some concerns | Low risk | No concerns | No concerns | No concerns | Major concerns | Low |
| DPP4:GLP1 | 0 | Some concerns | Low risk | No concerns | No concerns | No concerns | Major concerns | Low |
| DPP4:GLP1+GIP | 0 | Some concerns | Low risk | No concerns | No concerns | No concerns | Major concerns | Low |
| DPP4:L-arginine + lifestyle intervention | 0 | Some concerns | Low risk | No concerns | Major concerns | No concerns | Major concerns | Very low |
| DPP4:Metformin + DPP4 | 0 | Some concerns | Low risk | No concerns | No concerns | Some concerns | Major concerns | Very low |
| DPP4:Thiazolidindione | 0 | Some concerns | Low risk | No concerns | Some concerns | No concerns | Major concerns | Very low |
| DPP4:Vitamin D | 0 | Some concerns | Low risk | No concerns | No concerns | Some concerns | Major concerns | Very low |
| DPP4:Zinc | 0 | Some concerns | Low risk | No concerns | Some concerns | No concerns | Major concerns | Very low |
| DPP4:control | 0 | Some concerns | Low risk | No concerns | No concerns | No concerns | Major concerns | Low |
| DPP4:curcumin | 0 | Some concerns | Low risk | No concerns | No concerns | No concerns | Major concerns | Low |
| DPP4:diet | 0 | Some concerns | Low risk | No concerns | Some concerns | No concerns | Major concerns | Very low |
| DPP4:enhanced_education | 0 | Some concerns | Low risk | No concerns | No concerns | No concerns | Major concerns | Low |
| DPP4:intensive lifestyle intervention | 0 | Some concerns | Low risk | No concerns | Some concerns | No concerns | Major concerns | Very low |
| DPP4:physical_activity | 0 | Some concerns | Low risk | No concerns | No concerns | No concerns | Major concerns | Low |
| GLP1:GLP1+GIP | 0 | Some concerns | Low risk | No concerns | No concerns | Some concerns | Major concerns | Very low |
| GLP1:L-arginine + lifestyle intervention | 0 | Some concerns | Low risk | No concerns | Some concerns | Some concerns | Major concerns | Very low |
| GLP1:Metformin | 0 | Some concerns | Low risk | No concerns | No concerns | No concerns | Major concerns | Low |
| GLP1:Metformin + DPP4 | 0 | Some concerns | Low risk | No concerns | Some concerns | No concerns | Major concerns | Very low |
| GLP1:Thiazolidindione | 0 | Some concerns | Low risk | No concerns | No concerns | No concerns | Major concerns | Low |
| GLP1:Vitamin D | 0 | No concerns | Low risk | No concerns | No concerns | No concerns | Major concerns | Moderate |
| GLP1:Zinc | 0 | No concerns | Low risk | No concerns | No concerns | No concerns | Major concerns | Moderate |
| GLP1:curcumin | 0 | No concerns | Low risk | No concerns | Some concerns | No concerns | Major concerns | Low |
| GLP1:diet | 0 | No concerns | Low risk | No concerns | No concerns | No concerns | Major concerns | Moderate |
| GLP1:enhanced_education | 0 | No concerns | Low risk | No concerns | No concerns | No concerns | Major concerns | Moderate |
| GLP1:intensive lifestyle intervention | 0 | Some concerns | Low risk | No concerns | No concerns | No concerns | Major concerns | Low |
| GLP1:lifestyle_intervention_education | 0 | No concerns | Low risk | No concerns | No concerns | No concerns | Major concerns | Moderate |
| GLP1:physical_activity | 0 | Some concerns | Low risk | No concerns | No concerns | No concerns | Major concerns | Low |
| GLP1+GIP:L-arginine + lifestyle intervention | 0 | Some concerns | Low risk | No concerns | Some concerns | No concerns | Major concerns | Very low |
| GLP1+GIP:Metformin | 0 | Some concerns | Low risk | No concerns | No concerns | No concerns | Major concerns | Low |
| GLP1+GIP:Metformin + DPP4 | 0 | Some concerns | Low risk | No concerns | Some concerns | No concerns | Major concerns | Very low |
| GLP1+GIP:Thiazolidindione | 0 | Some concerns | Low risk | No concerns | No concerns | No concerns | Major concerns | Low |
| GLP1+GIP:Vitamin D | 0 | Some concerns | Low risk | No concerns | No concerns | No concerns | Major concerns | Low |
| GLP1+GIP:Zinc | 0 | Some concerns | Low risk | No concerns | No concerns | No concerns | Major concerns | Low |
| GLP1+GIP:curcumin | 0 | Some concerns | Low risk | No concerns | No concerns | Some concerns | Major concerns | Very low |
| GLP1+GIP:diet | 0 | Some concerns | Low risk | No concerns | No concerns | No concerns | Major concerns | Low |
| GLP1+GIP:enhanced_education | 0 | Some concerns | Low risk | No concerns | No concerns | No concerns | Major concerns | Low |
| GLP1+GIP:intensive lifestyle intervention | 0 | Some concerns | Low risk | No concerns | No concerns | No concerns | Major concerns | Low |
| GLP1+GIP:lifestyle_intervention_education | 0 | Some concerns | Low risk | No concerns | No concerns | No concerns | Major concerns | Low |
| GLP1+GIP:physical_activity | 0 | Some concerns | Low risk | No concerns | Some concerns | No concerns | Major concerns | Very low |
| L-arginine + lifestyle intervention:Metformin | 0 | Some concerns | Low risk | No concerns | Major concerns | No concerns | Major concerns | Very low |
| L-arginine + lifestyle intervention:Metformin + DPP4 | 0 | Some concerns | Low risk | No concerns | Major concerns | No concerns | Major concerns | Very low |
| L-arginine + lifestyle intervention:Thiazolidindione | 0 | Some concerns | Low risk | No concerns | Major concerns | No concerns | Major concerns | Very low |
| L-arginine + lifestyle intervention:Vitamin D | 0 | Some concerns | Low risk | No concerns | Major concerns | No concerns | Major concerns | Very low |
| L-arginine + lifestyle intervention:Zinc | 0 | Some concerns | Low risk | No concerns | Major concerns | No concerns | Major concerns | Very low |
| L-arginine + lifestyle intervention:control | 0 | Some concerns | Low risk | No concerns | Major concerns | No concerns | Major concerns | Very low |
| L-arginine + lifestyle intervention:curcumin | 0 | Some concerns | Low risk | No concerns | Some concerns | No concerns | Major concerns | Very low |
| L-arginine + lifestyle intervention:diet | 0 | Some concerns | Low risk | No concerns | Major concerns | No concerns | Major concerns | Very low |
| L-arginine + lifestyle intervention:enhanced_education | 0 | Some concerns | Low risk | No concerns | Major concerns | No concerns | Major concerns | Very low |
| L-arginine + lifestyle intervention:intensive lifestyle intervention | 0 | Some concerns | Low risk | No concerns | Major concerns | No concerns | Major concerns | Very low |
| L-arginine + lifestyle intervention:physical_activity | 0 | Some concerns | Low risk | No concerns | Major concerns | No concerns | Major concerns | Very low |
| Metformin:Thiazolidindione | 0 | Some concerns | Low risk | No concerns | No concerns | No concerns | Major concerns | Low |
| Metformin:Vitamin D | 0 | Some concerns | Low risk | No concerns | No concerns | No concerns | Major concerns | Low |
| Metformin:Zinc | 0 | Some concerns | Low risk | No concerns | No concerns | No concerns | Major concerns | Low |
| Metformin:curcumin | 0 | Some concerns | Low risk | No concerns | No concerns | No concerns | Major concerns | Low |
| Metformin:diet | 0 | Some concerns | Low risk | No concerns | No concerns | No concerns | Major concerns | Low |
| Metformin:enhanced_education | 0 | Some concerns | Low risk | No concerns | No concerns | No concerns | Major concerns | Low |
| Metformin:physical_activity | 0 | Some concerns | Low risk | No concerns | No concerns | No concerns | Major concerns | Low |
| Metformin + DPP4:Thiazolidindione | 0 | Some concerns | Low risk | No concerns | Some concerns | No concerns | Major concerns | Very low |
| Metformin + DPP4:Vitamin D | 0 | Some concerns | Low risk | No concerns | Some concerns | No concerns | Major concerns | Very low |
| Metformin + DPP4:Zinc | 0 | Some concerns | Low risk | No concerns | Some concerns | No concerns | Major concerns | Very low |
| Metformin + DPP4:control | 0 | Some concerns | Low risk | No concerns | No concerns | Some concerns | Major concerns | Very low |
| Metformin + DPP4:curcumin | 0 | Some concerns | Low risk | No concerns | Some concerns | No concerns | Major concerns | Very low |
| Metformin + DPP4:diet | 0 | Some concerns | Low risk | No concerns | Some concerns | No concerns | Major concerns | Very low |
| Metformin + DPP4:enhanced_education | 0 | Some concerns | Low risk | No concerns | No concerns | Some concerns | Major concerns | Very low |
| Metformin + DPP4:intensive lifestyle intervention | 0 | Some concerns | Low risk | No concerns | Some concerns | No concerns | Major concerns | Very low |
| Metformin + DPP4:lifestyle_intervention_education | 0 | Some concerns | Low risk | No concerns | Some concerns | No concerns | Major concerns | Very low |
| Metformin + DPP4:physical_activity | 0 | Some concerns | Low risk | No concerns | Some concerns | No concerns | Major concerns | Very low |
| Thiazolidindione:Vitamin D | 0 | Some concerns | Low risk | No concerns | No concerns | Some concerns | Major concerns | Very low |
| Thiazolidindione:Zinc | 0 | Some concerns | Low risk | No concerns | No concerns | Major concerns | Major concerns | Very low |
| Thiazolidindione:curcumin | 0 | Some concerns | Low risk | No concerns | No concerns | No concerns | Major concerns | Low |
| Thiazolidindione:diet | 0 | Some concerns | Low risk | No concerns | No concerns | No concerns | Major concerns | Low |
| Thiazolidindione:enhanced_education | 0 | Some concerns | Low risk | No concerns | No concerns | Some concerns | Major concerns | Very low |
| Thiazolidindione:intensive lifestyle intervention | 0 | Some concerns | Low risk | No concerns | No concerns | Some concerns | Major concerns | Very low |
| Thiazolidindione:lifestyle_intervention_education | 0 | Some concerns | Low risk | No concerns | No concerns | No concerns | Major concerns | Low |
| Thiazolidindione:physical_activity | 0 | Some concerns | Low risk | No concerns | No concerns | Some concerns | Major concerns | Very low |
| Vitamin D:Zinc | 0 | No concerns | Low risk | No concerns | No concerns | Some concerns | Major concerns | Low |
| Vitamin D:curcumin | 0 | No concerns | Low risk | No concerns | No concerns | No concerns | Major concerns | Moderate |
| Vitamin D:diet | 0 | No concerns | Low risk | No concerns | No concerns | No concerns | Major concerns | Moderate |
| Vitamin D:enhanced_education | 0 | Some concerns | Low risk | No concerns | No concerns | No concerns | Major concerns | Low |
| Vitamin D:intensive lifestyle intervention | 0 | Some concerns | Low risk | No concerns | No concerns | Some concerns | Major concerns | Very low |
| Vitamin D:lifestyle_intervention_education | 0 | Some concerns | Low risk | No concerns | No concerns | No concerns | Major concerns | Low |
| Vitamin D:physical_activity | 0 | Some concerns | Low risk | No concerns | No concerns | No concerns | Major concerns | Low |
| Zinc:curcumin | 0 | No concerns | Low risk | No concerns | No concerns | No concerns | Major concerns | Moderate |
| Zinc:diet | 0 | No concerns | Low risk | No concerns | No concerns | No concerns | Major concerns | Moderate |
| Zinc:enhanced_education | 0 | No concerns | Low risk | No concerns | No concerns | Some concerns | Major concerns | Low |
| Zinc:intensive lifestyle intervention | 0 | Some concerns | Low risk | No concerns | No concerns | Some concerns | Major concerns | Very low |
| Zinc:lifestyle_intervention_education | 0 | No concerns | Low risk | No concerns | No concerns | No concerns | Major concerns | Moderate |
| Zinc:physical_activity | 0 | Some concerns | Low risk | No concerns | No concerns | Some concerns | Major concerns | Very low |
| curcumin:diet | 0 | No concerns | Low risk | No concerns | No concerns | No concerns | Major concerns | Moderate |
| curcumin:enhanced_education | 0 | No concerns | Low risk | No concerns | No concerns | No concerns | Major concerns | Moderate |
| curcumin:intensive lifestyle intervention | 0 | Some concerns | Low risk | No concerns | No concerns | No concerns | Major concerns | Low |
| curcumin:lifestyle_intervention_education | 0 | No concerns | Low risk | No concerns | No concerns | No concerns | Major concerns | Moderate |
| curcumin:physical_activity | 0 | Some concerns | Low risk | No concerns | Some concerns | No concerns | Major concerns | Very low |
| diet:enhanced_education | 0 | Some concerns | Low risk | No concerns | No concerns | No concerns | Major concerns | Low |
| diet:intensive lifestyle intervention | 0 | Some concerns | Low risk | No concerns | No concerns | No concerns | Major concerns | Low |
| diet:lifestyle_intervention_education | 0 | Some concerns | Low risk | No concerns | No concerns | No concerns | Major concerns | Low |
| diet:physical_activity | 0 | Some concerns | Low risk | No concerns | No concerns | No concerns | Major concerns | Low |
| enhanced_education:intensive lifestyle intervention | 0 | Some concerns | Low risk | No concerns | No concerns | Some concerns | Major concerns | Very low |
| enhanced_education:physical_activity | 0 | Some concerns | Low risk | No concerns | No concerns | No concerns | Major concerns | Low |
| intensive lifestyle intervention:physical_activity | 0 | Some concerns | Low risk | No concerns | No concerns | Some concerns | Major concerns | Very low |
| lifestyle_intervention_education:physical_activity | 0 | Some concerns | Low risk | No concerns | No concerns | No concerns | Major concerns | Low |

27. Table S9: Grading– HbA1c – 6 months (using CINeMA tool)

| Comparison | No of studies | Within-study bias | Reporting bias | Indirectness | Imprecision | Heterogeneity | Incoherence | Confidence rating |
| --- | --- | --- | --- | --- | --- | --- | --- | --- |
| Antihipertensive:control | 2 | No concerns | Low risk | No concerns | Some concerns | No concerns | Major concerns | Low |
| DPP4:Metformin | 1 | Some concerns | Low risk | No concerns | Some concerns | Some concerns | No concerns | Low |
| DPP4:control | 1 | Some concerns | Low risk | No concerns | No concerns | Some concerns | No concerns | Moderate |
| Diet:intensive lifestyle intervention | 1 | Some concerns | Low risk | No concerns | No concerns | No concerns | Major concerns | Low |
| GLP1:control | 1 | No concerns | Low risk | No concerns | No concerns | No concerns | Major concerns | Moderate |
| GLP1+GIP:control | 1 | Some concerns | Low risk | No concerns | No concerns | No concerns | Major concerns | Low |
| Metformin:Metformin + DPP4 | 1 | Some concerns | Low risk | No concerns | Some concerns | No concerns | Major concerns | Very low |
| Metformin:control | 3 | Some concerns | Low risk | No concerns | No concerns | Some concerns | No concerns | Moderate |
| Metformin:intensive lifestyle intervention | 1 | Some concerns | Low risk | No concerns | Some concerns | No concerns | No concerns | Moderate |
| Metformin:lifestyle_intervention_education | 1 | Some concerns | Low risk | No concerns | No concerns | Some concerns | No concerns | Moderate |
| SGLT2:control | 1 | Some concerns | Low risk | No concerns | No concerns | Some concerns | Major concerns | Very low |
| Vitamin D:control | 3 | No concerns | Low risk | No concerns | No concerns | Some concerns | Major concerns | Low |
| Zinc:control | 1 | No concerns | Low risk | No concerns | No concerns | Major concerns | Major concerns | Very low |
| control:curcumin | 1 | No concerns | Low risk | No concerns | No concerns | Some concerns | Major concerns | Low |
| control:diet | 2 | Some concerns | Low risk | No concerns | No concerns | Some concerns | Major concerns | Very low |
| control:enhanced_education | 7 | Some concerns | Low risk | No concerns | No concerns | Some concerns | No concerns | Moderate |
| control:intensive lifestyle intervention | 1 | Some concerns | Low risk | No concerns | No concerns | Some concerns | No concerns | Moderate |
| control:lifestyle_intervention_education | 4 | Some concerns | Low risk | No concerns | No concerns | Some concerns | No concerns | Moderate |
| control:physical_activity | 6 | Some concerns | Low risk | No concerns | No concerns | Some concerns | Major concerns | Very low |
| control:pro_pre_symbiotic | 1 | No concerns | Low risk | No concerns | Some concerns | No concerns | Major concerns | Low |
| enhanced_education:lifestyle_intervention_education | 5 | Some concerns | Low risk | No concerns | No concerns | No concerns | No concerns | High |
| intensive lifestyle intervention:lifestyle_intervention_education | 4 | Some concerns | Low risk | No concerns | No concerns | Some concerns | Some concerns | Low |
| Antihipertensive:DPP4 | 0 | Some concerns | Low risk | No concerns | Some concerns | No concerns | Major concerns | Very low |
| Antihipertensive:Diet | 0 | Some concerns | Low risk | No concerns | No concerns | No concerns | Major concerns | Low |
| Antihipertensive:GLP1 | 0 | No concerns | Low risk | No concerns | Some concerns | No concerns | Major concerns | Low |
| Antihipertensive:GLP1+GIP | 0 | Some concerns | Low risk | No concerns | No concerns | Some concerns | Major concerns | Very low |
| Antihipertensive:Metformin | 0 | Some concerns | Low risk | No concerns | Some concerns | No concerns | Major concerns | Very low |
| Antihipertensive:Metformin + DPP4 | 0 | Some concerns | Low risk | No concerns | Some concerns | No concerns | Major concerns | Very low |
| Antihipertensive:SGLT2 | 0 | Some concerns | Low risk | No concerns | Some concerns | No concerns | Major concerns | Very low |
| Antihipertensive:Vitamin D | 0 | No concerns | Low risk | No concerns | Some concerns | No concerns | Major concerns | Low |
| Antihipertensive:Zinc | 0 | No concerns | Low risk | No concerns | Some concerns | No concerns | Major concerns | Low |
| Antihipertensive:curcumin | 0 | No concerns | Low risk | No concerns | Some concerns | No concerns | Major concerns | Low |
| Antihipertensive:diet | 0 | Some concerns | Low risk | No concerns | Some concerns | No concerns | Major concerns | Very low |
| Antihipertensive:enhanced_education | 0 | Some concerns | Low risk | No concerns | Some concerns | No concerns | Major concerns | Very low |
| Antihipertensive:intensive lifestyle intervention | 0 | Some concerns | Low risk | No concerns | No concerns | Major concerns | Major concerns | Very low |
| Antihipertensive:lifestyle_intervention_education | 0 | Some concerns | Low risk | No concerns | No concerns | Some concerns | Major concerns | Very low |
| Antihipertensive:physical_activity | 0 | Some concerns | Low risk | No concerns | No concerns | Some concerns | Major concerns | Very low |
| Antihipertensive:pro_pre_symbiotic | 0 | No concerns | Low risk | No concerns | Major concerns | No concerns | Major concerns | Very low |
| DPP4:Diet | 0 | Some concerns | Low risk | No concerns | No concerns | No concerns | Major concerns | Low |
| DPP4:GLP1 | 0 | Some concerns | Low risk | No concerns | Major concerns | No concerns | Major concerns | Very low |
| DPP4:GLP1+GIP | 0 | Some concerns | Low risk | No concerns | Some concerns | Some concerns | Major concerns | Very low |
| DPP4:Metformin + DPP4 | 0 | Some concerns | Low risk | No concerns | Some concerns | Some concerns | Major concerns | Very low |
| DPP4:SGLT2 | 0 | Some concerns | Low risk | No concerns | Some concerns | No concerns | Major concerns | Very low |
| DPP4:Vitamin D | 0 | Some concerns | Low risk | No concerns | No concerns | Some concerns | Major concerns | Very low |
| DPP4:Zinc | 0 | Some concerns | Low risk | No concerns | Some concerns | No concerns | Major concerns | Very low |
| DPP4:curcumin | 0 | Some concerns | Low risk | No concerns | Major concerns | No concerns | Major concerns | Very low |
| DPP4:diet | 0 | Some concerns | Low risk | No concerns | Major concerns | No concerns | Major concerns | Very low |
| DPP4:enhanced_education | 0 | Some concerns | Low risk | No concerns | Some concerns | No concerns | Major concerns | Very low |
| DPP4:intensive lifestyle intervention | 0 | Some concerns | Low risk | No concerns | Some concerns | No concerns | Major concerns | Very low |
| DPP4:lifestyle_intervention_education | 0 | Some concerns | Low risk | No concerns | Some concerns | No concerns | Major concerns | Very low |
| DPP4:physical_activity | 0 | Some concerns | Low risk | No concerns | Some concerns | No concerns | Major concerns | Very low |
| DPP4:pro_pre_symbiotic | 0 | Some concerns | Low risk | No concerns | Some concerns | Some concerns | Major concerns | Very low |
| Diet:GLP1 | 0 | Some concerns | Low risk | No concerns | No concerns | No concerns | Major concerns | Low |
| Diet:GLP1+GIP | 0 | Some concerns | Low risk | No concerns | No concerns | No concerns | Major concerns | Low |
| Diet:Metformin | 0 | Some concerns | Low risk | No concerns | No concerns | No concerns | Major concerns | Low |
| Diet:Metformin + DPP4 | 0 | Some concerns | Low risk | No concerns | No concerns | No concerns | Major concerns | Low |
| Diet:SGLT2 | 0 | Some concerns | Low risk | No concerns | No concerns | Some concerns | Major concerns | Very low |
| Diet:Vitamin D | 0 | Some concerns | Low risk | No concerns | No concerns | Some concerns | Major concerns | Very low |
| Diet:Zinc | 0 | Some concerns | Low risk | No concerns | No concerns | Some concerns | Major concerns | Very low |
| Diet:control | 0 | Some concerns | Low risk | No concerns | No concerns | No concerns | Major concerns | Low |
| Diet:curcumin | 0 | Some concerns | Low risk | No concerns | No concerns | No concerns | Major concerns | Low |
| Diet:diet | 0 | Some concerns | Low risk | No concerns | No concerns | No concerns | Major concerns | Low |
| Diet:enhanced_education | 0 | Some concerns | Low risk | No concerns | No concerns | No concerns | Major concerns | Low |
| Diet:lifestyle_intervention_education | 0 | Some concerns | Low risk | No concerns | No concerns | No concerns | Major concerns | Low |
| Diet:physical_activity | 0 | Some concerns | Low risk | No concerns | No concerns | No concerns | Major concerns | Low |
| Diet:pro_pre_symbiotic | 0 | Some concerns | Low risk | No concerns | No concerns | No concerns | Major concerns | Low |
| GLP1:GLP1+GIP | 0 | Some concerns | Low risk | No concerns | Some concerns | No concerns | Major concerns | Very low |
| GLP1:Metformin | 0 | Some concerns | Low risk | No concerns | Some concerns | Some concerns | Major concerns | Very low |
| GLP1:Metformin + DPP4 | 0 | Some concerns | Low risk | No concerns | Some concerns | Some concerns | Major concerns | Very low |
| GLP1:SGLT2 | 0 | Some concerns | Low risk | No concerns | Some concerns | No concerns | Major concerns | Very low |
| GLP1:Vitamin D | 0 | No concerns | Low risk | No concerns | No concerns | No concerns | Major concerns | Moderate |
| GLP1:Zinc | 0 | No concerns | Low risk | No concerns | No concerns | Some concerns | Major concerns | Low |
| GLP1:curcumin | 0 | No concerns | Low risk | No concerns | Major concerns | No concerns | Major concerns | Very low |
| GLP1:diet | 0 | Some concerns | Low risk | No concerns | Some concerns | Some concerns | Major concerns | Very low |
| GLP1:enhanced_education | 0 | Some concerns | Low risk | No concerns | No concerns | Some concerns | Major concerns | Very low |
| GLP1:intensive lifestyle intervention | 0 | Some concerns | Low risk | No concerns | Some concerns | No concerns | Major concerns | Very low |
| GLP1:lifestyle_intervention_education | 0 | No concerns | Low risk | No concerns | No concerns | Some concerns | Major concerns | Low |
| GLP1:physical_activity | 0 | No concerns | Low risk | No concerns | Some concerns | No concerns | Major concerns | Low |
| GLP1:pro_pre_symbiotic | 0 | No concerns | Low risk | No concerns | Some concerns | No concerns | Major concerns | Low |
| GLP1+GIP:Metformin | 0 | Some concerns | Low risk | No concerns | Some concerns | No concerns | Major concerns | Very low |
| GLP1+GIP:Metformin + DPP4 | 0 | Some concerns | Low risk | No concerns | Major concerns | No concerns | Major concerns | Very low |
| GLP1+GIP:SGLT2 | 0 | Some concerns | Low risk | No concerns | No concerns | No concerns | Major concerns | Low |
| GLP1+GIP:Vitamin D | 0 | Some concerns | Low risk | No concerns | No concerns | No concerns | Major concerns | Low |
| GLP1+GIP:Zinc | 0 | Some concerns | Low risk | No concerns | No concerns | No concerns | Major concerns | Low |
| GLP1+GIP:curcumin | 0 | Some concerns | Low risk | No concerns | Some concerns | Some concerns | Major concerns | Very low |
| GLP1+GIP:diet | 0 | Some concerns | Low risk | No concerns | Some concerns | No concerns | Major concerns | Very low |
| GLP1+GIP:enhanced_education | 0 | Some concerns | Low risk | No concerns | No concerns | No concerns | Major concerns | Low |
| GLP1+GIP:intensive lifestyle intervention | 0 | Some concerns | Low risk | No concerns | No concerns | Some concerns | Major concerns | Very low |
| GLP1+GIP:lifestyle_intervention_education | 0 | Some concerns | Low risk | No concerns | No concerns | No concerns | Major concerns | Low |
| GLP1+GIP:physical_activity | 0 | Some concerns | Low risk | No concerns | Some concerns | No concerns | Major concerns | Very low |
| GLP1+GIP:pro_pre_symbiotic | 0 | Some concerns | Low risk | No concerns | Some concerns | No concerns | Major concerns | Very low |
| Metformin:SGLT2 | 0 | Some concerns | Low risk | No concerns | Some concerns | No concerns | Major concerns | Very low |
| Metformin:Vitamin D | 0 | Some concerns | Low risk | No concerns | No concerns | Some concerns | Major concerns | Very low |
| Metformin:Zinc | 0 | Some concerns | Low risk | No concerns | Some concerns | No concerns | Major concerns | Very low |
| Metformin:curcumin | 0 | Some concerns | Low risk | No concerns | Some concerns | Some concerns | Major concerns | Very low |
| Metformin:diet | 0 | Some concerns | Low risk | No concerns | Some concerns | Some concerns | Major concerns | Very low |
| Metformin:enhanced_education | 0 | Some concerns | Low risk | No concerns | No concerns | Some concerns | Major concerns | Very low |
| Metformin:physical_activity | 0 | Some concerns | Low risk | No concerns | No concerns | Some concerns | Major concerns | Very low |
| Metformin:pro_pre_symbiotic | 0 | Some concerns | Low risk | No concerns | Some concerns | Some concerns | Major concerns | Very low |
| Metformin + DPP4:SGLT2 | 0 | Some concerns | Low risk | No concerns | No concerns | Some concerns | Major concerns | Very low |
| Metformin + DPP4:Vitamin D | 0 | Some concerns | Low risk | No concerns | No concerns | No concerns | Major concerns | Low |
| Metformin + DPP4:Zinc | 0 | Some concerns | Low risk | No concerns | No concerns | No concerns | Major concerns | Low |
| Metformin + DPP4:control | 0 | Some concerns | Low risk | No concerns | No concerns | No concerns | Major concerns | Low |
| Metformin + DPP4:curcumin | 0 | Some concerns | Low risk | No concerns | Some concerns | Some concerns | Major concerns | Very low |
| Metformin + DPP4:diet | 0 | Some concerns | Low risk | No concerns | Some concerns | Some concerns | Major concerns | Very low |
| Metformin + DPP4:enhanced_education | 0 | Some concerns | Low risk | No concerns | No concerns | No concerns | Major concerns | Low |
| Metformin + DPP4:intensive lifestyle intervention | 0 | Some concerns | Low risk | No concerns | No concerns | Some concerns | Major concerns | Very low |
| Metformin + DPP4:lifestyle_intervention_education | 0 | Some concerns | Low risk | No concerns | No concerns | Some concerns | Major concerns | Very low |
| Metformin + DPP4:physical_activity | 0 | Some concerns | Low risk | No concerns | Some concerns | No concerns | Major concerns | Very low |
| Metformin + DPP4:pro_pre_symbiotic | 0 | Some concerns | Low risk | No concerns | Some concerns | No concerns | Major concerns | Very low |
| SGLT2:Vitamin D | 0 | Some concerns | Low risk | No concerns | Some concerns | Some concerns | Major concerns | Very low |
| SGLT2:Zinc | 0 | Some concerns | Low risk | No concerns | Some concerns | Some concerns | Major concerns | Very low |
| SGLT2:curcumin | 0 | Some concerns | Low risk | No concerns | Some concerns | No concerns | Major concerns | Very low |
| SGLT2:diet | 0 | Some concerns | Low risk | No concerns | Some concerns | No concerns | Major concerns | Very low |
| SGLT2:enhanced_education | 0 | Some concerns | Low risk | No concerns | No concerns | Major concerns | Major concerns | Very low |
| SGLT2:intensive lifestyle intervention | 0 | Some concerns | Low risk | No concerns | Some concerns | No concerns | Major concerns | Very low |
| SGLT2:lifestyle_intervention_education | 0 | Some concerns | Low risk | No concerns | No concerns | Some concerns | Major concerns | Very low |
| SGLT2:physical_activity | 0 | Some concerns | Low risk | No concerns | Some concerns | No concerns | Major concerns | Very low |
| SGLT2:pro_pre_symbiotic | 0 | Some concerns | Low risk | No concerns | Some concerns | No concerns | Major concerns | Very low |
| Vitamin D:Zinc | 0 | No concerns | Low risk | No concerns | Some concerns | Some concerns | Major concerns | Very low |
| Vitamin D:curcumin | 0 | No concerns | Low risk | No concerns | No concerns | Some concerns | Major concerns | Low |
| Vitamin D:diet | 0 | Some concerns | Low risk | No concerns | No concerns | Some concerns | Major concerns | Very low |
| Vitamin D:enhanced_education | 0 | Some concerns | Low risk | No concerns | No concerns | Some concerns | Major concerns | Very low |
| Vitamin D:intensive lifestyle intervention | 0 | Some concerns | Low risk | No concerns | Some concerns | No concerns | Major concerns | Very low |
| Vitamin D:lifestyle_intervention_education | 0 | Some concerns | Low risk | No concerns | No concerns | Some concerns | Major concerns | Very low |
| Vitamin D:physical_activity | 0 | Some concerns | Low risk | No concerns | No concerns | Some concerns | Major concerns | Very low |
| Vitamin D:pro_pre_symbiotic | 0 | No concerns | Low risk | No concerns | Some concerns | No concerns | Major concerns | Low |
| Zinc:curcumin | 0 | No concerns | Low risk | No concerns | Some concerns | No concerns | Major concerns | Low |
| Zinc:diet | 0 | Some concerns | Low risk | No concerns | Some concerns | No concerns | Major concerns | Very low |
| Zinc:enhanced_education | 0 | Some concerns | Low risk | No concerns | Some concerns | No concerns | Major concerns | Very low |
| Zinc:intensive lifestyle intervention | 0 | Some concerns | Low risk | No concerns | Some concerns | No concerns | Major concerns | Very low |
| Zinc:lifestyle_intervention_education | 0 | No concerns | Low risk | No concerns | Some concerns | No concerns | Major concerns | Low |
| Zinc:physical_activity | 0 | No concerns | Low risk | No concerns | Some concerns | No concerns | Major concerns | Low |
| Zinc:pro_pre_symbiotic | 0 | No concerns | Low risk | No concerns | Some concerns | No concerns | Major concerns | Low |
| curcumin:diet | 0 | Some concerns | Low risk | No concerns | Major concerns | No concerns | Major concerns | Very low |
| curcumin:enhanced_education | 0 | Some concerns | Low risk | No concerns | Some concerns | No concerns | Major concerns | Very low |
| curcumin:intensive lifestyle intervention | 0 | Some concerns | Low risk | No concerns | Some concerns | No concerns | Major concerns | Very low |
| curcumin:lifestyle_intervention_education | 0 | No concerns | Low risk | No concerns | Some concerns | No concerns | Major concerns | Low |
| curcumin:physical_activity | 0 | No concerns | Low risk | No concerns | Some concerns | No concerns | Major concerns | Low |
| curcumin:pro_pre_symbiotic | 0 | No concerns | Low risk | No concerns | Some concerns | Some concerns | Major concerns | Very low |
| diet:enhanced_education | 0 | Some concerns | Low risk | No concerns | Some concerns | No concerns | Major concerns | Very low |
| diet:intensive lifestyle intervention | 0 | Some concerns | Low risk | No concerns | Some concerns | No concerns | Major concerns | Very low |
| diet:lifestyle_intervention_education | 0 | Some concerns | Low risk | No concerns | Some concerns | No concerns | Major concerns | Very low |
| diet:physical_activity | 0 | Some concerns | Low risk | No concerns | Some concerns | No concerns | Major concerns | Very low |
| diet:pro_pre_symbiotic | 0 | Some concerns | Low risk | No concerns | Some concerns | Some concerns | Major concerns | Very low |
| enhanced_education:intensive lifestyle intervention | 0 | Some concerns | Low risk | No concerns | No concerns | Some concerns | Major concerns | Very low |
| enhanced_education:physical_activity | 0 | Some concerns | Low risk | No concerns | No concerns | Some concerns | Major concerns | Very low |
| enhanced_education:pro_pre_symbiotic | 0 | Some concerns | Low risk | No concerns | Some concerns | No concerns | Major concerns | Very low |
| intensive lifestyle intervention:physical_activity | 0 | Some concerns | Low risk | No concerns | No concerns | Some concerns | Major concerns | Very low |
| intensive lifestyle intervention:pro_pre_symbiotic | 0 | Some concerns | Low risk | No concerns | Some concerns | Some concerns | Major concerns | Very low |
| lifestyle_intervention_education:physical_activity | 0 | Some concerns | Low risk | No concerns | No concerns | Some concerns | Major concerns | Very low |
| lifestyle_intervention_education:pro_pre_symbiotic | 0 | No concerns | Low risk | No concerns | Some concerns | No concerns | Major concerns | Low |
| physical_activity:pro_pre_symbiotic | 0 | No concerns | Low risk | No concerns | Some concerns | Some concerns | Major concerns | Very low |

28. Table S10: Grading – 2hPG – 1 year (using CINeMA tool)

| Comparison | No of studies | Within-study bias | Reporting bias | Indirectness | Imprecision | Heterogeneity | Incoherence | Confidence rating |
| --- | --- | --- | --- | --- | --- | --- | --- | --- |
| AGI:control | 1 | No concerns | Low risk | No concerns | No concerns | Some concerns | Major concerns | Low |
| DPP4:Metformin | 1 | Some concerns | Low risk | No concerns | No concerns | Some concerns | No concerns | Moderate |
| DPP4:lifestyle_intervention_education | 1 | Some concerns | Low risk | No concerns | Some concerns | No concerns | No concerns | Moderate |
| L-arginine + lifestyle intervention:lifestyle_intervention_education | 1 | Some concerns | Low risk | No concerns | Some concerns | No concerns | Major concerns | Very low |
| Metformin:control | 1 | Some concerns | Low risk | No concerns | No concerns | Some concerns | No concerns | Moderate |
| Metformin:lifestyle_intervention_education | 1 | Some concerns | Low risk | No concerns | No concerns | Some concerns | No concerns | Moderate |
| Sulfonylureas:control | 1 | Some concerns | Low risk | No concerns | Some concerns | No concerns | Major concerns | Very low |
| Thiazolidindione:control | 1 | Some concerns | Low risk | No concerns | Some concerns | No concerns | Major concerns | Very low |
| Vitamin D:control | 5 | No concerns | Low risk | No concerns | No concerns | Some concerns | Major concerns | Low |
| Zinc:control | 1 | Major concerns | Low risk | No concerns | No concerns | No concerns | Major concerns | Very low |
| control:diet | 4 | Some concerns | Low risk | No concerns | No concerns | Some concerns | Major concerns | Very low |
| control:enhanced_education | 2 | Some concerns | Low risk | No concerns | No concerns | Some concerns | No concerns | Moderate |
| control:intensive lifestyle intervention | 6 | Some concerns | Low risk | No concerns | No concerns | Some concerns | No concerns | Moderate |
| control:lifestyle_intervention_education | 3 | Some concerns | Low risk | No concerns | No concerns | Some concerns | No concerns | Moderate |
| control:physical_activity | 4 | Some concerns | Low risk | No concerns | No concerns | Some concerns | Major concerns | Very low |
| enhanced_education:lifestyle_intervention_education | 1 | No concerns | Low risk | No concerns | No concerns | No concerns | No concerns | High |
| intensive lifestyle intervention:lifestyle_intervention_education | 4 | Some concerns | Low risk | No concerns | No concerns | Some concerns | No concerns | Moderate |
| AGI:DPP4 | 0 | Some concerns | Low risk | No concerns | Some concerns | No concerns | Major concerns | Very low |
| AGI:L-arginine + lifestyle intervention | 0 | Some concerns | Low risk | No concerns | Some concerns | Some concerns | Major concerns | Very low |
| AGI:Metformin | 0 | Some concerns | Low risk | No concerns | Some concerns | No concerns | Major concerns | Very low |
| AGI:Sulfonylureas | 0 | Some concerns | Low risk | No concerns | Some concerns | No concerns | Major concerns | Very low |
| AGI:Thiazolidindione | 0 | Some concerns | Low risk | No concerns | Some concerns | Some concerns | Major concerns | Very low |
| AGI:Vitamin D | 0 | No concerns | Low risk | No concerns | No concerns | Some concerns | Major concerns | Low |
| AGI:Zinc | 0 | Some concerns | Low risk | No concerns | No concerns | Some concerns | Major concerns | Very low |
| AGI:diet | 0 | No concerns | Low risk | No concerns | Some concerns | No concerns | Major concerns | Low |
| AGI:enhanced_education | 0 | No concerns | Low risk | No concerns | No concerns | Some concerns | Major concerns | Low |
| AGI:intensive lifestyle intervention | 0 | Some concerns | Low risk | No concerns | Some concerns | No concerns | Major concerns | Very low |
| AGI:lifestyle_intervention_education | 0 | No concerns | Low risk | No concerns | No concerns | Some concerns | Major concerns | Low |
| AGI:physical_activity | 0 | No concerns | Low risk | No concerns | Some concerns | No concerns | Major concerns | Low |
| DPP4:L-arginine + lifestyle intervention | 0 | Some concerns | Low risk | No concerns | Some concerns | Some concerns | Major concerns | Very low |
| DPP4:Sulfonylureas | 0 | Some concerns | Low risk | No concerns | Major concerns | No concerns | Major concerns | Very low |
| DPP4:Thiazolidindione | 0 | Some concerns | Low risk | No concerns | Some concerns | No concerns | Major concerns | Very low |
| DPP4:Vitamin D | 0 | Some concerns | Low risk | No concerns | Some concerns | No concerns | Major concerns | Very low |
| DPP4:Zinc | 0 | Some concerns | Low risk | No concerns | Some concerns | No concerns | Major concerns | Very low |
| DPP4:control | 0 | Some concerns | Low risk | No concerns | No concerns | Some concerns | Major concerns | Very low |
| DPP4:diet | 0 | Some concerns | Low risk | No concerns | Some concerns | No concerns | Major concerns | Very low |
| DPP4:enhanced_education | 0 | Some concerns | Low risk | No concerns | Some concerns | No concerns | Major concerns | Very low |
| DPP4:intensive lifestyle intervention | 0 | Some concerns | Low risk | No concerns | No concerns | Some concerns | Major concerns | Very low |
| DPP4:physical_activity | 0 | Some concerns | Low risk | No concerns | No concerns | Major concerns | Major concerns | Very low |
| L-arginine + lifestyle intervention:Metformin | 0 | Some concerns | Low risk | No concerns | Major concerns | No concerns | Major concerns | Very low |
| L-arginine + lifestyle intervention:Sulfonylureas | 0 | Some concerns | Low risk | No concerns | Major concerns | No concerns | Major concerns | Very low |
| L-arginine + lifestyle intervention:Thiazolidindione | 0 | Some concerns | Low risk | No concerns | Major concerns | No concerns | Major concerns | Very low |
| L-arginine + lifestyle intervention:Vitamin D | 0 | Some concerns | Low risk | No concerns | Some concerns | No concerns | Major concerns | Very low |
| L-arginine + lifestyle intervention:Zinc | 0 | Some concerns | Low risk | No concerns | Some concerns | No concerns | Major concerns | Very low |
| L-arginine + lifestyle intervention:control | 0 | Some concerns | Low risk | No concerns | Some concerns | No concerns | Major concerns | Very low |
| L-arginine + lifestyle intervention:diet | 0 | Some concerns | Low risk | No concerns | Major concerns | No concerns | Major concerns | Very low |
| L-arginine + lifestyle intervention:enhanced_education | 0 | Some concerns | Low risk | No concerns | Some concerns | Some concerns | Major concerns | Very low |
| L-arginine + lifestyle intervention:intensive lifestyle intervention | 0 | Some concerns | Low risk | No concerns | Some concerns | Some concerns | Major concerns | Very low |
| L-arginine + lifestyle intervention:physical_activity | 0 | Some concerns | Low risk | No concerns | Some concerns | Some concerns | Major concerns | Very low |
| Metformin:Sulfonylureas | 0 | Some concerns | Low risk | No concerns | Major concerns | No concerns | Major concerns | Very low |
| Metformin:Thiazolidindione | 0 | Some concerns | Low risk | No concerns | Some concerns | Some concerns | Major concerns | Very low |
| Metformin:Vitamin D | 0 | Some concerns | Low risk | No concerns | Some concerns | No concerns | Major concerns | Very low |
| Metformin:Zinc | 0 | Some concerns | Low risk | No concerns | Some concerns | No concerns | Major concerns | Very low |
| Metformin:diet | 0 | Some concerns | Low risk | No concerns | No concerns | Some concerns | Major concerns | Very low |
| Metformin:enhanced_education | 0 | Some concerns | Low risk | No concerns | Some concerns | No concerns | Major concerns | Very low |
| Metformin:intensive lifestyle intervention | 0 | Some concerns | Low risk | No concerns | No concerns | No concerns | Major concerns | Low |
| Metformin:physical_activity | 0 | Some concerns | Low risk | No concerns | No concerns | Some concerns | Major concerns | Very low |
| Sulfonylureas:Thiazolidindione | 0 | Some concerns | Low risk | No concerns | Some concerns | Some concerns | Major concerns | Very low |
| Sulfonylureas:Vitamin D | 0 | Some concerns | Low risk | No concerns | Some concerns | No concerns | Major concerns | Very low |
| Sulfonylureas:Zinc | 0 | Major concerns | Low risk | No concerns | Major concerns | No concerns | Major concerns | Very low |
| Sulfonylureas:diet | 0 | Some concerns | Low risk | No concerns | Some concerns | Some concerns | Major concerns | Very low |
| Sulfonylureas:enhanced_education | 0 | Some concerns | Low risk | No concerns | Some concerns | No concerns | Major concerns | Very low |
| Sulfonylureas:intensive lifestyle intervention | 0 | Some concerns | Low risk | No concerns | Some concerns | Some concerns | Major concerns | Very low |
| Sulfonylureas:lifestyle_intervention_education | 0 | Some concerns | Low risk | No concerns | Some concerns | No concerns | Major concerns | Very low |
| Sulfonylureas:physical_activity | 0 | Some concerns | Low risk | No concerns | Major concerns | No concerns | Major concerns | Very low |
| Thiazolidindione:Vitamin D | 0 | Some concerns | Low risk | No concerns | Some concerns | Some concerns | Major concerns | Very low |
| Thiazolidindione:Zinc | 0 | Major concerns | Low risk | No concerns | Some concerns | No concerns | Major concerns | Very low |
| Thiazolidindione:diet | 0 | Some concerns | Low risk | No concerns | Some concerns | Some concerns | Major concerns | Very low |
| Thiazolidindione:enhanced_education | 0 | Some concerns | Low risk | No concerns | No concerns | Major concerns | Major concerns | Very low |
| Thiazolidindione:intensive lifestyle intervention | 0 | Some concerns | Low risk | No concerns | Some concerns | No concerns | Major concerns | Very low |
| Thiazolidindione:lifestyle_intervention_education | 0 | Some concerns | Low risk | No concerns | No concerns | Major concerns | Major concerns | Very low |
| Thiazolidindione:physical_activity | 0 | Some concerns | Low risk | No concerns | Some concerns | No concerns | Major concerns | Very low |
| Vitamin D:Zinc | 0 | Some concerns | Low risk | No concerns | No concerns | No concerns | Major concerns | Low |
| Vitamin D:diet | 0 | Some concerns | Low risk | No concerns | No concerns | Some concerns | Major concerns | Very low |
| Vitamin D:enhanced_education | 0 | Some concerns | Low risk | No concerns | No concerns | Some concerns | Major concerns | Very low |
| Vitamin D:intensive lifestyle intervention | 0 | Some concerns | Low risk | No concerns | No concerns | Some concerns | Major concerns | Very low |
| Vitamin D:lifestyle_intervention_education | 0 | Some concerns | Low risk | No concerns | No concerns | No concerns | Major concerns | Low |
| Vitamin D:physical_activity | 0 | Some concerns | Low risk | No concerns | Some concerns | No concerns | Major concerns | Very low |
| Zinc:diet | 0 | Some concerns | Low risk | No concerns | No concerns | Some concerns | Major concerns | Very low |
| Zinc:enhanced_education | 0 | Some concerns | Low risk | No concerns | No concerns | Some concerns | Major concerns | Very low |
| Zinc:intensive lifestyle intervention | 0 | Some concerns | Low risk | No concerns | Some concerns | No concerns | Major concerns | Very low |
| Zinc:lifestyle_intervention_education | 0 | Some concerns | Low risk | No concerns | No concerns | Some concerns | Major concerns | Very low |
| Zinc:physical_activity | 0 | Some concerns | Low risk | No concerns | Some concerns | No concerns | Major concerns | Very low |
| diet:enhanced_education | 0 | Some concerns | Low risk | No concerns | No concerns | Some concerns | Major concerns | Very low |
| diet:intensive lifestyle intervention | 0 | Some concerns | Low risk | No concerns | No concerns | No concerns | Major concerns | Low |
| diet:lifestyle_intervention_education | 0 | Some concerns | Low risk | No concerns | No concerns | Some concerns | Major concerns | Very low |
| diet:physical_activity | 0 | Some concerns | Low risk | No concerns | No concerns | Some concerns | Major concerns | Very low |
| enhanced_education:intensive lifestyle intervention | 0 | Some concerns | Low risk | No concerns | No concerns | Some concerns | Major concerns | Very low |
| enhanced_education:physical_activity | 0 | Some concerns | Low risk | No concerns | No concerns | Some concerns | Major concerns | Very low |
| intensive lifestyle intervention:physical_activity | 0 | Some concerns | Low risk | No concerns | No concerns | No concerns | Major concerns | Low |
| lifestyle_intervention_education:physical_activity | 0 | Some concerns | Low risk | No concerns | No concerns | Some concerns | Major concerns | Very low |

29. Table S11: Grading – 2hPG – 6 months (using CINeMA tool)

| Comparison | No of studies | Within-study bias | Reporting bias | Indirectness | Imprecision | Heterogeneity | Incoherence | Confidence rating |
| --- | --- | --- | --- | --- | --- | --- | --- | --- |
| Antihipertensive:control | 1 | No concerns | Low risk | No concerns | Some concerns | No concerns | Major concerns | Low |
| DPP4:Metformin | 1 | Some concerns | Low risk | No concerns | No concerns | Some concerns | No concerns | Moderate |
| DPP4:control | 1 | Some concerns | Low risk | No concerns | Some concerns | No concerns | No concerns | Moderate |
| Metformin:control | 3 | Some concerns | Low risk | No concerns | No concerns | No concerns | No concerns | High |
| Metformin:lifestyle_intervention_education | 1 | Some concerns | Low risk | No concerns | No concerns | No concerns | Major concerns | Low |
| Metformin:lipid low | 1 | Some concerns | Low risk | No concerns | Some concerns | Some concerns | Major concerns | Very low |
| Sulfonylureas:control | 1 | Some concerns | Low risk | No concerns | Some concerns | Some concerns | Major concerns | Very low |
| Vitamin D:control | 4 | Some concerns | Low risk | No concerns | No concerns | No concerns | Major concerns | Low |
| Zinc:control | 1 | Major concerns | Low risk | No concerns | No concerns | Some concerns | Major concerns | Very low |
| control:diet | 1 | Some concerns | Low risk | No concerns | Some concerns | No concerns | Major concerns | Very low |
| control:intensive lifestyle intervention | 2 | Some concerns | Low risk | No concerns | No concerns | Some concerns | No concerns | Moderate |
| control:lifestyle_intervention_education | 3 | Some concerns | Low risk | No concerns | No concerns | No concerns | No concerns | High |
| control:physical_activity | 4 | Some concerns | Low risk | No concerns | No concerns | Some concerns | Major concerns | Low |
| intensive lifestyle intervention:lifestyle_intervention_education | 1 | Some concerns | Low risk | No concerns | No concerns | Some concerns | No concerns | Moderate |
| intensive lifestyle intervention:physical_activity | 1 | Some concerns | Low risk | No concerns | No concerns | No concerns | Major concerns | Low |
| Antihipertensive:DPP4 | 0 | Some concerns | Low risk | No concerns | Some concerns | Some concerns | Major concerns | Very low |
| Antihipertensive:Metformin | 0 | Some concerns | Low risk | No concerns | Some concerns | No concerns | Major concerns | Very low |
| Antihipertensive:Sulfonylureas | 0 | Some concerns | Low risk | No concerns | Some concerns | Some concerns | Major concerns | Very low |
| Antihipertensive:Vitamin D | 0 | No concerns | Low risk | No concerns | Some concerns | No concerns | Major concerns | Low |
| Antihipertensive:Zinc | 0 | Some concerns | Low risk | No concerns | Some concerns | Some concerns | Major concerns | Very low |
| Antihipertensive:diet | 0 | Some concerns | Low risk | No concerns | Major concerns | No concerns | Major concerns | Very low |
| Antihipertensive:intensive lifestyle intervention | 0 | Some concerns | Low risk | No concerns | Some concerns | Some concerns | Major concerns | Very low |
| Antihipertensive:lifestyle_intervention_education | 0 | No concerns | Low risk | No concerns | Some concerns | No concerns | Major concerns | Low |
| Antihipertensive:lipid low | 0 | Some concerns | Low risk | No concerns | Some concerns | No concerns | Major concerns | Very low |
| Antihipertensive:physical_activity | 0 | No concerns | Low risk | No concerns | Some concerns | Some concerns | Major concerns | Very low |
| DPP4:Sulfonylureas | 0 | Some concerns | Low risk | No concerns | Major concerns | No concerns | Major concerns | Very low |
| DPP4:Vitamin D | 0 | Some concerns | Low risk | No concerns | Some concerns | No concerns | Major concerns | Very low |
| DPP4:Zinc | 0 | Some concerns | Low risk | No concerns | Some concerns | No concerns | Major concerns | Very low |
| DPP4:diet | 0 | Some concerns | Low risk | No concerns | Major concerns | No concerns | Major concerns | Very low |
| DPP4:intensive lifestyle intervention | 0 | Some concerns | Low risk | No concerns | Some concerns | No concerns | Major concerns | Very low |
| DPP4:lifestyle_intervention_education | 0 | Some concerns | Low risk | No concerns | No concerns | Some concerns | Major concerns | Very low |
| DPP4:lipid low | 0 | Some concerns | Low risk | No concerns | Some concerns | Some concerns | Major concerns | Very low |
| DPP4:physical_activity | 0 | Some concerns | Low risk | No concerns | Some concerns | No concerns | Major concerns | Very low |
| Metformin:Sulfonylureas | 0 | Some concerns | Low risk | No concerns | Some concerns | Some concerns | Major concerns | Very low |
| Metformin:Vitamin D | 0 | Some concerns | Low risk | No concerns | No concerns | No concerns | Major concerns | Low |
| Metformin:Zinc | 0 | Some concerns | Low risk | No concerns | Some concerns | No concerns | Major concerns | Very low |
| Metformin:diet | 0 | Some concerns | Low risk | No concerns | Some concerns | Some concerns | Major concerns | Very low |
| Metformin:intensive lifestyle intervention | 0 | Some concerns | Low risk | No concerns | No concerns | Some concerns | Major concerns | Very low |
| Metformin:physical_activity | 0 | Some concerns | Low risk | No concerns | No concerns | Some concerns | Major concerns | Very low |
| Sulfonylureas:Vitamin D | 0 | Some concerns | Low risk | No concerns | Major concerns | No concerns | Major concerns | Very low |
| Sulfonylureas:Zinc | 0 | Major concerns | Low risk | No concerns | Some concerns | No concerns | Major concerns | Very low |
| Sulfonylureas:diet | 0 | Some concerns | Low risk | No concerns | Major concerns | No concerns | Major concerns | Very low |
| Sulfonylureas:intensive lifestyle intervention | 0 | Some concerns | Low risk | No concerns | Some concerns | No concerns | Major concerns | Very low |
| Sulfonylureas:lifestyle_intervention_education | 0 | Some concerns | Low risk | No concerns | Some concerns | Some concerns | Major concerns | Very low |
| Sulfonylureas:lipid low | 0 | Some concerns | Low risk | No concerns | Major concerns | No concerns | Major concerns | Very low |
| Sulfonylureas:physical_activity | 0 | Some concerns | Low risk | No concerns | Some concerns | No concerns | Major concerns | Very low |
| Vitamin D:Zinc | 0 | Some concerns | Low risk | No concerns | Some concerns | No concerns | Major concerns | Very low |
| Vitamin D:diet | 0 | Some concerns | Low risk | No concerns | Some concerns | Some concerns | Major concerns | Very low |
| Vitamin D:intensive lifestyle intervention | 0 | Some concerns | Low risk | No concerns | No concerns | Some concerns | Major concerns | Very low |
| Vitamin D:lifestyle_intervention_education | 0 | Some concerns | Low risk | No concerns | No concerns | No concerns | Major concerns | Low |
| Vitamin D:lipid low | 0 | Some concerns | Low risk | No concerns | Some concerns | Some concerns | Major concerns | Very low |
| Vitamin D:physical_activity | 0 | Some concerns | Low risk | No concerns | No concerns | Some concerns | Major concerns | Very low |
| Zinc:diet | 0 | Major concerns | Low risk | No concerns | Some concerns | Some concerns | Major concerns | Very low |
| Zinc:intensive lifestyle intervention | 0 | Some concerns | Low risk | No concerns | Some concerns | No concerns | Major concerns | Very low |
| Zinc:lifestyle_intervention_education | 0 | Some concerns | Low risk | No concerns | Some concerns | No concerns | Major concerns | Very low |
| Zinc:lipid low | 0 | Some concerns | Low risk | No concerns | Some concerns | No concerns | Major concerns | Very low |
| Zinc:physical_activity | 0 | Some concerns | Low risk | No concerns | Some concerns | No concerns | Major concerns | Very low |
| control:lipid low | 0 | Some concerns | Low risk | No concerns | Major concerns | No concerns | Major concerns | Very low |
| diet:intensive lifestyle intervention | 0 | Some concerns | Low risk | No concerns | Major concerns | No concerns | Major concerns | Very low |
| diet:lifestyle_intervention_education | 0 | Some concerns | Low risk | No concerns | Some concerns | Some concerns | Major concerns | Very low |
| diet:lipid low | 0 | Some concerns | Low risk | No concerns | Some concerns | Some concerns | Major concerns | Very low |
| diet:physical_activity | 0 | Some concerns | Low risk | No concerns | Some concerns | Some concerns | Major concerns | Very low |
| intensive lifestyle intervention:lipid low | 0 | Some concerns | Low risk | No concerns | Some concerns | No concerns | Major concerns | Very low |
| lifestyle_intervention_education:lipid low | 0 | Some concerns | Low risk | No concerns | Some concerns | Some concerns | Major concerns | Very low |
| lifestyle_intervention_education:physical_activity | 0 | Some concerns | Low risk | No concerns | No concerns | Some concerns | Major concerns | Very low |
| lipid low:physical_activity | 0 | Some concerns | Low risk | No concerns | Some concerns | No concerns | Major concerns | Very low |

30. Table S12: Grading – FPG – 1 year (using CINeMA tool)

| Comparison | No of studies | Within-study bias | Reporting bias | Indirectness | Imprecision | Heterogeneity | Incoherence | Confidence rating |
| --- | --- | --- | --- | --- | --- | --- | --- | --- |
| DPP4:Metformin | 1 | Some concerns | Low risk | No concerns | No concerns | No concerns | No concerns | High |
| DPP4:Metformin + DPP4 | 1 | Some concerns | Low risk | No concerns | Some concerns | No concerns | No concerns | Moderate |
| DPP4:lifestyle_intervention_education | 1 | Some concerns | Low risk | No concerns | Some concerns | No concerns | No concerns | Moderate |
| GLP1:control | 3 | No concerns | Low risk | No concerns | No concerns | No concerns | Major concerns | Moderate |
| GLP1+GIP:control | 1 | Some concerns | Low risk | No concerns | No concerns | No concerns | Major concerns | Low |
| Metformin:Metformin + DPP4 | 2 | Some concerns | Low risk | No concerns | No concerns | Some concerns | No concerns | Moderate |
| Metformin:control | 2 | Some concerns | Low risk | No concerns | No concerns | Some concerns | No concerns | Moderate |
| Metformin:intensive lifestyle intervention | 2 | Some concerns | Low risk | No concerns | No concerns | No concerns | No concerns | High |
| Metformin:lifestyle_intervention_education | 2 | Some concerns | Low risk | No concerns | No concerns | Some concerns | No concerns | Moderate |
| Metformin + DPP4:lifestyle_intervention_education | 1 | Some concerns | Low risk | No concerns | No concerns | No concerns | No concerns | High |
| Thiazolidindione:control | 1 | Some concerns | Low risk | No concerns | No concerns | Some concerns | Major concerns | Very low |
| Zinc:control | 2 | Some concerns | Low risk | No concerns | No concerns | No concerns | Major concerns | Low |
| control:diet | 2 | No concerns | Low risk | No concerns | No concerns | Some concerns | Major concerns | Low |
| control:enhanced_education | 4 | Some concerns | Low risk | No concerns | No concerns | No concerns | No concerns | High |
| control:intensive lifestyle intervention | 16 | Some concerns | Low risk | No concerns | No concerns | Some concerns | No concerns | Moderate |
| control:lifestyle_intervention_education | 10 | Some concerns | Low risk | No concerns | No concerns | No concerns | No concerns | High |
| control:physical_activity | 4 | Some concerns | Low risk | No concerns | No concerns | No concerns | Major concerns | Low |
| control:sulfonylureas | 1 | Some concerns | Low risk | No concerns | No concerns | No concerns | Major concerns | Low |
| control:vitamin D | 5 | No concerns | Low risk | No concerns | No concerns | Some concerns | Major concerns | Low |
| enhanced_education:lifestyle_intervention_education | 4 | Some concerns | Low risk | No concerns | No concerns | No concerns | No concerns | High |
| intensive lifestyle intervention:lifestyle_intervention_education | 14 | Some concerns | Low risk | No concerns | No concerns | No concerns | No concerns | High |
| DPP4:GLP1 | 0 | Some concerns | Low risk | No concerns | Some concerns | No concerns | Major concerns | Very low |
| DPP4:GLP1+GIP | 0 | Some concerns | Low risk | No concerns | Some concerns | No concerns | Major concerns | Very low |
| DPP4:Thiazolidindione | 0 | Some concerns | Low risk | No concerns | Some concerns | No concerns | Major concerns | Very low |
| DPP4:Zinc | 0 | Some concerns | Low risk | No concerns | Some concerns | No concerns | Major concerns | Very low |
| DPP4:control | 0 | Some concerns | Low risk | No concerns | Some concerns | No concerns | Major concerns | Very low |
| DPP4:diet | 0 | Some concerns | Low risk | No concerns | Some concerns | No concerns | Major concerns | Very low |
| DPP4:enhanced_education | 0 | Some concerns | Low risk | No concerns | Some concerns | No concerns | Major concerns | Very low |
| DPP4:intensive lifestyle intervention | 0 | Some concerns | Low risk | No concerns | No concerns | Some concerns | Major concerns | Very low |
| DPP4:physical_activity | 0 | Some concerns | Low risk | No concerns | Some concerns | No concerns | Major concerns | Very low |
| DPP4:sulfonylureas | 0 | Some concerns | Low risk | No concerns | No concerns | Some concerns | Major concerns | Very low |
| DPP4:vitamin D | 0 | Some concerns | Low risk | No concerns | No concerns | Some concerns | Major concerns | Very low |
| GLP1:GLP1+GIP | 0 | Some concerns | Low risk | No concerns | No concerns | Some concerns | Major concerns | Very low |
| GLP1:Metformin | 0 | Some concerns | Low risk | No concerns | No concerns | Some concerns | Major concerns | Very low |
| GLP1:Metformin + DPP4 | 0 | Some concerns | Low risk | No concerns | No concerns | Some concerns | Major concerns | Very low |
| GLP1:Thiazolidindione | 0 | Some concerns | Low risk | No concerns | No concerns | Major concerns | Major concerns | Very low |
| GLP1:Zinc | 0 | No concerns | Low risk | No concerns | No concerns | Some concerns | Major concerns | Low |
| GLP1:diet | 0 | No concerns | Low risk | No concerns | No concerns | Some concerns | Major concerns | Low |
| GLP1:enhanced_education | 0 | Some concerns | Low risk | No concerns | No concerns | No concerns | Major concerns | Low |
| GLP1:intensive lifestyle intervention | 0 | Some concerns | Low risk | No concerns | No concerns | Some concerns | Major concerns | Very low |
| GLP1:lifestyle_intervention_education | 0 | Some concerns | Low risk | No concerns | No concerns | Some concerns | Major concerns | Very low |
| GLP1:physical_activity | 0 | Some concerns | Low risk | No concerns | No concerns | Some concerns | Major concerns | Very low |
| GLP1:sulfonylureas | 0 | Some concerns | Low risk | No concerns | Some concerns | No concerns | Major concerns | Very low |
| GLP1:vitamin D | 0 | No concerns | Low risk | No concerns | No concerns | Some concerns | Major concerns | Low |
| GLP1+GIP:Metformin | 0 | Some concerns | Low risk | No concerns | Some concerns | No concerns | Major concerns | Very low |
| GLP1+GIP:Metformin + DPP4 | 0 | Some concerns | Low risk | No concerns | Some concerns | No concerns | Major concerns | Very low |
| GLP1+GIP:Thiazolidindione | 0 | Some concerns | Low risk | No concerns | Some concerns | Some concerns | Major concerns | Very low |
| GLP1+GIP:Zinc | 0 | Some concerns | Low risk | No concerns | Some concerns | No concerns | Major concerns | Very low |
| GLP1+GIP:diet | 0 | Some concerns | Low risk | No concerns | No concerns | Some concerns | Major concerns | Very low |
| GLP1+GIP:enhanced_education | 0 | Some concerns | Low risk | No concerns | No concerns | Some concerns | Major concerns | Very low |
| GLP1+GIP:intensive lifestyle intervention | 0 | Some concerns | Low risk | No concerns | Some concerns | No concerns | Major concerns | Very low |
| GLP1+GIP:lifestyle_intervention_education | 0 | Some concerns | Low risk | No concerns | No concerns | Some concerns | Major concerns | Very low |
| GLP1+GIP:physical_activity | 0 | Some concerns | Low risk | No concerns | Some concerns | No concerns | Major concerns | Very low |
| GLP1+GIP:sulfonylureas | 0 | Some concerns | Low risk | No concerns | Some concerns | No concerns | Major concerns | Very low |
| GLP1+GIP:vitamin D | 0 | Some concerns | Low risk | No concerns | Some concerns | No concerns | Major concerns | Very low |
| Metformin:Thiazolidindione | 0 | Some concerns | Low risk | No concerns | Some concerns | No concerns | Major concerns | Very low |
| Metformin:Zinc | 0 | Some concerns | Low risk | No concerns | Some concerns | No concerns | Major concerns | Very low |
| Metformin:diet | 0 | Some concerns | Low risk | No concerns | Some concerns | No concerns | Major concerns | Very low |
| Metformin:enhanced_education | 0 | Some concerns | Low risk | No concerns | No concerns | Some concerns | Major concerns | Very low |
| Metformin:physical_activity | 0 | Some concerns | Low risk | No concerns | No concerns | Some concerns | Major concerns | Very low |
| Metformin:sulfonylureas | 0 | Some concerns | Low risk | No concerns | No concerns | No concerns | Major concerns | Low |
| Metformin:vitamin D | 0 | Some concerns | Low risk | No concerns | No concerns | No concerns | Major concerns | Low |
| Metformin + DPP4:Thiazolidindione | 0 | Some concerns | Low risk | No concerns | Some concerns | No concerns | Major concerns | Very low |
| Metformin + DPP4:Zinc | 0 | Some concerns | Low risk | No concerns | No concerns | Major concerns | Major concerns | Very low |
| Metformin + DPP4:control | 0 | Some concerns | Low risk | No concerns | No concerns | No concerns | Major concerns | Low |
| Metformin + DPP4:diet | 0 | Some concerns | Low risk | No concerns | No concerns | No concerns | Major concerns | Low |
| Metformin + DPP4:enhanced_education | 0 | Some concerns | Low risk | No concerns | No concerns | No concerns | Major concerns | Low |
| Metformin + DPP4:intensive lifestyle intervention | 0 | Some concerns | Low risk | No concerns | No concerns | Some concerns | Major concerns | Very low |
| Metformin + DPP4:physical_activity | 0 | Some concerns | Low risk | No concerns | No concerns | Some concerns | Major concerns | Very low |
| Metformin + DPP4:sulfonylureas | 0 | Some concerns | Low risk | No concerns | Some concerns | No concerns | Major concerns | Very low |
| Metformin + DPP4:vitamin D | 0 | Some concerns | Low risk | No concerns | Some concerns | No concerns | Major concerns | Very low |
| Thiazolidindione:Zinc | 0 | Some concerns | Low risk | No concerns | Some concerns | No concerns | Major concerns | Very low |
| Thiazolidindione:diet | 0 | Some concerns | Low risk | No concerns | Some concerns | No concerns | Major concerns | Very low |
| Thiazolidindione:enhanced_education | 0 | Some concerns | Low risk | No concerns | Some concerns | No concerns | Major concerns | Very low |
| Thiazolidindione:intensive lifestyle intervention | 0 | Some concerns | Low risk | No concerns | Some concerns | No concerns | Major concerns | Very low |
| Thiazolidindione:lifestyle_intervention_education | 0 | Some concerns | Low risk | No concerns | Some concerns | No concerns | Major concerns | Very low |
| Thiazolidindione:physical_activity | 0 | Some concerns | Low risk | No concerns | Some concerns | No concerns | Major concerns | Very low |
| Thiazolidindione:sulfonylureas | 0 | Some concerns | Low risk | No concerns | Some concerns | No concerns | Major concerns | Very low |
| Thiazolidindione:vitamin D | 0 | Some concerns | Low risk | No concerns | Some concerns | No concerns | Major concerns | Very low |
| Zinc:diet | 0 | Some concerns | Low risk | No concerns | No concerns | No concerns | Major concerns | Low |
| Zinc:enhanced_education | 0 | Some concerns | Low risk | No concerns | No concerns | No concerns | Major concerns | Low |
| Zinc:intensive lifestyle intervention | 0 | Some concerns | Low risk | No concerns | No concerns | Some concerns | Major concerns | Very low |
| Zinc:lifestyle_intervention_education | 0 | Some concerns | Low risk | No concerns | No concerns | No concerns | Major concerns | Low |
| Zinc:physical_activity | 0 | Some concerns | Low risk | No concerns | No concerns | Some concerns | Major concerns | Very low |
| Zinc:sulfonylureas | 0 | Some concerns | Low risk | No concerns | Some concerns | No concerns | Major concerns | Very low |
| Zinc:vitamin D | 0 | Some concerns | Low risk | No concerns | Some concerns | No concerns | Major concerns | Very low |
| diet:enhanced_education | 0 | Some concerns | Low risk | No concerns | No concerns | No concerns | Major concerns | Low |
| diet:intensive lifestyle intervention | 0 | Some concerns | Low risk | No concerns | No concerns | Some concerns | Major concerns | Very low |
| diet:lifestyle_intervention_education | 0 | Some concerns | Low risk | No concerns | No concerns | Some concerns | Major concerns | Very low |
| diet:physical_activity | 0 | Some concerns | Low risk | No concerns | No concerns | No concerns | Major concerns | Low |
| diet:sulfonylureas | 0 | Some concerns | Low risk | No concerns | No concerns | No concerns | Major concerns | Low |
| diet:vitamin D | 0 | No concerns | Low risk | No concerns | Some concerns | No concerns | Major concerns | Low |
| enhanced_education:intensive lifestyle intervention | 0 | Some concerns | Low risk | No concerns | No concerns | Some concerns | Major concerns | Very low |
| enhanced_education:physical_activity | 0 | Some concerns | Low risk | No concerns | No concerns | No concerns | Major concerns | Low |
| enhanced_education:sulfonylureas | 0 | Some concerns | Low risk | No concerns | No concerns | No concerns | Major concerns | Low |
| enhanced_education:vitamin D | 0 | Some concerns | Low risk | No concerns | No concerns | Some concerns | Major concerns | Very low |
| intensive lifestyle intervention:physical_activity | 0 | Some concerns | Low risk | No concerns | No concerns | No concerns | Major concerns | Low |
| intensive lifestyle intervention:sulfonylureas | 0 | Some concerns | Low risk | No concerns | No concerns | No concerns | Major concerns | Low |
| intensive lifestyle intervention:vitamin D | 0 | Some concerns | Low risk | No concerns | No concerns | No concerns | Major concerns | Low |
| lifestyle_intervention_education:physical_activity | 0 | Some concerns | Low risk | No concerns | No concerns | No concerns | Major concerns | Low |
| lifestyle_intervention_education:sulfonylureas | 0 | Some concerns | Low risk | No concerns | No concerns | No concerns | Major concerns | Low |
| lifestyle_intervention_education:vitamin D | 0 | Some concerns | Low risk | No concerns | No concerns | Some concerns | Major concerns | Very low |
| physical_activity:sulfonylureas | 0 | Some concerns | Low risk | No concerns | Some concerns | No concerns | Major concerns | Very low |
| physical_activity:vitamin D | 0 | Some concerns | Low risk | No concerns | No concerns | Some concerns | Major concerns | Very low |
| sulfonylureas:vitamin D | 0 | Some concerns | Low risk | No concerns | No concerns | Some concerns | Major concerns | Very low |

31. Table S13: Grading – FPG – 6 months (using CINeMA tool)

| Comparison | No of studies | Within-study bias | Reporting bias | Indirectness | Imprecision | Heterogeneity | Incoherence | Confidence rating |
| --- | --- | --- | --- | --- | --- | --- | --- | --- |
| Antihipertensive:control | 2 | No concerns | Low risk | No concerns | No concerns | No concerns | Major concerns | Moderate |
| GLP1+GIP:control | 1 | Some concerns | Low risk | No concerns | No concerns | No concerns | Major concerns | Low |
| Metformin:Metformin + DPP4 | 2 | Some concerns | Low risk | No concerns | No concerns | No concerns | Major concerns | Low |
| Metformin:control | 3 | Some concerns | Low risk | No concerns | No concerns | No concerns | No concerns | High |
| Metformin:lifestyle_intervention_education | 2 | Some concerns | Low risk | No concerns | No concerns | No concerns | No concerns | High |
| Metformin:lipid_low | 1 | Some concerns | Low risk | No concerns | No concerns | No concerns | No concerns | High |
| Zinc:control | 2 | Some concerns | Low risk | No concerns | No concerns | No concerns | Major concerns | Low |
| control:diet | 1 | Some concerns | Low risk | No concerns | No concerns | No concerns | No concerns | High |
| control:enhanced_education | 4 | Some concerns | Low risk | No concerns | No concerns | No concerns | No concerns | High |
| control:intensive lifestyle intervention | 10 | Some concerns | Low risk | No concerns | No concerns | No concerns | No concerns | High |
| control:lifestyle_intervention_education | 7 | Some concerns | Low risk | No concerns | No concerns | No concerns | No concerns | High |
| control:lipid_low | 1 | Some concerns | Low risk | No concerns | No concerns | No concerns | No concerns | High |
| control:physical_activity | 5 | Some concerns | Low risk | No concerns | No concerns | No concerns | No concerns | High |
| control:probiotic | 1 | No concerns | Low risk | No concerns | No concerns | No concerns | Major concerns | Moderate |
| control:sulfonylureas | 1 | Some concerns | Low risk | No concerns | No concerns | No concerns | Major concerns | Low |
| control:vitamin D | 6 | No concerns | Low risk | No concerns | No concerns | No concerns | Major concerns | Moderate |
| diet:intensive lifestyle intervention | 1 | Some concerns | Low risk | No concerns | No concerns | No concerns | No concerns | High |
| enhanced_education:lifestyle_intervention_education | 3 | Some concerns | Low risk | No concerns | No concerns | No concerns | No concerns | High |
| intensive lifestyle intervention:lifestyle_intervention_education | 7 | Some concerns | Low risk | No concerns | No concerns | No concerns | No concerns | High |
| intensive lifestyle intervention:physical_activity | 1 | Some concerns | Low risk | No concerns | No concerns | No concerns | No concerns | High |
| lifestyle_intervention_education:physical_activity | 1 | Some concerns | Low risk | No concerns | No concerns | No concerns | No concerns | High |
| Antihipertensive:GLP1+GIP | 0 | Some concerns | Low risk | No concerns | No concerns | No concerns | Major concerns | Low |
| Antihipertensive:Metformin | 0 | Some concerns | Low risk | No concerns | No concerns | No concerns | Major concerns | Low |
| Antihipertensive:Metformin + DPP4 | 0 | Some concerns | Low risk | No concerns | No concerns | No concerns | Major concerns | Low |
| Antihipertensive:Zinc | 0 | Some concerns | Low risk | No concerns | No concerns | No concerns | Major concerns | Low |
| Antihipertensive:diet | 0 | Some concerns | Low risk | No concerns | No concerns | No concerns | Major concerns | Low |
| Antihipertensive:enhanced_education | 0 | Some concerns | Low risk | No concerns | No concerns | No concerns | Major concerns | Low |
| Antihipertensive:intensive lifestyle intervention | 0 | Some concerns | Low risk | No concerns | No concerns | No concerns | Major concerns | Low |
| Antihipertensive:lifestyle_intervention_education | 0 | Some concerns | Low risk | No concerns | No concerns | No concerns | Major concerns | Low |
| Antihipertensive:lipid_low | 0 | Some concerns | Low risk | No concerns | No concerns | No concerns | Major concerns | Low |
| Antihipertensive:physical_activity | 0 | Some concerns | Low risk | No concerns | No concerns | No concerns | Major concerns | Low |
| Antihipertensive:probiotic | 0 | No concerns | Low risk | No concerns | No concerns | No concerns | Major concerns | Moderate |
| Antihipertensive:sulfonylureas | 0 | Some concerns | Low risk | No concerns | No concerns | No concerns | Major concerns | Low |
| Antihipertensive:vitamin D | 0 | No concerns | Low risk | No concerns | No concerns | No concerns | Major concerns | Moderate |
| GLP1+GIP:Metformin | 0 | Some concerns | Low risk | No concerns | No concerns | No concerns | Major concerns | Low |
| GLP1+GIP:Metformin + DPP4 | 0 | Some concerns | Low risk | No concerns | No concerns | No concerns | Major concerns | Low |
| GLP1+GIP:Zinc | 0 | Some concerns | Low risk | No concerns | No concerns | No concerns | Major concerns | Low |
| GLP1+GIP:diet | 0 | Some concerns | Low risk | No concerns | No concerns | Some concerns | Major concerns | Very low |
| GLP1+GIP:enhanced_education | 0 | Some concerns | Low risk | No concerns | No concerns | No concerns | Major concerns | Low |
| GLP1+GIP:intensive lifestyle intervention | 0 | Some concerns | Low risk | No concerns | No concerns | No concerns | Major concerns | Low |
| GLP1+GIP:lifestyle_intervention_education | 0 | Some concerns | Low risk | No concerns | No concerns | No concerns | Major concerns | Low |
| GLP1+GIP:lipid_low | 0 | Some concerns | Low risk | No concerns | No concerns | No concerns | Major concerns | Low |
| GLP1+GIP:physical_activity | 0 | Some concerns | Low risk | No concerns | No concerns | No concerns | Major concerns | Low |
| GLP1+GIP:probiotic | 0 | Some concerns | Low risk | No concerns | No concerns | No concerns | Major concerns | Low |
| GLP1+GIP:sulfonylureas | 0 | Some concerns | Low risk | No concerns | Some concerns | No concerns | Major concerns | Very low |
| GLP1+GIP:vitamin D | 0 | Some concerns | Low risk | No concerns | No concerns | No concerns | Major concerns | Low |
| Metformin:Zinc | 0 | Some concerns | Low risk | No concerns | No concerns | No concerns | Major concerns | Low |
| Metformin:diet | 0 | Some concerns | Low risk | No concerns | No concerns | No concerns | Major concerns | Low |
| Metformin:enhanced_education | 0 | Some concerns | Low risk | No concerns | No concerns | No concerns | Major concerns | Low |
| Metformin:intensive lifestyle intervention | 0 | Some concerns | Low risk | No concerns | No concerns | No concerns | Major concerns | Low |
| Metformin:physical_activity | 0 | Some concerns | Low risk | No concerns | No concerns | No concerns | Major concerns | Low |
| Metformin:probiotic | 0 | Some concerns | Low risk | No concerns | No concerns | No concerns | Major concerns | Low |
| Metformin:sulfonylureas | 0 | Some concerns | Low risk | No concerns | Some concerns | No concerns | Major concerns | Very low |
| Metformin:vitamin D | 0 | Some concerns | Low risk | No concerns | No concerns | No concerns | Major concerns | Low |
| Metformin + DPP4:Zinc | 0 | Some concerns | Low risk | No concerns | No concerns | No concerns | Major concerns | Low |
| Metformin + DPP4:control | 0 | Some concerns | Low risk | No concerns | No concerns | No concerns | Major concerns | Low |
| Metformin + DPP4:diet | 0 | Some concerns | Low risk | No concerns | No concerns | No concerns | Major concerns | Low |
| Metformin + DPP4:enhanced_education | 0 | Some concerns | Low risk | No concerns | No concerns | No concerns | Major concerns | Low |
| Metformin + DPP4:intensive lifestyle intervention | 0 | Some concerns | Low risk | No concerns | No concerns | No concerns | Major concerns | Low |
| Metformin + DPP4:lifestyle_intervention_education | 0 | Some concerns | Low risk | No concerns | No concerns | Some concerns | Major concerns | Very low |
| Metformin + DPP4:lipid_low | 0 | Some concerns | Low risk | No concerns | No concerns | No concerns | Major concerns | Low |
| Metformin + DPP4:physical_activity | 0 | Some concerns | Low risk | No concerns | No concerns | Some concerns | Major concerns | Very low |
| Metformin + DPP4:probiotic | 0 | Some concerns | Low risk | No concerns | No concerns | Some concerns | Major concerns | Very low |
| Metformin + DPP4:sulfonylureas | 0 | Some concerns | Low risk | No concerns | No concerns | No concerns | Major concerns | Low |
| Metformin + DPP4:vitamin D | 0 | Some concerns | Low risk | No concerns | No concerns | No concerns | Major concerns | Low |
| Zinc:diet | 0 | Some concerns | Low risk | No concerns | No concerns | No concerns | Major concerns | Low |
| Zinc:enhanced_education | 0 | Some concerns | Low risk | No concerns | No concerns | No concerns | Major concerns | Low |
| Zinc:intensive lifestyle intervention | 0 | Some concerns | Low risk | No concerns | No concerns | No concerns | Major concerns | Low |
| Zinc:lifestyle_intervention_education | 0 | Some concerns | Low risk | No concerns | No concerns | No concerns | Major concerns | Low |
| Zinc:lipid_low | 0 | Some concerns | Low risk | No concerns | No concerns | No concerns | Major concerns | Low |
| Zinc:physical_activity | 0 | Some concerns | Low risk | No concerns | No concerns | No concerns | Major concerns | Low |
| Zinc:probiotic | 0 | Some concerns | Low risk | No concerns | No concerns | No concerns | Major concerns | Low |
| Zinc:sulfonylureas | 0 | Some concerns | Low risk | No concerns | No concerns | No concerns | Major concerns | Low |
| Zinc:vitamin D | 0 | Some concerns | Low risk | No concerns | No concerns | No concerns | Major concerns | Low |
| diet:enhanced_education | 0 | Some concerns | Low risk | No concerns | No concerns | No concerns | Major concerns | Low |
| diet:lifestyle_intervention_education | 0 | Some concerns | Low risk | No concerns | No concerns | No concerns | Major concerns | Low |
| diet:lipid_low | 0 | Some concerns | Low risk | No concerns | No concerns | No concerns | Major concerns | Low |
| diet:physical_activity | 0 | Some concerns | Low risk | No concerns | No concerns | No concerns | Major concerns | Low |
| diet:probiotic | 0 | Some concerns | Low risk | No concerns | No concerns | No concerns | Major concerns | Low |
| diet:sulfonylureas | 0 | Some concerns | Low risk | No concerns | No concerns | No concerns | Major concerns | Low |
| diet:vitamin D | 0 | Some concerns | Low risk | No concerns | No concerns | No concerns | Major concerns | Low |
| enhanced_education:intensive lifestyle intervention | 0 | Some concerns | Low risk | No concerns | No concerns | No concerns | Major concerns | Low |
| enhanced_education:lipid_low | 0 | Some concerns | Low risk | No concerns | No concerns | No concerns | Major concerns | Low |
| enhanced_education:physical_activity | 0 | Some concerns | Low risk | No concerns | No concerns | No concerns | Major concerns | Low |
| enhanced_education:probiotic | 0 | Some concerns | Low risk | No concerns | No concerns | No concerns | Major concerns | Low |
| enhanced_education:sulfonylureas | 0 | Some concerns | Low risk | No concerns | No concerns | No concerns | Major concerns | Low |
| enhanced_education:vitamin D | 0 | Some concerns | Low risk | No concerns | No concerns | No concerns | Major concerns | Low |
| intensive lifestyle intervention:lipid_low | 0 | Some concerns | Low risk | No concerns | No concerns | No concerns | Major concerns | Low |
| intensive lifestyle intervention:probiotic | 0 | Some concerns | Low risk | No concerns | No concerns | No concerns | Major concerns | Low |
| intensive lifestyle intervention:sulfonylureas | 0 | Some concerns | Low risk | No concerns | No concerns | No concerns | Major concerns | Low |
| intensive lifestyle intervention:vitamin D | 0 | Some concerns | Low risk | No concerns | No concerns | No concerns | Major concerns | Low |
| lifestyle_intervention_education:lipid_low | 0 | Some concerns | Low risk | No concerns | No concerns | No concerns | Major concerns | Low |
| lifestyle_intervention_education:probiotic | 0 | Some concerns | Low risk | No concerns | No concerns | No concerns | Major concerns | Low |
| lifestyle_intervention_education:sulfonylureas | 0 | Some concerns | Low risk | No concerns | No concerns | No concerns | Major concerns | Low |
| lifestyle_intervention_education:vitamin D | 0 | Some concerns | Low risk | No concerns | No concerns | No concerns | Major concerns | Low |
| lipid_low:physical_activity | 0 | Some concerns | Low risk | No concerns | No concerns | No concerns | Major concerns | Low |
| lipid_low:probiotic | 0 | Some concerns | Low risk | No concerns | No concerns | No concerns | Major concerns | Low |
| lipid_low:sulfonylureas | 0 | Some concerns | Low risk | No concerns | Some concerns | No concerns | Major concerns | Very low |
| lipid_low:vitamin D | 0 | Some concerns | Low risk | No concerns | No concerns | No concerns | Major concerns | Low |
| physical_activity:probiotic | 0 | No concerns | Low risk | No concerns | No concerns | No concerns | Major concerns | Moderate |
| physical_activity:sulfonylureas | 0 | Some concerns | Low risk | No concerns | No concerns | No concerns | Major concerns | Low |
| physical_activity:vitamin D | 0 | Some concerns | Low risk | No concerns | No concerns | No concerns | Major concerns | Low |
| probiotic:sulfonylureas | 0 | Some concerns | Low risk | No concerns | No concerns | No concerns | Major concerns | Low |
| probiotic:vitamin D | 0 | No concerns | Low risk | No concerns | No concerns | No concerns | Major concerns | Moderate |
| sulfonylureas:vitamin D | 0 | Some concerns | Low risk | No concerns | No concerns | No concerns | Major concerns | Low |

32. Table S14 – Risk of biass assessment

Refference

1.	Ackermann, R.T., et al., *A Randomized Comparative Effectiveness Trial for Preventing Type 2 Diabetes.* Am J Public Health, 2015. **105**(11): p. 2328-34.

2.	Admiraal, W.M., et al., *Intensive Lifestyle Intervention in General Practice to Prevent Type 2 Diabetes among 18 to 60-Year-Old South Asians: 1-Year Effects on the Weight Status and Metabolic Profile of Participants in a Randomized Controlled Trial.* PLoS ONE, 2013. **8**(7).

3.	Aekplakorn, W., et al., *Evaluation of a Community-Based Diabetes Prevention Program in Thailand: A Cluster Randomized Controlled Trial.* J Prim Care Community Health, 2019. **10**: p. 2150132719847374.

4.	AkbariRad, M., et al., *Probiotics for glycemic and lipid profile control of the pre-diabetic patients: a randomized, double-blinded, placebo-controlled clinical trial study.* Diabetol Metab Syndr, 2023. **15**(1): p. 71.

5.	Al Thani, M., et al., *The effect of vitamin D supplementation on the glycemic control of pre-diabetic Qatari patients in a randomized control trial.* BMC nutrition, 2019. **5**: p. 46.

6.	Al-Hamdan, R., et al., *Efficacy of lifestyle intervention program for Arab women with prediabetes using social media as an alternative platform of delivery.* J Diabetes Investig, 2021. **12**(10): p. 1872-1880.

7.	Al-Hamdan, R., et al., *Identification of Education Models to Improve Health Outcomes in Arab Women with Pre-Diabetes.* Nutrients, 2019. **11**(5).

8.	Alfawaz, H.A., et al., *Effects of Different Dietary and Lifestyle Modification Therapies on Metabolic Syndrome in Prediabetic Arab Patients: A 12-Month Longitudinal Study.* Nutrients, 2018. **10**(3).

9.	Lindblad, U., et al., *Can sulphonylurea addition to lifestyle changes help to delay diabetes development in subjects with impaired fasting glucose? The Nepi ANtidiabetes StudY (NANSY).* Diabetes Obes Metab, 2011. **13**(2): p. 185-8.

10.	Lindström, J., et al., *Prevention of diabetes mellitus in subjects with impaired glucose tolerance in the Finnish Diabetes Prevention Study: results from a randomized clinical trial.* J Am Soc Nephrol, 2003. **14**(7): p. S108-13.

11.	Lindström, J., et al., *Improved lifestyle and decreased diabetes risk over 13 years: long-term follow-up of the randomised Finnish Diabetes Prevention Study (DPS).* Diabetologia, 2013. **56**(2): p. 284-93.

12.	Liu, L., et al., *Comparing the effects of 12 months aerobic exercise and resistance training on glucose metabolism among prediabetes phenotype: A explorative randomized controlled trial.* Prim Care Diabetes, 2021. **15**(2): p. 340-346.

13.	Liu, X. and G. Wang, *The Effect of High-Intensity Interval Training on Physical Parameters, Metabolomic Indexes and Serum Ficolin-3 Levels in Patients with Prediabetes and Type 2 Diabetes.* Exp Clin Endocrinol Diabetes, 2021. **129**(10): p. 740-749.

14.	Liu, Y., et al., *Short-term effects of lifestyle intervention in the reversion to normoglycemia in people with prediabetes.* Prim Care Diabetes, 2022. **16**(1): p. 168-172.

15.	Luo, X., et al., *Effect of resistance vs. aerobic exercise in pre-diabetes: an RCT.* Trials, 2023. **24**(1): p. 110.

16.	Luo, Y., et al., *A Randomized Controlled Clinical Trial of Lifestyle Intervention and Pioglitazone for Normalization of Glucose Status in Chinese with Prediabetes.* J Diabetes Res, 2022. **2022**: p. 2971382.

17.	Kim, S.H., et al., *Benefits of liraglutide treatment in overweight and obese older individuals with prediabetes.* Diabetes Care, 2013. **36**(10): p. 3276-3282.

18.	Perreault, L., et al., *Changes in Glucose Metabolism and Glycemic Status With Once-Weekly Subcutaneous Semaglutide 2.4 mg Among Participants With Prediabetes in the STEP Program.* Diabetes Care, 2022. **45**(10): p. 2396-2405.

19.	Tao, T., et al., *Exenatide, Metformin, or Both for Prediabetes in PCOS: a Randomized, Open-label, Parallel-group Controlled Study.* Journal of clinical endocrinology and metabolism, 2021. **106**(3): p. e1420‐e1432.

20.	Færch, K., et al., *The effects of dapagliflozin, metformin or exercise on glycaemic variability in overweight or obese individuals with prediabetes (the PRE-D Trial): a multi-arm, randomised, controlled trial.* Diabetologia, 2021. **64**(1): p. 42-55.

21.	Inzucchi, S.E., et al., *Dapagliflozin and the Incidence of Type 2 Diabetes in Patients With Heart Failure and Reduced Ejection Fraction: An Exploratory Analysis From DAPA-HF.* Diabetes Care, 2021. **44**(2): p. 586-594.

22.	Kullmann, S., et al., *Empagliflozin Improves Insulin Sensitivity of the Hypothalamus in Humans With Prediabetes: A Randomized, Double-Blind, Placebo-Controlled, Phase 2 Trial.* Diabetes Care, 2022. **45**(2): p. 398-406.

23.	Ramírez-Rodríguez, A.M., M. González-Ortiz, and E. Martínez-Abundis, *Effect of Dapagliflozin on Insulin Secretion and Insulin Sensitivity in Patients with Prediabetes.* Exp Clin Endocrinol Diabetes, 2020. **128**(8): p. 506-511.

24.	Braun, B., et al., *Metformin modifies the exercise training effects on risk factors for cardiovascular disease in impaired glucose tolerant adults.* Obesity, 2013. **21**(1): p. 93-100.

25.	Caballero, A.E., et al., *The differential effects of metformin on markers of endothelial activation and inflammation in subjects with impaired glucose tolerance: a placebo-controlled, randomized clinical trial.* J Clin Endocrinol Metab, 2004. **89**(8): p. 3943-8.

26.	Gabriel, R., et al., *Reduction in the Risk of Peripheral Neuropathy and Lower Decrease in Kidney Function with Metformin, Linagliptin or Their Fixed-Dose Combination Compared to Placebo in Prediabetes: A Randomized Controlled Trial.* J Clin Med, 2023. **12**(5).

27.	González-Heredia, T., et al., *Effect of Linagliptin Versus Metformin on Glycemic Variability in Patients with Impaired Glucose Tolerance.* Diabetes Technol Ther, 2017. **19**(8): p. 471-475.

28.	Griffin, S.J., et al., *Metformin in non-diabetic hyperglycaemia: the GLINT feasibility RCT.* Health Technol Assess, 2018. **22**(18): p. 1-64.

29.	Guardado-Mendoza, R., et al., *The combination of linagliptin, metformin and lifestyle modification to prevent type 2 diabetes (PRELLIM). A randomized clinical trial.* Metabolism: clinical and experimental, 2020. **104**: p. 154054.

30.	Iqbal Hydrie, M.Z., et al., *Effect of intervention in subjects with high risk of diabetes mellitus in pakistan.* J Nutr Metab, 2012. **2012**: p. 867604.

31.	Knowler, W.C., et al., *Reduction in the incidence of type 2 diabetes with lifestyle intervention or metformin.* N Engl J Med, 2002. **346**(6): p. 393-403.

32.	Lehtovirta, M., et al., *Metabolic effects of metformin in patients with impaired glucose tolerance.* Diabet Med, 2001. **18**(7): p. 578-83.

33.	Li, C.L., et al., *Effect of metformin on patients with impaired glucose tolerance.* Diabet Med, 1999. **16**(6): p. 477-81.

34.	O'Brien, M.J., et al., *PREVENT-DM Comparative Effectiveness Trial of Lifestyle Intervention and Metformin.* Am J Prev Med, 2017. **52**(6): p. 788-797.

35.	Osei, E., et al., *Safety, feasibility and efficacy of metformin and sitagliptin in patients with a TIA or minor ischaemic stroke and impaired glucose tolerance.* BMJ Open, 2021. **11**(9): p. e046113.

36.	Ramachandran, A., et al., *The Indian Diabetes Prevention Programme shows that lifestyle modification and metformin prevent type 2 diabetes in Asian Indian subjects with impaired glucose tolerance (IDPP-1).* Diabetologia, 2006. **49**(2): p. 289-97.

37.	Viskochil, R., et al., *Exercise training and metformin, but not exercise training alone, decreases insulin production and increases insulin clearance in adults with prediabetes.* J Appl Physiol (1985), 2017. **123**(1): p. 243-248.

38.	Wan, Q., et al., *Regression to normoglycaemia by fenofibrate in pre-diabetic subjects complicated with hypertriglyceridaemia: a prospective randomized controlled trial.* Diabet Med, 2010. **27**(11): p. 1312-7.

39.	Wang, Z., et al., *Effects of saxagliptin on glucose homeostasis and body composition of obese patients with newly diagnosed pre-diabetes.* Diabetes Res Clin Pract, 2017. **130**: p. 77-85.

40.	Zhang, L., et al., *Safety and effectiveness of metformin plus lifestyle intervention compared with lifestyle intervention alone in preventing progression to diabetes in a Chinese population with impaired glucose regulation: a multicentre, open-label, randomised controlled trial.* Lancet Diabetes Endocrinol, 2023. **11**(8): p. 567-577.

41.	Zhao, X., et al., *Effects of different doses of metformin treatment for 6 months on aberrant crypt foci in Chinese patients with impaired glucose tolerance.* Eur J Cancer Prev, 2015. **24**(1): p. 27-36.

42.	Badaam, K.M. and U.S. Zingade, *The Effect of Traditional Aerobic Exercise and Sprint Interval Training on Insulin Resistance in Men With Prediabetes: A Randomised Controlled Trial.* Cureus, 2021. **13**(12): p. e20789.

43.	Malin, S.K., et al., *Impact of Short-Term Continuous and Interval Exercise Training on Endothelial Function and Glucose Metabolism in Prediabetes.* J Diabetes Res, 2019. **2019**: p. 4912174.

44.	RezkAllah, S.S. and M.K. Takla, *Effects of Different Dosages of Interval Training on Glycemic Control in People With Prediabetes: A Randomized Controlled Trial.* Diabetes Spectr, 2019. **32**(2): p. 125-131.

45.	Rowan, C.P., et al., *Aerobic Exercise Training Modalities and Prediabetes Risk Reduction.* Med Sci Sports Exerc, 2017. **49**(3): p. 403-412.

46.	Chen, X., et al., *Effects of aerobic training and resistance training in reducing cardiovascular disease risk for patients with prediabetes: a multi-center randomized controlled trial.* Primary care diabetes, 2021. **15**(6): p. 1063‐1070.

47.	Dai, X., et al., *Two-year-supervised resistance training prevented diabetes incidence in people with prediabetes: A randomised control trial.* Diabetes Metab Res Rev, 2019. **35**(5): p. e3143.

48.	Kargarfard, M., et al., *Effects of 12-week moderate aerobic exercise on ROCK2 activity, hs-CRP and glycemic index in obese patients with impaired glucose tolerance.* Physiol Behav, 2022. **257**: p. 113976.

49.	Ma, X., et al., *A randomized controlled trial of Baduanjin exercise to reduce the risk of atherosclerotic cardiovascular disease in patients with prediabetes.* Sci Rep, 2022. **12**(1): p. 19338.

50.	Yan, J., et al., *Effect of 12-Month Resistance Training on Changes in Abdominal Adipose Tissue and Metabolic Variables in Patients with Prediabetes: a Randomized Controlled Trial.* Journal of diabetes research, 2019. **2019**: p. 8469739.

51.	Yuan, X., et al., *Comparing the effects of 6 months aerobic exercise and resistance training on metabolic control and β-cell function in Chinese patients with prediabetes: a multicenter randomized controlled trial.* Journal of diabetes, 2020. **12**(1): p. 25‐37.

52.	Ariel, D., et al., *Effect of liraglutide administration and a calorie-restricted diet on lipoprotein profile in overweight/obese persons with prediabetes.* Nutrition, Metabolism and Cardiovascular Diseases, 2014. **24**(12): p. 1317-1322.

53.	Barclay, C., et al., *Can type 2 diabetes be prevented in UK general practice? A lifestyle-change feasibility study (ISAIAH).* Br J Gen Pract, 2008. **58**(553): p. 541-7.

54.	Barengo, N.C., et al., *Early Lifestyle Interventions in People with Impaired Glucose Tolerance in Northern Colombia: The DEMOJUAN Project.* Int J Environ Res Public Health, 2019. **16**(8).

55.	Ben-Yacov, O., et al., *Personalized Postprandial Glucose Response–Targeting Diet Versus Mediterranean Diet for Glycemic Control in Prediabetes.* Diabetes Care, 2021. **44**(9): p. 1980-1991.

56.	Bennett, W.C., et al., *Effects of exercise amount and intensity versus a combined exercise and lifestyle intervention on metabolic syndrome in adults with prediabetes: a STRRIDE-PD randomized trial.* Front Physiol, 2023. **14**: p. 1199763.

57.	Chen, X., et al., *The Effects of Mobile-App-Based Low-Carbohydrate Dietary Guidance on Postprandial Hyperglycemia in Adults with Prediabetes.* Diabetes therapy, 2020. **11**(10): p. 2341‐2355.

58.	Dodevska, M.S., et al., *Effects of total fibre or resistant starch-rich diets within lifestyle intervention in obese prediabetic adults.* Eur J Nutr, 2016. **55**(1): p. 127-37.

59.	Eriksson, J., et al., *Prevention of Type II diabetes in subjects with impaired glucose tolerance: the Diabetes Prevention Study (DPS) in Finland. Study design and 1-year interim report on the feasibility of the lifestyle intervention programme.* Diabetologia, 1999. **42**(7): p. 793-801.

60.	Errazuriz, I., et al., *Randomized Controlled Trial of a MUFA or Fiber-Rich Diet on Hepatic Fat in Prediabetes.* J Clin Endocrinol Metab, 2017. **102**(5): p. 1765-1774.

61.	Feferman, L., et al., *Carrageenan-Free Diet Shows Improved Glucose Tolerance and Insulin Signaling in Prediabetes: A Randomized, Pilot Clinical Trial.* Journal of Diabetes Research, 2020. **2020**.

62.	Lankinen, M., et al., *Whole grain products, fish and bilberries alter glucose and lipid metabolism in a randomized, controlled trial: the Sysdimet study.* PLoS One, 2011. **6**(8): p. e22646.

63.	Mashayekhi, M., et al., *Weight Loss-Independent Effect of Liraglutide on Insulin Sensitivity in Individuals with Obesity and Pre-Diabetes.* Diabetes, 2023.

64.	Oldroyd, J.C., et al., *Randomised controlled trial evaluating the effectiveness of behavioural interventions to modify cardiovascular risk factors in men and women with impaired glucose tolerance: outcomes at 6 month.* Diabetes research clinical practice, 2001. **52**(1): p. 29‐43.

65.	Palacios, O.M., et al., *Effects of Consuming Almonds on Insulin Sensitivity and Other Cardiometabolic Health Markers in Adults With Prediabetes.* J Am Coll Nutr, 2020. **39**(5): p. 397-406.

66.	Pan, X.R., et al., *Effects of diet and exercise in preventing NIDDM in people with impaired glucose tolerance. The Da Qing IGT and Diabetes Study.* Diabetes Care, 1997. **20**(4): p. 537-44.

67.	Parker, A.R., et al., *The effect of medical nutrition therapy by a registered dietitian nutritionist in patients with prediabetes participating in a randomized controlled clinical research trial.* J Acad Nutr Diet, 2014. **114**(11): p. 1739-48.

68.	Ramachandran, A., et al., *Effectiveness of mobile phone messaging in prevention of type 2 diabetes by lifestyle modification in men in India: a prospective, parallel-group, randomised controlled trial.* The lancet. Diabetes & endocrinology, 2013. **1**(3): p. 191‐198.

69.	Roncero-Ramos, I., et al., *Prediabetes diagnosis criteria, type 2 diabetes risk and dietary modulation: The CORDIOPREV study.* Clin Nutr, 2020. **39**(2): p. 492-500.

70.	Shahbazi, S. and Z. Vahdat Shariatpanahi, *Prevention of type 2 diabetes mellitus by changes in diet among subjects with abnormal glucose metabolism: a randomized clinical trial.* International Journal of Diabetes in Developing Countries, 2018. **38**(1): p. 69-74.

71.	Skoradal, M.B., et al., *Football training improves metabolic and cardiovascular health status in 55- to 70-year-old women and men with prediabetes.* Scand J Med Sci Sports, 2018. **28**: p. 42-51.

72.	Slentz, C.A., et al., *Effects of exercise training alone vs a combined exercise and nutritional lifestyle intervention on glucose homeostasis in prediabetic individuals: a randomised controlled trial.* Diabetologia, 2016. **59**(10): p. 2088-98.

73.	Swinburn, B.A., P.A. Metcalf, and S.J. Ley, *Long-term (5-year) effects of a reduced-fat diet intervention in individuals with glucose intolerance.* Diabetes Care, 2001. **24**(4): p. 619-24.

74.	Turner, K.M., J.B. Keogh, and P.M. Clifton, *Red meat, dairy, and insulin sensitivity: a randomized crossover intervention study.* Am J Clin Nutr, 2015. **101**(6): p. 1173-9.

75.	Utzschneider, K.M., et al., *Small changes in glucose variability induced by low and high glycemic index diets are not associated with changes in β-cell function in adults with pre-diabetes.* J Diabetes Complications, 2020. **34**(8): p. 107586.

76.	Vuksan, V., et al., *Beneficial effects of viscous dietary fiber from Konjac-mannan in subjects with the insulin resistance syndrome: results of a controlled metabolic trial.* Diabetes care, 2000. **23**(1): p. 9‐14.

77.	Wein, P., et al., *A trial of simple versus intensified dietary modification for prevention of progression to diabetes mellitus in women with impaired glucose tolerance.* Aust N Z J Obstet Gynaecol, 1999. **39**(2): p. 162-6.

78.	Wien, M., et al., *Almond consumption and cardiovascular risk factors in adults with prediabetes.* J Am Coll Nutr, 2010. **29**(3): p. 189-97.

79.	Zeng, H., et al., *Glucolipid metabolism improvement in impaired glucose tolerance subjects consuming a Quinoa-based diet: a randomized parallel clinical trial.* Front Physiol, 2023. **14**: p. 1179587.

80.	Stentz, F.B., et al., *High protein diet leads to prediabetes remission and positive changes in incretins and cardiovascular risk factors.* Nutrition, Metabolism and Cardiovascular Diseases, 2021. **31**(4): p. 1227-1237.

81.	Svensson, C.K., et al., *One-year follow-up on liraglutide treatment for prediabetes and overweight/obesity in clozapine- or olanzapine-treated patients.* Acta Psychiatr Scand, 2019. **139**(1): p. 26-36.

82.	Shukla, A.P., et al., *A Randomized Controlled Pilot Study of the Food Order Behavioral Intervention in Prediabetes.* Nutrients, 2023. **15**(20).

83.	Staite, E., et al., *A Wearable Technology Delivering a Web-Based Diabetes Prevention Program to People at High Risk of Type 2 Diabetes: Randomized Controlled Trial.* JMIR Mhealth Uhealth, 2020. **8**(7): p. e15448.

84.	Smith, J.R., et al., *The community-based prevention of diabetes (ComPoD) study: a randomised, waiting list controlled trial of a voluntary sector-led diabetes prevention programme.* International journal of behavioral nutrition and physical activity, 2019. **16**(1): p. 112.

85.	Sutton, E.F., et al., *Early Time-Restricted Feeding Improves Insulin Sensitivity, Blood Pressure, and Oxidative Stress Even without Weight Loss in Men with Prediabetes.* Cell Metab, 2018. **27**(6): p. 1212-1221.e3.

86.	Alfawaz, H., et al., *Improvements in Glycemic, Micronutrient, and Mineral Indices in Arab Adults with Pre-Diabetes Post-Lifestyle Modification Program.* Nutrients, 2019. **11**(11).

87.	Almukhtar, H.M., I.M. Faisal, and M.M. Merkhan, *Acute effect of atorvastatin in comparison with rosuvastatin on glucose homeostasis in hypercholesteremic patients.* Current Topics in Pharmacology, 2021. **25**: p. 25-34.

88.	Alvarez, C., et al., *[Effect of sprint interval training and resistance exercise on metabolic markers in overweight women].* Rev Med Chil, 2012. **140**(10): p. 1289-96.

89.	Amer, O.E., et al., *Reversal of Prediabetes in Saudi Adults: Results from an 18 Month Lifestyle Intervention.* Nutrients, 2020. **12**(3).

90.	Chiasson, J.L., *The effect of acarbose on insulin sensitivity in subjects with impaired glucose tolerance.* Diabet Med, 1996. **13**(3): p. S23-4.

91.	Chiasson, J.L., et al., *Acarbose for prevention of type 2 diabetes mellitus: the STOP-NIDDM randomised trial.* Lancet, 2002. **359**(9323): p. 2072-7.

92.	Chiasson, J.L., et al., *Acarbose for the prevention of Type 2 diabetes, hypertension and cardiovascular disease in subjects with impaired glucose tolerance: facts and interpretations concerning the critical analysis of the STOP-NIDDM Trial data.* Diabetologia, 2004. **47**(6): p. 969-75; discussion 976-7.

93.	Araki, R., et al., *Effect of partially-abraded brown rice consumption on body weight and the indicators of glucose and lipid metabolism in pre-diabetic adults: A randomized controlled trial.* Clinical Nutrition ESPEN, 2017. **19**: p. 9-15.

94.	Aramaki, M., *Alogliptin, but not voglibose, ameliorates dyslipidemia and LDL particle size in patients with impaired glucose tolerance or Type 2 diabetes.* Clinical Lipidology, 2013. **8**(5): p. 533-540.

95.	Arguelles-Tello, F.A., et al., *Metformin improves the weight reduction effect of mazindol in prediabetic obese Mexican subjects.* Int J Clin Pharmacol Ther, 2022. **60**(8): p. 336-345.

96.	Ata, N., et al., *Response of blood sugar fasting to vitamin D3 in individuals with pre-diabetes.* Rawal Medical Journal, 2022. **47**(1): p. 18-21.

97.	Attallah, H., et al., *Effects of growth hormone and pioglitazone in viscerally obese adults with impaired glucose tolerance: a factorial clinical trial.* PLoS Clin Trials, 2007. **2**(5): p. e21.

98.	Attia, J.R., et al., *The effect of zinc supplementation on glucose homeostasis: a randomised double-blind placebo-controlled trial.* Acta diabetologica, 2022. **59**(7): p. 965‐975.

99.	Barthow, C., et al., *A randomised controlled trial of a probiotic and a prebiotic examining metabolic and mental health outcomes in adults with pre-diabetes.* BMJ Open, 2022. **12**(3): p. e055214.

100.	Barzilay, J.I., et al., *Effects of telmisartan on glucose levels in people at high risk for cardiovascular disease but free from diabetes: the TRANSCEND study.* Diabetes Care, 2011. **34**(9): p. 1902-7.

101.	Bauman, V., et al., *Effect of dose of behavioral weight loss treatment on glycemic control in adults with prediabetes.* BMJ Open Diabetes Res Care, 2019. **7**(1): p. e000653.

102.	Bhatt, S.P., et al., *Vitamin D Supplementation in Overweight/obese Asian Indian Women with Prediabetes Reduces Glycemic Measures and Truncal Subcutaneous Fat: A 78 Weeks Randomized Placebo-Controlled Trial (PREVENT-WIN Trial).* Sci Rep, 2020. **10**(1): p. 220.

103.	Bhopal, R.S., et al., *Effect of a lifestyle intervention on weight change in south Asian individuals in the UK at high risk of type 2 diabetes: a family-cluster randomised controlled trial.* The lancet. Diabetes & endocrinology, 2014. **2**(3): p. 218‐227.

104.	Block, G., et al., *Diabetes Prevention and Weight Loss with a Fully Automated Behavioral Intervention by Email, Web, and Mobile Phone: A Randomized Controlled Trial Among Persons with Prediabetes.* J Med Internet Res, 2015. **17**(10): p. e240.

105.	Bloomer, R.J., K.H. Fisher-Wellman, and P.S. Tucker, *Effect of oral acetyl L-carnitine arginate on resting and postprandial blood biomarkers in pre-diabetics.* Nutr Metab (Lond), 2009. **6**: p. 25.

106.	Bock, G., et al., *The effect of DPP-4 inhibition with sitagliptin on incretin secretion and on fasting and postprandial glucose turnover in subjects with impaired fasting glucose.* Clinical Endocrinology, 2010. **73**(2): p. 189-196.

107.	Bone, H., et al., *Effects of pioglitazone on bone in postmenopausal women with impaired fasting glucose or impaired glucose tolerance.* Endocrine Reviews, 2012. **33**(3).

108.	Bosch, J., et al., *Effect of ramipril on the incidence of diabetes.* New England Journal of Medicine, 2006. **355**(15): p. 1551-1562.

109.	Cai, Y., et al., *Effects of Yijinjing combined with resistance training on body fat distribution and hepatic lipids in middle-aged and older people with prediabetes mellitus: A randomized controlled trial.* Exp Gerontol, 2023. **179**: p. 112250.

110.	Califf, R.M., et al., *Effect of valsartan on the incidence of diabetes and cardiovascular events.* New England Journal of Medicine, 2010. **362**(16): p. 1477-1490.

111.	Caviezel, F., A.G. Cattaneo, and G. Pozza, *Long-term administration of acetylsalicylic acid in impaired glucose tolerance in addition to the diet: effects and limits.* Acta Diabetol Lat, 1981. **18**(3): p. 225-33.

112.	Cezaretto, A., et al., *Lifestyle intervention using the psychoeducational approach is associated with greater cardiometabolic benefits and retention of individuals with worse health status.* Arch Endocrinol Metab, 2017. **61**(1): p. 36-44.

113.	Chair, S.Y., et al., *Intermittent Fasting in Weight Loss and Cardiometabolic Risk Reduction: A Randomized Controlled Trial.* J Nurs Res, 2022. **30**(1): p. e185.

114.	Chatterjee, R., et al., *Effects of potassium supplements on glucose metabolism in African Americans with prediabetes: A pilot trial.* American Journal of Clinical Nutrition, 2017. **106**(6): p. 1431-1438.

115.	Chauhan, S., et al., *An Artificial-Intelligence-Discovered Functional Ingredient, NRT_N0G5IJ, Derived from Pisum sativum, Decreases HbA1c in a Prediabetic Population.* Nutrients, 2021. **13**(5).

116.	Chen, M.F., S.L. Hung, and S.L. Chen, *Empowerment Program for People With Prediabetes: A Randomized Controlled Trial.* J Nurs Res, 2017. **25**(2): p. 99-111.

117.	Cicero, A.F.G., et al., *Effects of phytosomal curcumin on anthropometric parameters, insulin resistance, cortisolemia and non-alcoholic fatty liver disease indices: a double-blind, placebo-controlled clinical trial.* Eur J Nutr, 2020. **59**(2): p. 477-483.

118.	Cole, R.E., et al., *Effectiveness of prediabetes nutrition shared medical appointments: prevention of diabetes.* Diabetes Educ, 2013. **39**(3): p. 344-53.

119.	Corpeleijn, E., et al., *Improvements in glucose tolerance and insulin sensitivity after lifestyle intervention are related to changes in serum fatty acid profile and desaturase activities: the SLIM study.* Diabetologia, 2006. **49**(10): p. 2392-401.

120.	Costa, A., et al., *Effects of atorvastatin on glucose homeostasis, postprandial triglyceride response and C-reactive protein in subjects with impaired fasting glucose.* Diabet Med, 2003. **20**(9): p. 743-5.

121.	Davies, M.J., et al., *A community based primary prevention programme for type 2 diabetes integrating identification and lifestyle intervention for prevention: The Let's Prevent Diabetes cluster randomised controlled trial.* Preventive Medicine, 2016. **84**: p. 48-56.

122.	Pittas, A.G., et al., *Vitamin D Supplementation and Prevention of Type 2 Diabetes.* N Engl J Med, 2019. **381**(6): p. 520-530.

123.	de Bie, T.H., et al., *Effects of γ-aminobutyric acid supplementation on glucose control in adults with prediabetes: A double-blind, randomized, placebo-controlled trial.* Am J Clin Nutr, 2023. **118**(3): p. 708-719.

124.	DeFronzo, R.A., et al., *Pioglitazone for diabetes prevention in impaired glucose tolerance.* N Engl J Med, 2011. **364**(12): p. 1104-15.

125.	Derosa, G., et al., *Synergistic effect of doxazosin and acarbose in improving metabolic control in patients with impaired glucose tolerance.* Clinical Drug Investigation, 2006. **26**(9): p. 529-539.

126.	Derosa, G., et al., *Effects of n-3 pufas on fasting plasma glucose and insulin resistance in patients with impaired fasting glucose or impaired glucose tolerance.* Biofactors, 2016. **42**(3): p. 316-22.

127.	Dutta, D., et al., *Vitamin-D supplementation in prediabetes reduced progression to type 2 diabetes and was associated with decreased insulin resistance and systemic inflammation: an open label randomized prospective study from Eastern India.* Diabetes Res Clin Pract, 2014. **103**(3): p. e18-23.

128.	Endevelt, R., et al., *Diabetes prevention program in a Mediterranean environment: individual or group therapy? An effectiveness evaluation.* Prim Care Diabetes, 2015. **9**(2): p. 89-95.

129.	Eriksson, J.G., et al., *Long-term beneficial effects of glipizide treatment on glucose tolerance in subjects with impaired glucose tolerance.* J Intern Med, 2006. **259**(6): p. 553-60.

130.	Everett, B.M., et al., *Anti-Inflammatory Therapy With Canakinumab for the Prevention and Management of Diabetes.* J Am Coll Cardiol, 2018. **71**(21): p. 2392-2401.

131.	Rissanen, A., et al., *Effect of anti-IL-1β antibody (canakinumab) on insulin secretion rates in impaired glucose tolerance or type 2 diabetes: Results of a randomized, placebo-controlled trial.* Diabetes, Obesity and Metabolism, 2012. **14**(12): p. 1088-1096.

132.	Fagerberg, B., et al., *Metabolic effects of controlled-release metoprolol in hypertensive men with impaired or diabetic glucose tolerance: a comparison with atenolol.* J Intern Med, 1990. **227**(1): p. 37-43.

133.	Faghihimani, E., et al., *Reduction of insulin resistance and plasma glucose level by salsalate treatment in persons with prediabetes.* Endocr Pract, 2012. **18**(6): p. 826-33.

134.	Goldfine, A.B., et al., *A randomised trial of salsalate for insulin resistance and cardiovascular risk factors in persons with abnormal glucose tolerance.* Diabetologia, 2013. **56**(4): p. 714-23.

135.	Fischer, H.H., et al., *Text Message Support for Weight Loss in Patients With Prediabetes: A Randomized Clinical Trial.* Diabetes Care, 2016. **39**(8): p. 1364-70.

136.	Florentin, M., et al., *Colesevelam plus rosuvastatin 5 mg/day versus rosuvastatin 10 mg/day alone on markers of insulin resistance in patients with hypercholesterolemia and impaired fasting glucose.* Metab Syndr Relat Disord, 2013. **11**(3): p. 152-6.

137.	Fritsche, A., et al., *Different Effects of Lifestyle Intervention in High- and Low-Risk Prediabetes: Results of the Randomized Controlled Prediabetes Lifestyle Intervention Study (PLIS).* Diabetes, 2021. **70**(12): p. 2785-2795.

138.	Gagnon, C., et al., *A cost-effective moderate-intensity interdisciplinary weight-management programme for individuals with prediabetes.* Diabetes Metab, 2011. **37**(5): p. 410-8.

139.	Gao, F., et al., *[Effect of comprehensive intervention on the diabetes-relevant behaviors in aging patients with prediabetes in rural areas of Yiyang].* Zhong Nan Da Xue Xue Bao Yi Xue Ban, 2017. **42**(10): p. 1198-1203.

140.	Garcia, A.L., et al., *Arabinoxylan fibre consumption improved glucose metabolism, but did not affect serum adipokines in subjects with impaired glucose tolerance.* Hormone and Metabolic Research, 2006. **38**(11): p. 761-766.

141.	Gerstein, H.C., et al., *Impact of Acarbose on Incident Diabetes and Regression to Normoglycemia in People With Coronary Heart Disease and Impaired Glucose Tolerance: Insights From the ACE Trial.* Diabetes Care, 2020. **43**(9): p. 2242-2247.

142.	González-Ortiz, M., et al., *Effect of pioglitazone on insulin secretion in patients with both impaired fasting glucose and impaired glucose tolerance.* Diabetes Res Clin Pract, 2007. **75**(1): p. 115-8.

143.	Gosselin, L.E., L. Chrapowitzky, and T.C. Rideout, *Metabolic effects of α-lipoic acid supplementation in pre-diabetics: a randomized, placebo-controlled pilot study.* Food Funct, 2019. **10**(9): p. 5732-5738.

144.	Gravesteijn, E., R.P. Mensink, and J. Plat, *The effects of long-term almond consumption on whole-body insulin sensitivity, postprandial glucose responses, and 48 h continuous glucose concentrations in males and females with prediabetes: a randomized controlled trial.* Eur J Nutr, 2023. **62**(6): p. 2661-2672.

145.	Griadil, T.I., et al., *PREDICTING PROGRESSION TYPE 2 DIABETES MELLITUS: A 3-YEAR FOLLOW-UP STUDY EXAMINING RISK FACTORS FOR TYPE 2 DIABETES IN PATIENTS WITH PREDIABETES.* Pol Merkur Lekarski, 2023. **51**(3): p. 245-249.

146.	Grizales, A.M., et al., *Metabolic Effects of Betaine: A Randomized Clinical Trial of Betaine Supplementation in Prediabetes.* J Clin Endocrinol Metab, 2018. **103**(8): p. 3038-3049.

147.	Guerrero-Romero, F., et al., *Oral magnesium supplementation improves glycaemic status in subjects with prediabetes and hypomagnesaemia: A double-blind placebo-controlled randomized trial.* Diabetes Metab, 2015. **41**(3): p. 202-7.

148.	Gulati, S., et al., *Premeal almond load decreases postprandial glycaemia, adiposity and reversed prediabetes to normoglycemia: A randomized controlled trial.* Clin Nutr ESPEN, 2023. **54**: p. 12-22.

149.	Gunton, J.E., et al., *Chromium supplementation does not improve glucose tolerance, insulin sensitivity, or lipid profile: a randomized, placebo-controlled, double-blind trial of supplementation in subjects with impaired glucose tolerance.* Diabetes Care, 2005. **28**(3): p. 712-3.

150.	Handelsman, Y., et al., *Colesevelam HCl improves fasting plasma glucose and lipid levels in patients with prediabetes.* Postgrad Med, 2009. **121**(6): p. 62-9.

151.	Handelsman, Y., et al., *Colesevelam hydrochloride to treat hypercholesterolemia and improve glycemia in prediabetes: a randomized, prospective study.* Endocrine practice, 2010. **16**(4): p. 617‐628.

152.	Hansen, E., et al., *Insulin sensitivity after maximal and endurance resistance training.* J Strength Cond Res, 2012. **26**(2): p. 327-34.

153.	Hegde, S.V., et al., *Effect of community-based yoga intervention on oxidative stress and glycemic parameters in prediabetes: a randomized controlled trial.* Complement Ther Med, 2013. **21**(6): p. 571-6.

154.	Heisler, M., et al., *Using Peer Support to Prevent Diabetes: Results of a Pragmatic RCT.* Am J Prev Med, 2023. **65**(2): p. 239-250.

155.	Hellgren, M.I., P.A. Jansson, and U. Lindblad, *Report from an effort to prevent type 2 diabetes development in primary care.* Prim Care Diabetes, 2021. **15**(2): p. 240-244.

156.	Hellgren, M.I., et al., *A lifestyle intervention in primary care prevents deterioration of insulin resistance in patients with impaired glucose tolerance: A randomised controlled trial.* Scand J Public Health, 2016. **44**(7): p. 718-725.

157.	Hernández-Alonso, P., et al., *Beneficial effect of pistachio consumption on glucose metabolism, insulin resistance, inflammation, and related metabolic risk markers: a randomized clinical trial.* Diabetes Care, 2014. **37**(11): p. 3098-105.

158.	Hesselink, A.E., et al., *Effects of a lifestyle program in subjects with Impaired Fasting Glucose, a pragmatic cluster-randomized controlled trial.* BMC Fam Pract, 2015. **16**: p. 183.

159.	Holman, R.R., et al., *Effects of acarbose on cardiovascular and diabetes outcomes in patients with coronary heart disease and impaired glucose tolerance (ACE): a randomised, double-blind, placebo-controlled trial.* The Lancet Diabetes and Endocrinology, 2017. **5**(11): p. 877-886.

160.	Holman, R.R., et al., *Effect of nateglinide on the incidence of diabetes and cardiovascular events.* New England journal of medicine, 2010. **362**(16): p. 1463‐1476.

161.	Honsek, C., et al., *Fibre supplementation for the prevention of type 2 diabetes and improvement of glucose metabolism: the randomised controlled Optimal Fibre Trial (OptiFiT).* Diabetologia, 2018. **61**(6): p. 1295-1305.

162.	Hu, S., et al., *The effect of tai chi intervention on NLRP3 and its related antiviral inflammatory factors in the serum of patients with pre-diabetes.* Front Immunol, 2022. **13**: p. 1026509.

163.	Hu, Z., L. Qin, and H. Xu, *One-Year Results of a Synthetic Intervention Model for the Primary Prevention of T2D among Elderly Individuals with Prediabetes in Rural China.* Int J Environ Res Public Health, 2017. **14**(4).

164.	Huang, F., et al., *Effect of transcutaneous auricular vagus nerve stimulation on impaired glucose tolerance: a pilot randomized study.* BMC complementary and alternative medicine, 2014. **14**: p. 203.

165.	Huang, Y., et al., *Effect of Yijinjing combined with elastic band exercise on muscle mass and function in middle-aged and elderly patients with prediabetes: A randomized controlled trial.* Front Med (Lausanne), 2022. **9**: p. 990100.

166.	Hustad, K.S., et al., *No effect of salmon fish protein on 2-h glucose in adults with increased risk of type 2 diabetes: a randomised controlled trial.* Br J Nutr, 2021. **126**(9): p. 1304-1313.

167.	Islam, M.R., et al., *Zinc supplementation for improving glucose handling in pre-diabetes: A double blind randomized placebo controlled pilot study.* Diabetes Res Clin Pract, 2016. **115**: p. 39-46.

168.	Jorde, R., et al., *Vitamin D 20,000 IU per Week for Five Years Does Not Prevent Progression From Prediabetes to Diabetes.* J Clin Endocrinol Metab, 2016. **101**(4): p. 1647-55.

169.	Kabisch, S., et al., *Fasting Glucose State Determines Metabolic Response to Supplementation with Insoluble Cereal Fibre: A Secondary Analysis of the Optimal Fibre Trial (OptiFiT).* Nutrients, 2019. **11**(10).

170.	Kachuei, A., et al., *Effect of Helicobacter pylori eradication on insulin resistance among prediabetic patients: A pilot study and single-blind randomized controlled clinical trial.* J Res Med Sci, 2016. **21**: p. 8.

171.	Kaku, K., et al., *Sitagliptin improves glycaemic excursion after a meal or after an oral glucose load in Japanese subjects with impaired glucose tolerance.* Diabetes Obes Metab, 2015. **17**(11): p. 1033-41.

172.	Kanaya, A.M., et al., *Glycemic effects of postmenopausal hormone therapy: the Heart and Estrogen/progestin Replacement Study. A randomized, double-blind, placebo-controlled trial.* Ann Intern Med, 2003. **138**(1): p. 1-9.

173.	Karandish, M., et al., *The effect of curcumin and zinc co-supplementation on glycemic parameters in overweight or obese prediabetic subjects: A phase 2 randomized, placebo-controlled trial with a multi-arm, parallel-group design.* Phytother Res, 2021. **35**(8): p. 4377-4387.

174.	Karimi Fard, M., et al., *Alendronate improves fasting plasma glucose and insulin sensitivity, and decreases insulin resistance in prediabetic osteopenic postmenopausal women: A randomized triple-blind clinical trial.* J Diabetes Investig, 2019. **10**(3): p. 731-737.

175.	Kassaian, N., et al., *The effects of probiotics and synbiotic supplementation on glucose and insulin metabolism in adults with prediabetes: a double-blind randomized clinical trial.* Acta Diabetol, 2018. **55**(10): p. 1019-1028.

176.	Katula, J.A., et al., *Effects of a Digital Diabetes Prevention Program: an RCT.* American journal of preventive medicine, 2022. **62**(4): p. 567‐577.

177.	Katula, J.A., et al., *The healthy living partnerships to prevent diabetes study: 2-year outcomes of a randomized controlled trial.* American Journal of Preventive Medicine, 2013. **44**(4): p. S324-S332.

178.	Katula, J.A., et al., *One-year results of a community-based translation of the Diabetes Prevention Program: Healthy-Living Partnerships to Prevent Diabetes (HELP PD) Project.* Diabetes Care, 2011. **34**(7): p. 1451-7.

179.	Kawahara, T., et al., *Effect of active vitamin D treatment on development of type 2 diabetes: DPVD randomised controlled trial in Japanese population.* Bmj, 2022. **377**: p. e066222.

180.	Kawahara, T., et al., *Reduced progression to type 2 diabetes from impaired glucose tolerance after a 2-day in-hospital diabetes educational program: the Joetsu Diabetes Prevention Trial.* Diabetes Care, 2008. **31**(10): p. 1949-54.

181.	Kawamori, R., et al., *[Alpha-glucosidase inhibitor for the prevention of type 2 diabetes mellitus: a randomised, double-blind trial in Japanese subjects with impaired glucose tolerance].* Nihon Rinsho, 2009. **67**(9): p. 1821-5.

182.	Keech, A., et al., *Secondary prevention of cardiovascular events with long-term pravastatin in patients with diabetes or impaired fasting glucose: results from the LIPID trial.* Diabetes Care, 2003. **26**(10): p. 2713-21.

183.	Keerthi, G.S., et al., *Effect of 12 Weeks of Yoga Therapy on Quality of Life and Indian Diabetes Risk Score in Normotensive Indian Young Adult Prediabetics and Diabetics: Randomized Control Trial.* J Clin Diagn Res, 2017. **11**(9): p. Cc10-cc14.

184.	Kentrup, H., et al., *Efficacy and safety of acarbose in patients with cystic fibrosis and impaired glucose tolerance.* European Journal of Pediatrics, 1999. **158**(6): p. 455-459.

185.	Khalilzade, S.H., et al., *Efficacy of bromocriptine on glycemic and metabolic control of prediabetic patients.* Adv Biomed Res, 2015. **4**: p. 253.

186.	Kocher, T., et al., *Effect of Periodontal Treatment on HbA1c among Patients with Prediabetes.* Journal of dental research, 2019. **98**(2): p. 171‐179.

187.	Kosaka, K., M. Noda, and T. Kuzuya, *Prevention of type 2 diabetes by lifestyle intervention: a Japanese trial in IGT males.* Diabetes Res Clin Pract, 2005. **67**(2): p. 152-62.

188.	Kratochvílová, S., et al., *Intramyocellular lipid content in subjects with impaired fasting glucose after telmisartan treatment, a randomised cross-over trial.* Magn Reson Imaging, 2016. **34**(3): p. 353-8.

189.	Kuchay, M.S., et al., *Effect of Vitamin D supplementation on glycemic parameters and progression of prediabetes to diabetes: A 1-year, open-label randomized study.* Indian J Endocrinol Metab, 2015. **19**(3): p. 387-92.

190.	Kulzer, B., et al., *Efficacy of a behavioral lifestyle intervention group program (PREDIAS) for the prevention of type 2 diabetes.* Diabetes, 2009. **58**.

191.	Laaksonen, D.E., et al., *Physical activity in the prevention of type 2 diabetes: the Finnish diabetes prevention study.* Diabetes, 2005. **54**(1): p. 158-65.

192.	Lalla, E., et al., *Six-month outcomes in dental patients identified with hyperglycaemia: a randomized clinical trial.* J Clin Periodontol, 2015. **42**(3): p. 228-35.

193.	Le Roux, C.W., et al., *3 years of liraglutide versus placebo for type 2 diabetes risk reduction and weight management in individuals with prediabetes: a randomised, double-blind trial.* Lancet, 2017. **389**(10077): p. 1399‐1409.

194.	Lee, C.J., et al., *Effects of high-intensity statin combined with telmisartan versus amlodipine on glucose metabolism in hypertensive atherosclerotic cardiovascular disease patients with impaired fasting glucose: A randomized multicenter trial.* Medicine (Baltimore), 2022. **101**(36): p. e30496.

195.	Lee, J.H., et al., *Short-Term Effects of the Internet-Based Korea Diabetes Prevention Study: 6-Month Results of a Community-Based Randomized Controlled Trial.* Diabetes Metab J, 2021. **45**(6): p. 960-965.

196.	Lin, B., et al., *Intensive Blood Pressure Control and Diabetes Mellitus Incidence for Patients with Impaired Fasting Glucose: A Secondary Analysis of SPRINT.* Int J Hypertens, 2023. **2023**: p. 7533353.

197.	Lindahl, B., et al., *A randomized lifestyle intervention with 5-year follow-up in subjects with impaired glucose tolerance: pronounced short-term impact but long-term adherence problems.* Scand J Public Health, 2009. **37**(4): p. 434-42.

198.	Marrero, D.G., et al., *Comparison of Commercial and Self-Initiated Weight Loss Programs in People With Prediabetes: A Randomized Control Trial.* Am J Public Health, 2016. **106**(5): p. 949-56.

199.	Martin, J.S., et al., *Enhanced external counterpulsation improves peripheral artery function and glucose tolerance in subjects with abnormal glucose tolerance.* Journal of Applied Physiology, 2012. **112**(5): p. 868-876.

200.	Mensink, M., et al., *Lifestyle intervention according to general recommendations improves glucose tolerance.* Obes Res, 2003. **11**(12): p. 1588-96.

201.	Meripour, M., H. Mohamadian, and M.A. Khafaie, *Effect of Lifestyle Promotion in the PRECEDE-PROCEED model among pre-diabetic adults based on PERSIAN cohort study: a randomized controlled trial study.* Journal of Diabetes and Metabolic Disorders, 2023.

202.	Misra, P., et al., *A Community Based Randomized Controlled Trial to See the Effect of Vitamin D Supplementation on Development of Diabetes Among Women with Prediabetes Residing in A Rural Community of Northern India.* J Family Med Prim Care, 2021. **10**(8): p. 3122-3129.

203.	Mkrtumyan, A., et al., *A New Approach to Overcome Insulin Resistance in Patients with Impaired Glucose Tolerance: The Results of a Multicenter, Double-Blind, Placebo-Controlled, Randomized Clinical Trial of Efficacy and Safety of Subetta.* J Clin Med, 2022. **11**(5).

204.	MohammadniaMotlagh, K., et al., *Effect of theory-based education on promoting a healthy lifestyle in pre-diabetic women: RCT.* BMC Womens Health, 2022. **22**(1): p. 29.

205.	Monti, L.D., et al., *L-arginine enriched biscuits improve endothelial function and glucose metabolism: a pilot study in healthy subjects and a cross-over study in subjects with impaired glucose tolerance and metabolic syndrome.* Metabolism, 2013. **62**(2): p. 255-64.

206.	Monti, L.D., et al., *Decreased diabetes risk over 9 year after 18-month oral L-arginine treatment in middle-aged subjects with impaired glucose tolerance and metabolic syndrome (extension evaluation of L-arginine study).* Eur J Nutr, 2018. **57**(8): p. 2805-2817.

207.	Morey, M.C., et al., *Enhanced fitness: A randomized controlled trial of the effects of home-based physical activity counseling on glycemic control in older adults with prediabetes mellitus.* Journal of the American Geriatrics Society, 2012. **60**(9): p. 1655-1662.

208.	Moungngern, Y., et al., *Effects of a health promotion program conducted by nurses on stabilization of HBA1C in subjects at risk for diabetes: A phase III randomized controlled trial.* Journal of the Medical Association of Thailand, 2018. **101**(10): p. 1343-1348.

209.	Muilwijk, M., et al., *Effects of a lifestyle intervention programme after 1 year of follow-up among South Asians at high risk of type 2 diabetes: A cluster randomised controlled trial.* BMJ Global Health, 2021. **6**(11).

210.	Nahon, K.J., et al., *Effect of sitagliptin on energy metabolism and brown adipose tissue in overweight individuals with prediabetes: a randomised placebo-controlled trial.* Diabetologia, 2018. **61**(11): p. 2386-2397.

211.	Nanditha, A., et al., *A pragmatic and scalable strategy using mobile technology to promote sustained lifestyle changes to prevent type 2 diabetes in India and the UK: a randomised controlled trial.* Diabetologia, 2020. **63**(3): p. 486-496.

212.	Napoli, N., et al., *Effect of denosumab on fasting glucose in women with diabetes or prediabetes from the FREEDOM trial.* Diabetes Metab Res Rev, 2018. **34**(4): p. e2991.

213.	Nguyen, S.N., et al., *Lifestyle interventions reduce the risk of type II diabetes and cardiovascular diseases development among pre-diabetic adults.* International Journal of Pharmaceutical Research and Allied Sciences, 2021. **10**(2): p. 94-102.

214.	Nijpels, G., et al., *A study of the effects of acarbose on glucose metabolism in patients predisposed to developing diabetes: the Dutch acarbose intervention study in persons with impaired glucose tolerance (DAISI).* Diabetes Metab Res Rev, 2008. **24**(8): p. 611-6.

215.	Niroomand, M., et al., *Does high-dose vitamin D supplementation impact insulin resistance and risk of development of diabetes in patients with pre-diabetes? A double-blind randomized clinical trial.* Diabetes Res Clin Pract, 2019. **148**: p. 1-9.

216.	O'Dea, A., et al., *Can the Onset of Type 2 Diabetes Be Delayed by a Group-Based Lifestyle Intervention in Women with Prediabetes following Gestational Diabetes Mellitus (GDM)? Findings from a Randomized Control Mixed Methods Trial.* J Diabetes Res, 2015. **2015**: p. 798460.

217.	Obara, K., et al., *Isohumulones, the bitter component of beer, improve hyperglycemia and decrease body fat in Japanese subjects with prediabetes.* Clin Nutr, 2009. **28**(3): p. 278-84.

218.	Oh, M.R., et al., *Lactobacillus plantarum HAC01 Supplementation Improves Glycemic Control in Prediabetic Subjects: A Randomized, Double-Blind, Placebo-Controlled Trial.* Nutrients, 2021. **13**(7).

219.	Osei, K., et al., *Beneficial metabolic effects of chronic glipizide in obese African Americans with impaired glucose tolerance: implications for primary prevention of type 2 diabetes.* Metabolism, 2004. **53**(4): p. 414-22.

220.	Page, R.C.L., et al., *Can life-styles of subjects with impaired glucose tolerance be changed? A feasibility study.* Diabetic Medicine, 1992. **9**(6): p. 562-566.

221.	Palermo, A., et al., *Buccal spray insulin in subjects with impaired glucose tolerance: the prevoral study.* Diabetes Obes Metab, 2011. **13**(1): p. 42-6.

222.	Pamidi, S., et al., *Eight Hours of Nightly Continuous Positive Airway Pressure Treatment of Obstructive Sleep Apnea Improves Glucose Metabolism in Patients with Prediabetes. A Randomized Controlled Trial.* Am J Respir Crit Care Med, 2015. **192**(1): p. 96-105.

223.	Pan, C.Y., et al., *Efficacy of acarbose in Chinese subjects with impaired glucose tolerance.* Diabetes Res Clin Pract, 2003. **61**(3): p. 183-90.

224.	Panigrahi, A. and S. Mohanty, *Efficacy and safety of HIMABERB® Berberine on glycemic control in patients with prediabetes: double-blind, placebo-controlled, and randomized pilot trial.* BMC Endocr Disord, 2023. **23**(1): p. 190.

225.	Papakonstantinou, E., et al., *Effects of 6 vs 3 eucaloric meal patterns on glycaemic control and satiety in people with impaired glucose tolerance or overt type 2 diabetes: A randomized trial.* Diabetes Metab, 2018. **44**(3): p. 226-234.

226.	Penn, L., et al., *Prevention of type 2 diabetes in adults with impaired glucose tolerance: the European Diabetes Prevention RCT in Newcastle upon Tyne, UK.* BMC Public Health, 2009. **9**: p. 342.

227.	Perl, S., et al., *Telmisartan improves vascular function independently of metabolic and antihypertensive effects in hypertensive subjects with impaired glucose tolerance.* Int J Cardiol, 2010. **139**(3): p. 289-96.

228.	Peterson, C.M., et al., *Effect of 12 wk of resistant starch supplementation on cardiometabolic risk factors in adults with prediabetes: a randomized controlled trial.* Am J Clin Nutr, 2018. **108**(3): p. 492-501.

229.	Pimentel, G.D., et al., *Long-term nutrition education reduces several risk factors for type 2 diabetes mellitus in Brazilians with impaired glucose tolerance.* Nutrition Research, 2010. **30**(3): p. 186-190.

230.	Pouryousefi, E., et al., *Improved glycemic status, insulin resistance and inflammation after receiving oral oleoylethanolamide supplement in people with prediabetes: a randomized controlled trial.* Diabetol Metab Syndr, 2022. **14**(1): p. 77.

231.	Punthakee, Z., *Cardiovascular and Other Outcomes Postintervention With Insulin Glargine and Omega-3 Fatty Acids (ORIGINALE).* Diabetes Care, 2016. **39**(5): p. 709-16.

232.	Ranasinghe, P., et al., *Zinc supplementation in prediabetes: A randomized double-blind placebo-controlled clinical trial.* J Diabetes, 2018. **10**(5): p. 386-397.

233.	Rasekhi, H., et al., *Phylloquinone supplementation improves glycemic status independent of the effects of adiponectin levels in premonopause women with prediabetes: a double-blind randomized controlled clinical trial.* J Diabetes Metab Disord, 2015. **14**(1): p. 1.

234.	Rezki, A., et al., *Acute and long-term effects of saxagliptin on a set of cardiovascular targets measured at fasting and post-prandially in obese patients with impaired glucose tolerance: A placebo-controlled study.* Nutr Metab Cardiovasc Dis, 2021. **31**(10): p. 2945-2958.

235.	Riddle, M., *Characteristics associated with maintenance of mean A1C<6.5% in people with dysglycemia in the ORIGIN trial.* Diabetes Care, 2013. **36**(10): p. 2915-22.

236.	Rosenstock, J., et al., *Clinical effects of colesevelam in Hispanic subjects with primary hyperlipidemia and prediabetes.* Postgrad Med, 2012. **124**(4): p. 14-20.

237.	Roshanravan, N., et al., *Effect of zinc supplementation on insulin resistance, energy and macronutrients intakes in pregnant women with impaired glucose tolerance.* Iran J Public Health, 2015. **44**(2): p. 211-7.

238.	Saito, T., et al., *Lifestyle modification and prevention of type 2 diabetes in overweight Japanese with impaired fasting glucose levels: a randomized controlled trial.* Archives of internal medicine, 2011. **171**(15): p. 1352‐1360.

239.	Sakane, N., et al., *Prevention of type 2 diabetes in a primary healthcare setting: three-year results of lifestyle intervention in Japanese subjects with impaired glucose tolerance.* BMC public health, 2011. **11**(1): p. 40.

240.	Sakane, N., et al., *Effects of telephone-delivered lifestyle support on the development of diabetes in participants at high risk of type 2 diabetes: J-DOIT1, a pragmatic cluster randomised trial.* BMJ open, 2015. **5**(8): p. e007316.

241.	Sampson, M., et al., *Lifestyle Intervention With or Without Lay Volunteers to Prevent Type 2 Diabetes in People With Impaired Fasting Glucose and/or Nondiabetic Hyperglycemia: a Randomized Clinical Trial.* JAMA internal medicine, 2021. **181**(2): p. 168‐178.

242.	Santos-Lozano, J.M., et al., *Prevention of type 2 diabetes in prediabetic patients by using functional olive oil enriched in oleanolic acid: The PREDIABOLE study, a randomized controlled trial.* Diabetes Obes Metab, 2019. **21**(11): p. 2526-2534.

243.	Sazonov, V., et al., *Effects of niacin on the incidence of new onset diabetes and cardiovascular events in patients with normoglycaemia and impaired fasting glucose.* Int J Clin Pract, 2013. **67**(4): p. 297-302.

244.	Serebrovska, T.V., et al., *Intermittent Hypoxia/Hyperoxia Versus Intermittent Hypoxia/Normoxia: Comparative Study in Prediabetes.* High Alt Med Biol, 2019. **20**(4): p. 383-391.

245.	Sheikhbahaie, F., et al., *The effect of hydroxychloroquine on glucose control and insulin resistance in the prediabetes condition.* Adv Biomed Res, 2016. **5**: p. 145.

246.	Shek, N.W.M., et al., *Lifestyle modifications in the development of diabetes mellitus and metabolic syndrome in Chinese women who had gestational diabetes mellitus: A randomized interventional trial.* Archives of Gynecology and Obstetrics, 2014. **289**(2): p. 319-327.

247.	Shetty, B., et al., *Effect of non-surgical periodontal therapy with adjunct photodynamic therapy on periodontal and glycemic statuses in prediabetic patients with periodontal disease.* Photodiagnosis Photodyn Ther, 2023. **42**: p. 103362.

248.	Shionoiri, H., et al., *Effect of doxazosin therapy on glucose tolerance and lipid metabolism in hypertensive patients with impaired glucose tolerance.* Clinical Therapeutics, 1997. **19**(3): p. 527-536.

249.	Sollid, S.T., et al., *No effect of high-dose vitamin D supplementation on glycemic status or cardiovascular risk factors in subjects with prediabetes.* Diabetes Care, 2014. **37**(8): p. 2123-31.

250.	Spence, J.D., et al., *Pioglitazone Therapy in Patients With Stroke and Prediabetes: A Post Hoc Analysis of the IRIS Randomized Clinical Trial.* JAMA Neurol, 2019. **76**(5): p. 526-535.

251.	Suleman, F., et al., *Effects of delta-tocotrienol supplementation on glycaemic control in individuals with prediabetes: A randomized controlled study.* J Pak Med Assoc, 2022. **72**(1): p. 4-7.

252.	Sussman, J.B., et al., *Improving diabetes prevention with benefit based tailored treatment: risk based reanalysis of Diabetes Prevention Program.* Bmj, 2015. **350**: p. h454.

253.	Suzuki, K., O. Nakagawa, and Y. Aizawa, *Improved early-phase insulin response after candesartan treatment in hypertensive patients with impaired glucose tolerance.* Clin Exp Hypertens, 2008. **30**(5): p. 309-14.

254.	Talaei, A., et al., *The effect of dehydroepiandrosterone on insulin resistance in patients with impaired glucose tolerance.* Hormones (Athens), 2010. **9**(4): p. 326-31.

255.	Tenenbaum, A., et al., *Peroxisome Proliferator-Activated Receptor Ligand Bezafibrate for Prevention of Type 2 Diabetes Mellitus in Patients with Coronary Artery Disease.* Circulation, 2004. **109**(18): p. 2197-2202.

256.	Thirunavukkarasu, D., N.H. Kirubamani, and M.B. Naidu, *The effect of soy flour intake on systemic blood pressure and glycemic control in post-menopausal women with pre-diabetes and prehypertension.* Indian Journal of Pharmaceutical Education and Research, 2017. **51**(2): p. 349-354.

257.	Toprak, O., et al., *Magnesium replacement improve the metabolic profile in obese and pre-diabetic patients with mild-to-moderate chronic kidney disease: A 3-month randomized double-blind placebo-controlled study.* Endocrine Practice, 2017. **23**(1): p. 16A.

258.	Torgerson, J.S., et al., *XENical in the prevention of diabetes in obese subjects (XENDOS) study: a randomized study of orlistat as an adjunct to lifestyle changes for the prevention of type 2 diabetes in obese patients.* Diabetes Care, 2004. **27**(1): p. 155-61.

259.	Toro-Ramos, T., et al., *Mobile Delivery of the Diabetes Prevention Program in People With Prediabetes: Randomized Controlled Trial.* JMIR Mhealth Uhealth, 2020. **8**(7): p. e17842.

260.	Toshimitsu, T., et al., *Effects of 12-Week Ingestion of Yogurt Containing Lactobacillus plantarum OLL2712 on Glucose Metabolism and Chronic Inflammation in Prediabetic Adults: A Randomized Placebo-Controlled Trial.* Nutrients, 2020. **12**(2).

261.	Tuomainen, T.P., et al., *Glucose Metabolism Effects of Vitamin D in Prediabetes: The VitDmet Randomized Placebo-Controlled Supplementation Study.* Journal of Diabetes Research, 2015. **2015**.

262.	Tuomilehto, J., et al., *Prevention of type 2 diabetes mellitus by changes in lifestyle among subjects with impaired glucose tolerance.* N Engl J Med, 2001. **344**(18): p. 1343-50.

263.	Urakaze, M., et al., *The Beneficial Effects of Astaxanthin on Glucose Metabolism and Modified Low-Density Lipoprotein in Healthy Volunteers and Subjects with Prediabetes.* Nutrients, 2021. **13**(12).

264.	Uusitupa, M.I., et al., *Chromium supplementation in impaired glucose tolerance of elderly: effects on blood glucose, plasma insulin, C-peptide and lipid levels.* Br J Nutr, 1992. **68**(1): p. 209-16.

265.	Van Der Zijl, N.J., et al., *Valsartan improves β-cell function and insulin sensitivity in subjects with impaired glucose metabolism: A randomized controlled trial.* Diabetes Care, 2011. **34**(4): p. 845-851.

266.	Van Name, M.A., et al., *Effective Translation of an Intensive Lifestyle Intervention for Hispanic Women With Prediabetes in a Community Health Center Setting.* Diabetes Care, 2016. **39**(4): p. 525-31.

267.	van Poppel, P.C.M., et al., *The interleukin-1 receptor antagonist anakinra improves first-phase insulin secretion and insulinogenic index in subjects with impaired glucose tolerance.* Diabetes, Obesity and Metabolism, 2014. **16**(12): p. 1269-1273.

268.	Vitolins, M.Z., et al., *Long-term Weight Loss Maintenance in the Continuation of a Randomized Diabetes Prevention Translational Study: The Healthy Living Partnerships to Prevent Diabetes (HELP PD) Continuation Trial.* Diabetes Care, 2019. **42**(9): p. 1653-1660.

269.	Von Walden, F., et al., *Humanin skeletal muscle protein levels increase after resistance training in men with impaired glucose metabolism.* FASEB Journal, 2017. **31**(1).

270.	Wallace, H.J., et al., *Effect of vitamin D3 supplementation on insulin resistance and β-cell function in prediabetes: a double-blind, randomized, placebo-controlled trial.* Am J Clin Nutr, 2019. **110**(5): p. 1138-1147.

271.	Wang, J.F., et al., *A combination of omega-3 and plant sterols regulate glucose and lipid metabolism in individuals with impaired glucose regulation: a randomized and controlled clinical trial.* Lipids in health and disease, 2019. **18**(1): p. 106.

272.	Wani, K., et al., *Effects of A 12-Month Intensive Lifestyle Monitoring Program in Predominantly Overweight/Obese Arab Adults with Prediabetes.* Nutrients, 2020. **12**(2).

273.	Weinhold, K.R., et al., *A Randomized Controlled Trial Translating the Diabetes Prevention Program to a University Worksite, Ohio, 2012-2014.* Prev Chronic Dis, 2015. **12**: p. E210.

274.	Wong, C.K., et al., *A short message service (SMS) intervention to prevent diabetes in Chinese professional drivers with pre-diabetes: a pilot single-blinded randomized controlled trial.* Diabetes Res Clin Pract, 2013. **102**(3): p. 158-66.

275.	Wong, C.K.H., et al., *Five-year effectiveness of short messaging service (SMS) for pre-diabetes.* BMC Res Notes, 2018. **11**(1): p. 709.

276.	Xiao, W.Y., et al., *Effects of antihypertensive drugs losartan and levamlodipine besylate on insulin resistance in patients with essential hypertension combined with isolated impaired fasting glucose.* Hypertens Res, 2016. **39**(5): p. 321-6.

277.	Yan, Q., et al., *Probiotics intervention in preventing conversion of impaired glucose tolerance to diabetes: The PPDP follow-on study.* Front Endocrinol (Lausanne), 2023. **14**: p. 1113611.

278.	Yang, L., et al., *Anthocyanins increase serum adiponectin in newly diagnosed diabetes but not in prediabetes: a randomized controlled trial.* Nutr Metab (Lond), 2020. **17**: p. 78.

279.	Yates, T., et al., *Effectiveness of a pragmatic education program designed to promote walking activity in individuals with impaired glucose tolerance: a randomized controlled trial.* Diabetes Care, 2009. **32**(8): p. 1404-10.

280.	Yates, T., et al., *The Pre-diabetes Risk Education and Physical Activity Recommendation and Encouragement (PREPARE) programme study: are improvements in glucose regulation sustained at 2 years?* Diabet Med, 2011. **28**(10): p. 1268-71.

281.	Yeh, M.C., et al., *Translation of the Diabetes Prevention Program for diabetes risk reduction in Chinese immigrants in New York City.* Diabet Med, 2016. **33**(4): p. 547-51.

282.	Yin, Z., et al., *Cultural adaptation of an evidence-based lifestyle intervention for diabetes prevention in Chinese women at risk for diabetes: results of a randomized trial.* Int Health, 2018. **10**(5): p. 391-400.

283.	Yoo, J.Y. and K.S. Yum, *Effect of Coenzyme Q(10) on Insulin Resistance in Korean Patients with Prediabetes: A Pilot Single-Center, Randomized, Double-Blind, Placebo-Controlled Study.* Biomed Res Int, 2018. **2018**: p. 1613247.

284.	Youngwanichsetha, S., S. Phumdoung, and E. Cersosimo, *Effects of a metabolic syndrome self-management programme for women with pre-diabetes.* Focus on Alternative and Complementary Therapies, 2015. **20**(2): p. 74-80.

285.	Yuen, K., et al., *Short-term low-dose growth hormone administration in subjects with impaired glucose tolerance and the metabolic syndrome: effects on beta-cell function and post-load glucose tolerance.* Eur J Endocrinol, 2004. **151**(1): p. 39-45.

286.	Zappe, D.H., et al., *Metabolic and antihypertensive effects of combined angiotensin receptor blocker and diuretic therapy in prediabetic hypertensive patients with the cardiometabolic syndrome.* J Clin Hypertens (Greenwich), 2008. **10**(12): p. 894-903.

287.	Zarrin, R., P. Ayremlou, and F. Ghassemi, *The effect of vitamin D supplementation on the glycemic status and the percentage of body fat mass in adults with prediabetes: A randomized clinical trial.* Iranian Red Crescent Medical Journal, 2017. **19**(3).

288.	Raben, A., et al., *The PREVIEW intervention study: Results from a 3-year randomized 2 x 2 factorial multinational trial investigating the role of protein, glycaemic index and physical activity for prevention of type 2 diabetes.* Diabetes Obes Metab, 2021. **23**(2): p. 324-337.

289.	Rajabi-Naeeni, M., et al., *The effect of omega-3 and vitamin D co-supplementation on glycemic control and lipid profiles in reproductive-aged women with pre-diabetes and hypovitaminosis D: a randomized controlled trial.* Diabetol Metab Syndr, 2020. **12**: p. 41.

290.	Rizos, C.V., et al., *Effects of rosuvastatin combined with olmesartan, irbesartan, or telmisartan on indices of glucose metabolism in Greek adults with impaired fasting glucose, hypertension, and mixed hyperlipidemia: a 24-week, randomized, open-label, prospective study.* Clin Ther, 2010. **32**(3): p. 492-505.

291.	Rodriguez, B.L., et al., *Use of the Dietary Supplement 5-Aminiolevulinic Acid (5-ALA) and Its Relationship with Glucose Levels and Hemoglobin A1C among Individuals with Prediabetes.* Clinical and Translational Science, 2012. **5**(4): p. 314-320.

292.	Sakane, N., et al., *Effect of baseline HbA1c level on the development of diabetes by lifestyle intervention in primary healthcare settings: insights from subanalysis of the Japan Diabetes Prevention Program.* BMJ open diabetes research & care, 2014. **2**(1): p. e000003.

293.	Sakane, N., et al., *Impact of telephone support programme using telemonitoring on stage of change towards healthy eating and active exercise in people with prediabetes.* Journal of telemedicine and telecare, 2021. **27**(5): p. 307‐313.

294.	Basri, S., et al., *Effectiveness of a Web-based Educational Intervention according to the Health Belief Model in Improving Type-2 Diabetes Preventive Behaviors and Blood Sugar Control in Pre-Diabetic Patients in Bushehr.* Iranian Journal of Endocrinology and Metabolism, 2022. **24**(1): p. 12-23.

295.	Xu DF, S.J., Chen M, Chen YQ, Xie H, Sun WJ, Lin YF, Jiang JJ, Sun W, Chen AF, Tang QR, *Effects of lifestyle intervention and meal replacement on glycaemic and body-weight control in Chinese subjects with impaired glucose regulation:*

*a 1-year randomised controlled trial.* Br J Nutr, 2013.

296.	Zong, Y., et al., *[Effects of lifestyle and quantitative nutrition interventions on individuals with prediabetes].* Zhonghua Yi Xue Za Zhi, 2015. **95**(40): p. 3293-6.

297.	Alshehri, F. and F. Javed, *Impact of scaling and root planing on clinical periodontal status and glycemic levels in prediabetic patients.* Interventional medicine & applied science, 2015. **7**(1): p. 17-21.

298.	Bui, T.N., et al., *Pre-germinated brown rice reduced both blood glucose concentration and body weight in Vietnamese women with impaired glucose tolerance.* Journal of nutritional science and vitaminology, 2014. **60**(3): p. 183-187.

299.	Burtscher, M., et al., *Effects of supervised exercise on gamma-glutamyl transferase levels in patients with isolated impaired fasting glucose and those with impaired fasting glucose plus impaired glucose tolerance.* Experimental and clinical endocrinology & diabetes : official journal, German Society of Endocrinology [and] German Diabetes Association, 2012. **120**(8): p. 445-450.

300.	Burtscher, M., et al., *Supervised exercise in patients with impaired fasting glucose: impact on exercise capacity.* Clin J Sport Med, 2009. **19**(5): p. 394-8.

301.	Chuengsamarn, S., et al., *Curcumin Extract for Prevention of Type 2 Diabetes.* Diabetes care, 2012. **35**(11): p. 2121-2127.

302.	Davy, B.M., et al., *Resist diabetes: A randomized clinical trial for resistance training maintenance in adults with prediabetes.* PloS one, 2017. **12**(2): p. e0172610-NA.

303.	de la Luz Alvarez-Canales, M.F., et al., *Effect of linagliptin on glucose metabolism and pancreatic beta cell function in patients with persistent prediabetes after metformin and lifestyle.* Scientific reports, 2021. **11**(1): p. 8750-8750.

304.	Eriksson, J.G., et al., *Aerobic endurance exercise or circuit-type resistance training for individuals with impaired glucose tolerance?* Hormone and metabolic research = Hormon- und Stoffwechselforschung = Hormones et metabolisme, 1998. **30**(1): p. 37-41.

305.	Guess, N., et al., *A Randomised Crossover Trial: The Effect of Inulin on Glucose Homeostasis in Subtypes of Prediabetes.* Annals of nutrition & metabolism, 2015. **68**(1): p. 26-34.

306.	Joseph, R., et al., *Nonsurgical periodontal-therapy improves glycosylated hemoglobin levels in pre-diabetic patients with chronic periodontitis.* World journal of diabetes, 2017. **8**(5): p. 213-221.

307.	Knowler, W.C., et al., *10-year follow-up of diabetes incidence and weight loss in the Diabetes Prevention Program Outcomes Study.* Lancet (London, England), 2009. **374**(9702): p. 1677-1686.

308.	König, D., et al., *A meal replacement regimen improves blood glucose levels in prediabetic healthy individuals with impaired fasting glucose.* Nutrition (Burbank, Los Angeles County, Calif.), 2014. **30**(11): p. 1306-1309.

309.	Rave, K., et al., *Improvement of insulin resistance after diet with a whole-grain based dietary product: results of a randomized, controlled cross-over study in obese subjects with elevated fasting blood glucose.* The British journal of nutrition, 2007. **98**(5): p. 929-936.

310.	Liberopoulos, E., et al., *Effects of manidipine plus rosuvastatin versus olmesartan plus rosuvastatin on markers of insulin resistance in patients with impaired fasting glucose, hypertension, and mixed dyslipidemia.* Journal of cardiovascular pharmacology and therapeutics, 2012. **18**(2): p. 113-118.

311.	McDermott, K., et al., *A yoga intervention for type 2 diabetes risk reduction: a pilot randomized controlled trial.* BMC complementary and alternative medicine, 2014. **14**(1): p. 212-212.

312.	Monti, L.D., et al., *Effect of a long-term oral l-arginine supplementation on glucose metabolism: a randomized, double-blind, placebo-controlled trial.* Diabetes, obesity & metabolism, 2012. **14**(10): p. 893-900.

313.	Oldroyd, J., et al., *Randomised controlled trial evaluating lifestyle interventions in people with impaired glucose tolerance.* Diabetes research and clinical practice, 2005. **72**(2): p. 117-127.

314.	Ramachandran, A., et al., *Pioglitazone does not enhance the effectiveness of lifestyle modification in preventing conversion of impaired glucose tolerance to diabetes in Asian Indians: results of the Indian Diabetes Prevention Programme-2 (IDPP-2).* Diabetologia, 2009. **52**(6): p. 1019-1026.

315.	Rosenstock, J., et al., *Effects of the Dipeptidyl Peptidase-IV Inhibitor Vildagliptin on Incretin Hormones, Islet Function, and Postprandial Glycemia in Subjects With Impaired Glucose Tolerance.* Diabetes care, 2007. **31**(1): p. 30-35.

316.	Saloranta, C., et al., *Nateglinide improves early insulin secretion and controls postprandial glucose excursions in a prediabetic population.* Diabetes care, 2002. **25**(12): p. 2141-2146.

317.	Stentz, F.B., et al., *Remission of pre-diabetes to normal glucose tolerance in obese adults with high protein versus high carbohydrate diet: randomized control trial.* BMJ open diabetes research & care, 2016. **4**(1): p. e000258-NA.

318.	Wang, B., et al., *Effects of a whole rice diet on metabolic parameters and inflammatory markers in prediabetes.* e-SPEN Journal, 2013. **8**(1): p. e15-e20.

319.	Weinstock, T.G., et al., *A controlled trial of CPAP therapy on metabolic control in individuals with impaired glucose tolerance and sleep apnea.* Sleep, 2012. **35**(5): p. 617-625.

320.	Wohl, P., et al., *Effect of telmisartan on selected adipokines, insulin sensitivity, and substrate utilization during insulin-stimulated conditions in patients with metabolic syndrome and impaired fasting glucose.* European journal of endocrinology, 2010. **163**(4): p. 573-583.

321.	Wolever, T.M.S. and C.C. Mehling, *High-carbohydrate–low-glycaemic index dietary advice improves glucose disposition index in subjects with impaired glucose tolerance.* The British journal of nutrition, 2002. **87**(5): p. 477-487.

322.	Röhling, M., et al., *Prediabetes Conversion to Normoglycemia Is Superior Adding a Low-Carbohydrate and Energy Deficit Formula Diet to Lifestyle Intervention-A 12-Month Subanalysis of the ACOORH Trial.* Nutrients, 2020. **12**(7): p. 2022-NA.

323.	Amer, H.M., et al., *OBETICHOLIC ACID AND INSULIN SENSITIVITY IN OVERWEIGHT PATIENTS WITH PREDIABETES.* Obesity and Metabolism, 2024. **21**(2): p. 161-169.

324.	Bai, Z., et al., *Effects of Dorzagliatin, a Glucokinase Activator, on α- and β-Cell Function in Individuals With Impaired and Normal Glucose Tolerance.* Diabetes, 2025. **74**(11): p. 2111-2122.

325.	Basiri, R., et al., *Daily Mango Intake Improves Glycemic and Body Composition Outcomes in Adults with Prediabetes: A Randomized Controlled Study.* Foods, 2025. **14**(17).

326.	Cesar, T., et al., *Citrus flavonoid supplement enhances glycemic and metabolic control in prediabetic patients on metformin: a randomized controlled trial.* Front Nutr, 2025. **12**: p. 1639901.

327.	Chotiprakornkul, N., et al., *Effects of banana blossom supplementation on metabolic parameters in prediabetic adults: a double-blind randomized clinical trial.* Sci Rep, 2025. **15**(1): p. 42519.

328.	Crater, G.D., et al., *Effects of CB1R inverse agonist, INV-202, in patients with features of metabolic syndrome. A randomized, placebo-controlled, double-blind phase 1b study.* Diabetes Obes Metab, 2024. **26**(2): p. 642-649.

329.	Dwibedi, C., et al., *Effect of broccoli sprout extract and baseline gut microbiota on fasting blood glucose in prediabetes: a randomized, placebo-controlled trial.* Nat Microbiol, 2025. **10**(3): p. 681-693.

330.	Guzman, E., et al., *Effects of Diets Containing Beef Compared with Poultry on Pancreatic β-Cell Function and Other Cardiometabolic Health Indicators in Males and Females with Prediabetes: A Randomized, Crossover Trial.* Curr Dev Nutr, 2025. **9**(12): p. 107589.

331.	Hankosky, E.R., et al., *Tirzepatide and the 10-year predicted risk of cardiovascular disease and type 2 diabetes in adults with obesity and prediabetes: A post hoc analysis from the three-year SURMOUNT-1 trial.* Diabetes Obes Metab, 2025. **27**(12): p. 7385-7394.

332.	Jeong, Y., et al., *Efficacy and safety of red ginseng extract powder (KGC05pg) in achieving glycemic control in prediabetic Korean adults A 12-week, single-center, randomized, double-blind, parallel-group, placebo-controlled study.* Medicine (United States), 2024. **103**(52): p. e41130.

333.	Kamali, K., et al., *The effect of barley savigh consumption on blood sugar among pre-diabetic postmenopausal women.* Sci Rep, 2025. **15**(1): p. 23072.

334.	Ma, Y., et al., *Effectiveness of an Individualized Diabetes Health Education Program Using Real-Time Continuous Glucose Monitoring in Improving Blood Glucose: A Pilot Interventional Study on Subjects with Prediabetes.* Diabetes Metab Syndr Obes, 2025. **18**: p. 1939-1948.

335.	Mathioudakis, N., et al., *An AI-Powered Lifestyle Intervention vs Human Coaching in the Diabetes Prevention Program: A Randomized Clinical Trial.* Jama, 2025. **334**(23): p. 2079-2089.

336.	Mes, J.J., et al., *Bitter gourd (Momordica charantia L.) supplementation for twelve weeks improves biomarkers of glucose homeostasis in a prediabetic population.* J Ethnopharmacol, 2025. **347**: p. 119756.

337.	Mohamed, I.A., et al., *Evaluating whole fruit feijoa powder for type 2 diabetes risk prevention: the FERDINAND study*, in *medRxiv*. 2025: J.L. Miles-Chan, Human Nutrition Unit, 18 Carrick Place, Mount Eden, Auckland, New Zealand.

338.	Pickering, E., et al., *Effect of Coccinia grandis (Linn.) Voigt extract on glucose metabolism markers in a prediabetic population: a double-blind randomised clinical trial.* 2023. **3**(4).

339.	Saadat, N., et al., *Does Twice-weekly Cabergoline Improve Anthropometrical and Biochemical Profiles in Prediabetes? A Randomized Double-blind Clinical Trial Pilot Study.* 2015. **14**: p. 77‐86.

340.	Şahin, H., et al., *INVESTIGATING THE THERAPEUTIC POTENTIAL OF (CHENOPODIUM QUINOA) WITH CINNAMON SUPPLEMENTATION AGAINST HYPERGLYCEMIA BIOMARKERS IN PRE-DIABETIC HUMAN FEMALE SUBJECTS.* Journal of Population Therapeutics and Clinical Pharmacology, 2024. **31**(2): p. 2479-2485.

341.	Valder, S., et al., *Effects of two weeks of daily consumption of (poly)phenol-rich red berry fruit juice, with and without high-intensity physical training, on health outcomes in individuals with pre-diabetes mellitus.* Nutr Metab Cardiovasc Dis, 2025. **35**(10): p. 104121.

342.	Zelicha, H., et al., *Effect of cinnamon spice on continuously monitored glycemic response in adults with prediabetes: a 4-week randomized controlled crossover trial.* Am J Clin Nutr, 2024. **119**(3): p. 649-657.

343.	Afshari, A., et al., *The effect of a family health nurse model on blood sugar control in middle-aged prediabetics: a randomized clinical trial (RCT).* BMC Nurs, 2025. **24**(1): p. 1086.

344.	Ahmed, A., et al., *Impact of lifestyle changes on prediabetes remission: Results from the Pakistan diabetes prevention trial.* Diabetes Research and Clinical Practice, 2026. **231**.

345.	Arias-Fernández, M., et al., *Effectiveness of a low-intensity nurse-led lifestyle intervention on glycaemic control in individuals with prediabetes: The PREDIPHONE randomized controlled clinical trial.* Int J Nurs Stud, 2025. **165**: p. 105034.

346.	Baker, C., et al., *Effect of low-volume exercise on hepatic steatosis in adults with obesity plus normal glucose, prediabetes or type 2 diabetes: a randomised controlled trial.* BMJ Open Sport Exerc Med, 2024. **10**(4): p. e001878.

347.	Bhowmik, B., et al., *Faith-Based Lifestyle Intervention for Diabetes Prevention Among Adults in Bangladesh: A Cluster Randomized Clinical Trial.* JAMA Netw Open, 2025. **8**(10): p. e2538101.

348.	Braun, T.S., et al., *Validation of Nutritional Approaches to Modulate Cardiovascular and Diabetic Risk Factors in Patients with Hypertriglyceridemia or Prediabetes-The MoKaRi II Randomized Controlled Study.* Nutrients, 2024. **16**(9).

349.	Brown, S.A., et al., *Transitioning From an In-Person Intervention to Augmented Text Messaging During COVID-19 in Mexican Americans With Prediabetes: The Starr County Diabetes Prevention Randomized Clinical Trial.* Sci Diabetes Self Manag Care, 2024. **50**(2): p. 107-115.

350.	Cakmak, R., et al., *A randomised clinical trial to evaluate a digital therapeutic (TH-001) for improving glycaemic control in adults with prediabetes.* Digit Health, 2025. **11**: p. 20552076251376260.

351.	Chen, X., et al., *Effective of high-intensity interval training and moderate-intensity continuous training on body composition, glycolipid metabolism, and cardiopulmonary function in patients with pre-diabetes: a randomized controlled trial.* Front Endocrinol (Lausanne), 2025. **16**: p. 1614149.

352.	Cheng, W.Y., L.H. Chang, and H.S. Chen, *The effect of statin treatment on glucose homeostasis in prediabetic individuals: A prospective, randomized, controlled trial.* J Chin Med Assoc, 2024. **87**(7): p. 664-669.

353.	Fridén, M., et al., *Effects of an anti-lipogenic low-carbohydrate high polyunsaturated fat diet or a healthy Nordic diet versus usual care on liver fat and cardiometabolic disorders in type 2 diabetes or prediabetes: a randomized controlled trial (NAFLDiet).* Nat Commun, 2025. **16**(1): p. 11130.

354.	Hafeez, S., et al., *Impact of Exercise Manual Program on Biochemical Markers in Sedentary Prediabetic Patients: A Randomized Controlled Trial.* Medicina (Kaunas), 2025. **61**(2).

355.	Hall, C.V., et al., *Effects of a diverse prebiotic fibre supplement on HbA1c, insulin sensitivity and inflammatory biomarkers in pre-diabetes: a pilot placebo-controlled randomised clinical trial.* Br J Nutr, 2024. **132**(1): p. 68-76.

356.	Jadhav, R.A., et al., *Effect of a Customized Physical Activity Promotion Program on Visceral Fat and Glycemic Parameters in Individuals With Prediabetes: A Randomized Controlled Trial.* J Phys Act Health, 2024. **21**(2): p. 164-170.

357.	Jastreboff, A.M., et al., *Tirzepatide for Obesity Treatment and Diabetes Prevention.* N Engl J Med, 2025. **392**(10): p. 958-971.

358.	Karaman, A. and E. Angın, *Effects of clinical Pilates exercises on glycemic control, blood lipids, and physical fitness parameters in women with prediabetes: a randomized controlled trial.* Physiother Theory Pract, 2025: p. 1-14.

359.	Lamichhane, G., et al., *Twelve-Week Curcumin Supplementation Improves Glucose Homeostasis and Gut Health in Prediabetic Older Adults: A Pilot, Double-Blind, Placebo-Controlled Trial.* Nutrients, 2025. **17**(13).

360.	Madhu, S.V., et al., *Yoga and prevention of type 2 diabetes - The Indian Prevention of Diabetes Study (IPDS).* Diabetes Metab Syndr, 2024. **18**(7): p. 103088.

361.	Mari, A., et al., *Tirzepatide Treatment and Associated Changes in β-Cell Function and Insulin Sensitivity in People With Obesity or Overweight With Prediabetes or Normoglycemia: A Post Hoc Analysis From the SURMOUNT-1 Trial.* Diabetes Care, 2025. **48**(9): p. 1622-1627.

362.	Mashayekhi, M., et al., *Weight Loss-Independent Effect of Liraglutide on Insulin Sensitivity in Individuals With Obesity and Prediabetes.* Diabetes, 2024. **73**(1): p. 38-50.

363.	Molani-Gol, R., et al., *Effects of vitamin D supplementation on metabolic factors, serum omentin-1, and anthropometric indices in middle-aged women with prediabetes: a double-blind randomized controlled trial.* Nutr Diabetes, 2025. **15**(1): p. 44.

364.	Pasdar, Y., et al., *Effect of intermittent fasting, portfolio-moderate-carbohydrate, and anti-inflammatory diets on cardio-metabolic status in pre-diabetic patients; an open-label randomized clinical trial.* Diabetol Metab Syndr, 2025. **17**(1): p. 319.

365.	Saboo, N. and S. Kacker, *A Study on Yoga-Based Lifestyle Intervention versus Dietary Intervention Alone on Cardiometabolic Risk Factors among People with Prediabetes.* Ann Afr Med, 2024. **23**(2): p. 202-212.

366.	Sangeetha, M. and A. John, *Comparative Study of Safety, Efficacy of Metformin versus Pioglitazone on HOMA-IR, and HbA1c in prediabetes patient.* 2023. **13**(4): p. 1664‐1670.

367.	Sarabi, M., et al., *Impact of Probiotics Administration on the VEGF, Adiponectin, and Glycolipid Metabolism, in Prediabetic Patients: A Randomized, Double-Blinded, Placebo-Controlled Clinical Trial.* Food Sci Nutr, 2025. **13**(5): p. e70146.

368.	Sawangwong, P., et al., *The Effects of 12-Week Traditional Thai Exercise (Ruesi Dadton) on Glycemic Control and Inflammatory Markers in Prediabetes: a Randomized Controlled Trial.* 2023. **13**(11).

369.	Stice, E., et al., *A preliminary randomized trial of a brief dissonance-based type 2 diabetes prevention programme for adults with pre-diabetes.* Diabetes Obes Metab, 2025. **27**(3): p. 1572-1579.

370.	Sukatta, U., et al., *Synergistic Effects of Microencapsulated Polyphenols and Concurrent Training on Metabolic Health and Fitness in Overweight/Obese Adults with Prediabetes.* Nutrients, 2025. **17**(21).

371.	Jariya Hangsantea, D.A., Pannamas Maneekan, Sompob and N.S. Saralamba, *Effectiveness of Mobile Web Application Promoting Healthy Behaviors among Adults with Prediabetes in Muang District, Phitsanulok Province, Thailand: A Randomized Controlled Trial Study.* 2024.

372.	Toshimitsu, T., et al., *Ingesting probiotic yogurt containing Lactiplantibacillus plantarum OLL2712 improves glycaemic control in adults with prediabetes in a randomized, double-blind, placebo-controlled trial.* Diabetes Obes Metab, 2024. **26**(6): p. 2239-2247.

373.	Vargas, M.C., et al., *Promotora intervention for metabolic and mental health to reduce type 2 diabetes risk: a pilot randomized controlled trial.* Sci Rep, 2024. **14**(1): p. 31988.

374.	Xavier Pi‑Sunyer, M.D., Arne Astrup, M.D., D.M.Sc., Ken Fujioka, M.D., Frank Greenway, M.D.,, M.D. Alfredo Halpern, Michel Krempf, M.D., Ph.D., David C.W. Lau, M.D., Ph.D., Carel W. le Roux, F.R.C.P., Ph.D.,, and M.D. Rafael Violante Ortiz, Christine Bjørn Jensen, M.D., Ph.D., and John P.H. Wilding, D.M., *A Randomized, Controlled Trial of 3.0 mg of Liraglutide in Weight Management.* N Engl J Med, 2015.

375.	Kahn, S.E., et al., *Effect of Semaglutide on Regression and Progression of Glycemia in People With Overweight or Obesity but Without Diabetes in the SELECT Trial.* Diabetes Care, 2024. **47**(8): p. 1350-1359.

376.	Barbara M McGowan, J.M.B., Matt Capehorn, Sue D Pedersen, Kirsi H Pietiläinen, Hanna Angelene Kudiyanur Muniraju, Maria Quiroga, and D.C.W.L. Anette Varbo, *Efficacy and safety of once-weekly semaglutide 2·4 mg versus placebo in people with obesity and prediabetes (STEP 10): a randomised, double-blind, placebo-controlled, multicentre phase 3 trial.* Lancet Diabetes Endocrinol, 2024.

377.	Yiming Mu, X.B., Freddy G Eliaschewitz, Morten Rix Hansen, Bom Taeck Kim, Anna Koroleva, Ronald C W Ma, Tao Yang, Ning Zu, Ming Liu,, *Efficacy and safety of once weekly semaglutide 2·4 mg for weight management in a predominantly east Asian*

*population with overweight or obesity (STEP 7): a double-blind, multicentre, randomised controlled trial.* Lancet Diabetes Endocrinol, 2024.

378.	Garvey, W.T., et al., *Two-year effects of semaglutide in adults with overweight or obesity: the STEP 5 trial.* Nat Med, 2022. **28**(10): p. 2083-2091.
